# Poster presentations

**DOI:** 10.1054/bjoc.2001.1918

**Published:** 2001


*British Journal of Cancer* (2001) **85**(Suppl 1), 33–109
© 2001 Cancer Research Campaign
doi: 10.1054/ bjoc.2001.1918, available online at http://www.idealibrary.com on IDEAL®

### P1 PRODUCTION OF ET-1 AND BIG ET-1 BY HUMAN CELL LINES

C Arun[1], RFL James[1], KE Porter[1], NJM London[1], DM Hemingway[3], [1]Dept of Surgery, University of Leicester, [2]Dept of Oncology, [3]Dept of Surgery, Leicester Royal Infirmary, Leicester LE1 5WW, UK

**Aims** Endothelin, the most potent vasoconstrictor known has been implicated in the development and spread of malignancy. In this study, we assessed the production of endothelin-1 (ET-1) and its precursor big endothelin (big ET-1) by human cancer cell lines.

**Methods** Ten human cancer cell lines were cultured (lung $n = 4$, colorectal $n = 3$, gastro-oesophageal $n = 2$, pancreatic $n = 1$). The culture media were replaced with fresh media after the cells attained confluence. After 48 hours, the conditioned media were batch analysed for ET-1 and big ET-1 by using a sandwich enzyme linked immunoassay (ELISA) (Biomedica, Austria). To elucidate the action of endothelin converting enzyme (ECE), big ET-1 was added to one of the oesophageal cancer cell lines after they attained confluence. Similarly, the media were analysed for the presence of ET-1 and big ET-1

**Results** All the ten cancer cell lines produced ET-1 and nine of ten cancer cell lines produced big ET-1. ET-1 and big ET-1 were not produced in equimolar amounts. The ratio of ET-1 to big ET-1 was 0.56–11.88 (range). All three colorectal cancer cell lines and four of the lung cancer cell lines produced both ET-1 and Big Et-1. Interestingly, the oesophageal cancer cell line that produced high concentrations of ET-1 did not produce any measurable big ET-1. Addition of Big ET-1 into this cell line medium to assess the action of ECE, measuring ET-1 and big ET-1 after 48 hours resulted in complete cleavage of big ET-1 and there was no measurable big ET-1 in the medium.

| | Human Cancer Cell lines | ET-1 (pg/ml per $10^6$ cells)* | Big ET-1 (pg/ml per $10^6$ cells)* |
|---|---|---|---|
| 1 | Lung | 13.9 (0.46–73.1) | 7.9 (2.3–22.2) |
| 2 | Colorectal | 9.2 (2.61–126.6) | 13.2 (4.1–51.1) |
| 3 | Gastro-oesophageal | 70.7 (48.8–92.6) | 5.95 (0–11.9) |
| 4 | Pancreas | 10.36 (9.3–11.3) | 18.6 (16.4–20.1) |

* Expressed as median (range).

**Conclusions** Human cancer cell lines produce both ET-1 and Big ET-1. The difference in ET-1 and big ET-1 production suggests that the ECE activity is variable in different human cancer cell lines.

### P2 PLASMA BIG ET-1 – A TUMOUR MARKER AND SURROGATE MARKER FOR ANGIOGENESIS IN LUNG CANCER

C Arun[1], KE Porter[1], G McMahon[3], NJM London[1], KO'Byrne[2], DM Hemingway[3], [1]Dept of Surgery, University of Leicester, [2]Dept of Oncology, [3]Dept of Surgery, Leicester Royal Infirmary, Leicester, UK

**Introduction** Endothelin-1 (ET-1), a potent vasoactive peptide, is a hypoxia inducible angiogenic growth factor associated with the development and growth of solid tumours. This study was performed to assess whether plasma big endothelin levels (big ET-1), a stable precursor of ET-1, could be used as a tumour marker in lung cancer.

**Patients and methods** The plasma concentration of big ET-1 was measured in 31 patients with proven lung cancers prior to any treatment from February 2000 to December 2000 and in 20 age/sex matched controls. Twenty-three patients had non-small cell lung cancer (NSCLC) and 8, small cell lung cancer (SCLC). EDTA plasma samples were analysed within 2 months of collection using a sandwich enzyme linked immunoassay (Biomedica, Austria).

**Results** Median big ET-1 plasma levels in patients with lung cancer were 4.4 pg/ml, range 0–22.7 pg/ml. These were significantly elevated compared to median big ET-1 plasma levels in controls and were 2.1 pg/ml, range 1.2–13.4 pg/ml (Mann-Whitney test $P = 0.0001$). No significant difference was seen between median plasma levels of big ET-1 in patients with NSCLC 5.0 pg/ml, range 0–22.4 compared to those in patients with SCLC 4.3, range 0–8.8 pg/ml.

| | Groups | Median plasma levels (pg/ml) | Range (pg/ml) | Mann–Whitney |
|---|---|---|---|---|
| 1 | Controls ($n = 20$) | 2.1 | 1.2–13.4 | |
| 2 | Lung Cancer ($n = 31$) | 4.4 | 0.0–22.7 | $P = 0.0001$ |

**Conclusion** Plasma big ET-1 levels were significantly elevated in lung cancer patients. This study indicates that plasma big ET-1 levels should be evaluated as a tumour marker and potential surrogate marker of angiogenesis in this disease.

### P3 PRODUCTION OF ET-1 AND BIG ET-1 BY HUMAN CELL LINES

C Arun*[1], RFL James[1], KE Porter[1], NJM London[1], DM Hemingway[3], [1]Dept of Surgery, University of Leicester, [2]Dept of Oncology, [3]Dept of Surgery, Leicester Royal Infirmary, Leicester LE1 5WW, UK

**Aims** Endothelin, the most potent vasoconstrictor known has been implicated in the development and spread of malignancy. In this study, we assessed the production of endothelin-1 (ET-1) and its precursor big endothelin (big ET-1) by human cancer cell lines.

**Methods** Ten human cancer cell lines were cultured (lung n = 4, colorectal n = 3, gastrooesophageal n = 2, pancreatic n = 1). The culture media were replaced with fresh media after the cells attained confluence. After 48 hours, the conditioned media were batch analysed for ET-1 and big ET-1 by using a sandwich enzyme linked immunoassay (ELISA) (Biomedica, Austria). To elucidate the action of endothelin converting enzyme (ECE), big ET-1 was added to one of the oesophageal cancer cell lines after they attained confluence. Similarly, the media were analysed for the presence of ET-1 and big ET-1

**Results** All the ten cancer cell lines produced ET-1 and nine of ten cancer cell lines produced big ET-1. ET-1 and big ET-1 were not produced in equimolar amounts. The ratio of ET-1 to big ET-1 was 0.56–11.88 (range). All three colorectal cancer cell lines and four of the lung cancer cell lines produced both ET-1 and Big ET-1. Interestingly, the oesophageal cancer cell line that produced high concentrations of ET-1 did not produce any measurable big ET-1. Addition of Big ET-1 into this cell line medium to assess the action of ECE, measuring ET-1 and big ET-1 after 48 hours resulted in complete cleavage of big ET-1 and there was no measurable big ET-1 in the medium.

| | Human Cancer Cell lines | ET-1 (pg/ml per $10^6$ cells)* | Big ET-1 (pg/ml per $10^6$ cells)* |
|---|---|---|---|
| 1 | Lung | 13.9 (0.46–73.1) | 7.9 (2.3–22.2) |
| 2 | Colorectal | 9.2 (2.61–126.6) | 13.2 (4.1–51.1) |
| 3 | Gastro-oesophageal | 70.7 (48.8–92.6) | 5.95 (0–11.9) |
| 4 | Pancreas | 10.36 (9.3–11.3) | 18.6 (16.4–20.1) |

*Expressed as median (range)

**Conclusions** Human cancer cell lines produce both ET-1 and Big ET-1. The difference in ET-1 and big ET-1 production suggests that the ECE activity is variable in different human cancer cell lines.

### P4 MODIFICATIONS TO ENDOTHELIAL TUBULIN BY NITRIC OXIDE (NO) – RESPONSE TO MICROTUBULE-DEPOLYMERISING AGENT, COMBRETASTATIN A-4-P

CS Parkins*, IH Cook, GM Tozer. Tumour Microcirculation Group, Gray Laboratory Cancer Research Trust, Northwood, Middx, UK

Combretastatin A-4-P (CA-4-P) is a novel vascular-targeting agent showing tumour cytotoxicity due to its significant reduction in tumour blood flow. Its mechanism of action is due to its potent microtubule-depolymerising activity, showing selective action against proliferating endothelial cells that occur in tumour vasculature. We have previously reported that NO protects against the anti-vascular actions of CA-4-P in solid tumours (Tozer et al., Cancer Res, 1999; 59: 1626–1634. Parkins et al., Br J Cancer, 2000; 83: 811–6).

Post-translational addition of tyrosine to tubulin, performed by the enzyme tubulin tyrosine ligase (TTL), is a common modification to tubulin conferring changes in protein stability. However, it has been shown that NO-dependent nitration of free tyrosine yields 3-nitrotyrosine (3-NT) which is also a substrate of TTL and becomes irreversibly incorporated into tubulin.

We therefore hypothesise that NO increases the generation of 3-NT, thereby generating an isotype of tubulin with increased resistance to the microtubule-depolymerising agent CA-4-P.

In vitro studies of tyrosine-and 3-NT-modifications to tubulin structure were performed in murine SVEC4-10 endothelial or two tumour cell lines, isolated from tumours showing widely different response to CA-4-P in vivo. Cells were cultured in the absence of tyrosine or 3-NT for 24 h. Subsequent addition of either tyrosine or 3-NT to the media allowed for incorporation into tubulin, which was detected using western blot analysis. Results indicate that tyrosine and 3-NT were incorporated into tubulin protein only, although the incorporation of 3-NT was strongly inhibited by low concentrations of tyrosine. TTL enzyme was detected in all three cell lines.

To model the in vivo situation, cultured endothelial cells were exposed to tumour-conditioned medium, obtained from cloned tumour cell lines isolated from the CA-4-P-responsive tumour. Exposure to this tumour-conditioned medium resulted in significantly reduced incorporation of 3-NT into endothelial tubulin and may account for the relatively greater vascular damage induced by CA-4-P in this tumour type.

Further studies are underway to test the hypothesis that incorporation of 3-NT into tubulin of endothelial cells confers resistance to the microtubule-depolymerising and anti-vascular actions of CA-4-P.

This work is funded by the Cancer Research Campaign.

### P5 COMBRETASTATIN A-4-PHOSPHATE PREVENTS NEUTROPHIL RECRUITMENT TO TNF-α ACTIVATED ENDOTHELIUM UNDER FLOW

AC Brooks[a], GE Nash[b], CS Parkins[a], GM Tozer[a], [a]Tumour Microcirculation Group, Gray Laboratory Cancer Research Trust, Mt Vernon Hospital, Northwood, Middx, [b]Dept. of Physiology, Medical School, University of Birmingham, Birmingham, UK

Combretastatin A-4-phosphate (CA-4-P) is a potent tubulin-binding agent at or near the colchicine-binding site. In vivo it causes vascular shutdown at relatively nontoxic doses in a range of murine and human tumours [Chaplin et al., 1999], and significant infiltration of neutrophils in murine tumours [Parkins et al., 2000]. Adherence of neutrophils to endothelium may be regarded as a stepwise process, characterized by *rolling, adhesion* and *migration*. Previously we have found that CA-4-P directly induces neutrophil recruitment to HUVECs [Unpublished data]. In the present study we have examined the effects of CA-4-P upon the recruitment of neutrophils to TNF- α stimulated endothelium.

HUVECs were seeded into glass capillaries (microslides) and grown under conditions of flow as previously described [Luu et al., 1999]. HUVECs were treated with TNF-α for 4 hours at 37°C, and for the final 30 minutes of this incubation either CA-4-P or colchicine were added. Slides were incorporated in a flow system mounted on a video microscope, and isolated human neutrophils were perfused through the microslides, and images captured in real time. From the video recording, the number of neutrophils falling into each of the 3 categories of adhesion, per field of view was calculated.

Pre-treatment of HUVECs with TNF-α or CA-4-P alone resulted in a dose-dependent increase in neutrophil recruitment compared to control, as previously described. Incubation with colchicine alone also resulted in an increase in neutrophil recruitment. However, incubation of TNF-α stimulated endothelium with CA-4-P or colchicine prior to neutrophil perfusion resulted in an attenuation of neutrophil recruitment compared to TNF-α treated controls.

The data presented demonstrate that under certain circumstances CA-4-P is able to attenuate the recruitment of neutrophils to endothelium. This may therefore have an effect upon neutrophil-related vascular damage following CA-4-P treatment *in vivo*.

Chaplin DJ, Pettit GR, Hill SA (1999). *Anticancer Res* **19**: 189–95

Luu NT, Rainger GE, Nash GB (1999 *J Vasc Res* **36**: 477–85

Parkins CS, Holder AL, Hill SA, Chaplin DJ, Tozer GM (2000) *Br J Cancer* **83**: 811–816

The author is a recipient of a grant from the Association for International Cancer Research.

### P6 INDOLE-ETHER QUINAZOLINES: A NOVEL SERIES OF SELECTIVE AND POTENT VEGF RECEPTOR TYROSINE KINASE INHIBITORS

SR Wedge[1], LF Hennequin[2], DJ Ogilvie[1], J Kendrew[1], M Dukes[1], ESE Stokes[1], D McKerrecher[1], P Plé[2], B Curry[1], [1]Cancer and Infection Research, AstraZeneca, Alderley Park, SK10 4TG, UK, and [2]AstraZeneca Pharma, Centre de Recherches, Z.I. Parc Industriel Pompelle, BP1050, 51689 Reims Cedex 2, France

Inhibition of VEGF-signalling is an attractive anti-tumour target given the apparent pivotal role of VEGF in the regulation of tumour angiogenesis and vascular permeability. Two high-affinity receptors for VEGF with associated tyrosine kinase activity have been identified on human vascular endothelium: VEGF-R1 (Flt-1) and VEGF-R2 (KDR). We are developing inhibitors of VEGF-receptor tyrosine kinase activity which are orally bioavailable and therefore compatible with chronic administration for continual constraint of tumour angiogenesis.

Novel indole ether quinazolines have been identified which are potent inhibitors of Flt-1 and KDR tyrosine kinase activity in isolated enzyme assays in vitro. Subsequent inhibition of VEGF-stimulated proliferation of human umbilical vein endothelial cells (HUVECs) was demonstrated at subnanomolar concentrations ($IC_{50}$ values) with these compounds. Data indicate that the indole ether quinazolines are extremely selective molecules. Selectivity ratios of greater than 1000-fold have been achieved in kinase enzyme assays (e.g. versus EGFR, FGFR and CDK2) and in cell proliferation assays stimulated by different growth factors (e.g. EGF, and FGF). This class of compound demonstrates significant antitumor activity in established Calu-6 lung tumor xenografts following chronic oral once-daily dosing at doses of 1–10 mg/kg/day. These compounds are also known to have broad-spectrum antitumour activity in a range of human tumour xenograft models, which is consistent with the inhibition of VEGF-signalling.

### P7 A MECHANISTIC EXAMINATION OF ZD6474, A VEGF RECEPTOR TYROSINE KINASE INHIBITOR, IN VIVO

J Kendrew[1], SR Wedge[1], DJ Ogilvie[1], M Dukes[1], JO Curwen[1], LF Hennequin[4], ESE Stokes[1], B Curry[1], PF Wadsworth[2], GHP Richmond[2], D Checkley[3] and JC Waterton[3], Departments of [1]Cancer and Infection, [2]Safety Assessment and [3]Enabling Science and Technology, AstraZeneca, Alderley Park, SK10 4TG, UK, and [4]AstraZeneca Pharma, Centre de Recherches, Z.I. Parc Industriel Pompelle, BP1050, 51689 Reims Cedex 2, France

ZD6474 is a low molecular weight inhibitor of KDR tyrosine kinase which demonstrates significant broad-spectrum oral activity in histologically diverse human tumor xenograft models (12.5–100 mg/kg/day, p.o.). ZD6474 can also produce tumor regression in well-established PC-3 prostate tumours (Wedge et al Proc AACR 2000 41: Abstract 3610).

For confirmation of inhibition of VEGF-signalling and angiogenesis *in vivo*, a number of pharmacodynamic endpoints were examined. VEGF has been shown to induce a profound hypotension in anaesthetised rats when administered as a large bolus dose, an effect attributed to a specific signaling response through the growth factor's cognate receptor. ZD6474 (50 mg/kg, p.o.), 2 h prior to VEGF (8 ug, i.v.), inhibited VEGF-induced hypotension by 67% when compared to vehicle-treated controls ($P$ < 0.002 by Anova). Measurement of tumor vascular permeability by dynamic GdDTPA contrast-enhanced MRI was also examined as VEGF is known to have a permeabilising effect on the endothelium. Human prostate tumor xenografts (PC-3, 0.6–1.4 $cm^3$ volume) were established in athymic mice and the tumor vascular permeability surface area product K(trans) determined by GdDTPA-MRI using the kinetic model of Tofts and Kermode (Magn. Res. Med., 17: 357, 1991). ZD6474 (12.5–100 mg/kg, p.o.), or vehicle, was then administered to mice 24 h and 2 h prior to a second measurement of K(trans). Comparison of pre- and post-treatment K(trans) values showed a 28% reduction with 100 and 50 mg/kg ZD6474 and a 14 and 15% reduction with 25 and 12.5 mg/kg respectively. VEGF-driven angiogenesis is also a prerequisite for ossification during long-bone extension. The effect of ZD6474 on growth plate morphology was therefore examined. ZD6474 produced a dose-dependent hypertrophy of the femoro-tibial growth plates of young growing rats, when administered once-daily (p.o.) for 14 days. A dose of 50 mg/kg/day increased the combined growth plate area by 57% ($P$ < 0.001, one-tailed t-test). Similar effects have also been reported with agents which specifically sequester VEGF (e.g. Gerber et al Nat Med 1999 5: 623). These studies support the inhibition of VEGF-signalling in vivo by ZD6474.

### P8 ACTIVITY OF COMBRETASTATIN A1 PHOSPHATE IN MURINE MODELS OF LIVER METASTASIS

SE Holwell, MC Bibby, Cancer Research Unit, University of Bradford, Richmond Rd, Bradford, West Yorkshire, BD7 1DP, UK

The combretastatins are a group of novel antitumour agents derived from the African shrub *Combretum caffrum*. Combretastatin A4 phosphate has previously been shown to demonstrate significant antitumour and antivascular effects in pre-clinical trials and more recently has shown promising results in clinical trials. Combretastatin A1 phosphate, a prodrug of a close structural analogue of combretastatin A4 phosphate, has recently been developed. In order to assess the potential clinical activity of this novel agent, clinically relevant models were required. In this study, syngeneic (MAC 15A) and human xenograft (DLD-1, HT29) models of liver secondaries of colon cancer in mice were developed by direct injection of cells into the liver. These models have been used to compare combretastatin A4 phosphate with the novel analogue combretastatin A1 phosphate. The A1 analogue showed antitumour activity in all three tumours in the subcutaneous site ($P$<0.01) but the A4 analogue, although active against MAC 15A and DLD-1 ($P$<0.01), was less active against HT29 ($P$<0.05). Responses in the more clinically relevant hepatic tumour models were varied. MAC 15A tumours showed no treatment-associated necrosis with either compound at doses that were effective against subcutaneous tumours whereas treatment-associated necrosis was seen in both DLD-1 and HT29. Image analysis indicated that combretastatin A1 phosphate (83% necrosis in HT29, 91% in DLD-1) was more effective in the liver deposits than A4 (18% necrosis in HT29, 22% in DLD-1). MAC 15A failed to vascularised in the liver despite growing to >5 mm in diameter whereas the other two tumours indicate clear vascularisation even when <3mm in diameter. These studies suggest that combretastatins work by an anti-vascular mechanism against colon carcinoma in the murine liver. In addition, the antitumour activity of the compounds was found to differ depending upon tumour site. Perhaps most significantly, results suggest that combretastatin A1 phosphate may have greater potential than A4 for treating metastatic disease.

This work has been funded by War on Cancer and the Cancer Research Campaign.

### P9 HAEM OXYGENASE AND COMBRETASTATIN A4-PHOSPHATE VASCULAR DAMAGE

AF Khelifi[1], VE Prise[1], C Kanthou[1], JE Clark[2], R Foresti[2], R. Motterlini[2] and GM Tozer[1], [1]Tumour Microcirculation Group, Gray Laboratory Cancer Research Trust, Mt Vernon Hospital, Middlesex HA6 2JR, [2]Vascular Biology Unit, Department of Surgical Research, Northwick Park Institute for Medical Research, Middlesex HA1 3UJ, UK

Combretastatin A4-Phosphate (CA-4-P) is a tubulin binding agent which causes prolonged and extensive shut down in tumour blood flow leading to secondary tumour cell death. Phase I clinical trials with this tumour vascular targeting compound have now been completed. Haem oxygenase (HO) catalyses the degradation of haem producing iron, bilirubin and carbon monoxide. HO-1, the inducible form of the enzyme, is thought to play a protective role against various cellular stresses possibly including the vascular injury induced by CA-4-P. In the present study, the effects of CA-4-P on HO activity and protein levels were investigated in vivo, in subcutaneous transplants of the P22 rat carcinosarcoma tumour, and in vitro using the P22 tumour cell line.

Treatment with CA-4-P produced a decrease in total HO activity and HO-I protein levels in P22 tumours in vivo and in P22 tumour cells in culture. This was in contrast to the liver, where CA-4-P stimulated HO-1 production and the kidney where there was no effect. This suggests that HO-1 might play a protective role against CA-4-P induced vascular injury in the liver but not in the tumour.

These preliminary results indicate that there may be a differential in HO-1 production in response to CA-4-P treatment between tumours and normal tissues. It may be possible to exploit this differential and the protective role of HO-1 to improve the therapeutic ratio of this drug.

### P10 REDUCTION IN IMMUNOHISTOCHEMICAL STAINING OF THROMBOSPONDIN-1 PREDICTS PROGRESSION IN SUPERFICIAL BLADDER CANCER

JC Goddard[1], CD Sutton[2], JL Jones[3], KJ O'Byrne[4], RC Kockelbergh[1], Dept. of Urology[1] Surgery[2], Pathology[3] & Oncology[4], University Hospitals of Leicester NHS Trust, UK

**Introduction** Thrombospondin-1 (TSP-1) is a large multifunctional glycoprotein which has been shown to have antiangiogenic functions. Reduced expression has been associated previously with poor outcome in invasive bladder cancer. The purpose of this study was to investigate the relationship of TSP-1 expression and outcome in a series of superficial bladder cancers.

**Methods** A series of 231 cases of superficial bladder cancer were stained with antibody against TSP-1. We assessed the location and percentage of TSP-1 staining. A scoring system based on location and percentage staining was developed and applied to all cases.

**Results** Of the 231 cases, 143 presented as pTa and 88 as pT1. Fifty cases progressed to muscle invasive disease and 181 did not. Immunostaining of TSP-1 was discrete and confined to four distinct areas: Epithelial-Stromal junction, Perivascular, Tumour cell and Stromal. Perivascular staining was present in 26% of cases that progressed compared to 60% that did not ($P = 0.00002$), epithelial-stromal staining was seen in 44% of cases that progressed versus 50% ($P = 0.4$), stromal staining was present in 10% of cases that progressed versus 11% ($P = 0.8$) and tumour cell staining was in 4% of cases that progressed versus 2% of cases that did ($P = 0.5$). The perivascular TSP-1 staining score was significantly lower in cases which progressed to muscle invasive disease ($P = 0.00006$). In a univariate binary logistic regression analysis decreasing perivascular TSP-1 staining was significantly associated with risk of progression (Hazard Ratio 1.74, 95% Cl 1.50–2.03 $P = < 0.000001$). In a multivariate binary logistic regression model, independent predictors of progression were, decreasing perivascular TSP-1 staining ($P = 0.011$) and stage pT1 at presentation ($P < 0.000001$)

**Conclusion** In this series of superficial bladder cancer, a reduction in the immunohistochemical expression of TSP-1 was significantly associated with disease progression to muscle invasion. This is consistent with the hypothesis that TSP-1 is antiangiogenic.

### P11 GLUCOSE TRANSPORTER GLUT-1 EXPRESSION CORRELATES WITH TUMOUR HYPOXIA AND PREDICTS METASTASIS FREE SURVIVAL IN ADVANCED CARCINOMA OF THE CERVIX

R Airley[1,2], J Loncaster[1], M Bromley[1], S Roberts[1], C West[1] & I Stratford[2], [1]CRC Experimental Radiation Oncology Group, Paterson Institute for Cancer Research, [2]Experimental Oncology Group, School of Pharmacy and Pharmaceutical Sciences, University of Manchester, Oxford Road, Manchester M13 9PL, UK

Hypoxic tumours are known to be more malignant, more likely to metastasise and to have a poor prognosis. They are also radio- and chemoresistant. For this reason, it is desirable that a clinically useful marker of hypoxia is found, so that treatment with radiotherapy and bioreductive chemotherapy can be rationally applied to individual patients. Eppendorf histography, by virtue of its proven predictive qualities, has been used in our laboratory to validate markers of hypoxia that can be detected using immunohistochemistry. These markers include antibodies to pimonidazole adducts and intrinsic markers such as the enzyme carbonic anhydrase IX and the glucose transporter GLUT-1. The latter is a facilitative glucose transporter that is ubiquitously expressed in normal tissue and expressed at higher levels in a number of tumours. Its potential as an intrinsic hypoxia marker arises from its dual control in hypoxic conditions by reduced oxidative phosphorylation and the hypoxia-inducible factor (HIF-1) oxygen sensing pathway.

GLUT-1 protein expression has been assessed in individual biopsy sections taken from 54 patients with locally advanced cervical carcinoma, who prior to treatment with radiotherapy were assessed for tumour oxygenation status with the Eppendorf oxygen electrode. By utilising a low-tech scoring system, the intensity of GLUT-1 expression was found to correlate weakly with $pO_2$ ($r = 0.28$, $P = 0.04$). In order to extrapolate this correlation to the known adverse effects of tumour hypoxia on outcome, we examined the prognostic significance of Glut-1 staining in a retrospective series of 121 advanced stage cervical carcinoma treated with radiotherapy, and we found that patients with an absence of Glut-1 significantly increased the likelihood of metastasis-free survival ($P = 0.022$). These findings suggest that Glut-1 may be an intrinsic marker of hypoxia, with prognostic significance, that can easily be applied in a clinical setting.

### P12 EXPRESSION OF HYPOXIA-INDUCIBLE FACTOR 1 IN PRIMARY AND SECONDARY HUMAN OVARIAN CARCINOMAS

C Baluch, B Burke, K Corke, M Wells, CE Lewis, Tumour Targeting Group, Division of Genomic Medicine, University of Sheffield Medical School Sheffield S10 2RX, UK

Patients with epithelial ovarian cancer (EOC) have a survival rate over 5 years of only about 30%. Such a poor prognosis is due to the majority already having local (pelvic and/or peritoneal) or distant metastases at diagnosis. This means that effective treatments need to be developed to halt or slow the development of both the primary and the secondary tumours in EOC. Multiple areas of hypoxia (low oxygen) are common in most forms of malignant tumour and hypoxic tumour cells are relatively resistant to radio-and chemotherapy. Hypoxia stimulates gene expression in mammalian cells via the induction of various transcription factors, including hypoxia-inducible factor-1 (HIF-1) which transactivates cognate binding sites (hypoxia response elements, HRE's) in or near the promoters of oxygen-sensitive genes. Recently, HRE-driven therapeutic DNA constructs have been used to target gene therapy to hypoxic sites (i.e. HIF-1 expressing cells) in human tumour xenografts in mice. This approach may hold particular promise in the selective targeting of secondary tumours in EOC as these have been postulated to be severely hypoxic. Here, we have used immunohistochemistry to demonstrate HIF-1 alpha expression in 22/28 (79%) serous EOC and in virtually all omental deposits examined. Where matched primary/secondary tumour samples were available, secondary tumours expressed similar or more HIF-1 than corresponding primary tumours. Both malignant cells and stromal cells expressed HIF-1, the proportion/extent of which varied between different areas of a given tumour as well as between different tumours. HIF-1 expression was usually maximal in peri-necrotic tumour cells and in areas of rapid growth near to the invasive margin of tumours. In sum, this study demonstrates abundant expression of HIF-1 in both primary and secondary ovarian tumours, suggesting that both may be suitable targets for HRE-regulated gene therapy.

### P13 TUMOUR HYPOXIA AND VASCULARITY IN LYMPHOMA

A Sibtain[1], GD Wilson[2], PJ Hoskin[1], Marie Curie Research Wing, Mount Vernon Hospital, Northwood, Middlesex HA6 2RN, UK

**Introduction** Tumour hypoxia has been shown to occur in a variety of solid tumours and is a factor in limiting treatment outcome. This study examines hypoxia in lymphoma using the Eppendorf microelectrode and the 2-nitroimidazole compound, pimonidazole. The use of pimonidazole with immunohistochemical staining of tissue biopsy specimens for hypoxia demonstrates microregional of hypoxia from which hypoxic fraction can be calculated. Dual staining with CD34 enables analysis of hypoxia in relation to blood vessels.

**Methods and Materials** Twelve patients with accessible lymphoma masses were recruited (five high grade). Five underwent intratumoural oxygen measurements using the Eppendorf probe and tumour biopsy 12–24 hours after intravenous pimonidazole. Adequate biopsies were only obtained in three. Seven patients underwent post pimonidazole biopsy only. Immunohistochemical staining for both pimonidazole and blood vessels was performed on sections of the tissue biopsy samples. A dichromic staining technique was used to demonstrate these characteristics on the same section. Light and dark pimonidazole staining, vessel density and vessel to hypoxia distance were quantified with the aid of an image analysis software package.

**Results** The median $pO_2$ measured by the Eppendorf probe for each mass ranged from 11.2 to 40.4 mmHg. The median hypoxic fraction, defined as the number of readings less than 5mmHg (HF5), was 26 mmHg (range from 0–36%). The median hypoxic fraction, estimated by the proportion of pimonidazole staining, was 0% (range for light staining 0–5%, range for dark staining 0–1.6%). Three of the ten biopsy samples exhibited pimonidazole staining and four of the five tumours that under went Eppendorf measurements showed hypoxia. The median vascular density was 77 vessels per mm2 (range 36–131 vessels/$mm^2$). The median vessel to light staining distance was 87 microns (range 12–203 microns), and to dark staining was 104 microns (range 21–250 microns).

A negative correlation emerged between vascular density and hypoxic fraction measured by pimonidazole ($r$ = –0.9). No correlation emerged between HF5 and hypoxic fraction measured by dark or light pimonidazole, when staining was present ($r$ = 0.13).

**Conclusion** This cohort of lymphomas exhibited little hypoxia when estimated by pimonidazole staining. However a significant hypoxic fraction was seen on Eppendorf probe measurements, although mean $p0_2$ was relatively high compared to data in epithelial solid tumours. Vascular density is inversely proportional to the degree of pimonidazole staining seen.

### P14 ROLE OF NOS IN THE METABOLISM OF PIMONIDAZOLE, A HYPOXIC MARKER

EC Chinje, KJ Williams, SL Lockyer, BA Telfer IJ Stratford, Experimental Oncology Group, School of Pharmacy & Pharmaceutical Sciences, University of Manchester, Oxford Road, Manchester, M13 9PL, UK

Nitric oxide synthase (NOS) represents a relatively unexplored example of an endogenously up-regulated enzyme that could provide tumour selective metabolism of chemotherapeutic prodrugs. Two main features of the enzyme make it a very attractive target to improve cancer therapy. Firstly, it generates nitric oxide (NO), which is important in tumour angiogenesis and maintenance of vascular homeostasis. Of significant importance, elevated levels have been found in several neoplastic tissues. Secondly, its dimeric nature provides two distinct catalytic domains (a reductase and an oxidase) that will bioactivate a broad repertoire of established and novel chemotherapeutic agents. The enzymes are haem-based proteins similar to members of the cytochrome P450 family. In addition, the reductase domain of NOS shares a high degree of sequence homology with cytochrome P450 reductase (P450R), known to be important for the bioactivation of many bioreductive drugs under low oxygen tension (hypoxia). This requirement for a reductive environment refines the specificity of the prodrug target, since hypoxia is a unique characteristic of solid tumours.

We have successfully employed an optimised expression vector in which the human elongation factor 1α promoter produces a bicistronic message containing the genes for human iNOS and puromycin resistance. Following transfection of the human breast cell line, MDA231, clones have been selected that have been demonstrated by immunoblotting and enzyme activity to stably express NOS at levels that have been reported for clinical samples. A 40-fold increase in NOS activity was mirrored by a 4–5 fold increase in reductase activity using cytochrome c as substrate. In this study, we have examined the role of NOS in contributing to the extent of pimonidazole-adducts formation under hypoxia. Parental cells, sense and empty-vector clones were rendered hypoxic for 2 hours prior to addition of the drug and followed by further 3 hour incubation under hypoxia. FACS analysis was then performed with appropriate antibodies to determine the extent of adducts formation.

Preliminary results do indicate that NOS activity may contribute to the overall extent of pimonidazole-adduct formation in these tumour cells under hypoxia. Hence, development and evaluation of our model in vivo will be very useful in evaluating the use of NOS-activated hypoxia-mediated prodrugs as probes in selected patient populations and could represent a gain in the rational application of prodrug chemotherapy.

### P15 NUCLEAR TRANSLOCATION OF THE p65 SUBUNIT OF NFκB IS REGULATED BY WILD-TYPE p53 IN MCF-7 CELLS IN RESPONSE TO HYPOXIA AND REOXYGENATION

CA Parker, CE Lewis, JA Royds, Section of Oncology and Cellular Pathology, Division of Genomic Medicine, University of Sheffield Medical School, Beech Hill Road, Sheffield, S10 2RX, UK

The presence of chronic or transient hypoxia within solid tumours contributes to tumour progression and resistance to therapy. p53 and NFκB transcription factors both respond to multiple stress stimuli, including hypoxia. NFκB has an anti-apoptotic function and promotes proliferation, whereas p53 can induce cell cycle arrest and apoptosis.

This study aimed to identify any interactions between these two transcription factors during the response of MCF-7 cells to hypoxia and reoxygenation.

MCF-7 breast carcinoma cell lines were incubated with control or antisense oligonucleotides directed against wild-type p53 for 24 hours and then exposed to normoxia (21% $O_2$), hypoxia (0.5% $O_2$) or reoxygenation following a 6 hour period of hypoxia. Levels of cellular proliferation and viability were assessed and nuclear extracts assayed for p53 and p65 protein levels by western blotting.

In normoxic conditions, control and antisense treated cells showed very similar growth and protein levels, with no nuclear expression of p53 and p65. Hypoxic antisense treated cells showed increased growth and viability compared to controls. Control cells in hypoxia showed p53 upregulation from 1–24 hours and a window of nuclear p65 at 4 hours. In contrast, antisense treated hypoxic cells lacked p53 expression and exhibited constitutive nuclear p65 expression. During reoxygenation, antisense treated cells had enhanced growth and viability compared to controls. Control and antisense treated cells showed identical protein expression during reoxygenation, with a lack of p53 but with nuclear p65 expressed during 1–24 hours of reoxygenation.

This data demonstrates that antisense inhibition of wild-type p53 leads to constitutive nuclear translocation of p65 in response to hypoxia. When p53 is absent from tumour cells, hypoxia may be able to further promote tumour progression via a prolonged activation of NFκB.

This work is supported by Yorkshire Cancer Research.

### P16 MODIFYING LOW-DOSE HYPER-RADIOSENSITIVITY BY RIBOZYME MEDIATED DOWN-REGULATION OF POLY (ADP-RIBOSE) POLYMERASE

A Chalmers, S Scott, B Marples, M Joiner, Gray Laboratory, Mount Vernon Hospital, Northwood, Middlesex HA6 2JR, UK

**Aim** To elucidate the role of the DNA damage-sensing protein poly(ADP-ribose) polymerase (PARP) in mediating the hypersensitive response of human glioma cell lines to low doses of ionising radiation.

**Background** In many mammalian cell systems, small acute doses of radiation below ~50 cGy are more lethal per unit dose than higher doses, at which radioresistance increases. These dual phenomena have been termed low-dose hyper-radiosensitivity (HRS) and increased radioresistance (IRR). There is evidence that IRR reflects dose-dependent changes in DNA repair mechanisms but the details remain unclear. PARP is a highly-conserved nuclear enzyme activated by binding to DNA strand breaks that has been shown to modulate the activity of key components of the DNA repair system, including p53 and DNA-dependent protein kinase. Abrogation of the IRR response in tumour cell lines by chemical inhibitors of PARP provides evidence that this enzyme plays a key role in the HRS/IRR phenomenon.

**Procedures** To study the role of PARP more specifically, we have generated a dual-expression plasmid vector encoding a ribozyme (short, catalytic RNA species with site-specific cleavage activity) targeted against PARP mRNA, and a green fluorescent protein which allows selection and sorting of transfected cells. The 38 base ribozyme RNA molecule binds to PARP mRNA at a site immediately downstream from the start codon; it is anticipated that cleavage at this site will substantially down-regulate expression of the PARP protein. Negative control vectors encoding the reverse ribozyme sequence and a scrambled version have also been cloned. Using a liposomal transfection agent, vectors were introduced in to T98G human glioma cell lines. After a 48 hour incubation, transfected cells were selected using a fluorescence-activated cell sorter (FACS). Established low-dose cell survival assays of FACS-sorted cells will be used to generate low-dose survival curves. The effect of the ribozyme on PARP protein and activity levels will be assessed by Western blotting and NAD+ incorporation assays respectively.

**Findings** Introduction of active and control vectors in to T98G glioma cells has been achieved, with transfection rates of ~30%. Western blots and cell-survival assays are in progress, and a PARP activity assay is in development. Any changes in the low-dose survival characteristics of the ribozyme-transfected cells, compared with untransfected and negative control-transfected cells, will be examined in relation to changes in levels of PARP expression and activity. Future studies will address the effect of PARP down-regulation in cell lines with similar radiosensitivity but which express varying degrees of the HRS phenomenon. The results will allow elucidation of the role of PARP in mediating low-dose HRS in gliomas.

### P17 EVALUATION OF RH1 AS AN INNOVATIVE BIOREDUCTIVE AGENT AND A POTENTIAL RADIOSENSITIZER IN THE TREATMENT OF SOLID TUMORS J Kim, DP Wilks, CML West,

JY Kim[1], H Valentine[1], DP Wilks, CML West[1], AV Patterson[2], IJ Stratford[2], JH Hendry[1], CRC Group of Experimental Radiation Oncology, PICR, Christie Hospital NHS Trust, Wilmslow Road, Manchester M20 4BX[1], Dept. of Pharmacy & Pharmaceutical Sciences, University of Manchester, Oxford Road, Manchester M13 9PL, UK[2]

One of the most important causes of radiotherapy failure in solid tumor is the presence of hypoxic cells. Bioreductive drugs, which are designed to target radioresistant hypoxic tumor cells has clear rationale to be used in conjunction with radiotherapy. RH1 is an innovative bioreductive agent that was developed in the Paterson Institute as a water-soluble analogue of MeDZQ (2,5-diaziridinyl-3,6-dimethyl-1,4-benzoquinone) and is currently under phase I trials as a joint collaboration between the CRC and the NCI. RH1 cytotoxicity to cancer cells is mainly via bioreductive metabolites that alkylate DNA and lead to the production of inter-strand cross-links[2]. The aim of this study is to investigate the drug's ability to interact with radiation either additively or by a radiosensitizing effect. In order to understand the types and extent of enzymes involved in drug metabolism, several cloned MDA231 breast cancer cell lines have been obtained. These are MDA231 D7, R4 and wild type (WT); the first two of which were infected with DT-diaphorase and P-450 reductase, respectively. In order to determine the radiosensitizing effect of RH1, radiation and drug response are assessed alone and in combination. Cells in an actively metabolizing and proliferating status were used. Radiation survival curves were obtained for the three cell lines and their radiosensitivity assessed as SF2 yielding similar values of 0.38, 0.37 and 0.35 for D7, R4 and WT, respectively. RH1 dose response curves were established for each line by exposing cells to graded doses of RH1 (0–30 nM). RH1 was cytotoxic towards all three cell lines studied with greatest toxicity in D7 cells that have the highest level of DT-diaphorase. The sensitivity of the three cell lines to the drug was shown to be significantly different with $IC_{40–50\%}$ values of 1 nM for D7, 4–6 nM for R4 and WT, respectively. But the magnitude of these differences between lines did not mirror differences in DT-diaphorase (450-fold higher in D7 Vs WT). These results suggest that there should be the roles of other unknown enzymes or individual host factors such as cellular repair. Drug-radiation interaction study was done under aerobic condition for the three cell lines. Radiation and RH1 interaction was investigated in oxic condition using $IC_{90\%}$ drug dose. Regardless of the extent of cell killing by its own, addition of RH1 before irradiation shifted cell survival curve down in parallel way by the amount of the drug effect which suggesting an additive cell killing effect of RH1 with radiation. Future work includes hypoxic cell drug sensitivity and radiation-drug interaction under hypoxic condition. In addition, a clinical study will determine the

**P17 Cont'd**

levels of relevant bioreductive enzymes in tumor and normal tissues taken from patients with tumors. Studies about series of repair protein that is relevant to the damage caused by the drug and radiation will be carried out in parallel. This will provide information on inter-patient heterogeneity in terms of the cytotoxic effect caused by the drug and radiation and so examine the feasibility of using an individualised cancer treatment using RH1.

### P18 THE P53 FUNCTIONAL YEAST ASSAY PREDICTS RADIATION SENSITIVITY IN GASTRO-OESOPHAGEAL CELL LINES

SD Oglesby[1], E Warbrick[1], DA Johnston[2], JF Dillon[2], AJ Munro[1], TR Hupp[2], AM Thompson[1], [1]Dept. Surgery & Mol. Oncology, University of Dundee, [2]Dept. Molecular and Cellular Pathology, University of Dundee, UK

**Introduction** p53 is known to play a key role in determining the cellular response to chemotherapy and radiotherapy, with tumours containing mutant p53 tending to be resistant to treatment. Traditional methods for determining p53 status are either laborious and expensive (sequencing), or correlate poorly with true p53 function (immunohistochemistry). In addition, both methods suffer when tumour material is contaminated with normal tissue as is the case with endoscopic biopsies.

The functional yeast assay (FYA) is a relatively new technique which is simple, and has inherent properties which allow it's use even on biopsy material containing normal tissue. The assay allows for the rapid screening of large numbers of samples. We have used the FYA to assess a number of gastro-oesophageal cell lines and oesophageal cancers, and attempted to correlate the p53 status of the cell lines with their response to clinical doses of radiation.

**Materials and Methods** Cell lines: OE21 (oesophageal squamous carcinoma), OE33 (oesophageal adenocarcinoma), AGS (gastric adenocarcinoma).

Each cell line was irradiated with 2–5 Gy of gamma irradiation, this dosage being similar to that used in many radiotherapy regimes. Evidence of apoptosis or growth arrest were then sought for 24 hours post treatment using flow cytometry.

The p53 status of the cell lines and also of 6 sequentially presenting oesophageal cancers was assessed using FYA.

**Results** The OE21 and OE33 cell lines score as mutant p53 with the FYA, and are resistant to radiation. The AGS cell line contains wild type p53, and apoptoses following 2Gy irradiation. The p53 status of the oesophageal cell lines has not previously been reported. Four of the 6 oesophageal cancers scored as mutant with the FYA.

**Discussion** Chemo-radiotherapy plays an important part in the management of gastro-oesophageal malignancy. Predicting those patients likely to respond would be advantageous. The functional yeast assay is a powerful tool for assessing p53 status in human tumours, we have shown that it can be applied to biopsy material from gastro-oesophageal tumours. FYA predicts response in-vitro, further studies are required to determine whether it predicts response in-vivo.

### P19 RADIOSENSITIZATION BY NOVEL INHIBITORS OF PARP, PI, AND DNA-PK, NU7026, IN VITRO.

S Veuger*[1], BW Durkacz[1], BT Golding[1], RJ Griffin[1], N Martin[1], DR Newell[1], L Rigoreau[1], M Stockley[1], SE Webber[2], Z Hostomsky[2] and NJ Curtin[1], University of Newcastle upon Tyne UK[1], Agouron Pharmaceuticals, San Diego, USA[2]

Cell lines deficient in poly(ADP-ribose) polymerase (PARP) or DNA dependent protein kinase (DNA-PK) are unable to carry out DNA single strand break (SSB) and double strand break (DSB) repair, respectively, making them highly radio- and chemo-sensitive. Inhibitors of these enzymes should therefore have potential as radio- and chemo-sensitizers for cancer therapy. Radiosensitization by the novel PARP and DNA-PK inhibitors, PI and NU7026, respectively, was investigated in Chinese hamster ovary cells: DNA-PK deficient V3 cells and their DNA-PK proficient counterpart, V3 YAC (transfected with a yeast artificial chromosome containing human DNA-PKcs). NU7026 was neither growth inhibitory (sulphorhodamine B (SRB) assay) nor cytotoxic (clonogenic assay) per se at concentrations $\leq 20$ μM in either cell line. Exponentially growing V3 YAC cells were sensitized to IR by 0.4 μM PI and 10 μM NU7026 (1.4 and 1.5-fold, respectively) with greater than additive effects (2.8-fold sensitization) of the drugs in combination, being observed. In the V3 cells, PI caused a 1.4-fold sensitization whilst no radiosensitization was observed with NU7026, confirming that the primary intracellular target of NU7026 is DNA-PK. Recovery from potentially lethal damage (PLDR) was investigated in growth arrested V3 and V3 YAC cells. G1 arrest in plateau phase cells was confirmed by flow cytometric analysis. V3-Yac and V3 cells were exposed to equitoxic doses of γ-IR (6Gy and 1.4GY respectively) and following a 24 hr delay in cloning, which allows quiescent cells to repair PLD, the surviving fraction of V3 YAC cells increased 8–10 fold but survival of V3 cells only increased 1–2 fold. In the V3 YAC cells this PLD recovery was reduced by approx. 70% by inclusion of PI during the recovery period whilst NU7026 abolished this recovery. Co-incubation with both inhibitors reduced survival to below that of cells that had not been allowed to recover. The modest recovery seen in V3 cells was not inhibited by NU7026 but was fully inhibited by PI. The mechanism underlying the effects of PI and NU7026 were investigated by measuring DNA DSB levels by neutral elution. Following exposure of V3 YAC cells to 75 GYγ IR the majority of DNA strand break rejoining (~90%) occurred within 60 min. Repair was inhibited by 35% by PI and 55% by NU7026, and the two drugs in combination completely inhibited rejoining. In DNA-PK deficient V3 cells only about 50% of the DNA DSB had rejoined by 60 min in the absence of drug, As expected, NU7026 had no effect on rejoining but PI (alone or in combination with NU7026) inhibited rejoining by 50–60%. These studies are consistent with PARP and DNA-PK activity being required for the repair of and recovery from radiation induced DNA damage and that radiosensitization can be achieved by inhibition of these enzymes.

### P20 A RAPID DUAL-FLUORESCENCE DNA MISREPAIR ASSAY FOR PREDICTING RADIOSENSITIVITY IN UROLOGICAL CANCER CELL LINES

Sangar VK[1,3], Collis SJ[1], Cowan R[2], Roberts SA[1], Hendry JH[1], Margison GP[1], Clarke NW[2,3], CRC/Paterson Institute for Cancer Research[1], Christie Hospital[2], Manchester, M20 4BX & Hope Hospital[3], Salford, M6 8HD, UK

**Introduction** Radiotherapy is an important treatment modality for bladder and prostate cancer. Cellular radiosensitivity is related to incorrect repair (misrepair) of DNA double-strand breaks. This study reports the use of a novel Rapid Dual-Fluorescence (RDF) Assay for DNA misrepair in urological cancers.

**Methods** A circular DNA plasmid, pREVY, containing the genes encoding enhanced green fluorescent protein (EGFP) and enhanced yellow fluorescent protein (EYFP) was linearised using a restriction endonuclease that cleaves between the enhancer and promoter regions controlling EGFP expression. The circular (control) and linear plasmids were individually transfected into tumour cell lines of differing radiosensitivity: prostate (LNCaP) and bladder (MGHU-1 and S40b). The DNA misrepair was quantified using flow cytometry and computer analysis of the shift in the intensities of the two fluorescent proteins. The colony-forming assay was used to produce radiation survival curves from which a measure of radiosensitivity, the alpha parameter, was recorded.

**Results** The alpha values for LNCaP, MGHU-1 and S40b were 0.35, 0.025 and 0.17 respectively. The corresponding DNA misrepair values as measured by the above assay were 83.4%, 48.2% and 39.9%. A trend between percentage misrepair and the alpha value was observed ($R$ = 0.72, $P$ = 0.02), with the more radiosensitive cells showing greater misrepair.

**Conclusion** The RDF assay, which takes only three days to perform, is a promising new technique for measuring DNA misrepair and may enable assessment of radiosensitivity in prostate and bladder cancers prior to treatment. (Supported in part by The CRC)

### P21 DIFFERENTIAL RADIOSENSITISATION OF INTRA-TUMOUR CELL POPULATIONS BY GEMCITABINE

Sangar VK[1,3], Ramani VAC[2], Cowan R[2], Roberts SA[1], Hendry JH[1], Margison GP[1], Clarke NW[2,3], CRC/Paterson Institute for Cancer Research[1], Christie Hospital[2], Manchester, M204BX & Hope Hospital[3], Salford, M6 8HD, UK

**Introduction** Gemcitabine is a nucleoside analogue which has known radiosensitising properties in certain tumours. Its effect is highly cell type and schedule dependent. Here we highlight its radiosensitising effects in bladder cancer cells in vitro and assess the differential intra-tumour activity by studying two related bladder tumour cell lines with different radiosensitivities.

**Methods & materials** Two invasive bladder tumour cell lines were studied, the radioresistant line MGHU-1 (parent) and its radiosensitive clone S40b. Standard clonogenic assays were undertaken to establish the radiosenistivities of both cell lines. In order to screen these cell lines for the radiosensitising effects of Gemcitabine the MTT assay was utilised. Each cell line was treated with three different doses of Gemcitabine (0.01, 0.25 and 0.5 umol for MGHU-1 and 0.01, 0.35 and 0.5 umol for S40b) at three different time points prior to radiotherapy (48, 24 and 12 hours; 0–6 Gy). After approximately six doublings viable fractions were calculated and survival curves were fitted to the LQ model. Using unwieghted non-linear least-squares regression those time points where there was possible synergy between Gemcitabine and radiation were highlighted.

**Results** The clonogenic survival curves confirmed the radiosensitivities of both cell lines with SF2s of 0.90 and 0.64; and alpha values of 0.025 and 0.18 for MGHU-1 and S40b respectively. Synergy between Gemcitabine and radiation was achieved with 0.01 umol of Gemcitabine given at 48 and 12 hours prior to radiation in the S40b cell line ($P$ < 0.001 at 48 hrs, $P$ = 0.021 at 12 hrs). Further evidence of radiosensitisation was noted at 0.35 umol (S40b) Gemcitabine given 12 hrs prior to radiation ($P$ = 0.021). No significant synergy was evident in MGHU-1.

**Conclusion** Gemcitabine radiosensitises bladder tumour cell lines. The results suggest that its effects are greater in those tumour cell populations which are more radiosensitive.

### P22 DO OXIDATIVE DNA LESIONS ACCUMULATE IN BRAIN TISSUE IN XERODERMA PIGMENTOSUM?

AE Kiltie[1], JL Ravanat,[3] SW Wijnhoven[2], H Vrieling[2], J Cadet[3], T Lindahl[1], [1]Department of Mutagenesis, ICRF Clare Hall Laboratories, EN6 3LD, UK, [2]Leiden University Medical Centre, 2333 AL, Netherlands, [3]CEA Grenoble, F-38054, France

Some xeroderma pigmentosum (XP) patients (20%) suffer from neurodegeneration, which may be caused by free-radical induced DNA damage produced during endogenous cellular metabolism. XP patients are deficient in nucleotide excision repair (NER). Forms of DNA damage such as those causing major helix distortion, are repaired by NER rather than by the base excision repair (BER) pathway, which corrects most endogenous DNA damage. Cyclopurine deoxynucleoside lesions are formed by free-radicals in hypoxic conditions. Lesions are produced in which the base and 2′deoxyribose are joined by an extra covalent bond, and this prevents DNA glycosylases from releasing such adducts. These lesions are highly efficient blocking lesions to DNA polymerases and to 3′ exonucleases and are therefore cytotoxic.

As cyclopurine lesions are repaired by NER but not BER, it is hypothesised that cyclopurines may accumulate in XP brains to cause neurodegeneration.

Organs have been obtained from old (1–2 years old) and young mice with *XPA*-and *XPC*-deficiencies. Preliminary work has been carried out using tissue from normal mice. Genomic DNA was extracted from tissues (brain, liver, kidney, skin, spleen, heart, lung, testes) using a sodium iodide method to minimise oxidative damage during DNA processing. DNA was then digested to nucleosides, using nuclease P1, phosphodiesterases I and II, and alkaline phosphatase. Preliminary (non-XP) brain tissue samples spiked with a 22mer oligonucleotide containing a single 5′, 8-cyclo-2′deoxyadenosine lesion (5′R diastereoisomer; 5′cyclodAdo) have been analysed using hypersensitive HPLC and tandem mass spectrometry (HPLC MS/MS). No cyclopurine lesions were detected in brain tissue from normal mice, but 5′cyclodAdo was detected in the spiked samples in expected amounts.

Once the sensitivity of the HPLC MS/MS technique has been optimised, tissue from old and young *XPA*-and *XPC*-deficient mice and normal young and old controls will be analysed for the presence of cyclopurine deoxynucleotides.

### P23 HYPOFRACTIONATED PALLIATIVE RADIOTHERAPY IN ADULT GLIOBLASTOMA MULTIFORME

VR Bulusu, SG Russell, K Burton, G Robinson, NG Burnet, Oncology centre, Addenbrooke's Hospital, Hill's road, Cambridge, CB2 2QQ, UK

**Introduction** Glioblastoma multiforme (GBM) in adults carries a poor prognosis. In elderly or poor performance status patients (pts), a short course of radiotherapy may be appropriate for palliation.

**Aim** To assess the overall survival and subjective response to a hypofractionated 2 weeks course of palliative radiotherapy (RT) in adult GBM pts treated at Addenbrooke's hospital from 1998–2000.

**Materials and Methods** 40 adult pts with a histologically proven GBM have been irradiated with 30 Gy in six fractions over 2 weeks using 6MV photons (27 Gy if 260 kV) using a parallel pair cut down whole brain radiotherapy technique. Age, WHO performance status, duration of symptoms, presence or absence of fits, site of tumour, details of neurosurgical procedure (NSP), subjective response, overall survival (from the date of neurosurgical procedure) and post radiotherapy survival were recorded. The mean, median, 95% CI and p values for significance in difference in means were calculated.

**Results** $N$ = 40, Males = 20, Females = 20, WHO PS ≤ 1 = 15 and ≥ 2 = 25. Mean age 64.3 yrs (Range 41–76 years). 83% had no history of fits at presentation. Partial debulking was carried out in 50% pts. Symptoms were stable or improved after RT in 75% of pts. RT was tolerated with minimal side effects in the majority of patients. The mean overall survival (OS) from NSP for the whole group was 22.3 weeks (17–27.6, 95% CI). The mean OS for the good and poor PS groups was 35.7 weeks (25.7–45.7, 95%CI) and 14 weeks (11–17, 95%CI) respectively ($P$ < 0.05). In poor PS (WHO ≥ 2), the mean post RT survival was only 9.5 weeks (6.5 – 12.5, 95% CI).

| Duration | Mean (weeks) | 95%CI | Median (weeks) |
|---|---|---|---|
| Symptoms | 5.8 | 4.7–6.9 | 5 |
| NSP to Consult | 1.6 | 1.2–2 | 1.1 |
| Consult to RT | 1.8 | 1.5–2.1 | 1.5 |
| End of RT-death | 17.4 | 12.1–22.7 | 17.1 |

**Conclusions** Hypofractionated RT in adult pts with GBM is well tolerated and offers either stabilization or improvement of symptoms in 75% of the patients. WHO PS is a strong prognostic factor for survival. No significant correlation between other clinical variables and survival was seen. For poor PS adult pts with GBM best supportive care might be a suitable alternative to RT though this may have to be evaluated in a randomized clinical trial.

### P24 ZD1839 ('IRESSA'), AN EGFR-TKI, ENHANCES THE ANTITUMOUR EFFECT OF RADIOTHERAPY IN A HUMAN COLORECTAL CARCINOMA XENOGRAFT MODEL

KJ Williams[1], BA Telfer[1], IJ Stratford[1], SR Wedge[2], [1]School of Pharmacy, The University of Manchester, Manchester, M13 9PL, UK, [2]Cancer and Infection Bioscience, AstraZeneca, Alderley Park, Macclesfield, Cheshire, SK10 4TG, UK

ZD1839 is an orally active epidermal growth factor receptor tyrosine kinase inhibitor (EGFR-TKI) that has been demonstrated to block EGFR-mediated mitogenic signaling pathways that promote the proliferation and survival of cancer cells. ZD1839 has demonstrated promising efficacy and tolerability in Phase I clinical studies. Recently, ionising radiation has been implicated as a potential stimulus for EGFR signaling. Hence, we have evaluated the possible therapeutic benefit of ZD1839 in combination with radiotherapy. The effect ZD1839 in combination with single and fractionated dose radiotherapy was analysed in two separate experiments. In each case, nude mice (8 weeks old) bearing subcutaneous LoVo colon carcinoma xenografts (200–250 $mm^3$) were randomly assigned into the designated treatment groups. In Protocol 1, mice received vehicle or ZD1839 (100 mg $kg^{-1}$) for 14 days, either alone or combined with a single 5 Gy dose of X-irradiation. In Protocol 2, mice received vehicle or ZD1839 (100 mg $kg^{-1}$) for 14 days, either alone or combined with $3 \times 2$ Gy fractions at 24-h intervals. ZD1839 or vehicle was administered 2 h before radiotherapy. Experiments were terminated when a relative tumour volume four times that at the initiation of treatment was obtained ($7.4 \pm 0.4$ and $9.8 \pm 0.6$ days for vehicle controls in Protocols 1 and 2, respectively). Protocol 1 revealed comparable growth delays (GD) for vehicle plus 5 Gy or ZD1839 alone treated tumours ($7.6 \pm 0.6$ and $7.8 \pm 0.6$ days, respectively). ZD1839 plus 5 Gy produced tumour regression and a GD of 14.1 (±1) days, a radiation dose modification of 1.6. In Protocol 2, ZD1839 alone induced a GD of 5.1 (± 0.9) days and $3 \times 2$ Gy plus vehicle a GD of 7.4 (±1.2) days. Combining ZD1839 with $3 \times 2$ Gy enhanced the therapeutic effect of radiation alone, and a GD of 18.7 (± 2.8) was achieved. The GD obtained with the combined therapy exceeded that seen with $5 \times 2$ Gy ($11.6 \pm 1.3$ days). These data support a clinical evaluation of ZD1839 and radiotherapy.

'Iressa' is a trade mark of the AstraZeneca group of companies.

### P25 ASSESSMENT OF PRECLINICAL MODELS FOR COLORECTAL CANCER

RW Wilkinson[1], D Ellison[2], R Poulsom[3], J Staub[4], M Ilyas[4], WF Bodmer[4], D Snary[1], SJ Mather[2], EL Ross[1], [1]Imperial Cancer Research Technology, Dominion House, 59 Bartholomew Close, London EC1A 7BE, UK, [2]ICRF Nuclear Medicine Group, Dominion House, 59 Bartholomew Close, London EC1A 7BE,[3] ICRF Histopathology Dept., 44 Lincoln Inns Field, London,[4] ICRF Cancer and Immunogenetics Lab, Institute of Molecular Medicine, University of Oxford, UK

In this study a human carcinoembryonic antigen transgenic (CEA.Tg) mouse model was validated for the pre-clinical assessment of anti-CEA-directed agents. CEA.Tg mice were derived from a colony which express the complete human CEA gene and flanking regulatory sequences which results in cell-type and organ specific expression of CEA. As with humans, the mice had low serum levels of CEA and cell surface CEA expression was localised in the gastrointestinal (GI) tract. Pharmacokinetic studies in wildtype and CEA.Tg mice were performed after intravenous injection of $I^{125}$-labelled murine monoclonal antibodies (mAbs) [either an anti-human CEA mAb, PR1A3, or an isotype control (IC)] and tissues were sampled at 4, 24 and 48 hours after the injection. Both mAbs showed similar biodistribution patterns in the wildtype mice while in the CEA.Tg, PR1A3 specifically localised to the CEA expressing tissues. In the GI tract the percentage injected dose (%ID) for PR1A3 was significantly higher than the IC mAb at all the time points sampled. In CEA.Tg mice bearing a murine tumour transfected with human CEA, PR1A3 targeted and was retained at the tumour site at high levels. When CEA.Tg mice were backcrossed with the natural mutant Min (multiple intestinal neoplasia) mouse offspring developed spontaneous CEA positive intestinal polyps that were bound by $I^{125}$ labelled PR1A3. These results demonstrate the therapeutic potential of the anti CEA antibody, PR1A3, and emphases the validity of using relevant models in preclinical studies.

### P26 DEVELOPMENT OF A MURINE CEA TUMOUR MODEL

JJ Irlam, DE Gilham, RE Hawkins, CRC Department of Medical Oncology, University of Manchester, Paterson Institute for Cancer Research, Manchester, M20 4BX, UK

The primary aim of this study was to develop a murine tumour model expressing the human tumour associated antigen, Carcinoembryonic antigen (CEA). CEA expression has been linked with colorectal / breast and ovarian tumours. The development of this model will then be used to test targeted adoptive immunotherapy technology and primarily the targeting of modified T-lymphocytes to CEA expressing tumours.

The murine Lewis Lung (LL-2) carcinoma cell line is of C57/bl-6 background, are negative by flow cytometry and Western blot analysis for CEA. A 900 base pair domain of the 5′ end of human CEA cDNA was engineered to be expressed on the cell surface by a coupling with a murine MHC class I transmembrane anchor domain (CEAdo.MTM). This domain is specifically targeted by the single chain antibody C23, derived from the monoclonal antibody MFE23. The CEA fragment was cloned into a retroviral vector, and the LL-2 cells were transduced. The transduced cells were then quickly cloned by limiting dilution. The expanded clones were analysed for the expression of CEAdo.mtm on their surface, using the single chain antibody C23hufc (kindly provided by Chris Allinson, Department of Med. Onc.). A clone expressing high level was identified; Clone 28. This initial clone was further sub-cloned through 3 rounds of limiting dilution, in an attempt to collect a pure population of high CEAdo.mtm expressing cells. These sub-clones were chosen due to their varying levels of CEAdo.mtm surface expression, ranging from low to high. These clones were used in *in vitro* assays with modified T-lymphocytes. Effector T-cells were modified to specifically target the CEA domain using the C23 single chain antibody coupled to CD3. After co-culture with targetted lymphocytes the remaining target cell population was found to be CEA negative by flow cytometry and western blot analysis. This infers that the CEA positive target cells had been successfully targeted by the modified lymphocytes. The target population exposed to non-specific modified T-cells showed no change in the cell viability and no loss in CEA domain expression when analysed by Flow cytometry and western blot analysis.

The highest expressor of the CEAdo.mtm, clone 28–7–12 was used to establish an In vivo mouse model, to investigate the maintainence of expression and the growth kinetics of the LL-2 cells transduced with CEAdo.mtm. Preliminary growth curves indicated that there was no difference in the growth kinetics between the transduced cell line and the parent LL-2 cell line. Immunostaining using cryostat sections clearly demonstrated a maintainance of CEAdo.mtm expression throughout the 3 week growth period of the tumour.

It is anticipated that these cells will provide a basis of a stringent model to further investigate the effectiveness of gene modified cellular adoptive immunotherapy.

### P27 A RAPID METHOD FOR THE ANALYSIS OF CELL PROLIFERATION AND SURVIVAL IN INTESTINAL ADENOMA FROM THE APC$^{MIN}$ MOUSE

JL Burns, JA Howell, and AB Hassan*, Cell and Development Group, Department of Zoology, University of Oxford, South Parks Road, Oxford OX1 3PS, UK basshassan@zoo.ox.ac.uk

Mutation of the murine gene coding for Apc has provided a model of human familial adenomatous polyposis coli ($Apc^{Min1+}$). The number and size of small intestinal polyps depends on age, strain dependent genetic modifiers, dietary fat and gut organisms. Combination of $Apc^{Min1+}$ with disruption of genes implicated in colorectal carcinoma (e.g. *Msh2*, *Egf*, *Igf2*, *p53*, *Dnmt*) has extended the utility of this model to the analysis of the genetic pathways relevant to human cancer. Insights into the mechanistic effects of genetic modification have been limited, partly because of the difficulty in developing reliable and reproducible assays using intestinal tissue.

We describe a rapid method which may help in the analysis of cell survival and proliferation in intestinal adenoma. Small and large intestines from C57B1/6J wild type and $Apc^{Min1+}$ adult mice were removed into ice-cold PBS. Rapid processing of intestines, with removal of intestinal contents and exposure of the lumen, was performed using a jig and blade designed (by us) for this purpose. Segments of tissue or intestinal adenoma were incubated in ice-cold PBS ($Ca^{2+}$ and $Mg^{2+}$ free)/EDTA and gently filtered (70 µm filter). Single cell suspensions of epithelial cells free from smooth muscle were generated. Cell processed in this way are amenable to either culture, antibody labelling (for analysis of chimeras) or flow cytometric analysis. To investigate cell proliferation, we utilised antibodies to Mcm2 and BrdU incorporation. We present new information following pulse and pulse-chase BrdU incorporation of normal and adenomatous epithelium which reveal aspects of cell turnover within adenoma. We have analysed normal intestinal tissue and adenoma cell death using sub-G1 analysis of flow cytometric profiles and FAM-VAD-FMK to detect caspase activity (1–9). Finally, we present the results of analysis following combination of $Apc^{Min1+}$ with genetic modification of *Igf2* and *Msh2*. Experiments were performed following approval of the Home office and Departmental ethics committee and following the guidelines of the UKCCR.

*Supported by Senior Clinical Research Fellowship of the Cancer Research Campaign.

### P28 CYCLOOXYGENASE-2 (COX-2) EXPRESSION IN *Apc*$^{Min/+}$ MOUSE INTESTINAL MACROPHAGES

OO Faluyi, J Henwood, C Bonifer, MA Hull, PL Coletta, Molecular Medicine Unit, University of Leeds, St James's University Hospital, Leeds, LS9 7TF, UK

There is compelling genetic and pharmacological evidence to suggest that the inducible isoform of the cyclooxygenase gene, Cyclooxygenase-2 (Cox-2) plays an important role in intestinal tumorigenesis. Elevated levels of Cox-2 expression have been demonstrated in colorectal cancers and adenomas in humans as well as in intestinal tumours in animal models. Previously, we have shown by immunohistochemistry that Cox-2 expression is up-regulated and localised to interstitial macrophages in *Apc*$^{Min/+}$ mouse intestine as well as in human sporadic colorectal adenomas. To further define the Cox-2 expressing cell population, intestinal lamina propria mononuclear cells (LPMNCs) have been isolated from *Apc*$^{Min/+}$ mice and wild-type littermates. Isolated LPMNCs were subjected to semi-quantitative reverse transcription-polymerase chain reaction (RT-PCR) analysis. Higher levels of Cox-2 expression were detected in LPMNCs isolated from *Apc*$^{Min/+}$ mouse intestine as compared with wild-type littermates.

In order to isolate the Cox-2 expressing macrophage population, the *Apc*$^{Min/+}$ mouse has been crossed with the *EGFP-lysozyme* mouse in which green fluorescent protein (GFP) is expressed specifically in cells of the myelomonocytic lineage. Using these mice and the macrophage specific monoclonal antibodies ER-HR3, F4/80 and MOMA-1, interstitial macrophages have been separated from other LPMNCs by flow cytometry and fluorescence-activated cell sorting techniques.

Utilizing semi-quantitative RT-PCR, we have shown that there is increased Cox-2 expression in *Apc*$^{Min/+}$ mouse LPMNCs. Further studies on *Apc*$^{Min/+}$ mice expressing GFP in their macrophages will allow prostaglandin and cytokine secretion profiles of interstitial macrophages in *Apc*$^{Min/+}$ mice to be determined which will help in clarifying the role of Cox-2 in intestinal tumorigenesis as well as in revealing potential therapeutic targets for colon cancer prevention.

### P29 ELEVATED PYRIMIDOPURINONE-DEOXYGUANOSINE ADDUCTS IN ADENOMAS OF *APC*$^{MIN}$ MICE AND SUPPRESSION BY DIETARY CURCUMIN

Ricky A Sharma, Sarah Perkins, Raj Singh, Will P Steward, Andy Gescher, Oncology Department & MRC Toxicology Unit, University of Leicester, LE1 9HN, UK

Pyrimidopurinone adducts of deoxyguanosine ($M_1G$) reflect oxidative damage of DNA *via* base propenals, and production of malondialdehyde (MDA) by endogenous lipid peroxidation and by catalysis via cyclooxygenase (COX). COX-2 is upregulated at early stages of adenoma formation in Min mice, a model of intestinal carcinogenesis characterized by an *Apc* gene defect[1]. Curcumin, a polyphenolic antioxidant derived from the spice turmeric, has been shown to inhibit lipid peroxidation and COX-2 expression in vitro. Dietary curcumin diminishes rat colon mucosal $M_1G$ levels[2], and suppresses adenoma formation in Min mice[3]. We tested two hypotheses: MDA and $M^1G$ levels are elevated in intestinal adenomas of *Apc* mutant Min mice; and $M_1G$ levels are modified by dietary curcumin in a manner favourable to cancer chemoprevention. Mice were fed a standard AIN-76A diet from 4 to 18 weeks of age, containing 0.1% or 0.2% curcumin. MDA concentration was measured by colorimetric assay, and $M_1G$ adduct levels by immunoslot blot. Curcumin and its metabolites were analyzed by HPLC. In control animals, although MDA levels did not differ significantly between intestinal mucosa and adenoma tissue (0.9–2.1 nmol/mg protein), $M_1G$ levels were 3-fold higher in adenomas than mucosa (12.0 ± 3.8 *versus* 3.7 ± 1.3 adducts per $10^7$ nucleotides, $P < 0.001$ by ANOVA). The 0.1% and 0.2% curcumin diets did not alter $M_1G$ levels in normal mucosa, but both diets decreased adenoma $M_1G$ by approximately 43% when compared to control animals ($P = 0.006$ by ANOVA). In mice fed the 0.2% diet, the concentrations of curcumin in intestinal and colonic mucosa were 507 ± 86 and 111 ± 23 nmol/g tissue respectively. The results demonstrate for the first time that levels of $M_1G$ adducts are elevated in premalignant lesions in this genetic mouse model, and that the discrepancy with normal mucosal $M_1G$ is not accounted for by MDA concentration. The dietary antioxidant curcumin can attenuate this elevation, and merits evaluation in patients with colorectal polyposis.

1. Oshima M et al 1996 *Cell* **87**: 803–809
2. Sharma RA et al 2001 *Clin Cancer Res*: *in press*
3. Mahmoud NN et al 2000 *Carcinogenesis* **21**: 921–927

### P30 PILOT STUDY OF *CURCUMA* EXTRACT IN PATIENTS: SAFETY AND BIOLOGICAL ACTIVITY

RA Sharma, HR McLelland, CR Ireson, DJL Jones, KA Hill, SA Euden, ML Williams, M Pirmohamed[2], SM Plummer, MM Manson, AJ Gescher, WP Steward, Oncology Department & MRC Toxicology Unit, University of Leicester, LE1 9HN, UK[2], Department of Pharmacology, University of Liverpool, L69. 3GE, UK

*Curcuma* spp. extracts, particularly curcumin, prevent colon cancer in rodents, and may be therapeutic against established malignancy. Information on the pharmacodynamics and pharmacokinetics of curcumin in humans is lacking. A dose-escalation study of standardized *Curcuma* extract in capsule form (P54FP, Phytopharm plc.) was conducted at doses between 440 and 2200 mg per day, containing 36 to 180 mg curcumin and 400 to 2000 mg essential oils. Fifteen patients with advanced progressive colorectal cancer, refractory to standard chemotherapy, received *Curcuma* extract daily for up to 4 months. In addition to chemotherapeutic parameters, 3 potential markers of systemic biological activity were measured in blood: Lymphocytic glutathione S-transferase (GST) activity; leukocytic levels of a DNA adduct ($M_1G$) formed by malondialdehyde, a product of lipid peroxidation and prostaglandin (PG) biosynthesis; and $PGE_2$ concentrations, both basal and those induced in vitro by lipopolysaccharide. Patients were also genotyped for *GSTM1*, *GSTP1* and *GSTT1* polymorphisms. Pretreatment levels of leukocytic $M_1G$ in patients with *GSTM1* null genotype were 74% higher than those in *GSTM1* expressers ($P < 0.001$ by ANOVA). Oral *Curcuma* extract was well tolerated, and dose-limiting toxicity was not observed. Neither curcumin nor its metabolites were detected in blood or urine (limit of detection 5 pmol/ml). Curcumin was recovered from the faeces of all patients and curcumin sulphate was identified in one, confirmed by mass spectrometry. Ingestion of 440 mg *Curcuma* extract for 28 days was accompanied by a 59% decrease in lymphocytic GST activity ($P < 0.001$ by ANOVA). Lymphocytic GST activity in patients at higher doses and leukocytic $M_1G$ levels in all patients were constant within each patient and unaffected by treatment with *Curcuma* extract. Basal and induced $PGE_2$ values hinted at dose-dependent inhibition. Five patients exhibited stable disease on CT scan for 2–4 months of treatment. The results suggest that i) *Curcuma* extract can be administered to patients at doses of up to 2.2 g daily without significant toxicity, ii) curcumin has low oral bioavailability in humans, and iii) whole blood $PGE_2$ concentration and lymphocytic GST activity may be useful as systemic pharmacodynamic biomarkers in trials incorporating larger patient numbers.

Supported by the Medical Research Council, Phytopharm plc., and the University Hospitals of Leicester.

### P31 THE METABOLISM OF THE CHEMOPREVENTIVE AGENT CURCUMIN BY HUMAN GUT

CR Ireson[1], S Orr[3], D Jones[1], ML Williams[2], CK Lim[1], RD Verschoyle[1], WP Steward[2], AJ Gescher[1], [1]MRC Toxicology Unit, [2]Department of Oncology, University of Leicester LE1 9HN, and [3]UK Human Tissue Bank, De Montfort University, Leicester, UK

Curcumin (diferuloylmethane) is the major active ingredient in the spice turmeric, a yellow pigment that can be isolated from the rhizome of the *Curcuma longa* plant. Whilst there is substantial evidence for the chemopreventive activity of curcumin in animal models and in cell lines, little is understood regarding the role of metabolism in its efficacy. Recently we characterised curcumin metabolites in rats in vivo and in suspensions of human and rat hepatocytes (1). These studies and evidence presented earlier suggest the possibility that curcumin is conjugated in the gut. Therefore we compared metabolism of curcumin to its sulphate and glucuronide conjugates and products of its reduction by subcellular fractions prepared from human small intestine and liver. Curcumin (0.1 mM) was incubated with cytosol or microsomes for 1 hour. Putative metabolites were identified by liquid chromatography-electrospray mass spectrometry in the selected ion-monitoring mode. Curcumin sulphate (m/z = 447) was identified in cytosol of gut (103 ± 23 nmol/mg protein) and liver (39 ± 8 nmol/mg protein). Microsomes metabolised curcumin to its glucuronide conjugate (m/z = 543) at 255 ± 229 nmol/mg of protein in gut and 94 ± 30 nmol/mg in liver. The reduction products of curcumin, tetrahydrocurcumin (m/z = 371) and hexahydrocurcumin (m/z = 373) were found in the cytosol of both tissues. These results demonstrate that reduction of curcumin occurs in both liver and gut cytosol and are consistent with the notion that glucuronidation and sulphation are important routes of curcumin metabolism in human gut. As these metabolites are less capable of interfering with cyclooxygenase-2 expression than parent curcumin[1], gut seems to play a deactivating role in curcumin disposition.

1. Ireson et al, *Cancer Research*, in press

### P32 GLYCINE-EXTENDED GASTRIN PROMOTES COLON CARCINOGENESIS

A Aly, GS Baldwin, A Shulkes, University of Melbourne, Department of Surgery, Austin Campus, ARMC, Studley Rd., Heidelberg, Victoria 3084, Australia

Recent interest has focussed on the role of gastrin precursors as potential promoters of carcinogenesis in colorectal carcinoma.[1] A useful model for the identification of potential promoters of carcinogenesis is the formation of aberrant crypt foci (ACF) in the colons of rats exposed to colon-specific carcinogens such as azoxymethane.[2] The risk of malignancy is related to the total number and nature of ACF formed. The aim of this project was to investigate the short term effect of continuous systemic infusion of the gastrin precursor glycine-extended gastrin (G-gly) on the development of ACF in rat colon.

**Methods** G-gly was delivered systemically via Alzet miniosmotic pumps at a dose of 2.5 nmol/kg/hr over a four week experimental period. Rats were injected with azoxymethane (15 mg/kg) subcutaneously in two doses one week apart at the beginning of the experiment. Colons were harvested, fixed in formalin and stained with 0.1% methylene blue to allow visualisation of ACF. The number and nature of ACF were counted and compared to controls not exposed to G-gly.

**Results** Azoxymethane reliably produced aberrant crypts in both control and G-gly-treated rats. The number of ACF per centimeter of colon in the G-gly-treated group was increased by 56% compared to controls ($P = 0.006$). There was an increase in all ACF subtypes (with one, two, three or more crypts per focus) in the G-gly-treated rats, and the relative proportions of each subtype were similar to azoxymethane-treated controls. There was no significant change in the distribution of ACF throughout the colon after G-gly treatment.

**Conclusion** The observation that G-gly increased the overall number of all types of ACF induced in azoxymethane-treated rats suggests that G-gly may act as a promoter of carcinogenesis.

1. Baldwin GS, Shulkes A (1998) Gastrin, gastrin receptors and colorectal carcinoma. *Gut* **42**: 581–584
2. McLellan EA, Bird RP (1988) Aberrant crypts: Potential preneoplastic lesions in the murine colon. *Cancer Res* **48**: 6187–6192

### P33 LOSS OF EXPRESSION OF ESTROGEN RECEPTOR BETA IN COLON CANCER

N Jassam[1], SM Bell[1], S Gray[1], L Harkin[1], G Skliris[2], V Speirs[2], P Quirke[1], [1]Academic Unit of Pathology, Algernon Firth Building and [2]Molecular Medicine Unit, Clinical Sciences Building, University of Leeds, Leeds, West Yorkshire, LS2 9JT, UK

Gender differences in the incidence and behaviour of colon cancer suggest a hormonal influence. The age-adjusted incidence rates for colon cancer are lower in women than men and furthermore epidemiological data suggest a protective effect for hormone replacement therapy (HRT) on this disease. However the biological mechanism by which estrogen influences the pathogenesis of colorectal cancer is unknown. Evidence exist for the expression of estrogen receptors (ER) in human colon tissue, recently it has been shown that the predominant ER is ERβ. The aim of the present study is to investigate ERβ expression in normal and colorectal cancer samples, at both the protein and mRNA level in a large well-defined patient cohort.

We have performed immunohistochemistry using a monoclonal antibody directed against the C-terminus of ERβ on 91 archival formalin-fixed paraffin embedded samples from consecutive patients diagnosed with colorectal cancer at Leeds General Infirmary during 1998 (53 males, 38 females, age range = 48–92). Strong nuclear immunoreactivity was observed in epithelial cells lining the colonic crypts and in tumour cell nuclei. Occasional positivity was observed in fibroblasts. Loss of ERβ expression was found in 21% of the colon cancer samples irrespective of the gender, age, or tumour stage of the patients. High levels of ERβ expression were identified in the adjacent normal colon mucosa. Interestingly loss of ERβ expression was higher in left colon and rectal cancers (27%) than right colon cancers (8%). Currently the immunohistochemistry results are being confirmed by Western analysis of 20 paired normal and colonic tumours.

Our results support epidemiological studies, which suggest that hormone replacement therapy and or dietary estrogen influence the course of colon cancer and that these effects are mediated by ERβ.

### P34 THE PATTERN OF EXPRESSION OF CDC25B INVERSELY COMPLEMENTS THAT OF CYCLIN B1 IN NORMAL COLUMNAR INTESTINAL EPITHELIUM AND NEOPLASTIC OESOPHAGEAL TISSUE

CM Pring[1], AGK Li[1], C Verbecke[2], PJ Guillou[1], GWB Clark[1], PA Robinson[2], Department Surgery[1]/ Molecular Medicine[2], Clinical Sciences Building, St. James's University Hospital, Leeds, UK

**Aims** Increased levels of transcripts encoding the $G_2$/M cell cycle checkpoint phosphatase, CDC25B, have been reported in oesophageal adenocarcinoma as well as many other carcinomas including breast, gastric and head and neck cancers, compared to matched normal tissues. Our aim was to compare the protein expression of CDC25B with the $G_2$/M cyclin, Cyclin B1, and the cell proliferation marker Ki-67, in both normal columnar mucosa from the small intestine and neoplastic oesophageal tissue.

**Procedures** CDC25B, Ki-67 and Cyclin B1 were detected by standard immunohistochemical techniques and their expression was compared in parallel tissue sections. Immunoreactivity was scored by two independent assessors and graded; –, +, ++ or +++ (– = no expression; +++ = strong expression). Six sections of normal columnar epithelium and twenty one sections of oesophageal adenocarcinoma were compared.

**Major findings** Cells in the basal layer of normal columnar intestinal mucosa and in crypts stained (+++) for Ki-67 and (++) for Cyclin B1 but were negative for CDC25B in all six sections. In marked contrast, the more differentiated cells of the villi were negative (–) for Ki-67 and Cyclin B1 but stained (++/+++) for CDC25B. Furthermore, whereas strong expression of CDC25B was observed in 6/7 well differentiated tumours (positive predictive value 75%) and in 2/8 moderately differentiated tumours, only weak staining (–/+) was noted in the majority (5/6) of poorly differentiated tumours. This pattern correlates inversely with the expression of Cyclin B1.

**Significance of research** As increased levels of *CDC25B* transcripts have been reported previously in oesophageal adenocarcinomas, our results infer that the apparent absence of CDC25B expression is likely to be due to its rapid turnover.

**Conclusion** We have determined an inverse relationship between the levels of expression of CDC25B and Cyclin B1 in cells of normal intestinal mucosa and oesophageal adenocarcinomas. This finding was surprising, as the established function of active CDC25B in cells is to promote $G_2$/M transition. We propose that its apparent absence in proliferating cells is due to its rapid turnover and its stabilisation in more differentiated cells. Its role in the more differentiated phenotype is not clear at the present time.

### P35 c-erbB-2 PROTEIN EXPRESSION AND GENE POLYMORPHISM IN COLORECTAL CANCER

JA McKay[1], JF Loane[2], VG Ross[2], MM Ameyaw[1], GI Murray[2], J Cassidy[1], HL McLeod[1]. Departments of [1]Medicine and Therapeutics, and [2]Pathology, University of Aberdeen, Aberdeen, Scotland, AB25 2ZD, UK

The c-erbB-2 oncogene is frequently activated in epithelial tumours, and amplification and/or overexpression is clinically associated with poor prognosis, and a reduced response to chemotherapy and endocrine therapy, in breast cancer. In addition, a $Val^{655}$ Ile polymorphism in the transmembrane domain of the c-erbB-2 gene has been associated with an increased risk of breast cancer in a Chinese population[1]. The prognostic utility of c-erbB-2 in colorectal cancer remains equivocal, while the relevance of the $Val^{655}$Ile polymorphism is unknown in this disease. We have investigated the protein expression of c-erbB-2 in a large cohort of 248 well-characterised colorectal tumours, and in a subset of 42 lymph node metastases. c-erbB-2 protein was assessed using immunohistochemistry, and staining was evaluated according to the US FDA-approved scoring system[2], which classifies complete membrane staining of >10% of tumour cells as positive c-erbB-2 expression. Furthermore, we have evaluated the $Val^{655}$Ile polymorphism by PCR-RFLP in 151 colorectal cancer patients and 257 healthy control subjects. Immunohistochemical studies revealed that 203/248 (81.8%) tumours expressed c-erbB-2, although only 8/248 (3.2%) showed strong positive immunoreactivity. Colon tumours were significantly more likely to express c-erbB-2 protein than rectal tumours ($P = 0.005$), however there was no correlation with Dukes' stage, differentiation grade, age or sex of the patient. Moreover, no association was seen with patient survival ($P = 0.56$), which suggests that c-erbB-2 is not a prognostic factor in colorectal cancer. Only 22/42 (52.4%) lymph node metastases displayed staining patterns concordant with their corresponding primary tumour, with the majority of divergent cases showing higher staining of primary tumours (18/42 cases, 42.9%). This indicates that c-erbB-2 immuno-histochemistry of primary colorectal tumours cannot be used to predict the status of lymph node metastases. PCR-RFLP analysis of the c-erbB-2 $Val^{655}$Ile polymorphism in colorectal cancer patients and a control group of Caucasian subjects demonstrated that allele frequencies were identical between cases and controls (Ile = 0.80 and Val = 0.20 in each case), indicating that this polymorphism is not related to the risk of developing colorectal cancer in this population. In addition, there was no relationship between c-erbB-2 protein expression and the $Val^{655}$Ile polymorphism ($P = 0.58$). In conclusion, it would appear that c-erbB-2 is not a major factor involved in the development or progression of colorectal cancer, and that novel therapies targeting this molecule may be of little benefit in this disease.

1. Xie D, Shu X-O, Deng Z et al 2000 *J Natl Cancer Inst* **92**: 412
2. Jacobs TW, Gown AM, Yaziji H 1999 *J Clin Oncol* **17**: 1983

### P36 THE PROGNOSTIC VALUE OF HER-2 IN DUKES' B COLORECTAL CANCER

S Essapen[1]*, M Green[2], C de Vries[1], C Shotton[1], C Topham[2], C Marks[2], M Cook[2], H Modjtahedi[1], H Thomas[1]
[1]Cancer Study Group, University of Surrey, Guildford, Surrey GU2 7XH, UK, [2]Royal Surrey County Hospital, Guildford, Surrey GU2 5XX, UK

**Introduction** Human Epidermal Growth Factor Receptor-2 (HER-2) is a transmembrane glycoprotein, which is overexpressed in a number of epithelial cancers. The prognostic role of HER-2 has been established in breast cancer but remains controversial in colorectal cancer.

**Aim and methods** In the present study, the prognostic significance of HER-2/*neu* (c-*erb*B-2), was investigated in fifty patients with Dukes' B colorectal cancer who underwent radical surgery between 1990 and 1995, using an anti-HER-2 mAb HM64.13 (developed by Dr Modjtahedi) and a standard avidin-biotin horseradish peroxidase immunohistochemistry method.

Four categories were defined for the percentage of tumour cells showing cytoplasmic and/or membranous immunostaining (CMI) (≤10%, > 10–30%, >30–50%, >50%). The percentage of tumour cells exhibiting membranous immunostaining (MI) was similarly categorised. The intensity of the immunostaining was categorised as negative, weak, moderate, and strong. The association between these scores and cancer death or time to progression was estimated using Cox regression. Covariates included in the model were age, sex, tumour site, size, stage, grade, and lymphovascular invasion. Correlation between variables was tested using $\chi^2$ tests. The mean age of the study population was 72.5. Of the 50 patients, 19 had a tumour size of >5 cm; 8 had a T4N0 cancer; and 4 patients had lymphovascular invasion.

**Major findings** Six (12%) patients had negative (≤10%) HER-2 immunostaining. Of those tumours showing HER-2 overexpression, 29 (58%) cases had >50% CMI, and 7 (14%) cases showed MI in >50% of the tumour. The intensity of the MI and CMI was moderate to strong in 28 (56%) and 26 (52%) cases respectively. A significant correlation was found between T4N0 cancers and the percentage of tumour cells showing MI ($P = 0.05$). Furthermore, there was a suggestion of a correlation between the percentage of tumour cells with CMI and lymphovascular invasion ($P = 0.15$). No correlation with clinical outcome was seen which might be explained by the lack of events in this small cohort of patients.

**Conclusion** HER-2 overexpression is seen in a significant number of colorectal cancers. This data suggests that there may be a correlation between HER-2 overexpression and T4N0 cancers and lymphovascular invasion. This needs to be explored in a larger cohort of patients, as it may aid the selection of Dukes' B patients at higher risk of recurrence, and for whom adjuvant chemotherapy is indicated.

### P37 UP-REGULATION OF HEPARIN-BINDING EPIDERMAL GROWTH FACTOR-LIKE GROWTH FACTOR BY GASTRIN IN COLORECTAL CANCER CELLS

DF McWilliams, PA Clarke, SA Watson Academic Unit of Cancer Studies, University Hospital, Nottingham, NG7 2UH, UK

**Introduction** Gastrin is a central, autocrine growth factor in colorectal tumours. Gastrin also modulates expression of the epidermal growth factor family in the stomach. At least three gastrin peptide species are secreted by colorectal tumours and may act to promote growth via different receptor isoforms. In this study we used antisense gastrin mRNA technology to investigate the effects of this hormone upon colorectal tumour proliferation and downstream gene activation.

**Materials and methods** Total RNA was extracted from 20 paired colorectal tumour and resection margin tissue removed at Nottingham University Hospital. Reverse transcription real-time PCR was performed for gastrin, epidermal growth factor (EGF), amphiregulin (AR), heparin binding EGF-like growth factor (HB-EGF) and transforming growth factor-α (TGF-α). Quantification of relative gene expression levels (the ΔΔct value) was obtained by comparison to the housekeeping gene, GAPDH. Antisense gastrin transfections were performed on HCT116, Pan l, MGLVA1 acites and ST16 cell lines, which were selected for stability with G418. RNA extraction and RT-real time PCR for the above cDNA's were then performed. A tetrazolium based proliferation assay (MTT assay) was performed to determine growth rates of antisense cell lines. Immunohistochemistry staining for HB-EGF, AR and CD31 were performed on 6 tumour and resection margin paraffin-embedded tissue sections. Image analysis software was used to quantify staining intensity from 8 observations per section (HB-EGF and AR).

**Results** Gastrin was detected in 80% (16/20) of colorectal tumour cDNA's. Members of the EGF family were almost ubiquitously detected in all cDNA's At the mRNA level, a highly significant correlation between gastrin and HB-EGF was observed ($P < 0.01$, Durban-Watson). All other EGF family members showed non-significant positive correlations to gastrin mRNA levels. Antisense gastrin significantly reduced proliferation and HB-EGF mRNA levels in the four tumour cell lines examined. In the colorectal tumour cell line (HCT116), gastrin antisense reduced the MTT uptake ($P < 0.05$, Student's t-test) and median ΔΔct value of HB-EGF, from 0.00298 to 0.00024 ($P < 0.01$, Mann-Whitney). The HB-EGF staining intensity was significantly higher in 5/6 colorectal tumours over paired resection margins ($P < 0.05$, T-test). Blood vessels from consecutive sections of tumour and resection margin stained positively for CD31 and HB-EGF.

**Conclusions** Autocrine gastrin up-regulates proliferation rates and HB-EGF mRNA in tumour cell lines. The HB-EGF protein is over expressed in colorectal tumour tissue compared to resection margin mucosa. Gastrin may increase proliferation directly in colorectal tumours and also modulate HB-EGF levels. The HB-EGF levels in colorectal cancer may also influence the proliferation of epithelial cells and, possibly, tumour vasculature.

### P38 EXPRESSION OF EGFR and HER-2/neu IN GASTRIC CARCINOMA AND THEIR PROGNOSTIC SIGNIFICANCE

H Modjtahedi[1], B Gharesi-Fard[2], M Vasei[2], A Malekhosseini[2], A Ghaderi*[2]
[1]Cancer Study Group, University of Surrey, Guildford GU2 7XH, UK, [2]Dept Immunology, Pathology and Surgery, Shiraz University of Med. Sciences, Shiraz, Iran

Overexpression of the members of the type-I growth factor receptor family, in particular EGFR and HER-2/*neu*, has been reported in a number of epithelial malignancies and this in turn has been associated with a poor prognosis in many such patients.

In this study 124 specimens of known cases of gastric carcinoma in Iranian patients were examined immunocytochemically for the expression of the EGFR and HER-2 molecules and results were compared with clinicopathological data. Indirect immunoperoxidase method using specific mAbs was applied on paraffin-embedded tissues.

Results indicated that 47 (37.9%) and 19 (15.3%) of tumors showed positive (either membrane or cytoplasmic) staining with anti EGFR mAb ICR16 and anti-HER-2 mAb ICR12 respectively. Expression of the EGFR was significantly associated with lymph node involvement ($P < 0.02$), locally invasive ($P < 0.002$), large tumor size ($P < 0.02$), and high-stage (III/IV) tumors ($P < 0.0004$), but no correlation was observed between nuclear grade and the location of the tumor. Expression of HER-2 was significantly correlated to low stage tumors (I/II). Although, HER-2 positive tumors were more likely to be associated with serosal invasion and lymph node metastasis, these correlations were not significant.

The results of this study support the hypothesis that overexpression of the EGFR may be a predictor for poor prognosis in patients with gastric carcinoma.

### P39 CHANGES IN THE EXPRESSION OF COMPONENTS OF THE IGF SYSTEM IN ADENOCARCINOMA OF THE COLON AND RECTUM

M Davies[1], S Coe[1], S Gupta[1], D Adeyemo[1], G Goldspink[2] , M Winslet[1]
Department of Surgery[1], Department of Anatomy[2], Royal Free Hospital & University College Medical School, Pond Street, London. NW3 2QG, UK

**Aims** The mitogenic and anti-apoptotic signals of the IGF-I and -II are mediated via the insulin-like growth factor type-I receptor (IGF-IR). Insulin-like binding protein-4 (IGFBP-4) has been shown to be solely inhibitory to the actions of IGF-I and -II. Our aim was to compare the expression and distribution of IGF-IR and the inhibitory IGFBP-4 in malignant and non-malignant human colorectal tissue.

**Materials** Samples were removed from resected colorectal tissue (30 patients) and were frozen immediately. RNA was extracted and fragments of IGF-IR and IGFBP-4 were cloned using RT-PCR. Protein was extracted and separated using SDS PAGE and probed using a polyclonal antibody to IGFBP-4. Frozen sections were cut and immuno-histochemistry was used to determine the distribution of IGF-IR and IGFBP-4 in the tissue, using an avidin-biotin immuno-staining complex. This was carried out in five fields in both superficial and deep portions of samples. The staining was repeated with immuno-fluorescence and quantified using an image analyser.

**Results** We detected mRNA for both IGFBP-4 and IGF-IR in all samples. Using western immuno-blotting a band corresponding to 28 kDa, (representing IGFBP-4) was detected in all samples. Densitometric analysis of the radiographs confirmed that the expression of IGFBP-4 was significantly less ($P < 0.05$) in the malignant tissue. Immuno-staining for IGF-IR was greatest in the crypts, lamina propria and the basement membrane. This expression was quantified using immuno-florescence and shown to be similar in both groups. There was no correlation between strong IGF-IR positivity and either grade or staging of tumours. IGFBP-4 stained most strongly in the muscle layers and stronger in the non-malignant tissue.

**Conclusion** Though the IGF-IR receptor is still abundantly expressed we did not find increased expression in malignant tissue – contrary to recent publications[1]. Therefore, the potential exists for IGF-I and -II, both of which are expressed in colorectal cancer[2], to exert anti-apoptotic and mitogenic effects via the IGF-IR. The expression of inhibitory growth factor IGFBP-4 was less in malignant colorectal tissue and so there may be increased bio-availability of IGFs in colorectal cancer. The Roche light-cycler is currently being used to quantify the IGFBP-4 mRNA to establish whether the reduced expression occurs at the gene level.

1. Hakam A, Yeatman T, Lu L, Mara L, Marcet G, Nicosa S, Karl N and Coppola D (1999) Expression of insulin-like growth factor type-I receptor in human colorectal cancer. *Human Pathology* **30** (10): 1128–1133
2. Freier S, Weiss O, Eran M, Flyvberg R, Dahan R, Nepesh I, Safra T, Shilani E and Raz I (1999) Expression of insulin growth factor in adenocarcinoma of the colon *Gut* **44**: 704–708

### P40 PRIMARY TUMOUR EXCISION REVERSES THE REDUCTION OF CIRCULATING DENDRITIC CELLS IN COLORECTAL CANCER

A Huang[1], J Gilmour[2], P Amjadi[2], DC Henderson[2], TG Allen-Mersh[1], Depts. of Surgery[1] & Immunology[2], ICSM, Chelsea & Westminster Hospital, London, UK

**Aims** Antigen-presenting dendritic cells (DCs) are important in the anti-cancer response and their deficiency may predispose to the escape of cancer from immune control. We hypothesized that circulating DCs are quantitatively reduced in colorectal cancer and that this is reversed by curative cancer excision, as well as being qualitatively deficient in expressing costimulatory molecules (CD86) that are required for effective antigen presentation.

**Methods** Flow cytometric analyses were performed on blood samples taken pre-operatively and 8 weeks post-operatively. DCs defined as negative for surface expression of CD3, CD14, CD16, CD19, CD20 & CD56 and positive for HLA-DR were measured as a percentage of the mononuclear cell population. The prevalence and intensity of CD86 expression in DCs were also measured.

**Results** 67 colorectal cancer patients, 51 healthy controls and 25 patients with inflammatory bowel diseases were studied. Pre-operatively there was a significant reduction in the percentage of circulating DCs in colorectal cancer patients (median = 0.25%) compared with controls (0.41%; Mann–Whitney U test, $P < 0.0001$) and patients with colonic inflammation (0.39%, $P = 0.005$). There was no significant increase in the other mononuclear cells indicating an absolute decrease in the number of DCs. The extent of DC reduction did not relate to cancer stage. Post-operatively DCs increased significantly in patients who underwent curative cancer resection (Wilcoxon signed rank test, ($P = 0.0001$) but not in those with residual cancer ($P = 0.33$), control subjects ($P = 0.07$), or inflammatory cases ($P = 0.18$). The pre-operative prevalence of $CD86^+$ DCs was increased above controls (41%) in inflammatory cases (60%; $P = 0.003$) but not in colorectal cancer patients (38%; $P = 0.53$). There was no difference in the immunofluorescent intensity of $CD86^+$ DCs between the three groups before and after surgery.

**Conclusion** In colorectal cancer there is a reduction in circulating DCs that is reversed by tumour excision. The lack of rise in CD86 molecule expression in DCs suggests that antigen presentation is impaired in colorectal cancer. This quantitative and qualitative DC deficiency may be one mechanism by which colorectal cancer escapes from immune destruction.

### P41 FLOW CYTOMETRY CORRELATES WITH RT-PCR FOR *IN VITRO* BUT NOT *IN VIVO* DETECTION OF CIRCULATING COLORECTAL CANCER CELLS.

G Tsavellas, H Patel, A Huang, R Araia, C Glover, TG Allen-Mersh, Department of Academic Surgery, ICSM, Chelsea and Westminster Hospital, London, UK

**Aims** Circulating tumour cells (CTCs) may predict survival in colorectal cancer, but methods for their detection require validation. We developed a flow cytometric assay for CTC detection and compared its sensitivity with the reverse transcription polymerase-chain reaction (RT-PCR) as a clinically validated gold standard.

**Methods** Peripheral venous blood from healthy 'no-cancer' patients was spiked with HT29 colon cancer cells and analysed using flow cytometry (FACS). Cancer cells were defined as positive for pan-cytokeratin and negative for CD45 pan-leucocyte antigen. Blood (3 × 28 ml) from primary colorectal cancer patients ($n = 15$) and 'no cancer' controls ($n = 22$) was equally divided for FACS analysis as above, and RT-PCR for CEA and CK20 cDNA.

**Results** In non-spiked control blood, the median number of cytokeratin positive/CD45 negative events detected by FACS was 0 (upper 95 percentile = 13). In spiking experiments using FACS, there was a significant correlation between the number of spiked cells and their recovery ($r^2 = 0.89$, $P < 0.05$), with a mean cell recovery of 78%. With an upper normal threshold of 13 events, the lowest detectable abnormal concentration was 20 spiked cells in 14 ml blood (median recovery = 14 cells). An abnormal spiked cell result on FACS (> 13 cells) concurred significantly with a positive RT-PCR result ($P < 0.05$, Fisher's Exact test). FACS detection of tumour cells was significantly higher in spiked blood (at the lower sensitivity limit of 20 cells in 14 ml) compared with blood from cancer patients (Mann-Whitney U test, $P < 0.05$). No difference was identified by FACS, between blood taken from healthy controls (median 0 events, iqr = 0–3) and colorectal cancer patients (median 2 events, iqr = 0–3). There was no concordance in CTC detection between FACS and RT-PCR in cancer patient blood ($P = 0.8$, Fisher's Exact test).

**Conclusion** FACS detection of tumour cells is feasible in-vitro and correlates well with RT-PCR. Its sensitivity in-vivo is poor and does not correlate with RT-PCR detection of CTC's. Reasons for this might include in-vivo cell aggregation, heterogeneity of cytokeratin expression or fragility of CTC's, compared with cultured tumour cells.

### P42 IDENTIFICATION OF DIFFERENTIALLY EXPRESSED PROTEINS IN COLORECTAL CANCER

LC Lawrie[1,2], GI Murray[2], Departments of [1]Molecular & Cell Biology, [2]Dept. of Pathology, University of Aberdeen, Aberdeen, UK

Colorectal cancer is one of the most common malignancies in the UK with 30,000 new cases and 20,000 deaths from the disease each year. Improving prognosis requires a greater understanding of the biology of colorectal cancer (CRC) and the identification of molecular markers of CRC which may be useful as novel therapeutic targets or as diagnostic aids. Proteomics is a powerful analytical tool which can help elucidate mechanisms of disease development and progression by comparing differences in protein expression profiles from normal or colonic tumour tissues.

We have examined fresh frozen paired samples of normal and tumour tissue from patients with Duke's stage C adenocarcinomas located in the sigmoid colon, which were selected from the Aberdeen Colorectal Cancer Database. Two-dimensional gel electrophoresis was performed to produce protein expression maps from samples of normal and tumour tissue. Image analysis of the gel maps identified proteins which showed altered expression. These proteins were excised from the gel and subject to mass spectrometric analysis then identified through database searching. As well as proteomic examination of bulk normal and tumour tissue, we have also used immunomagnetic beads to obtain distinct populations of tumour or normal colonic epithelial cells for proteome analysis.

Initial results have shown that there are a significant number of differences in protein expression in colon cancer compared with normal colonic epithelium. Proteins which have shown increased expression in tumours include; peptidylprolyl cis-trans isomerase, calgranulin B and triosephosphate isomerase. Proteins which have been identified as showing decreased expression in colon tumours include; smooth muscle protein 22-alpha, heat shock 27 kDa protein, tropomyosin alpha chain and epithelial muscle type, alpha-1-antitrypsin precursor, myosin and desmin. Proteomic analysis of normal and colonic tumour tissues can be expected to provide a further insight into the molecular mechanisms of colorectal cancer tumour development and progression.

### P43 A PILOT STUDY OF THE USE OF OXALIPLATIN IN METASTATIC COLORECTAL CANCER AT VELINDRE

SS Mitra, AE Brewster. T Crosby, TS Maughan Velindre Hospital, Cardiff, CF4 7XL, UK

**Aims** Metastatic colo-rectal cancer has a median survival of 12–18 months. Newer drugs like Irinotecan and Oxaliplatin could improve survival.(1) (2) Is Oxaliplatin a safe and effective drug in metastatic colorectal cancer?

**Procedures and methods** We studied 31 patients with metastatic colorectal cancer who were treated with Oxaliplatin chemotherapy between September 1998 and April 2000. Out of the 31 patients, 3 patients had Oxaliplatin with 5FU chemotherapy as 1st line treatment while 28 had the drug as 2nd or 3rd line. The age of the patients ranged from 27 to 72 years with a median age of 60 years. Out of the 31 patients. 20 had metastases in the liver. 9 had recurrent disease in the abdomen, 4 had metastases in the lungs. 2 had skeletal metastases and I had mediastinal lymphadenopathy.

**Results** 2 of the 3 patients on 1st line Oxaliplatin had partial responses while the third patient has just completed 3 cycles and is unassessable.26 patients had 5FU as first line chemotherapy and 11 had a partial response to 5FU while 12 had progressive disease while on the 5FU.3 patients had stable disease on the 5FU. 1 patient had Tomudex and another had CPT-11 with Tomudex as first line treatment. 21 of the 28 patients had Oxaliplatin as 2nd line chemotherapy while 7 had it as 3rd line chemotherapy. There were no complete responses, 8 partial responses and 8 patients had stable disease. The duration of response varied from 0-11 months with a median of 4 months. 6 patients progressed on chemotherapy while 2 were not assessable because they are still on chemotherapy. Of the 7 patients who had Oxaliplatin as 3rd line chemotherapy, 3 had progression. 3 had stable disease while I patient had a partial response. Among the 28 patients, 22 had Oxaliplatin with 5FU while 6 had Oxaliplatin alone. Of the 6 patients who had Oxaliplatin as a single agent, 2 had a partial response. 2 progressed while 2 had stable disease. I patient died from renal failure on chemotherapy, 10 out of 31 patients had grade 1–2 peripheral sensory neuropathy aggravated by cold and particularly affecting the larynx in 3 patients. 6 patients had grade 2 nausea and vomiting, 2 had acute diarrhoea and 4 patients had neutropenic sepsis.

**Conclusions** Oxaliplatin used in combination with 5FU is a highly effective and safe drug in metastatic colorectal cancer with a partial response rate of 33% and stable disease rate of 28.5%. The duration of response is short with a median response of 4 months. It is effective even in 5FU refractory disease. The data suggests it should be used early on in the natural history of metastatic colorectal cancer.

1. Cunningham D et al A phase III multicentre randomized study of CPT-II versus supportive care (SC) alone in patients (Pts) with 5 FU – resistant metastatic colorectal cancer (MCRC). *Proc.ASCO* 17:A–1. Ia. 1998
2. Andre T, et al: Multicentre phase II study. *JCO* 17(11): 3560–3568. 1999

### P44 LOW DOSE FOLINIC ACID WITH INFUSIONAL AND BOLUS 5FLUOROURACIL IN ADVANCED COLORECTAL CARCINOMA

M Osborne, N Shah, PJ Hoskin, MG Powell, R Anthony-Pillau, R Glynne-Jones, A Makris. Mount Vernon Hospital, Northwood HA6 2RN, UK

**Aim** The de Gramont schedule incorporates bolus and infusional 5 fluorouracil (5FU) with high dose folinic acid (FA) and is widely used in the treatment of advanced colorectal carcinoma. However the benefit of high dose FA is uncertain. This study assesses the activity of low dose FA in the de Gramont schedule in metastatic colorectal carcinoma.

**Patients and Methods** 82 patients (49 male, 33 female), median age 68 years (range 35–79) were treated with a modified de Gramont schedule consisting on days 1 and 2 of FA at a dose of 20 mg/m$^2$ administered intravenously over 30 minutes, followed by 5FU bolus at 400 mg/m$^2$, then 5FU infusion 400 mg/m$^2$ (65 patients) or 600 mg/m$^2$ (17 patients) over 22 hours. Chemotherapy was given every two weeks to a total of six courses. Sites of metastatic disease were: liver 66 patients, lung 10, peritoneum 18, local recurrence 12, nodes 12 with 54 patients presenting at original diagnosis with metastatic disease. 14 patients were previously treated with adjuvant 5FU. Response assessment was radiological (37), with tumour markers (33) and clinical (12).

**Results** Median follow up 4.3 months (range 1–16). 27/82 (33%) failed to complete 6 courses, 25 due to uncontrolled disease, 1 due to toxicity. 7 patients required a delay, 4 due to neutropenia. Grade 3 neutropenia was recorded in 4/82(5%), Grade 3 diarrhoea in 4/82(5%), Grade 3 nausea in 1/82(1%). Overall response achieved: CR 2/82(2%), PR 32/82(39%), SD 16/82(20%), PD 32/82(39%).

**Conclusion** Low dose FA in combination with bolus and infusional 5FU is comparable to the standard de Gramont schedule with high dose FA in terms of toxicity and response rates. Further follow up is required to fully evaluate duration of response but potential cost benefit with the reduced dose of FA is important.

### P45 AN UPDATE ON THE MRC FOCUS/CR08 TRIAL: THE FIRST 300 PATIENTS

Matt Seymour on behalf of the MRC Colorectal Cancer Group and all the participants, Cancer Division, MRC Clinical Trials Unit, 222 Euston Road, London, UK

FOCUS is a large multi-centred randomised trial designed to assess the role of irinotecan and oxaliplatin in advanced colorectal cancer. Compared with the best evidence-based regimen – a Modified de Gramont 5FU/FA schedule (MdG), followed on progression by single agent irinotecan – FOCUS investigates each of the new agents in combination with MdG, as either first-line or second-line treatment.

Because of the relatively new status of both irinotecan and oxaliplatin, and the specialised demands of the new regimens on doctors, nurses and pharmacists, general minimum requirements, along with specific staff experience, are required before an Oncology Centre/Unit can be registered for participation in FOCUS.

A total of 2100 patients are required, and the first patient was entered in May 2000. After a slow start, accrual is now close to the target rate, and 300 patients had been entered from 36 centres by the end of January 2001. The pre-treatment characteristics of the patients were: median age 62 years (range 27–83 years); male 70%; WHO performance status 0 – 41%, 1–51%, 2–8%; colon cancer 55%; stoma 30%; objective measurable evaluability of disease 93%; resected primary 70%, unresected/unresectable 26%, local recurrence 4%; metastases 98% (liver mets 80%, nodes 32%, lung mets 30%, peritoneum 10%).

Data is available on 90 patients who have reached their 12-week re-assessment point. All patients received their allocated regimen, 80% receiving 6 cycles. Response was reported as complete 5%, partial 26%, stable (or no evidence of progression) 46%, progressed 11%, died 12%.

19 serious adverse events considered to be related to treatment have been reported. These were mainly related to prolonging hospitalisation (9) and venous access problems (7). None were fatal or life threatening. 16 patients have died (all disease-related except 1 who died following surgery for bowel obstruction).

The trial is accruing extremely well, and the increased emphasis on Quality Assurance appears to be resulting in a low level of serious adverse events, excellent compliance to treatment plans and good clinical form return and QL completion.

### P46 A COMPARISON OF DOSE AND SCHEDULING EFFECT OF 5-FU CHEMOTHERAPY IN ADVANCED COLORECTAL CANCER

PA O'Neill, DB Smith, E Marshall, PI Clark, SM O'Reilly, Department of Medical Oncology, Clatterbridge Centre of Oncology, Bebington, Wirral, Merseyside, CH63 4JY, UK

We conducted a single centre randomised trial to investigate the schedule and dose effect of 5 fluorouracil and folinic acid (5FU/FA) in advanced colorectal cancer.

We compared the maximum tolerated dose (650 mg/m$^2$) of 5FU/FA given as a bolus injection with the same dose by continuous intravenous infusion (CI) and with a third group given 50% higher dose (975 mg/m$^2$) of 5FU/FA by CI. All patients received FA at the same dose of 20 mg/m$^2$.

103 patients were randomised. The mean age for all patients was 63.1 years. Chemotherapy was completed as planned in 59% patients and 8% stopped early due to progressive disease. Delays occurred in 21% patients and dose reductions in 11%. Hand-foot syndrome was more common in the 975 mg/m$^2$ group ($P = 0.08$), as was mucositis ($P = 0.001$) whereas diarrhoea more frequent in the bolus group ($P = 0.02$). There was a higher incidence of cardiac events in the 975 mg/m$^2$ group ($P = 0.035$). Response rate was highest in the 975 mg/m$^2$ CI group at 21% Vs 8% in the bolus group. Median PFS was 4.6 months (bolus), 5.9 months (650 mg/m$^2$ CI) and 8.1 months (975 mg/m$^2$ CI) for the three groups (NS). There was a trend towards a longer median OS for the 975 mg/m$^2$ CI group at 10.7 months Vs 8.1 months for bolus and 8.5 months for 650 mg/m$^2$ CI group ($P = 0.3$).

These results suggest a dose intensity effect rather than a scheduling effect for the increased efficacy of CI schedules over bolus and highlight the potential for significant cardiac toxicity including sudden death in higher dose regimens given by continuous infusion.

### P47 RESECTION OF COLORECTAL LIVER METASTASES: A SYSTEMATIC REVIEW

J Colquitt[1], P Simmonds[1], J Primrose[1], M Rees[2], G Poston[3], [1]Cancer Sciences Division, Southampton University School of Medicine, [2]Dept. of Surgery, Basingstoke Hospital, RG24 9NA, [3]Dept. of Surgery, Royal Liverpool Hospital, L7 8XP, UK

Hepatic resection for colorectal metastases is increasingly being undertaken, although the efficacy has never been evaluated in a randomised controlled trial. Case series demonstrate 5 year survival up to 40% following curative resection, whereas 5 year survival in unresected patients is rare. However, patients undergoing resection may be a highly selected group with a better prognosis. Aim: to determine morbidity and survival following liver resection for colorectal metastases and to identify potential prognostic factors. Methods: A systematic review of published and unpublished studies was conducted. Studies were identified by computerised searches of databases and specialist registers, handsearching, scanning references and contacting investigators. Eligibility and quality of studies were assessed independently by 2 reviewers and data were extracted using a standard form. To be eligible for inclusion studies must have included at least 100 patients and had a median follow up of 24 months for surviving patients. Investigators were contacted where information was insufficient to determine eligibility. Preliminary results: of 517 identified publications, 48 (including multiple publications of the same study) met inclusion criteria, providing 28 eligible studies. Most common reasons for exclusion: 409 (79%) reported less than 100 patients, 379 (66%) had a follow-up of less than 24 months median or not reported. Six eligible (21%) studies were multicentre, 20 (71%) were single centre and 2 (7%) were from two centres. Seven (25%) studies were prospective, 19 (68%) were retrospective and 2 were unclear. The quality of studies varied. Eligibility criteria for surgery or inclusion in the study were not reported by 8 (29%) studies, and principal confounders (e.g. synchronous metastases, curative intent) were not reported by 7 (25%) studies. Primary end points such as postoperative mortality and survival were not clearly defined in 12 (43%) and 22 (79%) studies respectively. Operative mortality was excluded from survival analysis in 6 (21%) studies. Surgery was the only intervention received by a defined group of patients in just 5 (18%). In 12 (43%) studies additional therapies were received by all patients or by a subgroup who were not analysed separately, and use of additional therapies was not reported in 11 (39%) studies. Overall 5 year survival ranged from 25% to 58% although patient characteristics varied enormously. Conclusions: Although the many published studies, in general. suggest a substantial benefit from surgical resection, the identification of patients most likely to benefit is limited due to methodological weaknesses and inconsistencies between studies. Further summary information on the outcomes from these studies and potential prognostic factors will be presented.

### P48 CULTURED PURIFIED NORMAL BREAST EPITHELIAL CELLS RAPIDLY LOSE OESTROGEN RECEPTOR ALPHA (ER) EXPRESSION, WHEREAS PURIFIED MALIGNANT BREAST CELLS STABLY MAINTAIN THEIR ER STATUS

MS Kothari, M Slade*, S Ali*, HD Sinnett*, S Shousha*, N Livni*, L Buluwela*, R Vashisht[+], P Thorpe[+], RC Coombes*, *Imperial College School of Medicine and [+]West Middlesex Hospital, London, UK

The aim of this study was to purify malignant cells from primary breast cancers, characterise and culture them for a time length sufficient to be able to perform functional studies on them. Fresh pathological discard tissue was enzymatically digested and after appropriate filtration malignant epithelial cells were isolated using Ber-Ep4 immunolabelled beads (Dynal). Cells were maintained in short-term culture and characterised by cytology, fluorescent in situ hybridisation (FISH) (using centromere specific DNA probes to chromosomes 6,7,11,12,17 and 18) and immunostaining for oestrogen receptor alpha (ERα), progesterone receptor (PR) and cytokeratins 8 and 18. Normal epithelial cells were purified from reduction mammoplasty tissue and cultured and immunostaining done using the above markers. In addition these and MCF-7 cells served as controls. Cytological examination confirmed >95% purity of malignant cells in all 15 tumor samples stained for Haematoxylin and Eosin. A high rate (>90%) of aneusomy was observed by FISH on cells from all 6 tumours studied. These were also confirmed to be pure epithelial cultures using cytokeratin staining. In all 10 tumor samples studied for ER and PR it was found that these were retained in malignant cultures for the entire time length studied (7–14 days) but completely lost within 24 hrs (ER) to 60 hrs (PR) in the 6 benign cultures studied. Furthermore purified normal breast cells proliferate in culture, whereas malignant cells do not, although they can be maintained in culture for 3–4 weeks. We conclude that (a) it is possible to purify and maintain breast cancer cells for a sufficient period to permit functional studies and (b) ER is retained enabling us to use these cells for studies of endocrine resistance in vitro.

### P49 ATTENUATED OESTROGEN RECEPTOR ALPHA EXPRESSION FOLLOWING PURIFICATION OF NORMAL MAMMARY EPITHELIAL CELLS CAN BE RESTORED BY ADENOVIRUS MEDIATED GENE TRANSDUCTION

MS Kothari, RS Tolhurst, H Yahaya, MJ Slade, S Shousha, S Ali, L Buluwela, RC Coombes, Cancer Cell Biology Section, Division of Medicine, Imperial College School of Medicine, London, UK

Over half of all breast cancers overexpress oestrogen receptor alpha (ERα) and around 70% of these respond to anti-oestrogen therapy, clearly demonstrating the importance of oestrogen receptor in breast cancer. However, there is little data regarding the importance of ERα overexpression in breast cancer initiation. In this regard, there has been little success in the production of cultured luminal epithelial cells that express steroid receptors and respond to ovarian steroids. In the present study we have evaluated the expression of ERα in normal human mammary epithelial cells seeded in culture following immunomagnetic purification. To do this, luminal epithelial cells were isolated from normal reduction mammoplasty tissue using Ber-Ep4 immunolabelled beads (Dynal) and seeded in culture. Cytokeratin 8 and 18 immunostaining confirmed that these preparations were of a very high purity (>97%). Further, immunostaining analysis showed that ERα expression was restricted to between 15–30% of the cells immediately following purification. However, on subsequent culturing, we found that ERα expression was rapidly attenuated, resulting in loss of expression within the first 24–36 hours. There is a similar reduction in progesterone receptor expression, although very low levels can be detected for up to 60 hrs following culture. RT PCR experiments have further confirmed these findings. Together our results suggest that endogenous ERα expression is unstable in normal primary luminal cells in culture. However, in further experiments, we have gone on to show that purified primary luminal epithelial cells can be efficiently transduced with a recombinant adenovirus encoding the human oestrogen receptor. By doing this, we have found that it is possible to maintain ERα expression in luminal epithelial cells for the lifetime of primary cultures, thereby facilitating further studies to address the consequence of ERα overexpression in normal human mammary epithelial cells.

### P50 DIFFERENTIAL PROTEIN TYROSINE PHOSPHATASE EXPRESSION IN BREAST CANCER

M Rafferty, L McArdle, P Dervan, D Easty, Pathology Department, University College Dublin, Belfield Dublin 4, Ireland

In breast cancer, proliferation and differentiation is governed by protein phosphorylation. Tyrosine phosphorylation is particularly important and is regulated by the opposing actions of protein tyrosine kinases (PTKs) and protein tyrosine phosphatases (PTPs). Many PTKs encode oncogenes involved in initiation and progression of breast cancer. PTPs may act as tumour suppressor genes by antagonizing the effects of PTK. Alternatively they may augment the oncogenic effect by repriming substrates for PTKs.

In this study, PTP expression in mammary epithelial cells (HMEC) was compared to that in malignant breast cell lines. Degenerate RT-PCR was carried out and resultant products were cloned and sequenced. 107 and 108 clones were isolated from ZR75-1 and from HMEC respectively. Seventeen PTPs were isolated and used as probes for northern blotting. Northern blot analysis showed a down regulation of four genes, PTP-KAPPA, PTP-EPSILON, PTP-GAMMA and PTP-BAS, in malignant cell lines compared to HMEC. PTP-PEST, TC-PTP, PTP-1B, PTP-ALPHA, LAR and DEP-1 were up-regulated in cell lines. Further analysis for PTP-BAS at the protein level is currently being carried out using western blotting and immunohistochemistry techniques.

### P51 AKT2 IS DIFFERENTIALLY REGULATED IN HUMAN BREAST CANCER CELL LINES

KM Nicholson, CH Streuli, NG Anderson, School of Medicine, University of Manchester, Oxford Road, Manchester, M13 9PT, UK

Akt (protein kinase B) is a family of serine/threonine kinases consisting of three isoforms (Akt1/PKBα, Akt2/PKBβ and Akt3/PKBγ) with an important role in protecting cells from the induction of apoptosis by various stresses such as growth factor depletion and detachment of cells from their growth matrix. Activation of Akt in response to hormonal or growth factor stimulation is via the lipid products of phosphatidylinositol-3-kinase. Once activated Akt can phosphorylate and negatively regulate various pro-apoptotic targets within the cell such as BAD and members of the forkhead family of transcription factors including FKHR, FKHRL1 and AFX. Consistent with the role of Akt in cell growth and survival, Akt is known to be over-expressed and/or amplified in various human cancers (Akt1 in gastric, Akt2 in ovarian and pancreatic, Akt3 in breast). To date there is little information regarding the role of the individual isoforms in the processes involved in tumorigenesis, therefore this study aimed to determine the expression, activation and regulation of the individual isoforms of Akt in breast cancer. Nine breast cell lines were used in this study – 2 normal (MTSV1-7 and MCF10F), 2 estrogen receptor (ER) positive cancer cell lines (T47D and MCF7) and 5 ER negative cancer cell lines (MDAMB231, SKBR3, BT474, HS578T and CAL51). The expression and activity of each isoform was assessed using isoform-and phospho-specific antibodies. In addition, the expression of PTEN, a negative regulator of Akt often deleted or mutated in human cancers resulting in a high constitutive activation of Akt, and the expression and phosphorylation of FKHR, a downstream target of Akt were determined. Results showed that Akt1 activation in the presence of serum (steady-state) was similar across the cell line panel. Akt3 was active in only one breast cancer cell line (HS578T) at steady state. More interestingly, Akt2 activation at steady state was increased in 6/7 breast cancer cell lines relative to the normal breast cell lines (range 9–23 fold). Phosphorylation of FKHR did not correlate with Akt activation in these cell lines. Loss of PTEN expression correlated with a high level of Akt activation but not with phosphorylation of FKHR. Cell lines with all three isoforms activated at steady state were chosen to investigate the effects of EGF, IGF-I or heregulin α on the activation of each isoform of Akt. In each cell line Akt2 was activated to a greater extent than either Akt1 or Akt3 following growth factor stimulation, indicating a differential regulation of Akt2.

Collectively these results suggest an involvement of Akt2 in breast cancer growth and survival. Overexpression studies are ongoing to further elucidate the involvement of Akt2 in tumorigenesis.

This work was funded by the Breast Cancer Campaign, registered charity no 299758

### P52 SERUM OESTROGEN MODULATES THE IN VIVO LEVEL OF P53 EXPRESSION IN BREAST CANCER XENOGRAFTS

D Ziyaie[1], D Dornan[2], TR Hupp[2], NM Kernohan[2], AM Thompson[1], [1]Dept. of Surgery, Molecular Oncology, [2]Dept. of Molecular, Cellular Pathology, Ninewells Hospital, Dundee DD1 9SY, UK

**Background** Breast cancers, unlike most other epithelial malignancies often acquire abnormalities in p53 protein activity rather than p53 gene mutations. It is therefore postulated that these functional abnormalities in p53 activation may be influenced by specific cellular/environmental changes rather than genetic mutations. In breast cancer, activation of oestrogen receptor proteins is one of the most important regulatory events involved in the pathogenesis of these tumours. Although functional links between p53 and an individual's serum hormone levels have been explored previously the results have been inconclusive and a consistent relationship between p53 protein expression and circulating serum oestrogen hormone levels has not been defined.

**Methods** We have investigated p53 expression in response to serum oestrogen concentrations along with its two key gene products p21 WAF1 and MDM-2 which are known to be transcriptionally induced by p53 in a xenograft model established from an MCF-7 breast cancer cell line. Protein expression in relation to the varying oestrogen serum concentrations was determined by western blot analysis and further confirmed on histological sections using highly sensitive human specific p53 monoclonal antibodies.

**Results** Though immediately prior to the injection of oestrogen, the p53 protein levels exhibited strong immuno-reactivity, within hours following the injection, protein levels were sharply reduced and continued to be repressed in the presence of high serum oestrogen concentration. Following the natural reduction in hormone levels however, p53 protein levels rose coincident with the lowering levels of the serum oestrogen prior to a repeat bolus injection. Similar trends were observed with p21WAF1 whose expression also correlated inversely with serum oestrogen concentrations. However another key effector, MDM2 protein, did not change significantly under the same conditions highlighting the striking specificity that hormone levels have on the p53-p21WAF1 axis.

**Discussion** An inverse relationship between p53 protein and p21WAF1 protein expression compared with serum oestrogen concentration highlights a potential link between hormone level and the regulation of p53 activity rather than p53 degradation since MDM-2 protein levels remain constant under conditions where p53 protein and p21WAF1 levels respond to hormone changes. MDM2 protein levels are often uncoupled from p53 activity, and the fact that MDM2 protein levels are unperturbed during these hormone fluctuations provided, with hindsight, an excellent internal control for hormone specificity.

**P52 Cont'd**

**Conclusion** A functional correlation between oestrogen and p53 protein levels introduces novel practical implications in terms of the timing of drug delivery particularly in pre-menopausal women where the anti-cancer treatments are directed towards inducing cell death or apoptosis, which is one of the key properties that defines p53 as a tumour suppressor gene.

### P53 BIOCHEMICAL AND MOLECULAR MECHANISMS OF GROWTH INHIBITION IN HUMAN BREAST CANCER CELLS BY SULFAMOYLATED ESTRONE DERIVATIVES

L Wood,[1] LW Woo[2], A Purohit[2], BVL Potter[2], MJ Reed[3], G Packham[1], [1]CRC Wessex Medical Oncology Unit, Cancer Sciences Division, University of Southampton, SO16 6YD, [2]Wolfson Laboratory of Medicinal Chemistry and Sterix Ltd, University of Bath, BA2 7AY, [3]Endocrinology and Metabolic Medicine and Sterix Ltd, Imperial College, London W2 1NY, UK

We have previously shown that the sulfamoylated estrone derivatives, 2-Methoxyestrone-3-*O*-Sulfamate (2-MeOEMATE) and 2-Ethylestrone-3-*O*-Sulfamate (2-EtEMATE), potently inhibited cell growth, caused mitotic arrest and induced apoptosis in a panel of estrogen receptor positive and negative breast cancer cell lines (MacCarthy-Morrogh et al., 2000, Cancer Res 60:5441). The sulfamoylated estrone derivatives inhibited tubulin polymerisation in vitro, and induced BCL-2 phosphorylation and p53 expression in intact cells. The sulfamoylated compounds were significantly more active in all of these assays relative to their parental compounds or the endogenous estradiol metabolite 2-Methoxyestradiol (2-MeOE2). The aim of our present study is to discover the molecular basis for this enhanced activity.

We have used competitive binding assays to further investigate the interactions of the compounds with the colchicine binding site of tubulin. Although 2-MeOEMATE and 2-EtEMATE inhibited the ability of paclitaxel to induce in vitro polymerisation of tubulin more potently than 2-MeOE2, 2-MeOEMATE and 2-EtEMATE bind directly to the colchicine binding site with similar affinities to that of 2-MeOE2. This suggests that the sulfamoylated compounds may interact at multiple sites on the tubulin molecule, or that their interaction with the colchine binding site results in more potent effects on tubulin dynamics. Although, 2-MeOE2 has recently been shown to inhibit superoxide dismutase (SOD) activity, it is not known whether this activity is also enhanced by sulfamoylation and might contribute to the enhanced biological actions of 2-MeOEMATE and 2-EtEMATE. We have determined the effects of a series of estrone and estradiol derivatives on SOD activity. Most importantly, sulfamoylation completely destroys SOD inhibitory activity. Therefore, the enhanced activity of the sulfamoylated compounds correlates with increased tubulin polymerisation inhibitory actions (but not higher affinity binding) rather than increased SOD inhibitory activity.

Finally, we have shown that 2-MeOEMATE and 2-EtEMATE upregulate expression of the DR5 (TRAIL Receptor 2) death receptor in CAL51 breast cancer cells, and sensitised these cells to TRAIL induced apoptosis. Upregulation of DR5 by 2-MeOEMATE and 2-EtEMATE and their co-operation with TRAIL to induce apoptosis in breast cancer cells may have important implications for anti-cancer therapies.

### P54 CYP24 ACTION IN BREAST CANCER CELLS

AE Miles, JS Moore, FL Ng*, KW Colston*, MJ Campbell, Division of Medical Sciences, University of Birmingham, B15 2TH, *St. Georges Hospital Medical School, London

Breast cancer cell lines display a wide range of sensitivities to the induction of growth arrest by 1α,25dihydroxyvitamin $D_3$ (1α,25$(OH)_2D_3$) despite comparable expression of the vitamin D receptor (VDR). 1α,25$(OH)_2D_3$ induces its own metabolism by induction of *CYP24*, (25(OH)D3-24-hydroxylase), which generates sequentially 1α,24,25$(OH)_3D_3$, 1α,25$(OH)_2$-24-oxo-$D_3$, 1α,23,25$(OH)_3$-24-oxo-$D_3$ and ultimately calcitroic acid. This gene has recently been described as a putative oncogene in breast cancer although the mechanism remains unclear.

Using an in-house antibody to CYP24 we have examined expression levels in a panel of breast cancer cell lines (MCF-7, ZR-75-1, T47-D, MDA-MB-231). In all these cell lines we have found higher protein expression in exponentially proliferating than confluent cells, correlating with elevated cyclin E and increased numbers of cells in S and $G_2M$ phases of the cell cycle. Screening of proliferative responses to each CYP24 metabolite in soft agar and liquid culture revealed that in both the estrogen responsive MCF-7 and estrogen insensitive MDA-MB-231 cells the 1α,24,25$(OH)_3D_3$ metabolite demonstrated significantly reduced antiproliferative effects compared to 1α,25$(OH)_2D_3$ and 1α,25$(OH)_2$-24-oxo-$D_3$, and, in the case of MDA-MB-231, significant growth stimulation at physiological doses ($10^{-10}$ M, ($P < 0.01$) ). 1α,25$(OH)_2$-24-oxo-$D_3$ and 1α,23,25$(OH)_3$-24-oxo-$D_3$ were potent inducers of apoptosis and differentiation of MCF-7 cells but were ineffective in MDA-MB-231 cells.

We have also screened cell lines and primary breast tumour material to test for CYP24 amplification. Using a PCR-based analysis we have found CYP24 gene amplification in MCF-7 and ZR-75-1 cell lines and 1 out of 24 primary samples.

These studies indicate that breast cancer cells express CYP24 and that highest basal expression is associated with exponential proliferation. CYP24 metabolism modulates the antiproliferative effects of 1α,25$(OH)_2D_3$ by giving rise to ligands with divergent biological effects; we hypothesise that the balance of these actions normally integrates proliferation and differentiation. Malignant transformation may involve suppression of the antiproliferative and pro-apoptotic signals of 1α,25$(OH)_2D_3$ and its metabolites thereby deriving a clonal advantage.

### P55 INHIBITORY EFFECTS OF BISPHOSPHONATES ON GROWTH OF BREAST AND LUNG CARCINOMA CELLS

CD Macdonald, SG Jayasundara, JL Mansi, KW Colston, Department of OGEM St George's Hospital Medical School, London SW17 0RE, UK

Breast and lung cancer commonly metastasize to bone, causing pain and impaired mobility in addition to hypercalcaemia. Bisphosphonates (BP) are a group of drugs which are commonly used in the treatment of certain bone diseases due to their inhibitory effects on osteoclasts.

In patients with advanced breast cancer and bone metastases, BPs have been used to reduce the incidence of hypercalcaemia and skeletal morbidity. As yet few studies have addressed their effectiveness in lung cancer patients. We are studying the problem in a new way to determine if BPs may have direct effects on breast and lung cancer cells. This study investigated the in vitro effects of the BPs, zoledronic acid, pamidronate and clodronate on the growth, viability and induction of apoptosis in three lung cancer cell lines: NCI H292 (mucoepithelial) NCI H69 and NCI H510 (small cell carcinomas). Cells were cultured in multiwell plates for up to 3 days in the presence or absence of BPs (0–2000 μM). Results were compared with MDA-MB231 breast cancer cells.

Cell growth was monitored by Crystal Violet dye assay and Sulphorhodamine B, while cell viability was quantitated by MTS dye reduction. Changes in apoptosis related protein were determined by assessing bcl-2/bax ratio and cleavage of pro-caspase-3 into 17 kD & 11 kD fragments by immuno blotting. All three BPs induced inhibition of growth and viability in MDA-MB-231 breast cancer cells. This was accompanied by a decrease in bcl-2/bax ratio and pro-caspase-3 cleavage. BPs also reduced cell number in the 3 lung cancer cell lines by day 3 of treatment. NCI H292 cells were the most sensitive, displaying a significant dose related inhibition of cell growth and viability. A Time related decrease in bcl-2/bax ratio was seen in zoledronic acid treated NCI H292 cells. In contrast NCI H510 and NCI H69 both showed an increase in bcl-2/bax ratio suggesting that reduction in cell number may be due to inhibition of cell growth but not apoptosis. Taken together our results suggest that BPs have direct inhibitory effects on both lung and breast carcinoma cells in vitro. Both NCI H69 and NCI H510 cells grow as suspension cultures while NCI H292 cells are adherent. An integrin dependent pathway may be associated with BP induced apoptosis.

### P56 ZOLEDRONIC ACID AND PACLITAXEL HAVE SYNERGISTIC EFFECTS ON BREAST CANCER CELL APOPTOSIS IN VITRO EVIDENCE FROM ISOBOLGRAM AND GRAFIT SOFTWARE ANALYSIS.

S Jagdev[1], P Croucher[2], A Rostami-H[2], R Coleman[1], [1]YCR Dept of Clinical Oncology, [2]Human Metabolism and Clinical. Biochemistry, University of Sheffield, UK

Bisphosphonates are becoming increasingly important in the management of bone metastases from breast cancer. Recent clinical data suggest that the adjuvant use of bisphosphonates alongside anti-neoplastic agents may influence the development of extra-skeletal as well as skeletal metastases and confer a survival benefit. These observations raise the intriguing possibility that bisphosphonates may act in synergy with anti-neoplastic therapy. We have previously shown that the potent third generation bisphosphonate zoledronic acid reduces breast cancer cell number and induces apoptosis after long-and short-term exposure in vitro. The aim of this study was to investigate the effects of combining zoledronic acid with paclitaxel on MCF-7 cell number and apoptosis.

MCF-7 cells were treated with increasing concentrations of zoledronic acid (0.01–100 μM) alone and in combination with increasing concentrations of paclitaxel (0.01–10 nM). Cell number was determined and the proportion of apoptotic cells assessed by analysis of nuclear morphology. Dose response curves were plotted for the effect of each combination of drugs on cell number and apoptosis. These were used construct isobolgrams that revealed evidence of synergistic effects on MCF-7 cell number and apoptosis when zoledronic acid and paclitaxel were combined. However, quantifying the magnitude and assessing statistical significance of synergism required more rigorous non-linear 3D analysis. The latter was carried out by fitting user-defined models of synergism to data using the GraFit software (Ver 3).

The general interaction model (Greco WR et al., 1995) contained a non-additivity term, $\alpha$, and sigmoidicity function for curve shape, $\gamma$. If $\alpha = 0$ then an additive effect could be assumed; higher values indicated synergy. Using the GraFit software, the calculated $\alpha$ and $\gamma$ for the combined effects of zoledronic acid and paclitaxel on cell numbers were 3.31 (±2.33) and 0.90 (±0.13), respectively.

These data suggest that, when combined, zoledronic acid and paclitaxel have synergistic effects on breast cancer cells in vitro. The mechanisms require further in vitro and in vivo study.

1. Greco WR, Bravo G and Parsons J (1995). The search for synergy: A critical review from a response surface perspective. *Pharmacol Rev* **47**: 331–385

### P57 EFFECT OF NITRIC OXIDE ON MMP-9 SECRETION IN MCF-7 ADR AND MDA-MB 231 HUMAN BREAST CANCER CELL LINES

M Lahiri, A Martin, J Martin, School of Health Sciences, University of Wolverhampton, Wolverhampton WV1 1DJ, UK

The aim of the present study was to detect whether endogenously and/or exogenously produced nitric oxide (NO) had an effect on MMP-9 secretion in MCF-7, MCF-7 ADR, T47D and MDA-MB 231 human breast cancer cell lines.

Gelatin zymography analysis was used to detect MMP-9 secretion from the cell lines. MMP-9 secretion was detected in MCF-7 ADR and MDA-MB 231 cells but not in MCF-7 and T47D cell lines under quiescent conditions. The addition of TNF-α or exogenous NO via the NO donor, sodium nitroprusside (SNP) increased MMP-9 secretion in both MCF-7 ADR and MDA-MB 231 cells.

TNF-α treatment of MCF-7, MCF-7 ADR, T47D and MDA-MB 231 cell lines caused an increase in NO production. A decrease in NO production was observed upon the addition of either a general nitric oxide synthase (NOS) inhibitor (L-NAME), an inducible NOS (iNOS) specific inhibitor (AMT) or an endothelial NOS (eNOS) specific inhibitor (L-NIO). However, these inhibitors had no effect on TNF-α stimulated MMP-9 secretion in MCF-7 ADR and MDA-MB 231 cell lines.

In conclusion, our results indicated that the addition of an exogenous NO donor increased MMP-9 secretion but endogenously produced NO, following TNF-α treatment, had no effect on MMP-9 secretion.

### P58 ANTI-PROLIFERATIVE EFFECTS OF THE EGF RECEPTOR TYROSINE KINASE INHIBITOR ZD1839 IN MDA-MB231 BREAST CANCER CELLS

T Ahmad, KC Chan, NJ Bundred, NG Anderson School of Medicine, University of Manchester, Oxford Road, Manchester M13 9PT

Overexpression of the EGF receptor (EGFR) occurs frequently in a number of human cancers and correlates with aggressive tumour behaviour and poor patient prognosis. We studied the effects of a novel orally active EGF receptor tyrosine kinase inhibitor, ZD1839 (Iressa™), on the proliferation and signalling in the EGFR overexpressing MDA-MB231 breast cancer cell line.

ZD1839 dose-dependently inhibited the proliferation of MDA-MB231 cells cultured in serum-containing medium. Proliferation was inhibited by 59±2% at 1 μM ZD1839. The proliferation of MDA-MB231 cells in serum-free medium is maintained by autocrine growth factors secreted as a result of a mutant active K-Ras in these cells. ZD1839 (1 μM) inhibited the proliferation of MDA-MB231 cells in serum free medium by 32±7%.

ZD1839 dose dependently inhibited EGF-induced EGFR tyrosine phosphorylation in MDA-MB231 cells with an estimated $IC_{50}$ of 2 nM. Similarly, ZD1839 inhibited EGF-induced activation of protein kinase B (PKB)/Akt and ERK MAP kinases. However ZD1839 treatment did not reduce the constitutive high basal level of ERK activation due to the presence of mutant K-Ras suggesting that ERK inhibition is not essential for the anti-proliferative effects of ZD1839. The importance of the ERK pathway in mediating proliferation in MDA-MB231 cells was tested using PD098059, an inhibitor of MEK activation. Treatment of cells with PD98059 (100 μM) completely blocked ERK activation and inhibited proliferation by ≈93%. At doses of PD098059 that led to an inhibition of proliferation similar to that achieved by 1 μM ZD1839 (59%) there was a significant reduction in the level of activated ERK. Together, these results confirm that the ERK pathway is required for the maximal proliferation of MDA-MB231 cells. However ERK inhibition is not necessary for ZD1839-induced inhibition of proliferation, suggesting that EGFR utilises ERK-independent pathways to promote proliferation in these cells.

We investigated the role of the phosphoinositide 3-kinase (PI3-K) pathway in the proliferation of MDA-MB231 cells. LY294002 (50 μM), an inhibitor of PI3-K inhibited prolferation by 17%, a result which rules out any major contribution to proliferation by the PI3-K signalling pathway in this cell line. We are currently investigating the roles of other signalling pathways in the proliferation of MDA-MB231 cells.

In conclusion ZD1839 potently inhibits both serum-induced and autocrine regulated proliferation in EGFR overexpressing MDA-MB231 cells via mechanisms that do not involve reduced activation of ERK MAP kinases.

### P59 ANGIOGENESIS IN DUCTAL CARCINOMA IN SITU OF THE BREAST IS ASSOCIATED WITH THE DEVELOPMENT OF AN INVASIVE RECURRENCE

NB Teo[1,2], BS Shoker[1], MC Jarvis[1], C Holcombe[2], L Martin[2], JP Sloane[1], UK, [1]Pathology Department, University of Liverpool, [2]Breast Unit, Royal University Hospital, Liverpool L7 8XP, UK

**Background** Up to 50% of recurrences of ductal carcinoma *in situ* (DCIS) of the breast are associated with invasive carcinoma but to date no pathological or molecular features have been found to predict for the development of invasive disease. For a tumour to invade, it requires the formation of new blood vessels. Previous studies have described a vascular rim around ducts involved by DCIS raising the possibility that the characteristics of periductal vascularisation may be important in determining transformation from in situ to invasive disease.

**Methods** Periductal vascular density and phenotype were determined using morphometry and a panel of anti-endothelial antibodies (von Willebrand factor [vWF], CD31, CD141 and CD34) and related to a) the presence of invasive carcinoma b) other histological features and c) the risk of either invasive or *in situ* recurrence. For each section, the entire number of stained vessels within 100 micrometer of foci of DCIS were counted. Up to 50 foci of DCIS on a single slide were scored and the microvessel density (MVD) was calculated for each focus. Normal lobules at least 2 mm away were used as controls.

**Results** Pure DCIS in comparison to normal lobules exhibited a greater density of CD34+ vessels ($P = 0.004$) but a decrease in those immunopositive for vWF ($P = 0.001$), indicating a change in phenotype as well as density. DCIS associated with invasive carcinoma showed a profile similar to that of pure DCIS but with statistically significantly greater numbers of CD34+ ($P = 0.003$) vessels and fewer staining for vWF ($P = 0.030$). There was a significant negative correlation between vascular density and both the ductal cross-sectional areas (highest $P < 0.001$ except CD31 with $P = 0.300$) and the extent of the necrosis (highest $P = 0.002$). A correlation between vascular density and nuclear grade was also noted, being highest in the intermediate grade. In addition when cases of DCIS that subsequently recurred were compared with those that did not, the former cases of DCIS had a higher CD34 MVD ($P < 0.001$). Furthermore the highest MVD was seen in cases of DCIS that subsequently developed invasive cancer.

**Conclusions** Blood vessels surrounding DCIS appear to have a different immunophenotype when compared with blood vessels surrounding normal breast lobules. Furthermore increases in vascular density, as detected with the CD34 antibody, correlates with recurrence and the development of invasive carcinoma.

### P60 PERIDUCTAL AND STROMAL ANGIOGENESIS IN DUCTAL CARCINOMA IN SITU (DCIS): ITS RELATIONSHIP TO RECURRENCE AND THYMIDINE PHOSPHORYLASE EXPRESSION

NB Teo[1,2]*, BS Shoker[1], CM Jarvis[1], C Holcombe[2], JP Sloane[1],[1]Pathology Department, University of Liverpool, [2]Breast Unit, Royal University Hospital, Liverpool L7 8XP, UK

**Background** Angiogenesis is though to be important in invasive cancer and may also have a role in preinvasive disease. We have previously shown that periductal angiogenesis in DCIS is associated with an increased risk of subsequently developing a recurrence. The aims of the present study were a) to identify the relationship between periductal and stromal vascularity and its association with recurrence and b) to determine whether thymidine phosphorylase (TP) is associated with angiogenesis or recurrence in DCIS.

**Methods** Cases of DCIS which did not subsequently recur ($n = 20$), which developed a subsequent in situ recurrence ($n = 20$) and which developed a subsequent invasive recurrence ($n = 12$) were investigated. Periductal and stromal (hotspot) microvessel density (MVD) was determined quantitatively using CD34 and vWF antibodies. TP expression by DCIS was semiquantitatively assessed using the H-score method.

**Results** Using vWF the stromal and periductal MVD showed a strong positive correlation ($P < 0.001$) with both methods giving similar mean values. In contrast, when angiogenesis was assessed with CD34 this relationship was lost. Not only were the mean values for both stromal and periductal MVD higher than those obtained with vWF, but also the periductal MVD was significantly greater than the stromal MVD (highest $P = 0.037$). Furthermore, a relationship between recurrence and stromal MVD was not seen. For TP, although relationships were seen between the H-score and MVD, a relationship with recurrence was not identified.

**Conclusions** Periductal MVD appears to be more important than stromal MVD in predicting for recurrence after DCIS. However, recurrent disease does not appear to be related to TP expression by DCIS. Periductal vascularity as assessed by CD34 is a better predictor of recurrence than stromal assessment.

### P61 ASSESSMENT OF MICROREGIONAL BLOOD FLOW IN HUMAN BREAST CARCINOMAS AFTER ADMINISTRATION OF ANTHRACYCLINE CHEMOTHERAPY

K Goodchild[1], SA Hill[2], A Sibtain[1], DJ Chaplin[2], A Makris[1], [1]Marie Curie Research Wing, Mount Vernon Hospital, Northwood, HA6 2RN, UK, [2]Gray Laboratory Research Trust, Mount Vernon Hospital, Northwood, HA6 2RN, UK

**Introduction** Anthracyclines are effective anti cancer agents in the treatment of breast cancer. Delivery of systemic therapy requires an adequate blood supply. After administration of doxorubicin, mice bearing human tumour cell lines consistently show a sustained reduction in microregional blood flow for up to 60 minutes. Similar results in human tumours might compromise the delivery of multi-agent drug regimens. The use of neoadjuvant chemotherapy provides a useful in vivo model for studying the effects of anthracyclines on microregional blood flow in primary breast carcinoma.

**Aim** To investigate the effect of anthracycline administration on microregional human tumour blood flow using laser Doppler flowmetry.

**Methods** Microregional blood flow was measured in 24 consecutive females receiving primary medical therapy for biopsy proven invasive breast cancer. TNM staging T2–4, N0-1, M0. Age range 33–78 years. Fifteen patients received FEC (5-fluorouracil 600 mg/m2, epirubicin 60 mg/m2 and cyclophosphamide 600 mg/m2) and eight patients MM (methotrexate 35 mg/m2 and mitoxantrone 11 mg/m2). Up to 6 laser Doppler probes were inserted into the primary tumour. Blood flow was recorded for 10 minutes pre and up to 60 minutes post epirubicin or mitoxantrone administration prior to the administration of other drugs.

**Results** Results are available from 23 patients. Overall no change in tumour microregional perfusion was seen after anthracycline administration. However marked intra – and inter-tumour heterogeneity in blood flow was noted.

**Conclusions** Microregional perfusion has been measured in the context of primary medical therapy for breast cancer. Anthracyclines do not appear to reduce human tumour blood flow and should not therefore compromise the delivery of multi-agent systemic therapy.

### P62 PPROGNOSTIC VALUE OF ESTROGEN-PROGESTRONE RECEPTORS (ER, PR), EGFR and HER-2 IN BREAST CANCER,

A Ghaderi[1], B Gharesi-Fard[1], A Talei[1], M Vasei[1], H Modjtahedi[2], Dept. Immunology, Pathology and Surgery, Shiraz University of Med. Sciences. Shiraz-Iran, [2]Cancer Study Group, University of Surrey, Guildford GU2 7XH UK

Overexpression of the members of the type-I growth factor receptor family, in particular EGFR and HER-2/*neu*, has been reported in a number of epithelial malignancies and this in turn has been associated with a poor prognosis in many such patients. The anti-HER-2 monoclonal antibody trastuzumab (Herceptin®) has recently been approved for the treatment of HER-2 overexpressing metastatic breast cancer.

In the present study, the prognostic significance of estrogen receptor (ER), progesterone receptor (PR), and two members of the epidermal growth factor receptor (EGFR) family namely, EGFR (c-*erb*B-1) and HER-2/*neu* (c-*erb*B-2), were investigated in a series of breast tumor by comparing with the pathological data and Nottingham index. Indirect Immunoperoxidase method was used for determination of these receptors on formalin fixed paraffin-embedded sections of 112 breast carcinoma tissues from Iranian patients in Shiraz.

Results indicated that 51 (45.5%), 25 (22.3%), 51 (45.5%), 55 (49.1%), of tumors showed positive immunoreactivity with anti EGFR, HER-2, ER, or PR monoclonal antibodies respectively. Strong correlation between HER-2 staining and size ($P < 0.002$), histological grade ($P < 0.02$) and Nottingham index of tumors ($P < 0.007$) was observed but no significance association was found with the EGFR. In cases of ER, PR, strong relationship between lack of expression of these two receptors and size ($P < 0.008$, $P < 0.04$), histological grade ($P < 0.0005$, $P < 0.008$) and Nottingham index of tumors ($P < 0.04$, $P < 0.05$) was observed.

Strong correlation between HER-2 staining and negative staining for ER ($P < 0.03$), suggested that there is a reverse correlation between HER-2 and ER expression in patients with breast cancer in Iran.

### P63 HEAT SHOCK PROTEIN 27 PREDICTS SURVIVAL IN EARLY BREAST CANCER

Penny O'Neill[1], Abeer Shabaan[2], Andrew Dodson[2], Christopher S Foster[2], [1]Clatterbridge Centre for Oncology, Bebington, Wirral, CH 63 4JY, [2]Dept. Of Pathology, University of Liverpool, Merseyside, L69 3GA, UK

Heat shock proteins (hsps) occupy a central role in the regulation of intracellular homeostasis and have been shown to correlate with aggressive biological behaviour in prostate cancer. In previous studies hsp27 has shown conflicting associations with outcome in breast cancer.

Eighty-seven cases of primary invasive breast cancer diagnosed 1993/94 were retrieved from the archives of The Royal Liverpool University Hospital along with 40 cases of normal breast tissue for use as controls. All cases underwent histopathological review. The majority (80/87) of cases were invasive ductal carcinoma, 5 were invasive lobular, 1 tubular and 1 medullary. 8% were grade 1, 46% grade 2 and 46% grade 3. Chemotherapy was given to 19% cases and 56% received adjuvant hormone treatment. There have been 38 relapses and 27 deaths. Immunohistochemistry for hsp27 was performed using heat treatment antigen retrieval and a murine monoclonal antibody (Novocastra Laboratories Ltd). Positive staining was cytoplasmic and quantified by measuring the mean optical density (OD) and mean area of staining for each case using a morphometric image analysis system. hsp27 mean OD was quantified using 5 random fields per case. In addition the % of positive stained cells was estimated manually. In normal breast the expression was significantly lower than the expression in the malignant cases ($P < 0.001$). In cancers, higher hsp27 expression was significantly associated with positive ER status ($P < 0.001$) and ER% ($r = 0.44$, $P = 0.01$). Those patients who had died showed a significantly lower expression of hsp27 ($P = 0.04$). In normal breast the mean OD was 0.34 whereas in cancers it was 0.57. In cancers a higher hsp27 mean OD was significantly associated with hsp 27% ($r = 0.63$, $P = 0.01$), ER% ($r = 0.28$, $P = 0.01$), and positive ER status ($P < 0.001$). Those patients still alive had a significantly higher hsp27 mean OD compared with those who had died ($P = 0.004$).

In this cohort of primary breast cancer patients hsp27 expression was associated with a better outcome. In addition we have shown that quantitative analysis of positive staining using a morphometric image analysis system is feasible and provides objective data that are more strongly associated with outcome than manual interpretation.

### P64 FUNCTIONAL ACTIVITY OF ABC-TRANSPOR-TERS CAN BE AN ADDITIONAL PROGNOSTIC FACTOR OF HORMONE THERAPY EFFICACY IN BREAST CANCER PATIENTS

E Koldaeva, T Bogush, E Bogush, G Tchemeris, N Kushlinsky, E Gerstain, G Smirnova, I Shubina, Russian Blokhin Cancer Research Center, Moscow, Russia

Estrogen receptors (ER) are determinants of sensitivity to antiestrogen hormone therapy of breast cancer but the treatment is not effective enough in all the patients (pts) with ER-positive tumor status. We have supposed that among the various reasons of this situation activity of ABC-transporter(s)' can be an important one because antiestrogens can inhibit transporter activity binding to them and thereby decreasing an active (free) intracellular concentration of antiestrogens bound to ER and as a result – hormone therapy efficacy. The aim of the study was to evaluate ABC-transporters' functional activity in ER-positive primary breast cancer. Activity of both P-gp and other ABC-transporter(s) was assayed in 59 pts by a new methodology described by us previously using two transporter inhibitors – verapamil (for P-gp) and sodium azide (for all energy-dependent transporters). RESULTS. 1. No expression of both P-gp and non-P-gp transporter(s)' activity was shown in 38% of the ER-positive tumors. We believe that according to the index investigated these pts can have the best prognosis in terms of successful anticancer hormone therapy. 2. Expression of ABC-transporter(s)' functional activity (both P-gp and non-P-gp or non-P-gp only) was shown in about 62% of the ER-positive tumors. We believe that these pts (especially with tumor expression of both P-gp and non-P-gp transporters) can have worse prognosis in terms of successful anticancer' hormone therapy as compared to those with no tumor ABC-transporters' activity. We believe also that influence of expression of both types of ABC-transporters' functional activity (both P-gp and non-P-gp transporters) on antiestrogen therapy of breast cancer with low level of ER has to be the most pronounced. Clinical confirmation of the conclusion is under way.

### P65 CLINICAL, RADIOLOGICAL AND PATHOLOGICAL ASSESSMENT OF RESPONSE TO NEOADJUVANT CHEMOTHERAPY IN PATIENTS WITH LOCALLY ADVANCED BREAST CANCER

A Srivastava[1], NK Bosu[1], O Coshic[1], A Kumar[1], R Dawar[2], S Bandhu[3], A Goyal[1*], [1]Dept. of Surgery, [2]Dept of Pathology, [3]Dept of Radiology, All India Institute of Medical Sciences, New Delhi-110029, India

Clinical assessment is inaccurate for assessment of chemotherapeutic response. Mammography and ultrasonography of the breast may provide useful additional information. Histological assessment however remains the gold standard.

**Objectives** To evaluate the association between clinical, radiological (mammographic and ultrasonographic) and pathological response after neo-adjuvant chemotherapy in patients with locally advanced breast cancer.

**Material and Methods** Forty one patients of cytologically/histopathologically proven locally advanced breast cancer(stageIII) were studied for assessment of response of neoadjuvant chemotherapy. All patients received three cycles of neoadjuvant chemotherapy (CAF/CMF) at three weeks interval. Clinical, mammographic, ultrasonographic examination was done before starting the chemotherapy and 3 weeks after the 3$^{rd}$ cycle of chemotherapy to assess the response to neoadjuvant chemotherapy. Twenty five patients were operated 3 weeks after the 3$^{rd}$ cycle of neoadjuvant chemotherapy. Macroscopic and microscopic pathology review was used to assess the degree of tumor reduction. Clinical and radiological response was compared with the gold standard i.e-histological response.

**Results** Clinically 17(41.5%) patients had complete response(CR), 22(53.7%) patients had partial response(PR) and 2(4.9%) patients had no response(NR) or stable disease. Diagnostic indices compared to histological finding were-sensitivity = 100%, specificity = 55%, positive predictive value (PPV) = 35.7%, negative predictive value (NPV) = 55%, positive predictive value (PPV) = 35.7%, negative predictive value(NPV) = 100%. Mammographic examination was done for 34 patients(7 were lost to follow up). Six (17.6%) patients had CR, 25(73.5%) patients had PR and 3(8.8%) patients had NR or stable disease. Diagnostic indices compared to histological finding were-senstivity = 80%, specificity = 90%, PPV = 66.6%, NPV = 94.7%. Ultrasonographic examination was done for 33 patients (8 were lost to follow up). Six (18.2%) patients had CR, 23(69.7%) patients had PR and 4(12.1%) patients had NR or stable disease. Diagnostic indices compared to histological finding were-sensitivity = 60%, specificity = 90%, PPV = 60%, NPV = 90%. 14 of the 25 patients operated were clinically complete responders, 10 were clinical partial responders and 1 was non responder. In most cases the pathological response of chemotherapy correlated with clinical response. The greatest discordance was found in patients with clinical complete response. Of those operated 18(72%) patients had macroscopic and

**P65 Cont'd**

microscopic residual tumor. Seven patients had no macroscopic tumor. Of these five had no microscopic evidence of disease while 2 had microscopic residual tumor.

**Conclusions** Clinical assessment alone is inaccurate for assessment of chemotherapeutic response. The accurate assessment of tumor response to neoadjuvant chemotherapy in locally advanced breast cancer should be a reflection of clinical examination, mammographic and ultrasonographic evaluation and finally histopathological evaluation.

### P66 A COMBINATION OF VINORELBINE AND CARBOPLATIN AS FIRST LINE TREATMENT FOR PATIENTS WITH METASTATIC BREAST CANCER – A PHASE II STUDY.

AS Protheroe, S Rodwell, H Aram, D Dodwell, M Crawford, C Bradley, TJ Perren, S Hill, JK Joffe[1], On behalf of the Yorkshire Breast Cancer Research Group, [1]Huddersfield Royal Infirmary, Huddersfield, ICRF Cancer Medicine Research Unit, St James's University Hospital, Leeds UK LS9 7TF, UK

The combination of vinorelbine and carboplatin has been investigated as a first line treatment in the setting of metastatic breast cancer, within the Yorkshire region. The aim of the study is to identify the activity of this new regimen with potential for non-cross resistance with anthracycline and taxane containing treatments. The scheduling of this combination comprises day one and day eight vinorelbine 25 mg/m$^2$ and day one carboplatin AUC5, repeated on a three weekly cycle. Between October 1998 and February 2001 29 patients have been enrolled on this study from five centres within Yorkshire. Further patients continue to be entered. Of the 29 patients enrolled 23 have received 3 or more cycles of treatment, 2 of these are still ongoing and 21 are evaluable for response. 27 patients are evaluable for toxicity. One patient died from neutropenic sepsis and 7 (26%) patients withdrew because of toxicity. 19 out of 27 (70%) patients experienced a grade 3/4 neutropenia, 10/27 (37%) developed a grade 3/4 neutropenic infection. 2/27 (7%) developed grade 3 anaemia, 2/27 (7%) grade 3 thrombocytopenia, 2/27 (7%) grade 3 nausea, 1/27 (4%) grade 3 vomiting, 1/27 (4%) grade 3 diarrhoea and 1/27 (4%) grade 3 hyponatraemia. 11 out of 21 (52.4%) patients have achieved a partial response, 5/21 (23.8%) stable disease and 5/21 (23.8%) have had progressive disease. Data will be presented on response rates and progression free survival with up dated information on toxicity and tolerability.

### P67 *TANGO* – A PHASE III, RANDOMISED TRIAL OF GEMCITABINE IN PACLITAXEL-CONTAINING, EPIRUBICIN-BASED, ADJUVANT CHEMOTHERAPY FOR HIGHER RISK, EARLY STAGE, BREAST CANCER

CJ Poole[1], J Carmichael, RE Coleman, HM Earl, D Dodwell, M Verrill, RCF Leonard, J Mansi, D Cameron, C Twelves, P Canney, JA Dunn[1], HC Howard[1], S Bathers[1], [1]CRC Trials Unit, Institute for Cancer Studies, University of Birmingham, B15 2TT, UK

Despite recent advances, many women still succumb to the metastatic complications of breast cancer and more effective adjuvant treatment is required. The 1998 Oxford Overview confirmed that combination chemotherapy provides significantly improved relapse-free (RFS) and overall survival (OS), and that the substitution of anthracyclines confers further modest advantage. More recently, the 3000-patient CALGB 9344 trial has shown that the sequential addition of four cycles of paclitaxel to standard therapy with four cycles of AC offers additional survival advantage (HR = 0.86. However, retrospective subgroup analysis suggests that the improvement in RFS (901 events) is limited to the 1055 ER-negative patients randomised (HR = 0.75; $P = 0.02$). A similar trend is seen in the less mature (551 events) NSABP-B28 study (HR = 0.75; n.s.).

Since the Overview itself shows little effect from single agent adjuvant therapy, it seems plausible that consolidation with a paclitaxel-based combination may be superior to paclitaxel alone. Arguably, gemcitabine seems the best candidate for incorporation into such a regimen; not only does its use avoid the more difficult non-myelosuppressive toxicity exhibited by other agents such as capecitabine or vinorelbine in combination with paclitaxel, but three trials show encouraging activity for the gemcitabine-paclitaxel combination in advanced breast cancer, particularly in respect of CR rates (16–21%).

The aim of the *tAnGo* trial is to examine whether the addition of gemcitabine (1250 mg/m$^2$ days 1 and 8, q 3 weeks) to paclitaxel (175 mg/m$^2$/3 hours, day 1, q 3 weeks) following four cycles of epirubicin (90 mg/m$^2$ day 1, q 3 weeks) and cyclophosphamide (600 mg/m$^2$ day 1, q 3 weeks) improves RFS in relation to epirubicin and cyclophosphamide followed by paclitaxel alone. At present, the available data from CALGB 9344 and NSABP-B28 would perhaps support this approach most strongly in ER-negative and/or PGR-negative patients.

The primary endpoint of the study is RFS at 5 years. RFS at 10 years and OS at 5 and 10 years will be addressed as secondary endpoints. A safety study of pulmonary and cardiac function will involve 130 patients and provide a sensitive measure of any sub-clinical problems associated with the gemcitabine/paclitaxel/radiotherapy combination. Quality of life data will also be collected from a subset of 500 women. The accrual target is 3000 patients recruited within 3 to 4 years from centres within the UK, North America, and continental Europe. There are three planned interim analyses, the first at 440 events, the second at 800, and the final analysis after 1570. Log-rank analysis on intention to treat will be carried out on all causes of mortality and relapse.

### P68 EVALUATION OF CLINICAL COMPLETE RESPONSE TO NEOADJUVANT CHEMOTHERAPY IN PRIMARY BREAST CANCER USING ($^{18}$F)-FLUORODEOXYGLUCOSE POSITRON EMISSION TOMOGRAPHY

RJ Burcombe[1], A Makris[1], M Pittam[1], J Lowe[2], J Emett[2], W L Wong[2], [1]Marie Curie Research Wing, [2]The Paul Strickland Scanner Centre, Mount Vernon Hospital, Northwood, Middlesex HA6 2RN, UK

**Aim** To determine whether ($^{18}$F)-fluorodeoxyglucose (FDG) positron emission tomography (PET) can predict complete pathological response (pCR) in patients achieving a good clinical response to neoadjuvant chemotherapy for primary breast cancer.

**Patients and methods** Ten patients who achieved a good clinical response – complete clinical response (cCR) or minimal residual disease (MRD) – to six cycles of neoadjuvant FEC chemotherapy (5 fluorouracil 600 mg/m$^2$) epirubucin 60 mg/m$^2$ and cyclophosphamide 600 mg/m$^2$ underwent FDG PET scanning prior to definitive breast surgery. PET scan reports were correlated with subsequent histopathological findings.

**Results** No FDG uptake was visualised at the primary tumour site on PET scan in any patient. Nine patients had residual invasive carcinoma at operation, ranging from 2–20 mm in maximum dimension. Histology is awaited in one case. Of the five patients who underwent axillary surgery, no axillary FDG uptake was seen pre-operatively although 3 of the 5 were node positive at operation.

**Conclusions** FDG PET scans failed to detect residual invasive carcinoma pre-operatively at both the primary site and involved axillary lymph nodes in this series of good clinical responders. Consequently, its sensitivity as a tool to identify complete pathological response to neoadjuvant chemotherapy is inadequate for clinical use.

### P69 EVALUATION OF ER, PgR, HER2 AND Ki67 AS PREDICTORS OF RESPONSE TO NEOADJUVANT CHEMOTHERAPY FOR OPERABLE BREAST CANCER

RJ Burcombe[1], A Makris[1], PI Richman[1], M Pittam[1], F Daley[2], S Noble[2], GD Wilson[2], [1]Marie Curie Research Wing, [2]The Gray Laboratory, Mount Vernon Hospital, Northwood, Middlesex, HA6 2RN, UK

The increasing use of neoadjuvant chemotherapy for operable breast cancer provides an opportunity to identify biological markers that predict clinical response to treatment.

96 women (mean age 57 years, range 27–78) received 6 cycles of FEC chemotherapy (5-Fluouracil 600 mg/m$^2$, Epirubicin 60 mg/m$^2$, Cyclophosphamide 600 mg/m$^2$) prior to surgery and / or radical radiotherapy. Tamoxifen was commenced post-operatively in patients with ER positive tumours. Clinical response was assessed using conventional UICC criteria and categorised as ‘responders’ (complete or partial response) or ‘non-responders’ (stable or progressive disease). Immunohistochemical analysis was performed on all diagnostic core biopsies and 50 subsequent surgical specimens using monoclonal mouse antibodies for anti-HER2 (Zymed laboratories), ER and PgR (Novocastra) and Ki67 (Dako).

80% of patients were clinical responders, including 23 (24%) achieving complete clinical response. 68% of core biopsies were ER+, 60% PgR + and 15% HER2+. Ki67 proliferation index ranged from 1.5–58.5% (median 24.4%). Clinical response rates by marker status and Ki67 index (above and below median) are shown below:

| | ER+ | ER– | PgR+ | PgR– | HER2+ | HER2– | Low Ki67 | High Ki67 |
|---|---|---|---|---|---|---|---|---|
| Responders (%) | 82 | 77 | 84 | 74 | 64 | 82 | 83 | 76 |

Neither ER nor PgR status at diagnosis predicted clinical response. There was a trend towards poor response in HER2+ patients ($P = 0.11$). All HER2 overexpressors remained HER2+ after neoadjuvant chemotherapy. A reduction in Ki67 index before and after treatment was seen (median change –37%) but no significant difference was found between responders (median change –41%) and non-responders (–14%).

### P70 A PHASE II PILOT STUDY OF VINORELBINE AND CISPLATIN IN PATIENTS WITH METASTATIC BREAST CANCER (MBC),

J Abraham, K McAdam[1,2], C B Wilson[1], H M Earl[1], [1]Addenbrookes Hospital NHS Trust, Cambridge CB2 2QQ, [2]Peterborough District Hospital NHS Trust

**Purpose** Vinorelbine is a novel vinca alkaloid which has shown excellent response rates (RR) in patients with MBC – 40–60% first-line and 60–70% second-line in combination with anthracyclines, paclitaxel and 5fluorouracil. In addition there appears to be a lack of cross-resistance with anthracyclines. There have been few previous phase II studies using vinorelbine in combination with cisplatin involving a small number of patients (17–26) with sparse quality of life (QoL) data. RR were reported between 60–70% and median progression free survival (PFS) of 7.3 months (11.3 months for responders). The main dose-limiting toxicity was myelosuppression (10% CTC grade II/IV) with 5–43% grade (G) II/III nausea/vomiting and 11% grade II neuropathy. The aim of this study is to evaluate the activity and feasability of this combination in patients with MBC undergoing second or third-line therapy. Primary endpoints are PFS and RR and secondary endpoints are QoL and toxicity.

**Methods** All patients had MBC and had received at least one previous chemotherapy regimen for metastatic disease. WHO performance status was 0–2, with a treatment-free interval of > 3 months and a life expectancy of > 3 months with adequate renal, liver and haematological function. Patients were given Vinorelbine 25 mg/m$^2$ on day 1/day 8 and Cisplatin 80 mg/m$^2$ on day 1, repeated every 21 days up to a maximum of 6 cycles. Toxicity, QoL, renal, liver and hasematological function were assessed prior to each cycle and disease assessment was made after the 3rd and 6th cycle.

**Results** 8 patients have been recruited to date (mean age 55 years). 5 patients had previously received an anthracycline and 2 patients a taxane. The majority of patients (5) had only received one chemotherapy regimen previously and 1 patient had had 3 previous combinations. 4 patients had disease at a single site, 3 at 2 sites and 1 at 3 sites and these included liver (1 patient), lung (4), bone (2), nodal (3) and soft tissue (2). 3 patients received all 6 cycles, 1 patient each received 5, 4 and 1 cycle and 2 patients received 3 cycles. There were dose reductions in 4 patients (2 -neutropenia and 2 ototoxicity). Toxicity was generally mild: Nausea G I/II – 44% and G III 2%; neutropenia G1/II 11% and G III/IV 11%, constipation G I/II 14%, ototoxicity GIII 2%, nephrotoxicity G I 2% and neuropathy G I/II 14%. RR was 50% (1 CR, 3PR, 1SD and 3PD). The mean duration of response was 2.5 months (1–5 months).

**Conclusion** Vinorelbine and Cisplatin is an active combination in patients with MBC even in those pre-treated with anthracyclines. Toxicity was generally mild. This trial is still actively recruiting and full results together with the quality of life evaluation will be available later this year.

### P71 PROTEIN TYROSINE PHOSPHATASES DOWN-REGULATED IN MELANOMA

L McArdle[1], M Rafferty[1], O Bergin[1], PA Dervan[2], D Easty[1], [1]Pathology Department, University College Dublin, [2]Mater Misercordiae Hospital, Dublin

Protein-tyrosyl phosphorylation is a key control mechanism for growth and differentiation. Phosphotyrosine levels are controlled by the co-ordinated actions of protein tyrosine kinases (PTKs) and protein tyrosine phosphatases (PTPs). A number of PTKs are encoded by proto-oncogenes or viral oncogenes, and are implicated in carcinogenesis. Excessive expression of PTKs can contribute to melanoma formation; previously we found over-expression of PTKs in late stage melanoma (Easty *et al*., 1995). It was hypothesized but remains unproven that PTPs function as tumour suppressors. Here we survey PTP expression in pigment cells. PTP genes were cloned from a melanoma cell line and normal human melanocytes by RT-PCR, using degenerate primers based upon conserved sequences within the catalytic domain of published PTPs. Northern blotting was used to test for quantitative differences in PTP expression in normal and malignant cells. Fifteen genes were tested and two sequences, PTP-KAPPA and PTP-PI were selected for further study. Expression of these genes was decreased or lost in melanoma cell lines compared to normal melanocytes, and in some unmanipulated melanoma biopsies. This was confirmed at the protein level for PTP-KAPPA in lysates from unmanipulated melanoma biopsies. PTP-KAPPA was detected in compound nevi. Interestingly, both genes are members of the type 2 receptor PTPs and their structures suggest a role in cell adhesion. PTP-KAPPA is upregulated by TGFβ (a negative regulator of cell growth) and has been implicated in contact inhibition. Loss of PTP expression may contribute to the abnormal tyrosine phosphorylation seen in melanoma; these genes are candidate tumour suppressors.

Easty *et al*., *Cancer Research*, **55**, 2528–2532, (1995)

Sponsored by the Health Research Board

### P72 17β ESTRADIOL AND THE FEMALE SURVIVAL ADVANTAGE IN MELANOMA

R Molife[1,2], C Layton[2], P Lorigan[1], S MacNeil[2], [1]Dept of Oncology, Weston Park Hospital, Sheffield, S10 2SJ, UK, [2]University Section of Medicine, Northern General Hospital, S5 7AU, UK

Epidemiological data shows that female patients with primary melanoma have a significant survival advantage over males (1); this female survival benefit can also be demonstrated in immuno-deficient animals (2). In investigating a possible mechanism for this benefit, we have previously reported that the female steroid 17β estradiol significantly reduces invasion of a human melanoma cell line (A375SM) through fibronectin in vitro (3). Our aim was to extend this work into a preclinical model of melanoma using a reconstructed human skin model which allows the investigation of melanoma/cell/extracellular matrix (ECM) interactions. Three human cutaneous cell lines A375SM, HBL and C8161 were compared in this model. Human skin was sterilised and made acellular to form de-epidermalised dermis (DED) with retained basement membrane antigens. Dermal fibroblasts (Fib), epidermal keratinocytes (K) and human melanoma cells were added to the DED. The resultant composites were cultured in 17β estradiol ($10^{-11}$ to $10^{-7}$) at an air-liquid interface for 14–18 days prior to histology and immunohistochemistry. A quantitative blind scoring system was used to assess the effect of 17β estradiol on skin morphology and melanoma invasion.

All three melanoma lines showed marked invasion into the dermis in this model. 17β estradiol slightly reduced the invasion of HBL cells ($P > 0.05$) and significantly reduced the invasion of A375SM ($P < 0.005$ at $10^{-9}$) and C8161 cells ($P < 0.005$ at $10^{-8}$) (n = 3). In contrast, estradiol had no significant effect on the morphology of the epithelial layer in the absence of melanoma cells ($n = 6$). The introduction of melanoma cells in general had relatively little effect on the morphology of the epidermal layer until steroids were added. For one cell type (A375SM) estradiol improved the quality of the K layer whilst for HBLs, estradiol decreased the quality; there was no effect when C8161 cells were present. Thus, estradiol was consistent in reducing invasiveness of melanoma but did not appear to directly affect the epidermal layer.

These preliminary findings confirm that this model allows one to ask whether 17β estradiol may act directly on melanoma cells or indirectly via effects on the epidermal keratinocytes (which may act as a barrier to melanoma invasion). The simplest interpretation of our current data in this model is that it is the melanoma cells, rather than the keratinocytes, which respond directly to steroids with a modest reduction in their invasion.

1. Miller JG and MacNeil S, 1997 Br J Derm 136:657
2. Ladanyi A, Timar J, Bocsi J *et al*. 1995 *Melanoma Res* **5**:83
3. Richardson B, Price A, Wagner M *et al*. 1999 *Br J Canc* **80 (**12**)**: 2025

### P73 A PHASE I STUDY OF TEMOZOLOMIDE AND CARBOPLATIN IN PATIENTS WITH METASTATIC MELANOMA

SJ Strauss, M Marples, T Meyer, M Napier, J Boxall, GJS Rustin, Dept of Medical Oncology, Mount Vernon Hospital, Rickmansworth Road, Northwood, Middlesex HA6 2RN, UK

**Purpose** Temozolomide is an oral alkylating agent with equivalent response rates to DTIC in the treatment of metastatic melanoma, and carboplatin has a reported response rate of 16%. Both drugs have favourable toxicity profiles and can be administered as an outpatient. We conducted a Phase I trial to establish the maximum tolerated dose (MTD) of this combination.

**Patients and Methods** Thirty patients with metastatic melanoma were treated with escalating doses of both drugs. In phase I, patients were treated with temozolomide at 750 mg/m$^2$ divided over 5 days, and carboplatin administered on day 1. Cycles were repeated 4 weekly for a maximum of 6 cycles. Patients were treated in 4 cohorts of 3 at carboplatin doses escalating from AUC 3 to AUC 4, AUC 5 and AUC 6. In phase II the dose of temozolomide was increased to 1000 mg/m$^2$ and escalation of carboplatin dose repeated.

**Results** In Phase I, 12 patients, aged 39–67 years (median 55) received 35 cycles of treatment. Toxicity was primarily haematological (Table). There was one dose delay due to neutropenia. Nine patients progressed on treatment and 3 had stable disease. In phase II, 18 patients aged 40–81 years (median 55) received 49 cycles of treatment. There were 8 delays, 4 for thrombocytopenia, and 4 for neutropenia. There were 2 dose reductions for thrombocytopenia. The dose limiting toxicity was haematological with 2 patients experiencing grade III/IV neutropenia and 1 patient grade IV thrombocytopenia at a carboplatin dose of AUC 5. The MTD was carboplatin AUC 4. In both phases, only 4 patients had ≥ grade 2 nausea. Two patients achieved partial responses, 4 had stable disease, and 12 progressed on treatment.

No. Patients with Haematological Toxicity ≥ Grade 2 / No. Patients Treated

| **Temozolomide dose** | **Carboplatin dose** | | | |
|---|---|---|---|---|
| | AUC 3 | AUC 4 | AUC 5 | AUC 6 |
| *750 mg/m$^2$ | 1/3 | 1/3 | 1/3 | 1/3 |
| 100 mg/m$^2$ | 2/3 | 5/11 | 2/4 | – |

*No patients experienced grade III/IV toxicity

**Conclusion** The combination of carboplatin and temozolomide is well tolerated with acceptable toxicity at doses of temozolomide 1000 mg/m$^2$ and carboplatin AUC4. Preliminary response information is encouraging and further phase II trials should be considered.

### P74 A PHASE I/II STUDY OF TREOSULFAN(T) AND DACARBAZINE (D)IN CHEMONAIVE PATIENTS WITH MALIGNANT MELANOMA

CV Eswar[1], T Murdoch[1], M Sheddon[2], A McMurray[2], E Marshall[1], Clatterbridge Centre for Oncology, Bebington, Wirral, L63 4JY, [2]Medac, Glasgow, G3 6EQ, UK

Treosulphan (T) is a bifunctional alkylating agent, which has recently been reported to have activity in melanoma both in vitro against choroidal melanoma (Neale et al, BJC 1999) and chemoresistant melanoma cell lines. In vivo at relapse it has also shown activity in metastatic melanoma (MM) patients (Neuber et al, Melan Res 1999). Furthermore T appears to have enhanced in vitro activity when used in combination (Myatt et al, Anticancer Drugs 1997). In view of these findings, we have embarked on a phase I/II study on T in combination with DTIC (D) in chemotherapy naive patients with MM. D was administered at a set dose (850 mg/m$^2$) at 21 day intervals upto a maximum of 6 cycles. In addition patients received a short IV infusion of T immediately preceding D at one of 4 dose levels (4 gm/m$^2$, 6 gm/m$^2$, 7 gm/m$^2$, 8 gm/m$^2$) with a 3 day granisetron and dexamethasone antiemetic regimen. Eligibility criteria included; ECOG performance status ≤2, no prior chemotherapy (excluding limb perfusion and immunotherapy), anticipated survival >3 months, adequate hepatic/renal function and normal haemotological indices. Exclusion criteria were symptomatic CNS metastases. To date 15 patients (4 ocular, 11 cutaneous) have received 49 cycles. Cohorts 1&2 have completed treatment and 3&4 are continuing chemotherapy. So far the combination has been very well tolerated with mild non haemotological toxicity consisting of grade (G) 1–2 lethargy, G 1–2 nausea and vomiting, G 1 alopecia & G1 skin rashes. 3 patients developed G3 thrombocytopenia & G 3 neutropenia (one each in cohorts 1/2/3). There has been cumulative thrombocytopenia and neutropenia over successive cycles of chemotherapy, but there has been no dose relationship. Out of 10 patients who have completed treatment 3 have stable disease (SD)and seven patients had progressive disease (PD) of which 4 had PD in the CNS. Recruitment continues for the final cohort, which has now been expanded to determine the response rate to the combination.

### P75 PREDICTING PROGRESSION AND SURVIVAL IN BLADDER CANCER: USE OF P27$^{K1P1}$ (P27) AND EPIDERMAL GROWTH FACTOR RECEPTOR (EGFR)

A Valentine, JL Ritchie, GB Nevin, SR McKeown, School of Biomedical Sciences, University of Ulster, Jordanstown, N. Ireland

At initial presentation, 70% of bladder tumours are superficial while the remaining 30% are invasive. A minority of patients (around 30%) who present with superficial disease remain disease-free for life after tumour removal. The majority will have multiple recurrences, at some time in their lifetime and 10–20% of these will become invasive. The identification of prognostic markers which could predict disease progression, as early as possible, is vital and would allow for the establishment of appropriate treatment regimens. Absent or decreased expression of the cyclin dependent kinase inhibitor, p27$^{kip1}$ (p27) and over-expression of Epidermal Growth Factor Receptor (EGFR) have been found in many tumour types including breast, lung and prostate. The purpose of this study was to determine whether p27 and EGFR could be used individually, or in combination, as reliable prognostic markers in bladder cancer. Immunohistochemistry was used to assess bladder tumours for p27 (58 samples), nuclear EGFR (69 samples) and 53 biopsies for both markers. To assess p27 status, samples were divided into p27 negative (< 30%) and p27 positive (> 30%). P27 status in bladder tumours was associated with tumour stage ($p < 0.0001$), disease recurrence ($P = 0.0448$) and disease progression ($P = 0.0020$). Patients with p27 positive tumours had a longer disease-free survival, progression-free survival and overall survival than those patients with p27 negative tumours (log-rank analysis: $P = 0.0015$, 0.0006 and 0.0283 respectively). To assess nuclear EGFR status, samples were divided into EGFR positive (> 5%) and EGFR negative (< 5%). Nuclear EGFR expression was associated with disease progression ($P = 0.0044$) and progression-free survival (log-rank analysis: $P = 0.0129$). When p27 and nuclear EGFR expression were combined, a superior prognostic indicator to either p27 or nuclear EGFR status alone was created ($P = 0.0002$).

### P76 A PHASE II TRIAL OF CISPLATIN, METHOTREXATE AND VINBLASTINE CHEMOTHERAPY FOR PURE SQUAMOUS CELL CARCINOMA OF THE URINARY TRAC

JM Russell, GO Griffiths, BM Uscinska, RA Cowan, KM Grigor on behalf of the MRC Bladder Cancer Group, MRC Clinical Trials Unit, 222 Euston Road, London, UK

A UK multi-centre phase II trial was carried out to make an assessment of the response rate to cisplatin, methotrexate and vinblastine (CMV) chemotherapy in patients with pure squamous cell carcinoma of the urinary tract. Eligible patients had initial presentation with T3-T4 disease, pelvic relapse after radiotherapy or surgery, or nodal or metastatic disease, with at least one site of disease assessable for response. All patients were to receive 3 cycles of CMV over 3 weeks (30 mg/m$^2$ methotrexate and 4 mg/m$^2$ vinblastine on days 1 and 8, 100 mg/m$^2$ cisplatin on day 2 and 15 mg 6 hr × 4 folinic acid on days 2 and 9). The primary endpoint was response assessed 9 weeks after commencement of treatment, the secondary endpoint was survival. From October 1993 to February 1999 a total of 38 patients were entered by 13 centres. 55% of patients were male, 58% less than 60 years old and 76% WHO performance status 0/1, 58% of patients (n = 22) received all 3 cycles. The review pathologist reported 76% (n = 29) to be pure squamous cell carcinoma, 16% (n = 6) to be transitional cell carcinoma with squamous differentiation and 8% (n = 3) to be other. Using all 38 patients at 9 weeks 13% (n = 5) had a CR, 26% (n =10) a PR, 24% (n = 9) SD, 16% (n = 6) progression and 21% (n = 8) were not assessable. Using the 29 patients with pure squamous cell carcinoma only, at 9 weeks 14% (n = 4) had a CR, 28% (n = 8) a PR, 24% (n = 7) SD, 14% (n = 4) progression and 21% (n = 6) were not assessable. A total of 29 patients have died; 76% (n=22) were due to squamous cell carcinoma disease, 3% (n = 1) was treatment related and 21% (n = 6) were due to other reasons. CMV chemotherapy is active in squamous cell carcinoma of the bladder, of the same order as in transitional cell carcinoma.

### P77 A PHASE II TRIAL OF CONTINUOUS 5-FLUOROURACIL (5-FU) IN RECURRENT LOCALLY ADVANCED OR METASTATIC TRANSITIONAL CELL CARCINOMA OF THE URINARY TRACT

M Highley, G Griffiths, B Uscinska, P Harper, R Huddart J Barber on behalf of the MRC Bladder Cancer Working Party, MRC Clinical Trials Unit, 222 Euston Road, London NW1 2DA, UK

A UK multi-centre phase II trial was carried out to make an assessment of the response rate at 8, 16 and 24 weeks and toxicity to continuous infusion of 5-FU in patients with recurrent locally advanced or metastatic transitional cell carcinoma of the urinary tract. Eligible patients were those with either pelvic relapse after radiotherapy or surgery, or nodal or metastatic disease. Patients were not to have received previous systemic chemotherapy. All patients were to receive 24 weeks of continuously infused 5-FU at a dose of 300 mg/m$^2$/day via a Hickman line. A total of 50 patients were entered by 8 centres, 64% of patients were over 65 years old, 70% were male and 78% had a WHO performance status of 0/1. We currently have data on response at 8 weeks for 43 of the patients; none had a complete response, 16% (n = 7) had a partial response, 16% (n = 7) had stable disease, 53% (n = 23) progression and 14% (n = 6) were not assessable. A total of 34 patients have died, 88% (n = 30) of deaths were due to transitional cell carcinoma and 12% (n = 4) were due to other causes, there were no treatment related deaths. More detailed data, in particular response at 16 & 24 weeks, will be available for time of presentation.

### P78 ZD1839 ('IRESSA') INHIBITS THE GROWTH RESPONSE OF BLADDER TUMOUR CELL LINES TO EPIDERMAL GROWTH FACTOR AND THE INDUCTION OF TIMP1

JE Nutt[1], JKMellon[2], J Lunec[1], [1]Cancer Research Unit, [2]Dept of Surgery, University of Newcastle, Newcastle upon Tyne, NE2 4HH

The Epidermal Growth Factor Receptor (EGF-R) in bladder cancer is associated with high tumour stage and grade, and has been found to be a strong independent predictor of invasive tumour progression and poor long term survival. ZD1839 ('Iressa') is an orally active, selective EGF-R tyrosine kinase inhibitor which blocks signal transduction pathways implicated in cancer cell proliferation. The effect of both EGF stimulation and its inhibition with ZD1839 has been investigated in 2 EGF-R positive human bladder tumour cell lines, RT112 and RT4. Cells grown in serum free RPMI medium showed increased growth over 4 days when treated with 10 or 50 ng $ml^{-1}$ EGF as measured by the SulfurRhodamine B assay. 1μM ZD1839 alone had no effect on cell growth, but the rate was decreased with 5μM ZD1839. However, the proliferative effect of EGF stimulation in both cell lines was inhibited in the presence of 1μM ZD1839, demonstrating its effect on EGF-R signaling.

Matrix metalloproteinases (MMPs) and their inhibitors (TIMPs) are implicated in tumour invasion and metastasis. Conditioned medium from both cell lines treated with a combination of 10 ng $ml^{-1}$ EGF and 1 and 5μM ZD1839 for 48 h showed no change in MMP2 concentrations using zymograms. However, using Western blot analysis, TIMP1 was shown to increase in conditioned medium from RT112 cells treated with 50ng $ml^{-1}$ EGF. This was partially inhibited with both 1 and 5μM ZD1839, suggesting alterations in the balance of MMPs and their inhibitors in EGF stimulated cells, which may be important in the treatment of bladder tumours. The effects of ZD1839 on MMPs found in the urine of bladder cancer patients will be investigated.

'Iressa' is a trade mark of the AstraZeneca group of companies

### P79 META-ANALYSIS OF INTRAVESICAL THERAPY FOR SUPERFICIAL BLADDER CANCER: SUPERIORITY OF BACILLUS CALMETTE-GUERIN MAY BE CONFINED TO HIGH RISK PATIENTS

MD Shelley[1], J Court[1], K Burgon[1], B Coles[1], H Kynaston[2], T Wilt[3] and MD Mason[1], Cochrane Unit, Velindre Hospital, Cardiff CF14 2TL, [2] Dept. of Urology, UWCM, Heath Park, Cardiff CF4 4XW, [3] Dept. of Veterans Affairs, MN 55417, USA

**Background** Tumour recurrence following transurethral resection (TUR) for Ta and T1 bladder cancer is a major clinical problem. Intravesical administration of mitomycin C (MMC) or Bacillus Calmette-Guerin (BCG) has proven prophylactic activity but both are associated with local and systemic side effects. A systematic review and meta-analysis was carried out to compare the efficacy of these two agents.

**Methods** A comprehensive search of MEDLINE, CANCERLIT, EMBASE, Healthstar, BIDS, Cochrane Controlled Trials Register and DARE was performed, and hand searching of relevant journals undertaken. Trials in any language were included in the meta-analysis if they were properly randomised, included medium to high risk patients with Ta or T1 bladder cancer and compared intravesical MMC versus BCG. Time to event analyses of tumour recurrence with a sensitivity analysis for subgroups according to patients's risk of recurrence, were performed.

**Results** Twenty-seven articles were identified but only 6 were considered appropriate for analysis. This represented 1527 patients in total, 693 randomised to MMC and 834 to BCG. Both MMC and BCG were effective in delaying tumour recurrence following TUR. Statistical analysis indicated evidence of significant heterogeneity between trials ($P$ = 0.0009). The weighted mean (reciprocal of the variance) log hazard ratio (variance) for recurrence for the six trials was –0.016 (0.005). This indicated no significant difference between MMC or BCG ($P$ = 0.83). A subgroup analysis of three trials that included only high risk Ta and T1 patients, indicated no heterogeneity ($P$ = 0.25) and a log hazard ratio (variance) for recurrence of -0.371 (0.012). With MMC used as the control in the meta-analysis, a negative ratio is in favour of BCG and in this case it is highly significant ($P$ = 0.0079). Local toxicities (dysurea, cystitis, frequency and haematurea) were associated with both MMC (34%) and BCG (27%). Systemic toxicities, such as chills fever and malaise were observed with both agents although skin rash was more common with MMC.

**Conclusion** The data from the present meta-analysis indicates that recurrence-free survival was significantly prolonged with intravesical BCG compared to MMC in patients with a high risk of tumour recurrence.

### P80 PHOTODYNAMIC THERAPY FOR SUPERFICIAL BLADDER CANCER UNDER LOCAL ANAESTHETIC

C Briggs[1,2], DCShackley[1,2], A Gilhooley[1], C Whitehurst[2], JV Moore[2], CD Betts[1], K O'Flynn[1], NW Clarke[1], [1]Paterson Institute for Cancer Research, Christie Hospital, Manchester M20 9BX, [2]Hope Hospital Department of Urology, Salford M6 8HD

**Introduction** Photodynamic therapy (PDT) using 5-aminolaevulinic acid (ALA) is a novel technique used in the treatment of certain skin cancers. We report a phase 1 trial evaluating ALA PDT in the treatment of superficial bladder cancer under local anaesthetic (LA).

**Methods** 18 patients with recurrent superficial transitional cell carcinoma (stage Ta/CIS; grades 1–3) were treated using escalating doses of ALA (3–6%) and light (25–50 $Jcm^{-2}$). The following intravesical LA techniques were used: (i) no LA (ii) passive diffusion of 40 ml 2% lignocaine (iii) electromotive drug administration (EMDA) of salt-free 2% lignocaine. Pain was assessed using a linear analogue score on a scale of 0–10. Tolerability and toxicity was assessed using the different LA, light and ALA regimes.

**Results** Patients (n=2) undergoing 3% ALA PDT without LA experienced pain/spasm and the procedure was abandoned. Using lignocaine all patients (n=8) tolerated the procedure well [median pain score 1(range 0–2)]. On increasing the ALA dose to 6%, 6 patients reported pain with a median pain score of 8 (range 5–10) with 3 failing to tolerate the intended light dose. Using the most potent LA technique (EMDA of lignocaine) with maximum light and ALA dose, 2 patients were successfully treated (pain score 2).

All patients had short-lived bladder irritability following the procedure (resolving within 3 weeks) though this was substantially reduced with intravesical dexamethasone. There was no subjective/objective effect on long-term bladder function and no reported photosensitivity. Serum lignocaine levels in all patients were minimal. Of the 12 patients tolerating the procedure, 9 had significant tumour response, 6% ALA being most efficacious.

**Conclusion** Bladder ALA PDT is safe and with appropriate LA is well-tolerated. It is effective in a proportion of cases and with further refinement it may be possible for use on an out-patient basis under LA.

### P81 COULD CONFORMAL (CFRT) OR INTENSITY MODULATED RADIOTHERAPY (IMRT) REDUCE TOXICITY IN THE RADIOTHERAPY (RT) OF BLADDER CANCER?

RA Huddart[1,2], JN Staffurth[2], EJ Adams[1], VN Hansen[1], The Royal Marsden Hospital[1], The Institute of Cancer Research[2], Sutton, Surrey, SM2 5PT. UK

Trials of CFRT have demonstrated that reducing the volume of irradiated normal tissue can reduce incidence of late toxicity. The main organ at risk of toxicity in radiotherapy for bladder cancer is the bladder itself. This toxicity limits dose and hence efficacy of treatment. Our previous pilot data suggested that reducing the high dose volume may reduce bladder toxicity. This thesis is to be tested in a multicenter phase III study commencing 2001 (BC2000).

**Aims** To investigate the potential of different treatment strategies to reduce normal tissue irradiation while treating localised bladder cancer.

**Method** A patient with localised bladder carcinoma was planned using a conventional RT (CvRT) plan to cover whole bladder & extravesical disease with a 2 cm margin (WB). This was compared to a CFRT plan and to 2-phase treatment plans (PB)(50Gy to whole bladder and 14Gy to tumour with 2 cm margin) with CvRT and CFRT and a novel concomitant boost technique (ComRT) using 2 segments per beam. These plans were further compared to 3-field (3F) & 5F IMRT plan (generated on ©HELAX-TMS) and 3F, 5F & 9F IMRT plans (©CORVUS). Dose volume histograms (DVHs) were produced for non-target bladder (outside gross tumour), rectum and bowel.

**Results** Summarised in table below:

| STRUCTURE | Non-target bladder | | Rectum* | Bowel* | |
|---|---|---|---|---|---|
| PLAN | %>55 Gy | %>60 Gy | %>50 Gy | %>40 Gy | %>50 Gy |
| 3F CvRT WB | 100 | 100 | 33 | 80 | 56 |
| 3F CFRT WB | 100 | 100 | 29 | 69 | 47 |
| 3F CvRT PB | 45 | 41 | 15 | 70 | 49 |
| 3F CFRT PB | 38 | 31 | 15 | 61 | 38 |
| 3F Cv ComRT PB | 42 | 37 | 20 | 62 | 52 |
| 3F CF Com RT PB | 35 | 30 | 22 | 56 | 39 |
| 3F HELAX IMRT | 36 | 24 | 5 | 36 | 25 |
| 5F HELAX IMRT | 39 | 23 | 11 | 35 | 25 |
| 3F CORVUS IMRT | 50 | 20 | 15 | 53 | 29 |
| 5F CORVUS IMRT | 37 | 22 | 4 | 39 | 25 |
| 9F CORVUS IMRT | 36 | 23 | 5 | 43 | 21 |

*Proportion of small bowel or rectum in CT-scanned volume

**P81 Cont'd**

**Conclusion** The benefit of CFRT over CvRT is small. 2-phase bladder and boost plans can reduce the volume of non-target bladder, rectum and bowel irradiated. IMRT can achieve further gains in normal tissue avoidance over CFRT plans. On this preliminary data a 5F CORVUS IMRT plan or 3F HELAX plan would seem to be optimum. Data on additional patients is under evaluation and will be presented.

### P82 SCREENING PANCREATIC JUICE FOR P53 MUTATIONS USING A YEAST FUNCTIONAL ASSAY

JThreadgold[1], N Howes[1], I Ellis[2], I Gilmore[1], H Smart[1], M Lombard[1], W Greenhalf[1] & JP Neoptolemos[1], [1]Dept of Surgery, Univ of Liverpool, Liverpool, L69 3GA, [2]Dept of Clinical Genetics, Alder Hey Children's Hospital, Liverpool, L12 2AP

Despite only representing 2–3% of total cancer cases, pancreatic cancer is the 4th biggest cancer killer. The most common form of pancreatic cancer is Pancreatic Ductal Adenocarcinoma (PDAC). This disease is characterised by rapid metastasis and high mortality. Early surgery dramatically increases survival but differentiating malignancy from benign conditions, such as pancreatitis, is often impossible at this stage. The molecular pathogenesis of PDAC is thought to involve mutations in k-ras, p16, SMAD4 and p53 genes. Over 700 p53 mutations (>90% of which are found in exons 5–8) can be detected in cancer. A functional assay is required to detect and identify low copy numbers of mutant p53 in a background of wild type sequences.

The aim is to develop and test a novel screening approach for PDAC based on identification of p53 mutations in pancreatic juice. The European Registry of Hereditary Pancreatitis (HP) and Familial Pancreatic Cancer (FPC) (EUROPAC) has identified patients with a predisposition for pancreatic cancer. DNA was extracted from pancreatic juice collected from patients undergoing endoscopic examination (ERCP) and also from tumour tissue removed from cancer patients. A yeast functional assay of p53, based on the system developed by Dr. Iggo (ISREC) has been developed. p53 exons 5–8 are amplified and linked by polymerase chain reaction (PCR). The PCR product is then transformed along with a plasmid containing the coding sequence of human p53 into a yeast strain carrying a reporter gene, ADE2, under p53 transcriptional control. Wild type p53 will result in ADE2 activation and the production of white yeast, while recombination between plasmid p53 and mutant p53 PCR product prevents ADE2 activation and the resultant yeast are red in colour. DNA is extracted from selected red colonies and screened for the presence of a plasmid by PCR. Positive colonies are then transformed into *E. coli* or sequenced directly.

The viability of the screening technique has been confirmed with controls: 5 pancreatic cancer cell lines, 30 matched pancreatic juice and tissue samples from PDAC patients, 20 matched juice and tissue samples from chronic pancreatitis patients and 10 normal controls. We have found that this system of p53 mutation analysis is both sensitive and specific and may be a viable strategy for screening of high-risk patients.

### P83 THE ROLE OF HSP90 IN REGULATION OF APOPTOSIS IN PANCREATIC CANCER

AB Mason, M Burkitt, CJ Magee, D O'Connor and W Greenhalf, Department of Surgery, 5th Floor UCD, Duncan Building, Royal Liverpool University Hospital, Daulby Street, Liverpool, L69 3GA

Hsp90 is a molecular chaperone that binds and maintains proteins in a stable inactive state. Inhibition of Hsp90 results in cell cycle arrest and apoptosis. Targets of Hsp90 directly involved in cell cycle progression are well known (e.g. Cdk4, Raf-1), the role of Hsp90 in apoptosis regulation is unclear.

We have selected genes on the basis of their ability to overcome the toxicity in yeast. One of these was the human gene BASS2. We have shown by Northern blot that BASS2 is highly expressed in pancreas compared to other organs (skeletal muscle, heart, lung, liver and brain). In addition we identified the yeast mitochondrial import protein, MAS70 gene as a Bax antagonist. In a screen of a 2-hybrid cDNA library BASS2 was found to bind to Hsp90. MAS70 has previously been shown to also bind to Hsp90. We have shown by Immunohistochemistry that Hsp90 localises predominantly to the nucleus in pancreatic ductal adenocarcinoma (PDAC) whereas in normal pancreatic ductal cells it is predominantly cytoplasmic. We are at present using immunofluorescence to further characterise the localisation of Hsp90 and to study the affect of co-expression of BASS2 on this localisation.

To further characterise Hsp90 binding to BASS2, full length Hsp90 and the N- and C-terminal fragments of Hsp90 and MAS70 were amplified from cDNA. MAS70 and BASS2 were cloned into the yeast 2-hybrid vector pAS2-1, in frame with the Gal4 DNA binding domain, and the Hsp90 inserts were cloned into the complementary vector pGAD424, in frame with the Gal4 transcriptional activation domain. The plasmids were then co-transformed into the yeast strain CG1985 (Clontech), and the interactions between these proteins was assessed by the production of β-galactosidase assayed using X-gal. BASS2 binds to the C-terminal of Hsp90; co-expression of the Hsp90 co-chaperone Cdc37 was shown not to influence this binding.

### P84 EFFECT OF STABLE TRANSFECTION WITH GASTRIN ANTISENSE ON THE GROWTH OF AN ORTHOTOPICALLY TRANSPLANTED HUMAN PANCREATIC TUMOUR

AD Gilliam, DF McWilliams, TM Morris, PA Clarke SA Watson, Academic Unit of Cancer Studies, University Hospital, Nottingham, NG7 2UH

**Background** There is increasing evidence to suggest that human pancreatic carcinomas produce gastrin and express the CCK-2 receptor, contributing to their malignant potential.

**Aim** To assess the effect of transfection of the gastrin antisense gene on tumour progression of a xenografted human pancreatic tumour cell line, thereby investigating the role of gastrin as an autocrine growth factor.

**Methods** Lysates of the human pancreatic tumour cell line Pan 1 were assayed for CCKBR isoforms by Western blotting using rabbit polyclonal antibodies raised against an N-terminal epitope sequence of the classical CCK-2 receptor. RNA isolated from PAN-1 was reverse transcribed, amplified by polymerase chain reaction, and hybridised with gastrin and CCK-2 primers. Reverse Transcriptional real-time PCR was performed on the cDNA using the DNA binding fluorescent dye SYBR green. Quantification of relative gene expression levels (the ΔΔct value) was obtained by comparison to the housekeeping gene, GAPDH. Panl was stably transfected with gastrin antisense and vector control genes. The liposome-mediated method, Tfx-20 (Promega, UK), was used and the plasmid and reagent were mixed and added to the culture medium. Successful transfection was confirmed by Southern blotting with a relevant oligoprobe. In vitro cell proliferation was assessed using the colorimetric MTT assay. An in vivo study was performed according to UKCCCR guidelines. 1e6 transfected cells were injected in 20 µl of PBS into the tail of the pancreas of nude mice. There were 10 mice in each group. Mice were terminated at 30 days and the cross sectional area and weights of the pancreatic tumours were assessed.

**Results** Western blotting confirmed the presence of the CCK-2 receptor and real time PCR confirmed gastrin and CCK-2 expression in the Pan 1 cell line. The Pan1 gastrin antisense cell line had a significantly reduced basal growth in serum-free medium when compared to the vector control (43–50% inhibition, $P = 0.01$, Student's t-test). In addition, there was a reduction in the size ($P = 0.051$, paired t-test) and weight ($P = 0.033$, paired t-test) of the Pan 1 tumours transfected with the antisense gene vs control vector in vivo.

**Conclusion** Gastrin may play an important role in the progression of pancreatic carcinoma by means of an autocrine and paracrine pathway, supporting the use of anti-gastrin agents for treatment of the disease.

### P85 THE REGULATION OF EXPRESSION OF S100A4 IN PANCREATIC DUCTAL ADENOCARCINOMA BY THE ZINC FINGER BKLF

CW Herbert[1], BR Barraclough[2] and W Greenhalf[1], [1]Department of Surgery, 5th Floor UCD, Duncan Building, Royal Liverpool University Hospital, Daulby Street, Liverpool, L69 3GA, [2]School of Biological Sciences, University of Liverpool, L69 3BX

Pancreatic Ductal Adenocarcinoma has the highest incidence to mortality rate of any human cancer with a 5 year survival rate of just 0.4% worldwide. Of the 37220 cases diagnosed in Britain between 1986 and 1993, only 938 patients survived for 5 years or more. The prognosis for sufferers of this disease is bleak because of the high frequency of metastases. The use of chemotherapeutics and radiotherapeutics has only produced a marginal survival benefit. Surgical resection is the main treatment for this disease but in most cases the cancer has progressed to a stage where it has become inoperable. S100A4(p9Ka, mts1), a Calcium-binding protein, has been shown to be overexpressed in the advanced stages of many cancers and increased expression levels have been associated with the metastatic phenotype.

Using immunohistochemical staining and real-time PCR, we have shown that S100A4 is highly expressed in Pancreatic cancers, especially in the hepatic metastases, compared to normal tissue. Using a yeast one-hybrid system and DNA footprinting, we have shown that BKLF (Basic Kruppel-Like Factor), a Zinc Finger protein which acts as a repressor of transcription, binds to specific CACCC sequences in the minimal S100A4 promoter. BKLF has been shown to associate with the co-repressor CtBP (C-terminal Binding Protein), a short range repressor which acts on the core promoter region and can indirectly interact with Rb protein, *in vivo* and *in vitro*. The binding to CtBP has been shown to be essential for the repression activity of BKLF. Using real-time PCR we have studied the expression levels of these genes in normal and cancerous pancreas tissue samples, as well as in Pancreatic cell lines.

### P86 REAL-TIME KRAS MUTATIONAL ANALYSIS IN THE DIAGNOSIS OF EARLY PANCREATIC DUCTAL ADENOCARCINOMA

T Wong, N Howes, MG Lombard, HL Smart, I Gilmore, R Sutton, F Campbell, Y Kang, I Ellis, W Greenhalf, JP Neoptolemos, Dept. of Surgery, University of Liverpool, Liverpool L69 3GA

KRAS mutations occur in 75–80% of pancreatic ductal adenocarcinoma (PDAC). Conventional screening modalities are limited in detecting early, potentially curable lesions and in this study, we investigate the role of real-time mutational analysis as a tool for the diagnosis of early pancreatic ductal adenocarcinoma (PDAC).

DNA was prepared from tissue, or from pancreatic juice/bile extracted from patients during ERCP. This was analysed by real-time ARMS, involving DNA amplification using primers, which have a 3′ base corresponding to the relevant KRAS mutations. The appearance of product is compared to the appearance of a product that will be produced equally from mutant and wildtype KRAS.

From 55 patients with PDAC-15/22 (68%) had mutations in juice and 12/33 (52%) in bile. From 36 patients with chronic pancreatitis (CP), 7/27 (35%) had mutations in juice and 4/9 (44%) in bile. In patients with ampullary carcinoma, 6/6 (100%) had juice mutations and 6/9 (67%) bile mutations. Of patients with no pancreatic diseases, 4/9 (44%) had juice mutations and 14/42 (33%) had mutations in bile. The types of mutation were correlated with mutations in biopsy/surgical samples. There was no statistical correlation with age, smoking or alcohol history.

Although the technique used has detected KRAS mutations in PDAC patients successfully, it has also detected mutations in patients with CP and even in patients with no pancreatic diseases. KRAS mutation detection therefore lacks the specificity required to identify malignancy. Therefore KRAS mutation detection if it is to be used as a diagnostic tool needs to be used in combination with other molecular analysis approaches.

### P87 PRELIMINARY RESULTS OF PHASE I/II STUDY TO INVESTIGATE THE USE OF GEMCITABINE IN COMBINATION WITH RALITREXED IN LOCALLY ADVANCED OR METASTATIC ADENOCARCINOMA OF THE PANCREAS

A Michael[1], A Maravcyas[2], M Hill[3], H Wasan[4], F Lofts[1], [1]St. George's Hosp. London SW17 0QT, [2]Princess Royal Hospital Hull HU8 9HE, [3]GI Unit RMH Sutton Surrey, [4]Hammersmith Hospital London W12 0HS

Gemcitabine has been shown to improve the clinical status of patients with carcinoma of the pancreas relative to 5FU. Ralitrexed is a quinazololine folate analogue, specific thymidylate synthase inhibitor with a similar mode of action to 5FU. At a dose of 3 mg/m$^2$ ralitrexed has been shown to be equally effective to 5FU/LV in metastatic colon cancer. A phase II trial of ralitrexed in patients with advanced pancreatic cancer revealed a response rate of 5%. Combining gemcitabine with ralitrexed may result in increased response rate without marked increase in adverse events. We conducted a phase I/II study of gemcitabine in combination with ralitrexed in locally advanced and metastatic adenocarcinoma of the pancreas. Gemcitabine was given on day 1 and 8 with ralitrexed on day 1 of a 21 day cycle. Starting dose of ralitrexed was 3.0 mg/m$^2$ with gemcitabine 1000 mg/m$^2$. Gemcitabine was escalated by 200 mg/m$^2$ for each cohort up to a maximum dose of 1400 mg/m$^2$. Dose limiting toxicities (DLT) were defined as grade 3/4 haematologic toxicities according to NCIC-CTC and all grade 3/4 non-haematologic toxicities with the exception of nausea, vomiting, alopecia, transaminitis.

Six patient were recruited in cohort 1 (3M/3F), median age 66 (range 53–76) and median Karnofsky PS 85%. There were 2 cases of grade 3 leucopenia and 2 cases of neutropenic fever. One patient experienced grade 3 skin toxicity, with a pemphigoid-like picture. 2 patients had positive clinical benefit response (CBR), 2 were not evaluable and 2 had negative CBR. On CT criteria there were 2 PD, 2 SD and two non-evaluable patients.

Twelve patients were recruited to cohort 2 (10M/2F) with data available on 9, median age 57 (range 39–67) and median PS of 86%. 1 patient had neutropaenic fever and 1 experienced diarrhoea. 2 patients had negative CBR, 3 stable and 0 had a positive CBR. On CT criteria there were 4 cases of PD, 3 cases of SD and 2 patients were not evaluable. Due to poor tolerance of the dose level the study continues at a lower dose level of ralitrexed at 2.5 mg/m$^2$.

### P88 CHARACTERISATION OF OESTROGEN RECEPTOR TRANSCRIPTION COMPLEXES IN OVARIAN CANCER CELLS AND IDENTIFICATION OF OESTROGEN-REGULATED RESPONSES

AJM O'Donnell, D Burns, K Macleod, JF Smyth, SP Langdon,
ICRF Medical Oncology Unit, Western General Hospital, Edinburgh

The majority of ovarian cancers display expression of oestrogen receptors (ERs). Using a range of ovarian cancer cell lines, we have demonstrated that ovarian cancer cells that express moderate-high levels of ER, are growth-stimulated by 17β-oestradiol ($E_2$) in vitro (Br. J. Cancer: 62, 213, 1990). The anti-oestrogen tamoxifen is able to inhibit this growth stimulation in vitro and in vivo and clinical studies have shown that it possesses some therapeutic activity in ovarian cancer. Recent data have indicated that an oestrogen response in a target tissue is not only determined by the amount of receptor present but also by the composition of ER dimer and the presence of cofactors which integrate the receptor with components of the basal transcriptional machinery.

With an aim to characterising the composition of oestrogen transcriptional complexes present within ovarian cancer cells we have investigated the expression of ER isoforms (ER-α and ER-β) and transcription co-activators/co-repressors in a range of ovarian cell lines and primary cancers. RT-PCR showed that ER-α mRNA was expressed in PE01 and PE04 cell lines but not in the PE014 cell line. These results correlate with growth responsiveness to $E_2$. Interestingly however, ER-α mRNA was also found to be expressed in the SKOV3 cell line which failed to be growth stimulated by $E_2$. ER-β mRNA was found to be expressed in all 4 cell lines, with highest levels present in the ER-α negative PE014 cell line. The co-activators SRC-1, TIF-2, AIB-1, p300 and ZAC-1, co-repressors NCoR-1 and SMRT, and other modulatory cofactors, SUG-1, BRG-1, FAP-1 and prothymosin alpha were all expressed to varying degrees in ovarian cancer cell lines and primary tumours when measured by real-time PCR and associations with transcriptional responses are currently being explored. Oestrogen-regulated mRNA expression was investigated by differential display RT-PCR. Products that appeared differentially expressed in PE01 ovarian cancer cells after treatment with $10^{-10}$M $E_2$ for 24 h were excised from acrylamide, re-amplified by PCR and subcloned into pGEM-T Easy vector. These were then sequenced and identified. These included NF-1 related locus, ZXDA, Histone Deacetylase and S19 and expression levels of these are being quantified by real-time PCR. mRNA from $10^{-10}$M $E_2$-treated cells was also analysed on the Clontech Atlas Human Cancer 1.2 cDNAexpression array. Of 1185 genes analysed, 19 increased in expression by at least 2-fold in PE01 cells treated with $E_2$ relative to untreated PEO1 cells, while 78 genes decreased. Among the expressed genes increasing after $E_2$ treatment were FRA-1 and Cathepsin D while expression levels of erbB2, IGFBP-3, UPA and fibronectin were decreased.

This study highlights the complexity of the ER transcription complex and shows that oestrogen-regulated expression of critical genes involved in the aetiology and progression of ovarian cancer likely involves multiple transcriptional cofactors.

### P89 ERBB RECEPTOR ACTIVATION IS ASSOCIATED WITH GROWTH, MIGRATION AND MORPHOGENESIS IN OVARIAN CANCER

JM Sewell, LMR Gilmour, JF Smyth and SP Langdon, ICRF Medical Oncology Unit, Western General Hospital, Edinburgh, EH4 2XU

Members of the erbB or HER family of (type I) tyrosine kinase receptors are frequently over-expressed in human cancers, and increased expression of both the epidermal growth factor receptor (EGFr, erbB1, HER-1) and erbB2 (HER-2) have been associated with poor survival in ovarian cancer. Such data suggests an involvement of these receptors in the growth and progression of this disease. Transforming growth factor alpha (TGFα) activates the EGFr while the heregulins or neuregulins (eg HRGβ1) activate erbB3 and/or erbB4. Upon activation these receptors homo-or heterodimerise with other family members, preferentially erbB2, initiating intracellular signalling cascades such as the Ras/ERK kinase pathway.

To assess the relative contributions of these receptors to growth, migration and morphogenesis, a panel of ovarian cancer cell lines (PEO1, SKOV-3, OVCAR-5, 41M, PEO1$^{cddp}$ and A2780) were treated with either TGFα or HRGβ1 ($10^{-9}$M). TGFα stimulated growth of SKOV-3, PEO1 and 41M cells while HRGβ1 stimulated growth of PEO1, 41M and OVCAR-5 cells. Use of blocking antibodies indicated that HRGβ1 was acting via erbB3 and not erbB4. In all cell lines (except PEO1$^{cddp}$ cells which were growth inhibited), HRGβ1 was the more potent activator of growth. In migration assays this trend was reversed; while HRGβ1 stimulated migration of PEO1 and OVCAR-5 cells on collagen and fibronectin, and OVCAR-5 on laminin, TGFα gave a greater stimulation to each of these situations and also drove SKOV-3 cell migration on all three matrices. Furthermore, TGFα but not HRGβ1 stimulated morphogenic changes in several of these cell lines. Statistically significant associations were identified between the extent of migration promoted by TGFα and erbB2 expression levels (on collagen $P$=0.01, on laminin $P$=0.04: Pearson correlation), while using a wider cell panel of 13 cell lines, a significant association between magnitude of HRGβ1 growth response and erbB2 expression was also observed ($P$=0.005: Pearson correlation).

TGFα exposure activated both the ERK pathway and PI3 kinase pathway in these cell lines as indicated by Western blot detection of increased levels of phospho-ERK and phospho-Akt respectively. The relative contribution of these two pathways to the differing functions is being assessed by the use of specific inhibitors targeted to components of these pathways.

These data indicate that the erbB receptors are involved not only in growth regulation but also in migration and morphogenesis. Our findings suggest that while erbB3 activation primarily drives growth, EGFr signalling is more related to migration, although neither function is exclusive.

### P90 USE OF CA 125 TO DEFINE PROGRESSION OF OVARIAN CANCER IN PATIENTS WITH PERSISTENTLY ELEVATED LEVELS

Gordon JS Rustin, Maria Marples, Ann E Nelstrop, Mohamed Mahmoudi, Tim Meyer, Department of Medical Oncology, Mount Vernon Hospital, Northwood, Middlesex HA6 2RN, UK

**Purpose** To determine an accurate definition for progression of ovarian cancer in patients with a persistently elevated serum CA 125.

**Patients and Methods** A retrospective analysis was performed on 300 patients with epithelial ovarian carcinoma with at least one measurement of CA 125. Eighty-eight patients had persistently elevated CA 125 levels (>23 U/ml) on completion of first-line chemotherapy. The date of progression according to clinical or radiological criteria was ascertained in these patients, and compared with the date of progression according to CA 125. This was defined as the date the CA 125 level first rose to at least twice its nadir level confirmed by a second sample also at least twice the nadir.

**Results** Eighty of the 88 patients had evidence of progression by both standard and CA 125 criteria. In 6 of these patients no sample was obtained to confirm the doubling of the CA 125, and in 13 patients CA 125 doubling was documented after the date of clinical progression. Seven patients never developed doubling of CA 125; two died from causes other than ovarian cancer, and five are counted as false negative. Only one patient had a false positive prediction of progression according to CA 125, dying from myocardial infarction before evidence of clinical progression. This gave the CA 125 definition of progression a sensitivity of 94% and a positive predictive value of 98.8%.

**Conclusion** In patients with persistently elevated levels of CA 125, doubling of CA 125 from its nadir level accurately defines progression. If confirmed, these CA 125 criteria should be used as additional endpoints in clinical trials, as proposed by Vergote et al (JNCI 2000; 92:1534–5).

### P91 ANDROGENS HAVE DIRECT EFFECTS ON HUMAN OVARIAN SURFACE EPITHELIUM

RJ Edmondson[1,2], JM Monaghan[2], BR Davies[1], [1]Dept of Surgery, University of Newcastle upon Tyne, [2]Northern Gynaecological Oncology Centre, Gateshead, Tyne and Wear

**Introduction** The pathogenesis of epithelial ovarian cancer remains unclear. From epidemiological studies raised levels of androgens have been implicated to increase the risk of developing the disease. In addition, epithelial ovarian cancer cells have been shown to express the androgen receptor. The purpose of this study was to determine the responses of normal human ovarian surface epithelium (OSE) to androgens.

**Methods** We have established primary cultures of human ovarian surface epithelium from patients undergoing oophorectomy for benign disease. Epithelial phenotype was confirmed by staining with a panel of cytokeration antisera. Total RNA was isolated from these cultures and the presence or absence of mRNA encoding for the androgen receptor (AR) was demonstrated using RT-PCR. The presence of androgen receptor in sections of normal ovary was also investigated using an antibody against androgen receptor. The effects of androgens on DNA synthesis and apoptosis was determined using $^3$H thymidine incorporation and JAM assays respectively.

**Results** Eight of eight (100%) cultures tested expressed mRNA encoding androgen receptor. The presence of AR in OSE of sections of normal ovaries was demonstrated in all sections.

Mibolerone, a synthetic androgen, caused a marked stimulation of DNA synthesis in 8 of 10(80%) cultures when used at a concentration of 1 nM. Two cultures showed no significant induction of DNA synthesis. Mibolerone also caused a significant decrease in apoptosis in 2 of 4 (50%) cultures tested.

**Conclusions** We have demonstrated that the ovarian surface epithelium is an androgen responsive tissue and that androgens, at physiological doses, can cause an increase in proliferation and a decrease in apoptosis. These findings allow for the possibility that androgens may be involved in ovarian carcinogenesis.

### P92 A PHASE I INTRA- & INTER-PATIENT DOSE RANGING STUDY OF GEMCITABINE, CARBOPLATIN AND PACLITAXEL (GCP) IN PREVIOUSLY UNTREATED PATIENTS WITH EPITHELIAL OVARIAN CANCER, PRIMARY PERITONEAL MALIGNANCY, OVARIAN CARCINOSARCOMA AND FALLOPIAN TUBE CARCINOMA

CJ Poole, SD Jordan, HB Higgins, VR Archer, GE Pemberton, CRC Clinical Trials Unit, University of Birmingham B15 2TT and City Hospital Birmingham, B18 7QH

**Introduction** A Phase II trial of the GCP (C-AUC5, d1/G-800,d1&8/P-175,d1) triplet has shown encouragingly high response rates in EOC, but troublesome thrombocytopenia(1). As the median MTD of carboplatin and paclitaxel determined by intra-patient dose-escalation is comparable with that determined conventionally (2), we decided to undertake an intra-patient dose escalation study to determine the range of inter-patient variation in MTD and examine the feasibility of developing an individual dose optimisation nomogram. By using a novel hybrid inter-and intra-patient dose-escalation design we hoped to discriminate acute from cumulative toxic effects. We also wished to test whether co-scheduling paclitaxel with gemcitabine d1&8; might attenuate thrombocytopenia.

**Methods** Entry level doses: G-800 mg/m$^2$ d1&8; C-AUC5, d1 only; P-75 mg/m$^2$ d1&8. Patients (pts) were treated with 6–8 cycles in 3–6 pt cohorts. One component was dose-escalated by 10% each cycle (intra-patient), and successive cohorts started one dose-level above that of the previous entry level dose (inter-patient) towards a target ANC1.0–1.5×10$^9$/L, platelets 75–100×10$^9$/L, or grade<3 non-hematologic toxicity, all d22, thus defining MTD. Creatinine clearance was recalculated each cycle.

**Results** All 21 pts registered have completed ≥4 cycles (range 4–8) and 18, ≥6 cycles. Cohort 1, (entry level, n=6), cohort 2 (P-82.5 mg/m$^2$, n=3), cohort 3 (C-AUC5.5, n=3) & 4 (P-90 mg/m$^2$, n=3) are complete; entry level doses for cohort 5 = cohort 1, cycle 5, ie G-900 mg/m$^2$, C-AUC5.5, P90 mg/m$^2$ and cohort 1, cycle 6 increases C to AUC6. MTD's occurred at all 9 dose levels, with a median of G-800 mg/m$^2$ d1&8, C-AUC5.5 d1, & P-90 mg/m$^2$ d1&8, dose level 4. Myelosuppression was dose-limiting in 16 pts and grade 3 fatigue in 1 pt. 5/18 completed pts required platelet and 16/18, RBC transfusions. One suffered neutropenic sepsis (ANC nadir = 0.3). All pts with baseline raised CA125 (12/21) have Rustin-defined responses. Day 22 blood counts show evidence of a dose-response curve for a paclitaxel neutrophil and platelet-sparing effect in the range 75–90 mg/m$^2$ (but not with gemcitabine doses >800 mg/m$^2$).

**Conclusion** the intra-patient/inter-patient dose-escalation approach is feasible; GCP MTD's are widely variable, which prompt consideration of dose individualisation; paclitaxel has modest dose-related neutrophil and platelet protection effects; there is no evidence of cumulative myelosupression.

1. SW Hansen, et al Proc ASCO 1999: Abstract 1379
2. CJ Poole, et al, Annals of Oncology 2000: Vol 11; P141

### P93 ZD0473 PHASE II MONOTHERAPY TRIAL IN SECOND-LINE OVARIAN CANCER

M Gore[1], RJ Atkinson[2], L Dirix[3], D Rischin[4], P Beale[5], P Harnet[6], D Hacking[7], H Cure[8], J Cosaert[9], [1]Royal Marsden Hospital NHS Trust, London, UK, [2]Belfast City Hospital, N Ireland; [3]Oosterveldann, Wilrijk, Belgium, [4]Peter McCallum Cancer Institute, St Andrews Place, East Melbourne, Australia; [5]Royal Prince Alfred Hospital, Camperdown, Australia, [6]Nepean Hospital Penrith, Kingswood, Australia; [7]Durban Oncology Centre, Mayville, South Africa, [8]CAC Jean Perrin, Clermont Ferrand, France, [9]AstraZeneca, Alderley Park, UK

New drugs demonstrating activity in platinum-resistant ovarian tumours are required. ZD0473 is a new generation platinum drug that shows evidence of an extended spectrum of antitumour activity and overcomes platinum resistance mechanisms, and is a good candidate for evaluation in refractory ovarian cancer. This Phase II open-label, two-stage, multicentre trial aims to assess the efficacy and tolerability of ZD0473 as second-line therapy in patients with ovarian cancer who have failed one prior platinum-based chemotherapy regimen. Patients received ZD0473 (1-h iv infusion) on day 1, every 3 weeks and were initially recruited at a dose level of 120 mg/m$^2$. This dosage was subsequently increased to 150 mg/m$^2$ during the course of the trial, as the original starting dose was well tolerated. Patients were evaluated in 4 cohorts related to the time of relapse/progression following completion of first-line platinum-based chemotherapy: those who relapsed/progressed after (1) ≤12 weeks having shown no response; (2) ≤12 weeks having responded; (3) >12 weeks and ≤26 weeks; (4) >26 weeks. Cohorts 1–3 were considered to be resistant and cohort 4 sensitive. To date, 29 patients (15 resistant, 14 sensitive; median age 58 years; performance status 0/1) have been recruited and have received 84 cycles in total. Cycle delay due to toxicity was experienced by 7/29 patients. An objective response was observed in 2/14 evaluable resistant patients (1CR, 1PR) and 5/12 evaluable sensitive patients (2CR, 3PR). Stable disease was observed in 2 resistant and 3 sensitive patients, including evidence of tumour shrinkage in 3 patients. ZD0473 had a manageable safety profile; there were no drug-related withdrawals or deaths, and grade 3/4 haematological effects included thrombocytopenia (14/29 patients), neutropenia (13) and anaemia (12). No evidence of clinically significant nephrotoxicity or neurotoxicity was observed. Preliminary results indicate that ZD0473 is active in second-line ovarian cancer, including resistant disease. The trial is ongoing.

### P94 PHASE II STUDY OF OXALIPLATIN, 5-FU AND LEUCOVORIN IN PATIENTS WITH RELAPSED OVARIAN CANCER

AL Thomas[1], A Osman[1], B Morgan[2], S Khanna[1], RP Symonds[1], K O'Byrne[1], Depts of Oncology[1] & Radiology[2], Leicester Royal Infirmary, Leicester LEI 5WW

In relapsed ovarian cancer response rates to second-line therapies remain frustratingly low. There is a need to develop more effective regimens which are well-tolerated as these patients often have marked symptoms. Both oxaliplatin (OXA) and 5FU have shown single agent activity in pts with recurrent ovarian carcinoma. In view of the in vitro synergistic effect of OXA and 5FU and the tolerability of this combination in pts with colorectal cancer, we studied these agents in pts with relapsed ovarian carcinoma following failure of first-line platinum based chemotherapy. Oxaliplatin 85 mg/m$^2$ IV two weekly and weekly 5FU 370 mg/m$^2$ with leucovorin 30 mg IV were administered and efficacy and safety data collected. To date 14 patients, with median age 58 years (range 46–66) have been treated. Performance status was 0 in 2 pts, 1 in 7 pts and 2 in 5 pts. Pts had stage 3 (12 pts) or 4 (2 pts) disease. Twelve pts had at least 1 debulking operation 1 pt had 2 debulking procedures and 1 did not have any surgery. All pts had 1 prior platinum based chemotherapy and 3 had been rechallenged with carboplatin. Eight pts had also received a taxane. Pts received a median of 6 cycles of treatment (range 2–11). Most common toxicities were nausea and vomiting (1 pt at grade 3) and peripheral neuropathy (2 pts at grade 3). One grade 3 neutropenia and 1 grade 3 thrombocytopenia have been seen, however there has been no febrile neutropenia. Only 3 pts needed dose reductions due to toxicity and of these 1 discontinued treatment. Of 10 evaluable pts, 1 complete and 2 partial responses have been documented, based on WHO criteria. Using the technique of Rustin et al. (JCO 1996, 14:5: 1545–1551) Ca125 responses occurred in 3 patients. Median survival has yet to be reached. This combination of OXA and 5FU is extremely well tolerated and even in this small pt group encouraging responses have been documented. Recruitment continues and further data will be presented.

### P95 ASSESSMENT OF TUMOUR MARKERS AND APOPTOSIS IN LUNG CANCER

CD Macdonald, A Michael, KW Colston, JL Mansi, Department of OGEM St. George's Hospital Medical School, London SW17 ORE

Lung cancer is the commonest form of cancer in both sexes in the UK and has a poor prognosis. Numerous genes are associated with the growth and propagation of cancer including tumour suppresser gene p53, anti-apoptotic bcl-2, proliferation marker ki67 and growth factor receptor IGF-1R. These oncogenes have been widely studied in relation to prognosis and survival but findings have been inconsistent to date. There is no information available with respect to changes in gene expression with time or response to treatment.

The aim of this study was to determine intra and inter tumour variability of the markers ki67, bcl-2, p53 and IGF-1R and the level of apoptosis to determine if these are constantly expressed throughout the tumour.

Ten patients were selected at random from archived surgical material representing all types of lung cancer. Ten serial sections, 4–6 microns thick, were sectioned from each sample for each of the markers studied, a total of 50 slides per tumour. Oncogene expression was determined using an immunohistochemical kit from DAKO: EnVision™. The apoptotic index was determined using a kit from Intergen: ApopTag™. The slides were graded using two accepted methods by two independent markers. The coefficient of variation was calculated to give a percentage variation.

The results indicate that there is an inherent variation in expression of all the oncogenes studied. Ki67 expression, for example, varied from 10 to 200%. This raises questions about the reliability of these markers as a prognostic indicator when assessed by immunohistochemistry. Further research is required in order to evaluate the relevance of these tumour markers in a clinical setting.

### P96 BIOCHEMICAL EXPLORATION IN LUNG CANCER

Maria Dogaru, Natalia Rosoiu, Faculty of Medicine, "Ovidius" University, Constanta, Romania, Ro-8700

The glucose concentration and lactate dehydrogenase (LDH) in pleural effusion and blood samples guide the clinician for differential diagnosis between cancer and infectious diseases.

In this study we examined samples of pleural effusion and blood, collected from 50 patients, hospitalized in Constanta Lung Pathology Hospital. In 10 cases the cancer diagnosis have been confirmed; the other 40 have been diagnosed with infectious diseases, including tuberculosis.

We assayed the serum for glucose concentration (Glucose-oxydase method), protein concentration (Biuret method), LDH, ALT, AST (Kinetic method), urea (Urease method) and the pleural fluid for glucose, proteins, LDH (using the same methods).

In patients diagnosed with cancer, preliminary data were in accordance with the references, showing a significant decrease of pleural glucose concentration (15–60 mg/dl), compared with normal values of glycemia (70–110 mg/dl). Moderate increase of LDH (300–600 U/l) and moderate decrease (2,5–4,5 g/dl) of pleural proteins were noticed.

In infectious diseases, pleural glucose was slightly decreased or normal (50–100 mg/dl). Pleural LDH had high values (500–1000 U/l) compared to serum LDH decreased values. Proteins were higher than in cancer (4.0–6.5 g/dl).

In all patients, urea, ALT and AST had values in the reference range.

Our research shows the importance of pleural glucose and LDH assay in supporting diagnosis between cancer and infectious diseases. We consider that the decrease of pleural glucose concentration is determined by the increased catabolism of glucose in tumoral cells.

In the specialty references we did not find data referring to glucose metabolites (pyruvate, lactate, succinate), $NAD^+$ and FAD in lung pathology. We continue our research with the assay of these biochemical parameters and LDH isoenzyms in pleural fluid and blood samples.

### P97 CANCER SPECIFIC GENOMIC INSTABILITY IN BRONCHIAL LAVAGE AS A METHOD OF LUNG CANCER DETECTION

T Liloglou, P Maloney, G Xinarianos, M Hulbert & JK Field, Roy Castle International Centre for Lung Cancer Research, The University of Liverpool, 200 London Road, Liverpool L3 9TA

Genomic instability is the most common molecular alteration detected in human cancer[1,2]. Molecular alterations identified in lung cancer have also been detected in the bronchial lavage and sputum of lung cancer patients indicating a potential diagnostic use of such methods[3–6]. We have previously detected allelic imbalance and microsatellite alterations in DNA from bronchial lavage (BL) from patients with lung cancer but also individuals with no evidence of lung neoplasia posing the question whether genomic instability is an exclusive phenomenon of malignant diseases[3]. We have consequently established a robust multiplex fluorescent microsatellite assay and assessed the interassay variation leading to a calculated threshold of 0.23. By using this assay, we detected genomic instability in 95% of DNA specimens from lung tumours[6]. In this study, we assessed the specificity of above assay by examining DNAs from 80 BL samples from patients with lung cancer and patients with no malignant lung disease. When genomic instability at individual loci was analysed statistically in relation to clinical diagnosis, markers D3S1289 ($P$=0.033), D3S1300 ($P$=0.001), D13S171 ($P$=0.009) and D17S2179E ($P$=0.017) demonstrated significantly higher frequency of instability in BL specimens from lung cancer cases compared to those with non-malignant conditions. In contrast, markers D9S157, D9S161, D13S153 and D5S644 demonstrated lower specificity ($P$>0.05) for lung tumours. These results suggest that genomic instability in some regions may be related to high proliferation rates but not necessarily to cell commitment to malignancy. When genomic instability was scored using only the 4 cancer-specific markers the assay produced a sensitivity of 73.9% and a specificity of 76.5%. On combining the results from the cytological examination and the molecular assay, the detection sensitivity reached 82.6%. Our results indicate that genomic instability may be a potential marker for the early detection of lung cancer; however, we need to extend our observations and establish set(s) of cancer-specific genomic instability (CSGI) markers.

1. Perucho M (1996) Nature Med. *2*: 630–631
2. Lengauer C, et al. (1998) *Nature 396*: 643–649
3. Field JK, et al. (1999) *Cancer Res* 59: 2690–2695
4. Miozzo M, et al. (1996) *Cancer Res* 56: 2285–2288
5. Ahrendt SA, et al (1999) *JNCI* **91**: 332–339
6. Powell, et al (1999) *Clin Cancer Res* 5: 2025–2034
7. Liloglou T, et al. (2000), *Int J Oncol* 16: 5–14

### P98 SURVIVAL ANALYSIS AND PROGNOSTIC FACTORS IN NON-SMALL CELL LUNG CANCER

C Martin[1], H Bozcuk[2], [1]Dept. of Oncology, Norfolk and Norwich Hospital, Norwich, NR1 3SR, [2]Oncology division, Akdeniz University Medical Faculty, Antalya, 07070, Turkey

We analysed survival in relation both to time to starting treatment (hospital delay) and other clinical parameters in non-small cell lung cancer (NSCLC) patients. All patients diagnosed with NSCLC presenting in 1998 to Norfolk and Norwich Hospital were reviewed (189 patients). Median time to starting treatment in all patients was 48 days. In multivariate analysis, hospital delay did not affect survival in patients in any stage of the disease. Median survival for the whole group was 147 days and by stage 81 days in stage 4, 158 days in stage 3 and not yet reached in stages 1 and 2 combined ($P<0.001$). In stages 1 and 2, referral from GP to chest department as compared with other referral routes was an independent good prognostic indicator ($P=0.032$, HR = 0.08) as was having a surgical resection ($P=0.006$, HR=30.33). In stage 3 patients, laboratory normality (serum calcium <2.6 mmol/L, albumin < or = to 35 g/l, and Hb > or = to 12 g/dl) was a good prognostic indicator ($P=0.002$, HR=0.39). In stage 4 patients the presence of bone or liver metastases as opposed to other metastatic sites was associated with shorter survival ($P=0.005$, HR=2.65). The use of chemotherapy in stage 4 ($P<0.001$, HR=0.16) and in stage 3 the use of both radiation and chemotherapy ($P=0.015$, HR=0.17) were also associated with improved survival (non-randomised). The results suggest that expanding resources in oncology and improving referral pathways are better strategies to improve NSCLC survival than efforts in reducing hospital delay, although both are important.

### P99

**WITHDRAWN**

**P100** REGULATION OF APOPTOSIS BY CD40-LIGAND IN MALIGNANT B-CELLS Claire Dallman, Peter Johnson and Graham Packham, CRC Wessex Medical Oncology Unit, Cancer Sciences Division, University of Southampton, Southampton General Hospital, Southampton, SO16 6YD, UK

CD40 ligation promotes proliferation, immunoglobulin (Ig) production, Ig isotype switching, induction of memory and rescue from apoptosis in normal B-cells. CD40 ligation also induces proliferation and apoptotic rescue in some malignant B-cell lines, and in primary follicular lymphoma, mantle cell lymphoma and chronic lymphocytic leukaemia cultures. It is also apparent that in some cellular settings, CD40 activation paradoxically induces growth arrest and apoptosis.

The ability of CD40 signalling to rescue B-cells from apoptosis has been shown to correlate with expression of anti-apoptotic proteins. Various target proteins have been described, including the Bcl-2 family proteins, Bcl-$X_L$, Mcl-1 and Bfl-1, and A20. However, analysis of these molecules is often restricted to cell lines, and a side-by-side comparison of the extent and kinetics of their induction in various primary lymphoid malignancies has not been performed. The role of pro-apoptotic molecules in CD40 signalling is not known. The aim of this study is to perform a detailed comparison of the expression of CD40 target proteins in low and intermediate grade lymphomas.

We have isolated malignant B-cells from lymph node biopsies of patients with follicular lymphoma and diffuse large B-cell lymphoma. In all samples tested so far, treatment of the cells with soluble CD40 ligand (CD40L) for 48 hours, lead to rescue from etoposide-induced apoptosis and, in some cases, rescue from spontaneous apoptosis. We have prepared protein/RNA from the malignant B-cells with and without CD40L and these materials will be used for analysis of CD40 target molecules.

**P101** PRESENTATION SERUM SELENIUM PREDICTS FOR OVERALL SURVIVAL IN PATIENTS WITH DIFFUSE LARGE B-CELL (DLBC) LYMPHOMA Kim W Last[1], Victoria Cornelius[2], Trevor Delves[3], Jude Fitzgibbon[1], Andy Wilson[1], Ama ZS Rohatiner[1] and T Andrew Lister[1], [1]ICRF Dept. of Medical Oncology, St. Bartholomew's Hospital, London, UK, [2]ICRF Dept of Statistics, Oxford, UK, [3]Southampton General Hospital, Southampton, UK

Epidemiological, laboratory and chemoprevention trial evidence suggests that the essential micronutrient selenium can protect against the development of common malignancies. Selenium can induce apoptosis in cell lines; augment paclitaxel and doxorubicin induced cell death and prevent the development of cisplatin resistance in a mouse model of ovarian cancer. This study was undertaken to test the hypothesis that serum selenium concentration at presentation correlates with outcome in patients undergoing treatment for DLBC lymphoma. The total selenium content was analysed in frozen sera from a hundred patients using inductively coupled plasma mass spectrometry at a dedicated trace elements laboratory. The patients (median age 57 years, range 19–85) presented between July 1986 and March 1999 and received anthracycline-based chemotherapy and/or radiotherapy. Fifty-six patients were male. Twenty-one patients had stage I, 25 stage II, 15 stage III and 39 stage IV disease. B symptoms were present in 15 patients. The serum selenium concentration ranged from 0.33 to 1.51 µmol/l with a median of 0.91 µmol/l (UK adult reference range = 0.8–2.0 µmol/l). Twenty-seven patients had a level below the UK reference range and only 3 patients had a level above the UK median of 1.4 µmol/l. Selenium concentration was not significantly correlated with age, gender, stage or presence of B symptoms. On univariate analysis, age and selenium concentration correlated with overall survival. On multivariate analysis, using a Cox proportional hazard model, selenium concentration, as a continuous variable, was predictive of overall survival (hazard ratio 0.76, 95% CI 0.60, 0.95, $P = 0.018$), but not of response to first therapy. However, the patients in the highest quartile (≥1.11 µmol/l) had a significantly higher CR rate (57% vs. 29%, $\chi^2 = 6.75$, $P = 0.009$). In conclusion, serum selenium concentration at presentation predicts for long-term survival in patients treated for DLBC lymphoma. Prospective validation of these findings will be undertaken.

**P102** THE ASSESSMENT OF IMMUNE CAPACITY IN PATIENTS WITH B CELL MALIGNANCIES H McCarthy, C Ottensmeier, S Chilton, A Duncombe, P Johnson, T Hamblin, F Stevenson, Cancer Sciences Division, Southampton University School of Medicine, Southampton, SO16 6YD

**Background** It is known that patients with B cell malignancies may have impaired immune responses and that chemotherapy may add to this defect.

**Aim** The aim of this study was to assess the integrity of the immune system in patients with B cell malignancies at different stages of their disease and after first, second or third line treatment by measuring the ability to respond to a known recall immunogen (Tetanus Toxoid, TT).

**Method** Three disease categories were investigated as follows: 1) chronic lymphocytic leukaemia (CLL) Binet stage A, untreated, 2) diffuse large cell lymphoma (DLCL) following treatment and 3) follicular lymphoma (FL) following treatment. Cohorts of 14 CLL, 14 DLCL and 11 FL patients were studied. The majority of subjects had previously been vaccinated with TT.

Patients were vaccinated intramuscularly with 0.5 ml TT (Pasteur Merieux). Anti-TT antibody levels were measured by ELISA on serum samples obtained pre-and one month post-vaccination. The TT and WHO human antitoxin standard used in the ELISA were obtained from the National Institute of Biological Standards. The results were compared to those obtained from a cohort of 16 normal healthy volunteers.

**Results** All of the normal volunteers had antibody levels that boosted by at least 2 fold from their baseline values. We therefore assigned those patients who also boosted their baseline levels by 2 fold as responders and those that did not as non-responders. In addition, those patients who failed to boost post-vaccination antibody levels above the recognised protective value of ≥0.1 IU/ml were also counted as non- responders. Response rates were 57% (8/14) in CLL group, 71% (10/14) in DLCL group and 72% (8/11) in the FL group. All three categories responded less well than the control group ($P = >0.05$). The FL and DLCL cohorts were a heterogeneous group who had received diverse treatments. In the DLCL and FL groups, 60% and 50% of responders were in Ist clinical remission respectively. Numbers are currently insufficient for further subgroup analysis.

**Conclusions** The ability to elicit an antibody response to a recall antigen such as TT is dependent on both cellular and humoral arms of the immune system. Remarkably in our patient groups, a high proportion do respond positively to TT. This suggests that the responding groups may be suitable candidates for new treatment strategies or immunotherapy aimed at stimulating the patient's own immune system to combat their B cell malignancies. DNA vaccination using tumour derived idiotypic determinants would be an example of this.

**P103** SUCCESSFUL RADIOIMMUNOTHERAPY (RIT) OF B-CELL LYMPHOMAS DEPENDS ON SYNERGY BETWEEN LOW DOSE RATE IRRADIATION AND ANTIBODY (MAB) Y Du, J Honeychurch, PWM Johnson, TM Illidge, CRC Oncology Department, Cancer Sciences Division, Southampton General Hospital, Southampton, SO16, 6YD, UK

The aims of this study were to investigate the critical mechanisms involved in successful RIT of B cell lymphomas. High dose-rate (HDR-1 Gy/min) and low dose-rate (LDR-0.01 Gy/min) irradiation were compared with and without mAb in vitro and in vivo using murine B lymphoma cell lines that also establish as syngeneic animal models. Cell survival was assessed in vitro by analysing DNA content with Propidium Iodide using flow cytometry, clonogenic assays in vitro and animal survival. HDR and LDR irradiation was delivered using a modified 225 Kv X-ray machine. All of the four cell lines (π-$BCL_1$, $BCL_1$-3B3, EL4 and 38C13) studied demonstrated significantly more apoptosis (1.5–3 fold increase) and decreased clonogenic survival with LDR compared to HDR ($P < 0.01$). These observations were then confirmed in vivo and a dose dependent increase in animal survival was seen with LDR over HDR. We next investigated the role of increasing doses of low dose rate irradiation by using therapeutically inactive mAbs to deliver "systemic radiotherapy" as part of RIT. The $BCL_1$ model was used to investigate a dose response for RIT. Animals were inoculated with $10^5$ tumour cells and RIT was given by intravenous injection between 7–10 days later. A dose dependent increase in tumour protection of around 10 days was seen as the RIT dose of $^{131}$I-labelled anti-Class II mAb was increased from 3.7 to 18.5 MBq per animal. However, when unlabelled anti-Idiotype (surface Ig) or anti-CD40 were added in addition to the 18.5 MBq of $^{131}$I anti-Class II mAb highly significant increases in tumour protection of at least 30 days over that seen in groups treated with 18.5 MBq of $^{131}$I anti-Class II mAb or unlabelled anti-Id alone ($P < 0.001$). For those treated with anti-CD40 and 18.5 MBq of $^{131}$I anti-Class II mAb long-term tumour protection was seen (>150 days). We have demonstrated for the first time in syngeneic animal models that there is a radiation dose response for RIT. Our findings strongly suggest that LDR is more effective than HDR in inducing apoptosis and that antibody and irradiation can be synergistic in vivo. We believe this data provide new insights into our understanding of effective RIT in lymphomas.

### P104 IN VITRO EVIDENCE FOR DIFFERENCES IN 6-MERCAPTOPURINE VERSUS 6-THIOGUANINE METABOLISM DEPENDING ON TPMT STATUS

L Hogarth, M Little*, L Minto, C Redfern, A Hall and S Coulthard, LRF Molecular Pharmacology Group, Medical School, Newcastle University, UK. NE2 4HH

The thiopurine drugs are given as part of continuing therapy in childhood acute lymphoblastic leukaemia (ALL) and are key agents in preventing relapse. Variations in the level of thiopurine methyltransferase (TPMT) activity appear to be a major molecular determinant of the extent of thiopurine metabolism. TPMT cDNA was cloned into an inducible expression vector and transfected into an embryonic kidney cell line, EcR293. TPMT expression was induced in these cells to elucidate sensitivity of these cells to 6-thioguanine (TG) and 6-mercaptopurine (6-MP).

Using SRB assays, for cells treated with 6-TG, the $IC_{50}$ was higher in the induced cells (2.8 μM) compared to the un-induced cells (1.7 μM). Conversely, with the 6-MP treated cells, the $IC_{50}$ was four-fold higher in those with low TPMT activity (8 μM vs. 1.8 μM). Preliminary apoptosis measurements have supported these findings. In TPMT over expressing cells treated with 6TG, less apoptosis was observed than in cells with low TPMT expression. This was in contrast to 6MP treated cells, in which less apoptosis was observed in cells with high TPMT expression compared to low TPMT expression. Measurement of thioguanine nucleotide (TGN) incorporation into DNA showed increased levels of TGNs in TPMT induced cells for both 6TG and 6MP. TGN levels were identical in high and low TPMT expressing cells treated at $IC_{50}$ values for 6TG. However, treatment with $IC_{50}$ values for 6MP showed dramatically reduced incorporation in high TPMT expressing cells. These data suggest that the cytotoxic effect of 6MP is not solely due to the incorporation of fraudulent bases into DNA.

Studies are underway to measure levels of active methylated metabolites produced from 6MP and 6TG in the TPMT transfected cells. These will be correlated along with TGN incorporation into the DNA with cytotoxicity.

This work is supported by The Leukaemia Research Fund.

### P105 MEASUREMENT OF ASPARGINE SYNTHETASE mRNA LEVELS BY REAL-TIME PCR

M Leslie, MC Case, AG Hall, SA Coulthard, POU laboratory, CRU 2nd Floor, Cookson Bldg, Newcastle University, Newcastle, NE2 4HH

L-Asparaginase (L-asp) is a potent chemotherapeutic enzyme and a standard component in the treatment of patients suffering from acute lymphoblastic leukaemia (ALL). However, it has potentially severe side effects, including anaphylactic shock, pancreatitis, liver dysfunction and coagulopathy. Intramuscular injection can be very painful and cause local inflammation. Thus it would be beneficial if treatment could be targeted to patients most likely to respond. A detailed understanding of the molecular pharmacology of this drug *in vivo* will be advantageous in the individualisation of L-asp therapy.

The cytotoxic effect of L-asp results from the complete depletion of asparagine from blood and bone marrow. Leukaemic blasts lack asparagine synthetase (AS) activity unlike normal cells and thus rely on circulating asparagine for cell survival. Resistance to L-asp in vitro is due to increased cellular AS activity, though in vivo this has yet to be confirmed. AS levels may not be consistent between patients, and so there may be a subset of ALL patients expressing higher levels of AS at presentation for which L-asp therapy may be of little or no benefit. Some patients with AML have also shown favourable response to L-asp, however limited results have been published regarding AS expression in these patients.

There is a need for techniques sufficiently sensitive to provide direct evidence to support the hypothesis that L-asp provides a selective cytotoxic effect because of the reduced expression of AS in leukaemic blasts. We have developed a real- time PCR assay to detect AS mRNA levels in the lymphoblasts of patients with acute leukaemia. This newly described technique allows simple and accurate quantitation of mRNA levels over several orders of magnitude using dual labelled fluorogenic hybridisation probes. Real-time quantitative PCR requires the use of standard curves for both an endogenously expressed transcript as a control and a standard curve for unknown transcripts with known dilutions of cDNA template. The control in this case was human transcription binding protein, which is expressed at similar levels in all cells. The normalised AS mRNA levels in ALL patients varied from $0.09 \times 10^{-2}$ to $3.9 \times 10^{-2}$ (median = $0.19 \times 10^{-2}$, n=7). Interestingly, preliminary data extracted from AML patient samples assayed suggests a similar spread. We will present data showing considerable variability in the expression of AS between patients of ALL and AML.

This work is supported a Gordon Pillar Studentship (Leukaemia Research Fund).

### P106 SURVIVAL FROM RECURRENCE OF HODGKIN'S DISEASE: ST BARTHOLOMEW'S HOSPITAL, LONDON

CS Barlow, J Shamash, R Gupta, M Powell, A Wilson, A Norton, AZS Rohatiner, TA Lister Division of Haemato-Oncology and ICRF Medical Oncology Unit, St. Bartholomew's Hospital, London ECIA 7BE

211 patients (m:f 134:77) responding to treatment for Hodgkin's Disease at St. Bartholomew's Hospital between 1968 and 1999 developed recurrent disease and form the basis of this analysis. 48% (102/211) originally had early stage disease, 52% (109/211) being advanced stage. Patients had been treated according to the protocols in place at the time. Outcome according to original treatment had been documented as CR n=144, GPR n=52 and PPR n=15. Six patients died following 1st recurrence without further therapy. 205 were retreated (CT=163, RT=32, CT&RT=10). 82% (168/205) responded to treatment for 1st recurrence, 152 achieving CR or GPR. 19 of these received consolidation with high dose therapy in second remission. 55% of patients remain in continuous second remission. Second recurrence was documented in 62 patients. 57 received a third treatment with 33 responses (CR or PR). The median duration of 3rd remission was 3 years. Overall survival following first recurrence of Hodgkin's Disease is 60% at 5 years and 25% at 30 years. No significant difference was observed between those whose 1st remission was <12 months (58/211) and those for whom it was more than 1 year (153/211). This analysis shows that recurrent Hodgkin's disease is responsive to chemotherapy and that for a few, the long-term outlook is still good. Factors identifying those at risk of recurrence and better treatment for these patients is required.

### P107 p53, mdm2, p21/waf1, bc12 AND bcl-6 PROTEINS EXPRESSION IN NON-HODGKIN'S LYMPHOMAS

SF Al-Othman[1], JR Goepel[2] H Al-Husaini[3], J Martin[4], JA Saad Aldeen[5], Lawry[1], [2]Division of Oncology and Cellular Pathology, [2]Department of Pathology, University of Sheffield, Sheffield, [3]Department of Histopathology & [5]Oncology Department, Riyadh Armed Forces Hospital, Riyadh, K.S.A. [4]Department of Histopathology, King Faisal Specialist Hospital & Research Centre, Riyadh, KSA

**Methods** We have investigated the immunohistochemical expression of p53, p21/waf1, MDM2, bcl-6, and bcl-2 proteins in 3 reactive lymph nodes and 35 cases of non-Hodgkin's Lymphomas (14 follicular, 11 diffuse large B-cell (DLBC) nodular lymphomas and 10 DLBC lymphomas of gastric origin). Samples were scored for staining distribution and intensity using reactive lymph nodes as baseline controls.

**Results** All the reactive lymph nodes studied showed relatively constant labelling for three proteins, p53, MDM2, and p21, namely staining of isolated large germinal centre cells, together with occasional activated lymphocytes in the inter-follicular area. Conversely, bcl-6 expression was restricted to B cells within the germinal centre and rarely in the inter-follicular zone. Bcl-2 proteins, in contrast, showed opposite staining patterns to bcl-6 with expression in the inter- follicular zone, and other follicular components, but rarely within the germinal centres.

In malignant disease bcl-2 expression was identified in 87% (13 of 15) follicular lymphoma yet only half of the DLBC lymphomas studied (41% -nodal cases, 50% -extranodal cases). In contrast, bcl-6 protein expression was only detected in 33% (5 of 15) cases of follicular lymphoma, compared to 63% (12 of 22) cases of DLBC, with increased expression in extranodal cases (70%) compared to nodal cases (42%).

p53 protein expression was presented in 18 of 37 (48.6%) of non-Hodgkin's lymphoma, being more common in high grade (15 of 22, 68%) versus follicular lymphoma (3 of 15, 20%). While p21 and MDM2 were positive in about half of the nodal and gastric non-Hodgkin's Lymphomas, their was greater expression in low grade disease.

**Conclusions** Follicular lymphoma was characterised by the reciprocal expression of bcl-2 and bcl-6.

Comparable staining was seen for p53, MDM2 and p21 between reactive and malignant lymph nodes. However, there was an inverse correlation between p53 and bcl-2 protein expression. As p21 and MDM2 proteins are inducible by wild type (wt) p53, the expression of the three proteins (p53 / p21 / MDM2) may indicate functional p53 pathways in lymphoma and therefore a more favourable clinical response to treatment.

Research was supported by the Kingdom of Saudi Arabia and Yorkshire Cancer Research

### P108 ChlVPP/EVA HYBRID CHEMOTHERAPY FOR PREVIOUSLY UNTREATED HODGKIN'S DISEASE (HD) – A HIGHLY EFFECTIVE ETOPOSIDE CONTAINING REGIMEN WITH A LOW INCIDENCE OF SECONDARY AML/MDS. UPDATED RESULTS FROM CONSECUTIVE RANDOMISED CLINICAL TRIALS INVOLVING 701 PATIENTS

JA Radford[1], AZS Rohatiner[2], WDJ Ryder[1], DPD Deakin[1], T Barbui[4], NP Lucie[3], A Rossi[4], DJ Dunlop[3], RA Cowan[1], PM Wilkinson[1], RK Gupta[2], RD James[1], J Shamash[2], J Chang[1], D Crowther[1], TA Lister[2], [1]Christie Hospital, Manchester, UK, [2]St. Bartholomew's Hospital, London, UK, [3]West of Scotland Lymphoma Group, Glasgow, UK, [4]Ospedali Riuniti, Bergamo, Italy

Since 1984, a total of 701 patients (pts) with previously untreated HD, clinical stages I/II (plus mediastinal bulk and/or B symptom) and clinical stages III/IV have been randomised into two consecutive randomised phase III trials featuring ChlVPP/EVA hybrid chemotherapy (chlorambucil 6mgs/m$^2$ po, procarbazine 90 mg/m$^2$ po, prednisolone 50 mg/m$^2$ po all on days 1–7, etoposide 75–100 mg/m$^2$ po days 1–5, vincristine 1.4 mg/m$^2$ iv day 1, vinblastine 6mgs/m$^2$ iv and Adriamycin 50 mg/m$^2$ iv both on day 8 of a 28 day cycle). Following chemotherapy and a restaging evaluation radiotherapy was given to sites of previous bulk or residual radiographic abnormalities. Median follow-up is now 11 years for trial 1 (versus MVPP, n = 419 pts) and 4.9 years for trial 2 (versus VAPEC-B, n=282 pts) which was closed prematurely at the planned third interim analysis in September 1996.

In trial 1 the previously reported superiority of ChlVPP/EVA over MVPP has been maintained and in trial 2 freedom-from- progression (FFP) event-free-survival (EFS) and overall survival (OS) are all substantially better in the population treated with ChlVPP/EVA for whom the 2 and 5 year results are 87% and 82% (FFP), 85% and 78% (EFS) and 92% and 89% (OS). In both trials, 18 of 355 pts treated with ChlVPP/EVA have developed a second tumour and 11 have died of this complication. In 3 cases the diagnosis was AML/MDS representing an actuarial incidence of 0.7% and 1.4% at 5 and 10 years respectively.

ChlVPP/EVA hybrid is a highly effective chemotherapy for previously untreated Hodgkin's disease and is associated with a low incidence of secondary AML/MDS even after prolonged follow-up. Results will be further updated in April 2001.

### P109 WHAT EFFECT DOES CHEMOTHERAPY HAVE ON LYMPHOCYTE SUBSETS IN HIV RELATED LYMPHOMA?

T Powles, M Nelson, A Mohith BG Gazzard and M Bower, Dept Medical Oncology, Chelsea and Westminster Hospital. SW10 9NH

Chemotherapy causes prolonged suppression of lymphocyte subsets in the HIV negative population; the CD4 count can take up to 1 year to recover in adults. The recover in children is faster due to thymic activity which tails of with age. Other lymphocyte subsets return to normal levels over a shorter period of time. This study was designed to estimate the effect of chemotherapy on immune markers in 20 patients treated with both highly active antiretroviral therapy (HAART) and combination chemotherapy for AIDS related lymphoma (ARL). The T helper (CD4), T-suppressor (CD8), B (CD19) and natural killer cells (CD16 & 54) and HIV m RNA viral load were recorded before, during and up to 6 months after chemotherapy.

The CD4 and natural killer cells fell by 52% and 57% respectively but recovered to pre-treatment levels within 1 month of completing chemotherapy. There was no significant change in the viral load or CD8 count during the study period. B cells fell by >50% and the recovery was slower, taking 3 months.

The recovery of CD4 count was faster than documented in the HIV negative population and in HIV positive individuals not on HAART. We speculate that this recovery is due to up regulation of thymic activity caused by the HAART, which is well documented in the HIV literature. This data also enables us to estimate the degree to which the CD4 count will fall during chemotherapy, and therefore which individuals will require prophylaxis against opportunistic infections.

### P110 A PILOT STUDY TO ASSESS THE FEASIBILITY OF USING 3D DATASETS TO COMPARE RADIATION DOSE TO TARGET TISSUES AND CRITICAL STRUCTURES FROM TWO RADIATION TECHNIQUES

Nicola Thorp, Isabel Syndikus, Elizabeth Richards, Kirstie Brown, Joyce Warren, Helen Mayles, Department of Radiotherapy, Clatterbridge Centre for Oncology, Wirral, Merseyside. CH63 4JY

A technique comprising two modified tangential fields covering the breast (or chest wall) and the axilla has been used at our centre for many years to give adjuvant radiotherapy for early breast cancer. We aim to develop a method of comparing this "monobloc" technique with conventional tangential fields using 3 dimensional (3D) planning datasets. For this initial phase of the study, CT scans were performed on a conventional scanner and the scans were transferred to Nucletron Plato Planning Software. After outlining the relevant anatomical structures, isodose plans were constructed for the two techniques. Integral dose volume histograms (DVH'S) were calculated for target volumes and critical structures and the two sets of mean percentage doses were compared.

Nine patients were scanned for both techniques and one patient each was scanned for the monobloc and conventional tangential technique only. The resulting DVH's were as expected. Both techniques treated the breast (or chest wall) adequately. The monobloc also covered the axillary region but the mean percentage dose to the heart and ipsilateral lung was significantly greater than with conventional tangential portals.

It was necessary to modify the patients' arm positions for computed tomography (CT) scanning due to the limited aperture size. The conventional CT scanner therefore has a limited role in 3D breast planning in view of the constraints on patient position. We have now commenced the next phase of the study comparing the monobloc technique with 3 field techniques using a simulator with CT option which enables the acquisition of CT slices in the actual treatment positions.

### P111 THE USE OF A PRE-TRIAL QUESTIONNAIRE IN DETERMINING RECRUITMENT INTENT IN A STUDY OF SEQUENCING OF CHEMOTHERAPY AND RADIOTHERAPY IN BREAST CANCER

S Bowden[1], I Fernando[2], J Dunn[1] for the SECRAB Steering Committee

[1]CRC Trials Unit, Institute for Cancer Studies, The University of Birmingham, B15 2TT, [2]Cancer Centre, Queen Elizabeth Hospital, Birmingham. B15 2TH

A survey by the Royal College of Radiologists (RCR) in 1993 demonstrated that UK Oncologists were divided on how to sequence adjuvant chemotherapy (CT) and radiotherapy (RT) for the treatment of early breast cancer. 60% of Oncologists were giving CT and RT sequentially (CT→RT or RT→CT) and 40% were using a synchronous schedule (CT→RT→CT).

In 1997 a questionnaire was designed to determine 1) if Oncologists would participate in a trial to determine the optimal sequence of CT and RT; and 2) the standard CT and RT protocols used in the UK. This was sent to 200 Oncologists registered on the CRC Trials Unit database as treating breast cancer. 113 Oncologists replied of which 100 expressed an interest in participating in a trial. 10 different CT regimens were recorded, including CMF, CMF + anthracycline, FEC, AC, CEF, and Sutton MM. CMF was the most popular with 106 (96%) consultants using this alone or with another regimen, most frequently CMF + Anthracycline (31%). 15 different RT schedules were recorded, the most popular being 50 Gy in 25 F (33%), 40 Gy in 15 F (26%), 45 Gy in 20 F (13%), 39 Gy in 13 F (6%) and 46 Gy in 23 F (4%).

These data were used to design a national, phase III clinical trial to address the sequencing question (***SECRAB***). The intention being to randomised 2000 women to a sequential (CT→RT) or a synchronous (CT→RT→CT) schedule. The most frequently used CT and RT regimens were chosen for use in the trial and all consultants who expressed an interest were invited to participate. Recruitment commenced in July 1998. Of the 100 Oncologists who expressed an interest only 29 had taken up the invitation to participate in the trial at the end of January 2001 (a further 19 who did not receive the initial questionnaire were also participating in the study).

The data collected on the questionnaire has been compared to that provided at randomisation. The CT regimen chosen at randomisation (70% CMF; 30% CMF + anthracycline) mirrored the intent indicated on the questionnaire. In contrast the most popular RT schedule chosen at randomisation was 40 Gy in 15 F as opposed to 50 Gy in 25 F indicated on the questionnaire. The proportion of the other RT schedules did however reflect current UK clinical practice as determined by the questionnaire.

The results of this simple questionnaire and the survey performed by the RCR clearly demonstrate the huge variation in the adjuvant treatment of patients with breast cancer in the UK. ***SECRAB*** will determine whether the scheduling of CT and RT has an effect on local recurrence, survival and morbidity of treatment. It is already the largest trial in the world addressing this question.

### P112 AUDIT OF NON-COMPLETION OF RADIOTHERAPY TREATMENT

NG Mikhaeel, S D'Arcy, Mount Vernon Centre for Cancer Treatment, Northwood, Middlesex, UK

**Background & Aim** Demand on radiotherapy treatment is rising while current radiotherapy resources are limited. Most oncology departments across the UK have a waiting list for radiotherapy treatment. It is important to ensure that the current limited resources are optimally used. We conducted a prospective audit in Mount Vernon Centre for Cancer Treatment to assess the incidence of booked treatment courses which were not delivered (not started or started but not completed) and the reasons why.

**Methods** The audit covered a six months period from January to June 2000 inclusive. We kept a record of all patients who did not receive their radiotherapy treatment and who started but did not finish the prescribed course. At the end of the six-month period we reviewed the patients' notes for data collection.

**Results** There were 2169 radiotherapy courses booked in the audit period. 2054 courses (94.7%) were completed. Eighty (3.7%) courses were not started (i.e. cancelled) and 35 (1.6%) were started but not completed. 71% and 69%, in the non-start and non-completion groups respectively, were palliative treatments. 72% & 74% respectively aged more than 60 years. In the non-start group, the most common cause was death (68%). Eighteen percent were "too ill to attend" (TITA) at the time of treatment. In the non-completion group, the most common cause was death (51%) followed by TITA (20%). Disease progression during treatment accounted for 17% (6 patients). Seven patients received 1 out of planned 2 treatments of palliative radiotherapy for lung cancer. Lung cancer was the most common diagnosis in both groups. Comparison with a previous audit in 1997 showed a reduction in the non-completion rate from 2.2% to 1.6% (27% reduction).

**Conclusion** There was a high rate (94.7%) of completion of booked radiotherapy treatment in our centre during the audit period. Most of the undelivered treatments were palliative. There are some inevitable causes e.g. progression of disease during treatment or sudden death from cancer-unrelated causes. Further prospective audit with correlation to treatment waiting time is planned. Current data collection does not allow audit of death within one month of radiotherapy. Low rates of non-completion of treatment are possible and should be aimed for to optimise the use of limited resources.

### P113 NATIONAL AUDIT OF CHEMO-RADIATION PRACTICE IN HEAD AND NECK TUMOURS IN THE UNITED KINGDOM

RSD Brown, M McCormack, M Vilarino-Varela, L Melcher, HA Payne, Meyerstein Institute of Oncology, Middlesex Hospital, Mortimer Street, London WIN 8AA

**Purpose** To conduct a national audit of current chemo-radiation practice in the treatment of head and neck malignancies (as part of a wider survey of chemo-radiation practice for a range of different sites).

**Method** A postal questionnaire sent to all consultant clinical oncologists in the UK (April-July 2000), requesting details of current chemo-radiation practice. Consultants treating head and neck cancers were asked to provide details of subsites, dose-fractionation and chemotherapy schedules used.

**Results** Overall response rate to the questionnaire was 311/385 (81%). Sixty-one out of 82 (74%) of those treating head and neck cancer used chemotherapy and radiotherapy, with 52/61 (85%) using concurrent chemoradiotherapy. The commonest subsites treated with chemo-radiation were nasopharynx 30/61 (49%) and larynx 11/61 (18%). There was wide variation in total radiation dose and fractionation and in the choice of chemotherapy drugs, dose and scheduling for all sites treated. Twenty-six different radiotherapy regimes and 40 different chemotherapy regimes were used for treating head and neck cancer. The most frequently used radiotherapy schedule was 66 Gray in 33 fractions (18/60, 30%). The most commonly used chemotherapy was cisplatin, either alone (18/52, 35%) or in combination with 5-fluorouracil (22/52, 42%). However within each of these categories there were many different schedules. Detailed analysis of these results is presented.

**Conclusions** There is a wide variation in chemo-radiation schedules in current use for the treatment of head and neck cancer in the UK. This may reflect the lack of national guidelines, conflicting evidence from randomised controlled trials and a desire by oncologists to minimize toxicity.

### P114 IMPACT OF LUNG CANCER MULTIDISCIPLINARY TEAM MEETING (MDT) ON RATES OF HISTOLOGICAL DIAGNOSIS AND RADICAL THERAPY

CJ Ferguson[1], TK Rogers[2], S Rogers[2] and MQ Hatton[1], [1]Weston Park Hospital, Whitham Road, Sheffield, S10 2SJ, [2]Doncaster Royal Infirmary, Armthorpe Road, Doncaster, DN2 5LT

**Aim** To see if our lung cancer MDT had improved the rate of histological diagnosis and/or the proportion of patients referred for radical therapy.

**Introduction** In Doncaster prior to 1997 no MDT lung cancer meetings occurred, referral to an oncologist or cardiothoracic surgeon was at the discretion of the respiratory physicians. An audit of lung cancer patients was carried out in summer 1997. Since October 1997 weekly meetings have occurred supported by a site-specialised oncologist and thoracic surgeon.

**Methods** The 1997 audit comprised a prospective evaluation of 63 consecutive lung cancer patients, the "pre-MDT patients".

Between 1st October 1997 and 1st July 2000 599 incident cases of lung cancer have been discussed at the MDT meeting, the "MDT patients". Details of all patients discussed have been recorded prospectively.

Proportions were compared for significance using $_x2$ or Fischer's exact test.

**Results** There has been a trend towards MDT patients being older (59% of MDT patients vs 51% of pre-MDT over 70) and less fit (13% vs 1.5% ($P$<0.005) inoperable due to poor performance status).

There has been an increase in the histological diagnosis rate (77% MDT patients vs 65% pre-MDT).

There has been a reduction both in referral for thoracotomy (10.3% MDT patients vs 17.4% pre-MDT) and in successful thoracotomy rates (8.2% vs 12.7%).

Rates of referral for radical radiotherapy are unchanged (3%).

**Conclusion** Despite an increased rate of histological diagnosis this study shows no improvement in the rate of radical therapy as a result of the MDT.

Analysis of the effectiveness of the MDT may be complicated by changing referral patterns.

It will therefore be important to compare survival rates to avoid this potential bias.

### P115 TWO YEAR RESULTS OF A MULTI-DISCIPLINARY LUNG CANCER SERVICE

SG Russell, K Waite, L Magee, D Gilligan, Thoracic Oncology Unit, Addenbrooke's and Papworth NHS Trusts, Cambridge. CB2 2QQ

**Introduction** The improvement of cancer care in the UK currently has a high profile. The Calman-Hine recommendations and the NHS Cancer Plan aim to improve and provide equitable high quality care to those diagnosed with cancer. This abstract reports the 2 year results of a well established multidisciplinary team (MDT) for lung cancer. Patients suspected of having lung cancer attend the service on one day for a series of programmed diagnostic investigations. They also undergo an initial specialist nurse assessment to identify psychological or social problems likely to require early intervention. The results are discussed within the same week at a multidisciplinary meeting and a management plan made along agreed guidelines.

**Patients/Methods** During 1998 306 new patients were seen in the clinic of which 211 (69%) had lung cancer, of which 201 were histologically proven (95%). 131 were male (65%) and 70 female (35%). The mean age was 72 (range 40–91). 29 small cell (14%), 172 Non-small cell (NSCLC) (86%). Of the NSCLC 34 had a surgical resection (20%). The other modalities of treatment were radiotherapy 105 patients (61%) and chemotherapy 23 (13%). The staging was as follows 1–27 patients (16%), 11–24 (14%), IIIa-37 (22%), IIIb-28 (17%) and IV-49 (30%), 7 patients were not formally staged. There were 29 Small cell cancers, 15 were limited and 14 extensive stage. Treatment was with radiotherapy 7 patients, Chemotherapy 9 and both in 11.31% of patients were entered into clinical trials of which 19% were randomised controlled trials. The main outcome was survival and data was gathered for all patients, who were followed until death or at least 2 years.

**Results** The table below shows the overall NSCLC survival by stage.

| Stage | 1 Year | 2 Year |
|---|---|---|
| I | 70% | 48% |
| II | 58% | 50% |
| IIIa | 40% | 27% |
| IIIb | 28% | 21% |
| IV | 12% | 0% |

Of the 35 patients who had a resection the median survival by stage in all stages has not yet been reached with a median follow up of 30.7 months. In those with small cell lung cancer the median survival in limited stage disease was 13.2 months and 2.8 months in extensive stage disease.

**Conclusions** We believe these are the first published survival figures from a comprehensive lung cancer service in the UK. These results will be compared to other published series and discussed in context with Cancer Registry Data.

### P116 INTERNATIONAL VARIATION IN PALLIATIVE RADIOTHERAPY REGIMENS FOR LUNG CANCER: DOES EVIDENCE MATTER? A QUESTIONNAIRE SURVEY

Elizabeth Toy, Fergus Macbeth, Velindre Hospital, Cardiff, UK. CF14 2TL

**Background** Previous surveys have shown widespread variation in radiotherapy practice in the palliative treatment of lung cancer. Nine randomised controlled trials and a number of clinical guidelines have been published in the last 15 years. This study was designed to assess the impact of this evidence on clinical practice.

**Method** A systematic review of randomised trials of palliative radiotherapy in NSCLC was presented at three international conferences (ECCO 1999, 2nd Consensus Workshop in Palliative Radiotherapy and Symptom Control 2000 and The World Lung Cancer Conference 2000). A questionnaire was distributed to members of the audience who were invited to answer questions about their management of two hypothetical patients (one with symptoms and poor performance status (PS) and one asymptomatic with good PS) and about their response to the evidence presented.

**Results** 110 questionnaires were returned, with respondents from 24 countries in Europe, North America, Africa, Australia and the Far East. For the two cases a total of 49 different regimens were recommended (24 for case 1, 34 for case 2) with doses ranging from 8Gy/IF to 70 Gy/35 F. The most frequent reason for the choice of regimen (51%) was "departmental policy". Research findings were mentioned by 39%. A minority of respondents (17%) would change practice in the light of the evidence presented.

**Conclusions** There is still widespread practice variation in palliative radiotherapy and an apparent reluctance to change even in the light of good research evidence.

### P117 RADICAL RADIOTHERAPY FOR NON-SMALL CELL LUNG CANCER, OUTCOMES AND DELAYS IN TREATMENT – THE CAMBRIDGE EXPERIENCE 1996–99

KJ Waite, SG Russell, L Magee and D Gilligan. Thoracic Oncology Unit, Addenbrooke's Hospital, Cambridge. CB2 2QQ and Papworth Hospital NHS Trust

**Introduction** Radical radiotherapy has been shown to cure a proportion of patients with non-small cell lung cancer (NSCLC) who are considered to be medically unfit for surgery or who refuse surgery. Delays in commencing treatment have also recently been shown to be important. We present the results of our patients with NSCLC who have been treated radically with radiotherapy between 1996 and 1999.

**Patients/Methods** 53 patients with NSCLC, who were considered unfit or who refused surgery, were treated between 1996–99 with radical radiotherapy. Patients who were treated with chemo/radiotherapy have been excluded from this review. 38 were male and 15 were female. The mean age at diagnosis of this group was 71 years, (range 54–82 years). The performance status of these patients was as follows: 7 were P.S. 0, 30 P.S. 1, 10 P.S. 2, 1 P.S. 3 and the performance status was not recorded in the remaining 5 patient notes. 96% of patients had histologically proven disease. 37 had squamous cell carcinoma, 7 had adenocarcinoma, and 7 had undifferentiated lesions. All had CT planning of their radiotherapy and most patients received a dose to the tumour of 55Gy in 20 fractions daily, using 5–16 mV photons, (2 had 60Gy in 30 fractions, 1 had 64Gy in 32 fractions and 1 40Gy in 15 fractions). The mediastinum was not irradiated prophylactically. The median time between requesting radiotherapy and commencing treatment was 46 days (range 11 to 84 days). Two patients were treated with chemotherapy either immediately after radiotherapy or at the time of disease recurrence. There was a mean interruption during the course of radiotherapy of 1.5 days (range 0–6 days). All patients have had a minimum of 1-year follow up.

The overall one-year survival for this group of patients was 66.0% (35/53) and the median survival was 630 days (20.8 months, 95% CI 454–916 days). Amongst T1/2 patients the one-year survival was 62.5% (25/40) and the median survival was 630 days (20.8 months, 95% CI 353–903 days). This compares with a one-year survival of 76.9% (10/13) and median survival of 580 days (19.2 months, 95% CI 454–1124 days) for those patients with T3/T4 lesions. There was no statistical difference in survival between the T1/2 lesions and T3/4 lesions on the Log Rank Test ($P = 0.63$).

**Conclusion** Our results for patients with NSCLC who were considered to be medically inoperable are very similar to other published series. There was no statistical difference in survival between patients with T1/2 lesions compared with T3/4 lesions.

### P118 POOREST PROGNOSIS SMALL CELL LUNG CANCER (SCLC): TO TREAT OR NOT TO TREAT?

HE Innes[1], P Stockton[2], J Hendry[3], MJ Walshaw[2] and E Marshall[1], [1]Clatterbridge Centre for Oncology, Merseyside CH63 4JY, [2]The Cardiothoracic Centre, Merseyside L14 3PE and [3]Whiston Hospital, Prescot L35 3QY

Overall survival in SCLC remains disappointing. Even in those with the most favourable prognostic factors treated with dose-intense combination chemotherapy there are few survivors beyond 2 years. Many patients present with at least one adverse prognostic factor and for these patients the outlook is considerably worse. In a large MRC study (1) of patients with performance status (PS) ≥2 those treated with combination CAV chemotherapy had median overall survival of approximately 6 months. For those in the worst prognosis group the role of chemotherapy is less well defined: in the MRC study only 38% were PS 3 or 4 and just over half had extensive stage disease (57%).

We have retrospectively reviewed the characteristics and outcome of all patients with SCLC who were referred to a Medical Oncologist in central Liverpool between October 1997 and June 2000. A total of 142 patients were referred: almost a third (31%, 42) were PS 3 or 4, (37 PS3, 7 PS4) with a median age of 70y, (51 to 83y). Of these, 37 (84.1%) had extensive disease and only 7 (15.9%) had limited disease and the great majority (>90%) had significant co-morbidity. 20 patients either refused or were considered ineligible for chemotherapy and received supportive care. Only 1 patient fulfilled entry criteria for a clinical trial. Of the 24 who received chemotherapy this consisted of CAV (13), ACE (2), oral etoposide (8) or weekly chemotherapy (1). 58% had partial or complete responses, 17% had progressive disease and 25% were not assessable for response. Median survival in the supportive care group was 3 weeks and 12 weeks in the chemotherapy group. 71%, 48% and 26% of patients were alive at 1, 3 and 6 months respectively. Only 5 patients (21%) survived >40 weeks, of these 3 had presented with acute deterioration in PS due to SVCO, hyponatracmia and post surgery. In conclusion, a significant proportion of SCLC patients present with very poor performance status, extensive disease and significant co-morbidity. Few of these are currently eligible for clinical trials. For these patients prognosis is dismal despite chemotherapy. Future clinical trials should focus on quality of life issues, for example shorter, less toxic chemotherapy regimens.

1. MRC Lung Cancer Working Party, Lancet (1996) **348** 563

### P119 OPEN ACCESS FOLLOW-UP FOR LUNG CANCER – PATIENT AND STAFF SATISFACTION

AM Henry[1], J Joseph[1], JW Adlard[1], CV Brammer[2], GE Gerrard[1], [1]Yorkshire Regional Centre for Cancer Treatment, Cookridge Hospital, Leeds and [2]New Cross Hospital, Wolverhampton

**Background** Most lung cancer patients have incurable disease and treatments offered are aimed at palliation. Rather than have regular follow-up at the hospital, a pilot 'open access' lung cancer clinic has been set up. Patients are discharged to the community once initial palliative treatment has been completed and review at the 'open access' clinic can be arranged at short notice if needed. The outcomes and level of satisfaction of patients, their relatives and staff to this method of follow-up have been assessed.

**Methods** Between June 1997 and June 1999, all new patients who attended the oncology clinic at Halifax Royal Infirmary with primary thoracic malignancy were identified. Patients who received palliative radiotherapy and/or chemotherapy were given a routine out-patient follow-up appointment six weeks later. Those who attended this appointment were identified prospectively and offered further follow-up in the open access clinic. The care of patients who consented to 'open access' was transferred back to primary care. In January 2000, questionnaires were sent to those patients who were still alive and the next of kin of those who had died. Separate questionnaires were also sent to the local Macmillan nurses and the clinic nurses. For patients with problems, the patient's GP was contacted. Questionnaires were completed and analysed anonymously.

**Findings** 160 new patients attended in the period. Of these, 122 patients had palliative treatment or had no symptoms requiring intervention. 75 out of 122 attended at six-week follow-up. 65 patients agreed to open access follow-up. During the study period, 28 of the 65 patients had one open access visit, 10 patients had two visits and 6 patients had three visits. Therefore, there were a total of 66 visits made by 44 open access patients. 50% of patients were seen within 4 days of requesting an appointment (range 0.5 to 13 days). In January 2000 questionnaires were distributed to 59 of the possible 65 patients or their next of kin. 40 were returned (68% response rate). 98% of respondents felt care given was either good or excellent. 78% preferred open access to routine follow-up. All Macmillan nurses, clinic nurses and GPs responded. Nurses felt that 90% of patients knew how to make an appointment and had good support. GPs also favoured the open access follow-up system for patients who could cope with it.

**Interpretation** The outcomes and level of satisfaction of patients, their relatives and staff to this method of follow-up were found to be positive. Open access follow-up may be useful for many patients following completion of initial palliative treatment.

### P120 THE POTENTIAL DEMAND FOR CHEMOTHERAPY IN ENGLAND AND WALES: POPULATION-BASED CALCULATIONS AND ESTIMATES

MH Cullen[1], and M Muers[2], [1]Cancer Centre at the Queen Elizabeth Hospital, Birmingham, B15 2TH, [2]Killingbeck Hospital, Leeds, LS14 7UQ

**Introduction** It is well known that, relative to most other developed countries, the UK is extremely short of oncologists, particularly medical oncologists. A shortage of specialists was cited as a factor contributing to poor cancer survival in the UK, in the Eurocare study. The last decade has seen a huge increase in indications for chemotherapy (CT), with wide regional variations in usage. This work sets out to estimate the number of adult cancer patients in England and Wales (E&W) presenting annually for whom CT may be considered at least once during their disease, to help inform the National Cancer Plan implementation.

**Methods** Data on efficacy were obtained from randomised trials and meta-analyses; tumour incidence and age distribution figures from cancer registry statistics; other key determinants for chemotherapy like stage, performance status (PS), grade etc from unselected, prospective population-based data where available, and on estimates where population-based data are not available (eg for PS in most tumours). Rare cancers, those likely to amount to fewer than 600 cases annually for CT, paediatric cancers, leukaemias and myeloma are omitted and, *no* account is taken of need for CT at more than one stage (eg adjuvant, first line advanced, second line advanced etc).

**Results** There are over 37000 cases of lung cancer annually in E&W. Using population-based data from the Yorkshire Lung Cancer Referral Patterns audit of 1999, plus PS data from the RCP lung cancer bronchoscopy audit of 1680 patients (1999), we calculate that 61% of patients with SCLC (2733) are eligible for consideration for CT. This is the same as the figure reported for CT usage in Scotland and Yorkshire for SCLC, supporting the validity of the model. For NSCLC the model suggests 50% of cases (16336) eligible for chemotherapy. This compares with an actual usage varying from <5–22.5% in different areas. For colorectal cancer 11804 cases (41% of the total) are eligible for CT, and for breast cancer 15470 (48% of the total) should receive CT at one or more stages in their disease. For stomach, pancreas, oesophagus, ovarian, and testicular cancers, lymphomas, and melanoma a total of 17482 cases should be offered chemotherapy (44% of the total)

**Conclusions** For the commoner tumours where CT has an established place, 46% of patients (63825 per annum in E&W) are eligible for CT at least once during the illness. Resources for the delivery of chemotherapy need to expand urgently to accommodate this growing demand safely and effectively.

### P121 ATTITUDES TO CHEMOTHERAPY FOR THE OLDER PATIENT WITH CANCER

AE Brewster, P Hackett, TS Maughan, S O'Mahoney, L Branston, P Price, I Robbe, D Adams-Jones, R Cannings, V Morrison, D Cohen, On behalf of the Welsh Cancer Care for the Elderly Research Group. Velindre Hospital. Cardiff. CF14 2TL

**Background** Evidence suggests that older patients are under investigated and under-treated and experience worse outcomes than younger patients (1). Clinicians are unsure about offering chemotherapy to older patients because of uncertainty about whether the likely benefits in terms of survival and QOL are sufficiently worthwhile to an older person when balanced against likely toxicity and costs of treatment.

**Methods** A questionnaire has been developed, piloted and psychometrically validated to try and identify social attitudes towards cancer treatment for the older patient. 1000 questionnaires containing 24 individual and multidimensional questions were sent to a random sample of the general public.

**Results** The response rate, 17%, was low but analyses demonstrated 1). the reliability of the questionnaire 2). clear attitudes to the use of chemotherapy with respect to potential benefit and 3). that respondents understood the complex multivariable treatment scenarios. Although age modified response, QOL and survival benefits remained the overriding consideration. Over 95% of respondents felt that chemotherapy should be considered for patients aged 56 or less but 71.6% felt that it should still be considered even at age 79. 74.6% considered that a survival benefit of 6 months or more, as seen with palliative colorectal or breast cancer, was worthwhile. Survival benefits of between 1 and 10% (as seen with many adjuvant therapies) produced a wide range of responses suggesting that clinicians cannot assume how useful an individual may perceive a treatment. It is now our intention to send the questionnaire to a random sample of health professionals to compare and contrast attitudes.

1. Vercelli M, Quaglia A, Casella C, *et al*. Relative survival in elderly cancer patients in Europe. EJC 1998. **34** 2264–2270

### P122 RECRUITMENT OF PATIENTS TO CLINICAL TRIALS: SIZE OF POPULATION REPRESENTED

S Michael Crawford, Janet Peace, Dept of Medical Oncology, Airedale General Hospital, Skipton Road Steeton, Keighley, West Yorkshire BD20 6TD

Large randomised clinical trials are accepted as the best way of answering questions about the relative value of different approaches management and treatment. Recruitment of sufficient patients to a large trial can take several years, which may mean that the question is considered less relevant by the time the last patients are entered.

Airedale General Hospital is the venue of care for the overwhelming majority of residents of its catchment area. Since 1996, the medical oncology service has adopted entry into the NEAT trial as standard policy for adjuvant chemotherapy for eligible patients with breast cancer. Between 1995 and 1998 the QUASAR study was the policy for colorectal cancer. We have used this population base to estimate the size of population needed to recruit 1000 trial subjects per year when the policy for that population is that eligible patients are entered. Patients with non-small cell lung cancer (NSCLC) were entered into the Big Lung Trial (BLT) and those with small cell (SCLC) into various regional studies. These were mainly patients with advanced disease and at the time proportionally fewer incident cases were diagnosed in the hospital.

The average recruitment was NEAT was 15 patients per annum, QUASAR 11 pa. BLT 11 pa, SCLC 10 pa. The catchment population was derived using Northern and Yorkshire Cancer Registry population statistics as follows:

| Number of histological diagnoses (1998) | | Histology rate | Grossed-up cases | Crude annual incidence | Population represented |
|---|---|---|---|---|---|
| Breast | 147 | 0.88 | 167.0 | 55.9 | 298 829 |
| Colorectal | 124 | 0.86 | 143.7 | 55.1 | 260 771 |
| NSCLC | 54 | 0.64 | 84.4 | 60 | 140 625 |
| SCLC | 14 | 0.64 | 21.9 | 15 | 145 833 |

From this the required population to recruit 1000 cases per annum for adjuvant chemotherapy is 19.92 million for breast cancer and 23.71 million for colorectal cancer. For chemotherapy of advanced lung cancer the required population to recruit 1000 cases per annum is 14.59 million (SCLC) and 12.78 million (NSCLC).

These observations indicate the magnitude of the task to make the culture of clinical trials penetrate cancer services in the United Kingdom.

### P123 MALIGNANT CEREBRAL GLIOMA: IMPROVING THE CARE OF PATIENTS AND THEIR FAMILIES

AM Parr[1] and HR Gattamaneni[2], [1]SpR in Palliative Medicine, [2]Consultant in Clinical Oncology, Christie Hospital, Manchester M20 4BX

We interviewed 35 patients with malignant cerebral glioma attending the Christie Hospital regarding their difficulties, satisfaction with care and ideas for improving the service. Carers, who accompanied 29 patients, were also invited to comment. The median time from diagnosis was 20 months. 43% of patients had relapsed disease. The majority of patients were satisfied overall with their care and the level of support offered by the hospital. Crises had generally been handled well.

**Sources of distress identified**

1. Complex physical, social and psychological problems.
2. Relatively infrequent and late input from occupational therapy, physiotherapy, psychological support services and social workers.
3. Inability to see the same doctor at each clinic visit.
4. Insufficient communication between the various healthcare professionals involved resulting in the need for patients to repeat information.
5. Desire for more information and more time to discuss anxieties and difficulties.
6. Insensitive delivery of information by some doctors.
7. Concern about disturbing doctors for advice between clinic visits despite having been encouraged to do so if necessary.
8. Uncertainty about the availability of support from other services and organisations.
9. Delays preceding investigation or treatment.

**Implications for practice**

1. Staff should appreciate the wide-ranging impact of brain tumours on patients and their families/carers and be proactive in enquiring about difficulties in order to facilitate early referral to rehabilitation and support services and organisations. Expert psychological support should be readily accessible.
2. Communication skills training is essential for professionals involved in the care of cancer patients. Time needs to be dedicated to listening and to giving information. A manned information centre with internet access has recently been set up within the hospital to improve the availability of information to patients about all aspects of their disease, its management and sources of help and support.
3. Care must be well co-ordinated and continuous, with adequate communication between professionals. We devised a sheet to be placed prominently in each patient's notes to record dates of disclosure of key information and referral to other services/ professionals in the hope that it would assist this process.
4. A nurse specialist could potentially help to bridge gaps in the service, promote better pre-planning and continuity of care, minimise delays and improve liaison.

### P124 AUDIT OF THE USE OF IV PALLIATIVE CHEMOTHERAPY IN CANCER CENTRE AND LINKED CANCER UNITS

A Mayer, M Ward, R Benson, M Moody, Cancer Centre, Addenbrooke's NHS Trust, Cambridge CB2 2QQ

An audit was performed to ascertain that palliative IV chemotherapy was given appropriately and according to departmental guidelines laid down in a consensus meeting in January 1999. These require a potential survival or quality of life gain. Documentation of measurable disease (clinical, radiological or biochemical), performance status (PS), symptoms and the maximum number of cycles to be given was required at outset, documentation of PS, symptoms and toxicity at each cycle and an interim assessment after 3 cycles. A previous audit documented that second line palliative chemotherapy prescription was evidence-based, but that documentation of performance status and toxicity was insufficient.

127 randomly selected notes of non-trial patients (10 per consultant) who presented for their first cycle of chemotherapy on or after 1.4.99 were audited. 4 consultants practise at Addenbrooke's Hospital and 9 consultants at cancer units (Bedford, Bury St Edmunds, Huntingdon, King's Lynn, Peterborough). 21% of the patients had received prior chemotherapy. The maximum number of cycles to be given was stated in 38% prior to starting treatment. In 95% the evaluable disease was documented, clinically in 65%, radiologically in 88%, biochemically in 38%. Symptoms and performance status before chemotherapy were documented in 83% and 23%, respectively. At each of 559 cycles symptoms were documented in 45%, PS in 14% and chemotherapy related toxicity in 62%. Interim assessment after 2–3 cycles as required by departmental guidelines was carried out in 76%, however 17% were not evaluable in this respect because they had received less than 3 cycles at the time of evaluation. Appropriate 'stopping rules' were applied, i.e. treatment was never continued in spite of disease progression and lack of symptomatic benefit. 66% of the evaluable patients continued treatment, 63% had had no evidence of disease progression, 49% reported symptomatic benefit, 3% of those patients had signs of disease progression.

In summary patients received chemotherapy appropriately, documentation of measureable disease was satisfactory and an interim assessment was performed as required. However documentation of PS, symptoms and toxicity should be improved. The extended use of flowcharts is currently being implemented, a reaudit will be performed after appropriate changes have been made.

### P125 WHAT CONCERNS DO ONCOLOGISTS EXPRESS ABOUT SPECIALIST PALLIATIVE CARE?

AM Parr[1], WP Makin[2], [1]SpR in Palliative Medicine, [2]Macmillan Consultant in Clinical Oncology and Palliative Medicine, Christie Hospital, Manchester M20 4BX

Although most oncologists appear to appreciate the value of palliative care for their patients, many do not yet seem able to place their full trust in palliative care services and therefore reserve referral for those with far advanced disease. In order to explore why this might be, all doctors working at the Christie Hospital were invited to anonymously complete a questionnaire regarding their perceptions of specialist palliative care. 49 doctors (54%) replied, with no significant difference in response rates between grades, sub-specialties or sex. Several factors that might contribute to reluctance to use palliative care services at an earlier stage were highlighted:

1. The failure of some oncologists to fully understand the goals of palliative care.
2. A lack of appreciation of the range of investigations, procedures and interventions that might be offered in some hospices.
3. Fears of inadequate liaison with oncology services by palliative care specialists and consequent loss of control over patient care.
4. Anxiety about hospice staff managing their patients inappropriately through insufficient expertise, reluctance for active investigation and intervention, inadequate provision of nutritional support and excessive use of sedation.
5. A suspicion that palliative care professionals are generally less proficient than oncologists in discussing the pros and cons of further anti-cancer therapy and may tend more towards negotiating the discontinuation of treatment.
6. Recognition of patient apprehension and a concern that patients would perceive referral to palliative care services as an indication of the end of active treatment, loss of all hope and impending death.
7. The paucity of evidence for many of the management approaches used in palliative care.
8. Problems with the accessibility and availability of hospice care.
9. A belief that development of palliative care services would divert resources away from oncological research and practice.

Early referral to palliative care should be viewed as a positive step contributing to optimal symptom control and quality of life throughout a patient's illness. Improving liaison between oncology and palliative care would help to ensure safe, continuous care for patients with palliative care needs no matter what the stage of their disease. Better communication would also guide appropriate decision-making, facilitate learning, promote a mutual understanding, foster ideas for joint research and should alleviate some of the oncologists' anxieties. Work to address the evidence base in palliative care and inequalities in access to services is underway.

### P126 CAN THE NHS CANCER PLAN TARGET FOR WAITING TIMES BE ACHIEVED IN RECTAL CANCER?

P Chung, S Khanduri, S Sothi and RJ Grieve, Walsgrave Cancer Centre, Coventry CV2 2DX, UK

The NHS Cancer Plan states that all patients with cancer should start treatment within 28 days of diagnosis by the year 2005. We performed a retrospective audit at our centre to determine the time from diagnosis to treatment for all patients with rectal cancers who underwent curative surgery. From January 1997 to March 2000, 154 such patients were identified from databases in the oncology, pathology and radiology departments. The dates of diagnosis (date rectal biopsy taken), staging investigations, oncology appointment, start of radiotherapy and surgery were obtained.

Median time from diagnosis to surgery for all patients was 33 days (IQR 21 to 52 days). 37% of patients had surgery within 28 days of diagnosis. Median time from diagnosis to imaging request for ultrasound, CT scan, MRI scan and barium enema were 5, 11, 14 and 7 days respectively. Median time from imaging request to scan for ultrasound, CT scan, MRI scan and barium enema were 6, 10, 12 and 7 days respectively. Median time from diagnosis to last investigation was 17 days and from last investigation to surgery was 18 days. Short course pre-operative radiotherapy did not significantly prolong time to surgery (median 36 days, IQR 27 to 51 days). Median time from diagnosis to first oncology appointment was 14 days (IQR 8 to 20 days), from first oncology appointment to start of radiotherapy was 13 days (IQR 10 to 20) and from start of radiotherapy to surgery was 8 days (IQR 7 to 10 days).

The recommended time from diagnosis to surgery of 28 days was achieved in only a third of patients. This can be improved by organisational change using fast tracking within the multidisciplinary team with pre-scheduling of diagnostic investigations, which will reduce time from diagnosis to completion of staging investigations. Whilst this will improve time to treatment it will be at the expense of longer waiting times for routine requests. The most serious delay would still remain the time from the conclusion of investigations to surgery. This can only be alleviated by an increase in resources to reduce waiting lists for operation. With existing resources the NHS Cancer Plan waiting time targets for rectal cancer cannot be achieved in our centre. Any plan for reform can only succeed with substantial investment.

### P127 ANXIETY IN CANCER PATIENTS.

D Stark[1], M Kiely[1], A Smith[1], P Selby[1] and A House[2], [1]ICRF Cancer Medicine Research Unit, St James's Hospital, Leeds, [2]Dept. of Psychiatry and Behavioural Sciences, University of Leeds, UK

Cancer patients often report anxiety (A), but morbid anxiety is poorly defined in this setting. Recognition (and hence treatment) can be inadequate, and might be improved by examination of the problems inherent in questionnaires and clinical diagnosis.

**Aims** Describe the extent and nature of A. Compare self-reported (SRA) and psychiatric A. Examine the relation of A to cancer specific Quality of Life.

**Method Subjects** 178 out-patients with renal cell carcinoma, malignant melanoma, or a lymphoma. *Measures: 1*. Questionnaires, using touch-screen monitors; QLQ-C-30, HADS, and Close Persons Questionnaire (validated measure of social support). *2*. Psychiatric interview. *3*. Cancer histories from case note review.

**Results Extent** At cut-off of > 7/21 on the HADS A scale 85/178 had SRA. 26/85 fulfilled ICD-10 criteria for anxiety disorder (AD). 6/93 without SRA fulfilled criteria for AD. 27/85 with SRA experienced somatic symptoms of A but insufficient for psychiatric diagnosis. 32/85 with SRA reported no somatic A at interview. These observations are examined using the tripartite model of mood disorders, separating somatic anxiety from negative affect.

**Nature AD** Paroxysmal/Panic:7 subjects, situational/phobic:12, free-floating/generalised:3, mixed:7, organic:2, adjustment:1. *SRA not AD*: Panic type 2, free floating type 10, phobia type 8, mixture 7.

**Onset AD** 31% at ≥ 6 months before cancer diagnosis, 38% within 6 months before or after, and 31% at > 6 months after diagnosis.

**Associations** In regression analyses, both SRA and AD are independently associated with gender, performance status, negative aspects of social support, depression and past psychiatric history. *Quality of Life*. AD is associated with impaired emotional functioning, and insomnia when controlled for gender, extent of disease and depression. Explanations for this are examined from a cognitive model.

**Conclusions** A is frequently reported. Using the HAD-A scale, only 31% of SRA is sufficient to fulfil psychiatric criteria. This scale may not to discriminate somatic A from distress. AD by psychiatric criteria is often situational, but lesser degrees of free floating anxiety are prevalent. 1/3 of anxiety disorders predate cancer diagnosis by > 6 months. Both SRA and AD are much less associated with events such as cancer progression or treatment than with psychosocial variables and performance status. AD is associated with a quality of life deficit, which could contribute to its recognition. Cohort studies are ongoing to examine causation, prediction, and consequences of SRA and AD, and their impact on doctor-patient communication.

### P128 DEVELOPMENT OF A SOCIAL PROBLEMS SCREENING INSTRUMENT (SPSI) FOR USE IN ONCOLOGY CLINICS

EP Wright[1], M Kiely[1], A Cull[2], PJ Selby[1], [1]ICRF Cancer Medicine Research Unit, St James's University Hospital, Leeds LS9 7TF, [2]Imperial Cancer Research Fund, Medical Oncology Unit, Western General Hospital, Edinburgh, UK

**Aim** This research project seeks to explore the social problems and concerns of cancer patients with particular reference to the clinically useful ways of measuring and evaluating them. While the existence of social problems has been recognized, there is little information available concerning their prevalence and intensity or about how they should be managed in routine practice. These studies aim to demonstrate that screening for social problems in oncology practice is possible by presenting a specifically developed social problems screening instrument via a computer touchscreen to patients in cancer clinics.

**Item generation method** Items were generated independently for possible inclusion on to the questionnaire from 3 sources, 1.) a review of the literature and existing questionnaires 2.) oncology patients 3.) health and welfare staff in oncology. Items from all sources were coded and the lists pooled. The pooled list was reviewed and reduced in length by consensus of 6 oncology health and welfare experts to eliminate overlapping items and item redundancy.

**Results** One hundred and eleven articles and 10 questionnaires were reviewed with 51 items generated. Forty-nine staff participated in 7 face-to-face focus groups generating 39 items. Ninety six patients participated in 16 telephone focus groups, 2 face-to-face focus groups, 12 in-depth interviews resulting in 49 generated items. Items generated were grouped under broad social problem themes: in the home, health and welfare services, employment, legal issues, relationships, self image and recreation. Single items included housing, mobility, discrimination and isolation. The pooled item total was 61 which was reduced to 28 items.

**Questionnaire design method** The reduced item list was used as the framework for a 28 item questionnaire. The time frame was over the last month and response format a 4 point Likert scale. The first draft was reviewed by 7 oncology experts before being pre-tested on 28 patients. The instrument was modified and piloted again on a further 14 patients.

**Conclusions** A 28 item social problems questionnaire has been developed which patients found understandable, relevant and easy to complete. This now needs to be tested on a larger sample to evaluate its reliability and validity.

### P129 A STUDY OF COMPLICATION RATES IN GROSHONG AND PICC VENOUS CATHETERS IN ONCOLOGY PATIENTS

R Simcock, S Price, E Noon, M Renshaw, Sussex Oncology Centre, Royal Sussex County Hospital, Brighton, East Sussex, BN2 5BE

**Introduction** An increasing number of oncology patients require venous catheters either for persistent venous infusion or poor venous access following previous treatment. In our department we have both a nurse led PICC service and an anaesthetist led Groshong® service. We aimed to evaluate these services to establish a departmental protocol.

**Methods** All lines placed in oncology patients between 1/11/99 and 30/10/2000 were retrospectively evaluated for complications, life-span of line and delays in insertion. 60 patients were identified of whom 59 were available for study. 77 lines in total were placed in these patients (43 Groshong and 34 PICC).

**Results** There were longer delays in getting a Groshong placed (average 12.61 days) compared to PICC (8.4 days). In-patient admission was necessary for all Groshong patients but avoided in all PICC patients. PICC lines showed greater longevity than Groshong lines.

The overall complication rate for both lines was high (62.8% Groshong vs. 58.8% PICC) but in PICC lines these largely constituted clinically trivial exit site infections requiring no treatment (26.5%). Complications considered of high clinical significance (including pneumothorax, extravasation, SVC thrombus and line sepsis) were more common in Groshong lines (23.3% vs. 8.9% in PICC). Line failures (fracture or displacement) were similar in both groups (7% Groshong vs. 11.8% PICC). Procedure related complications in the Groshong group (16.3% vs. 2.9%) may have been reduced by the introduction of a fluoroscopic or USS guided line placement. Three lines placed without fluoroscopy and subsequently re-manipulated because of poor position leaked leading to an extravasation. Complications in the Groshong group led to higher patient costs with more investigation and in-patient treatment.

**Conclusion** The introduction of a nurse-led PICC service is more convenient, has reduced treatment delays and resulted in a lower complication rate compared with central venous Groshong lines over the same period.

### P130 AN AUDIT OF PERIPHERALLY INSERTED CENTRAL VENOUS CATHETERS (PICC LINES) IN PATIENTS WITH SOLID TUMOURS

K Jeffery, H McClelland & AL Thomas, Dept of Oncology, Leicester Royal Infirmary, UK

In our centre the number of patients (pts) receiving infusional chemotherapy has increased dramatically over the past 3 years with resultant major resource implications. To cope with the demand a ward-based PICC line service was established and run by a clinician with 2 nurses. In total 68 lines have been placed over a 25 month period. In 71% of cases the PICC line was requested for infusional chemotherapy, the rest of placements being for poor venous access (12%), vesicant chemotherapy (8.5%), inotropes or TPN (8.5%). Nine attempts were unsuccessful (in 4 pts unable to cannulate, in 5 pts line would not advance to correct position). Five pts needed a second line (3 pts initially had malpositioned lines, in 1 the line migrated, 1 line was replaced after resolved infection). Duration of survival of the line was from 1–237 days (median 43 days). Reason for removal was completion of treatment in 73% and line associated complications in the remainder. The complication rate for sepsis was 15%, line migration/blockage 7%, thrombosis 7% and irreparable fracture 1%. Of the 9 lines complicated with infection, 3 were treated with antibiotics by the GP and there was no record of a swab taken of the exit site. In 3 pts the catheter tip was positive and in 1 case the tip was negative when the exit site swab was positive. In 2 pts the line was removed probably for mechanical phlebitis since both the exit site swab and tip cultures were negative and the lines had been placed for less than 7 days. We found it very difficult to elucidate from the literature the desired standards for this practice since most of the literature deals with lines placed for parenteral nutrition or intravenous antibiotics. It is well-known that patients with solid malignancies pose different problems due to their associated pro-thrombotic tendency. In a literature search we found only 2 audits in a similar patient population against which to compare our figures. The complication rates in these series were very similar to those described here. We feel it is important for groups to report their PICC line figures so that national standards can be devised, particularly since more infusional chemotherapy is being prescribed. We have now developed a nurse-led clinic and organise a teaching programme for district nurses to ensure the lines are adequately dressed to reduce our infection rate.

### P131 CATHETER RELATED UPPER EXTREMITY THROMBOSIS IN ONCOLOGICAL PRACTICE – A STUDY OF INCIDENCE AND RISK FACTORS

Somnath Mukherjee, Velindre Hospital, Cardiff

**Background** Central lines are becoming increasingly important in oncology, especially in gastrointestinal cancers, where they are retained for months to deliver infusional chemotherapy. Yet data looking at line complications, like thrombosis, is scanty in UK. The incidence of pulmonary embolism has been reported to be higher with upper extremity deep venous thrombosis (UEDVT) and death due to UEDVT related pulmonary embolism is nearly four times when compared to lower limb DVT. Factors implicated for the higher incidence of thrombosis are displacement of lines into peripheral veins and left sided insertions. No national guideline exists for prophylactic anticoagulation of central lines; the multi-centre WARP trial is underway to evaluate the benefits of warfarinisation in patients with central catheters in oncological practice. At our centre Peripherally Inserted Central Catheters (PICCs) are not routinely anticoagulated.

**Aims** To determine the incidence of catheter – related thrombosis in hospital practice, and to identify any risk factors.

**Material and methods** This was a retrospective study looking at all PICC insertions between 31.6.99 and 31.5.00. Data was obtained from the PICC register. A list of patients referred for doppler of upper limb, doppler of neck veins and upper limb venograms between the period 1.7.99–31.3.01 was used to evaluate the number of catheter related thrombosis in the above cohort of patients. The following risk factors were evaluated – side of insertion, single or dual lumen catheters, site of primary tumour and the chemotherapy regimen

**Results** 20 of the 385 central catheters were complicated by thrombosis. The overall incidence of thrombosis was 5.2%. The mean time to thrombosis was 42.6 days (range 6–154 days). Males were at higher risk (5.5% vs 4.5%). Left sided insertions were at no more risk than the right. Dual lumen catheters were significantly more thrombogenic (11.8% vs 4.5%, 362 evaluable catheters). Overall risk for upper gastrointestinal malignancies was higher (8.7%, stomach – 11.1%, esophagus – 8.6%) The risk of PICC associated thrombosis was 2.8% for lower gastrointesinal malignancies. Risk of thrombosis for patients receiving ECF chemotherapy was 11%, for cisplatin and 5 Fluorouracil was 8.1%, and for continuous infusion 5 fluorouracil with without mitomycin was 3.7%.

**Conclusions** Overall incidence of catheter – related thrombosis was about 5%. Routine prophylactic anticoagulation of all peripherally inserted central catheters is probably not justified given the attendant risks of warfarinisation, especially in patients with lower gastrointestinal malignancies and those receiving continuous infusion 5 Fluorouracil with/without mitomycin. Patients with upper gastrointestinal malignancies may, however, benefit from warfarinisation.

### P132 COMPUTER-ASSISTED QUALITY OF LIFE QUESTIONNAIRES, AB Smith, G Velikova, and PJ Selby, ICRF Cancer Medicine Research Unit, St. James's Hospital, Leeds LS9 7TF

**Aim** We have used touchscreen technology successfully to present quality-of-life questionnaires to cancer patients to enable us to assess psychological morbidity, and social problems (Velikova et al., 1999). However, the standard questionnaires which have been used were designed for use in clinical trials, and therefore can be too long and time-consuming, thereby increasing the burden placed on patients, and may ignore areas that are relevant to them. Similarly, these questionnaires may exclude patients experiencing extremes of distress and/or social problems. Computer-assisted questionnaires present areas of concern to patients on a touchscreen computer. This enables patients to select aspects of their well-being which have been problematical and answer further questions, from standard questionnaires, on these concerns alone. In this way computer-assisted questionnaires can minimize or even eliminate some of the problems encountered with standard questionnaires.

**Method** A study was conducted to compare computer-assisted (CA) questionnaires with standard questionnaires (EORTC-QLQ c30) completed by patients on the same day. If patients respond to the questionnaires in the same way then agreement between the different question formats should be high. Patients were recruited from oncology wards and day clinics. Initially, to our surprise, the results demonstrated that absolute agreement between the questionnaires was very poor (25%), although this improved to 75% when differences of one on the rating scales were included. It appeared that rather than focusing on areas of concern, patients were reporting more problems on standard questionnaires. A second study therefore investigated whether the wording of the CA-questionnaires influenced patient responses. Two groups of 15 patients completed either the existing CA-questionnaire or a more inclusively worded CA-questionnaire, and the standard questionnaire. The results indicated only a slight improvement in agreement of 35% (absolute agreement). A third study investigated whether the amount of information (known as "chunks", Miller, 1956) presented to patients could explain differential responses. Patients were presented with the CA-questionnaire with the number of items reduced to 4 or 5 per screen. Greater agreement was observed between the two questionnaire format (75% absolute agreement). It is known that information capacity may be reduced in patients due to stress which may effect selection and choice processes. This is to be explored in future work.

**Conclusion** Computer-assisted questionnaires can reduce patient burden whilst still reflecting an accurate picture of patients' quality-of-life. However, this appears only to work if the number of presented per screen is modest. Future work will explore the interaction between psychological distress and selection processes on CA questionnaires.

**P132 Cont'd**

### REFERENCES

1. Miller GA (1956) The magical number seven, plus or minus two: Some limits on our capacity for processing information. *Psychological Review* **63**: 81–97
2. Velikova G, Wright EP and Smith AB et al (1999) Automated collection of quality-of-life data: A comparison of paper and computer touchscreen questionnaires. *Journal of Clinical Oncology* **17(3)**: 998–1007

### P133 COMPUTER-ADMINISTERED INDIVIDUAL QUALITY OF LIFE ASSESSMENTS IN ONCOLOGY PRACTICE

G Velikova[1], J Brown[2], A Smith[1], D Stark[1], T Perren[1], P Wright[1] and Peter Selby[1], [1]ICRF Cancer Medicine Research Unit, St James's Hospital, Leeds LS9 7TF, [2]Nothern and Yorkshire Clinical Trials and Research Unit, Leeds

It is well recognised that oncologists should consider patients' quality of life (QL) and functioning when planning and delivering anticancer treatment, but a comprehensive assessment how a patient feels requires a thorough inquiry. A standardised measurement of patients' QL may support clinicians in identifying important problems for discussion during the limited time of the medical consultations. This project investigates the use of individual QL data in practice and its impact on patients' care and well-being. In the Ist study we investigated the applicability of a standard QL questionnaire to individual cancer patients. 114 consecutive patients completed the EORTC QLQ-C30 on a touchscreen computer over a 6-month period. The QL results were compared with the corresponding medical records at individual and group level. For individual patients the serial measurement of QL allowed recognition of patterns over time corresponding to disease course. At group level, a higher proportion of patients reported problems on EORTC QLQ-C30, than were mentioned in the medical records (McNemar paired test, $P < 0.01$). Most often clinicians mentioned pain (22%–39%), and at the initial visit role (66%) and social issues (77%). For the rest of the symptoms and functions, the problems were recorded in between 1% and 25% of the notes, but 20% to 76% of the patients reported impairment. Problems which were not recorded in the medical notes tended to be of low severity with a significant trend observed for pain, fatigue, nausea/vomiting, dyspnoea, loss of appetite and physical function scale ($\chi^2$ test 11.55–34.42, df = 1, $P < 0.001$). Thus the QL data on individual patients was consistent with the clinical records but the QL profiles had more information on symptoms and particularly on functional issues.

In a 2nd study we assessed the feasibility of computer-administered individual QL measurements in oncology clinics with immediate feedback of results to clinicians and examined the impact of the information on consultations. The study employed a prospective non-randomised design with pre-test post-test within subjects comparisons and involved 3 medical oncologists and 28 consecutive cancer patients. When clinicians had the QL results they enquired more often about daily activities ($Z = -2.71$, $P = 0.007$), emotional problems ($Z = -2.11$, $P = 0.035$) and work related issues ($Z = -1.89$, $P = 0.058$). The computer measurement was well accepted by patients who felt that the questionnaires were a useful tool to tell the doctors about their problems. The clinicians perceived that the QL data broadened the range of the clinical inquiry and helped them identify issues for discussion.

The benefits for the patients from using individual QL data in practice is currently investigated in a randomised study involving 260 patients and 25 oncologists. The study will finish recruitment in May 2001.

### P134 BREAST CANCER PATIENTS USE OF A TOUCH SCREEN IN THE DAY TREATMENT AREA TO RECORD TOXICITY, HEALTH STATE AND QUALITY OF LIFE – A 12 MONTH EXPERIENCE

RCF Leonard, B Vickery, S Povey Edinburgh Breast Unit

Between the start and end of 2000, 256 patients with breast cancer had complete treatment records against which to assess their recorded symptoms attributable to disease and the effects of treatment on their lives whilst attending the day treatment area for chemotherapy. They were asked to use a touch screen questionnaire at each attendance for chemotherapy and individuals used the screen from once to 12 times. The screen questionnaire was derived from standard recording systems to assess toxicity severity and duration (31 questions) with 3 added questions on the patient's assessment of her health state, global quality of life and performance status. In this report only the major features are shown from over 31000 data items thus obtained. The records obtained were from 928 'form' completions, 617 adjuvant or neoadjuvant and 311 metastatic. The data were stored in MS Access allowing ease use of data storage and analysis. The patient data were then matched to the department treatment booking database to link outcomes against disease stage and treatment given. The data are presented as percentage scores for selected items in the 2 groups, metastatic and secondly, adjuvant or neo adjuvant.

| fatig | apptit | diarr | vom | naus | health | QL | PS | |
|---|---|---|---|---|---|---|---|---|
| 11/8 | 89/64 | 80/71 | 91/86 | 54/38 | **4/23** | **5/28** | 44/23 | **A** |
| 30/28 | 4/22 | **13/20** | 6/11 | 32/39 | 12/32 | 38/29 | 35/32 | **B** |
| 41/43 | 4/9 | **5/7** | 2/2 | 7/17 | 56/44 | 28/38 | **17/35** | **C** |
| 18/21 | 3/15 | **2/2** | 1/1 | 7/6 | **28/1** | **29/5** | 4/10 | **D** |

Figures show % incidence metastatic / % incidence adjuvant; A = nil toxicity or best health state; B = mild toxicity / impairmen of state; C = moderate toxicity or impairment; D = severe toxicity or impairment; **Bold figures** indicate clinically important differences between groups, either direction

This is a rapid and accurate method to record toxicity, functional health state, formal performance status and quality of life in a self-report, real-time format. It is quick, patient-friendly and is an extremely powerful, inexpensive technique for recording auditable data for non-trial as well as trial patients at all stages of disease for patients on treatment. We have plans to extend its use to the OPD waiting area for patients who may be on oral or hormonal therapy.

### P135 RETROSPECTIVE TREATMENT-RELATED MORBIDITY DATA DO NOT RELIABLY REFLECT CLINICAL PERFORMANCE

AS Denton[1], SJ Bond[1], S Matthews[2], SM Bentzen[1] and EJ Maher[1]. [1]Mount Vernon Centre for Cancer Treatment, Northwood, Middlesex HA6 2RN, [2]The Churchill Hospital, Oxford OX 3 7LJ

The drive for accountability has created interest in comparing individual institutions' performance using league tables. We have used data from the UK audit of cervical cancer treated with radiotherapy (RT) in 1993, to ask whether differences in morbidity between centres could be used as an indicator of quality of care.

A retrospective case-note audit of all patients receiving RT with curative intent for cervical cancer in 1993 identified 1558 cases drawn from 96% of the UK treatment centres. Severe toxicity (grade 3 and 4 according to the Franco-Italian glossary) was recorded. In all the centres but one, morbidity was graded retrospectively at the time of the audit. The 5-year prevalence of late severe morbidity was 6.1%, actuarial rate 8%.

Even if all the 53 centres had a common morbidity rate, testing each of them against the national average would produce a 94% chance that at least one of them was outside the acceptance region. We may reduce the overall risk of a false positive result to 5% by enlarging the acceptance region, according to the Bonferroni correction. After correcting for multiple testing, 49/53 centres with apparently differing rates of morbidity, were found not to be statistically different from the national average. Of the remaining four institutions, the one which had the most complete, prospectively collected data set, was the most statistically significant, $p<0.001$. The highest crude morbidity rate occurred at another centre where two out three patients experienced late morbidity.

Therefore, these results would not be appropriate for use in league tables of institutional performance. Notably, the one centre that appeared to do worse than the national average, was the only centre to have prospectively graded morbidity. League tables may be completely misleading unless data of this nature are recorded prospectively using standardised follow-up procedures.

### P136 A STUDY OF PSYCHOLOGICAL MORBIDITY IN ONCOLOGY INPATIENTS

S Amin[1], D Fyfe[2]. [1]Department of Psychological Medicine, Queens Medical Centre, Nottingham; [2]Department of Clinical Oncology, Nottingham City Hospital, NG51FP

Psychological morbidity is common in Oncology patient samples (20–40%). The Calman-Hine report (1995) stressed the importance of "providing psychosocial support". However, some patients with psychological morbidity may not be referred for specialist psychological assessment and treatment. We performed a study to compare different methods of identifying psychological morbidity, by nursing staff, junior medical staff, and a self administered questionnaire, (the Hospital Anxiety and Depression Scale (HADS), against a validating standard (a psychiatrist's assessment).

**Methods** 39 consecutive patients admitted to an oncology ward were assessed. The psychiatrist interviewed the patients using a standardised interview (Clinical Interview Scale-Revised (CIS-R)) and gave his diagnosis, the nursing staff rated the patient on a 3 point scale (good/moderate/poor adjustment), and the Senior House Officers were asked to give their psychiatric assessment. All assessments were given independently, without knowledge of the others'.

**Results** 2 patients were unable to comply with the interviews, due to their poor performance status. 6 patients were diagnosed as suffering from a major psychiatric disorder: severe needle phobia, mild depression, severe depression, generalised anxiety disorder, panic disorder, and alcohol abuse (self harm). Only 1 of these was correctly diagnosed by the SHOs, or felt to be coping poorly by the nurses. 11 patients were suffering from an adjustment disorder; 2 of these with anticipatory anxiety might have benefited from referral to a psychologist. There was good correlation between the HADS and the CIS-R score ($r = 0.81$ (95% CI 0.66–0.90), $P<0.001$). There was poor correlation between the nurses' and SHOs' assessments, and the psychiatrist's diagnosis. The SHOs tended to over-diagnose: they diagnosed depression in 4 and adjustment reaction in 4 of 20 patients without a psychiatric diagnosis. However, this may have been because they knew they were under scrutiny.

**Discussion** Despite increased awareness of emotional problems in cancer patients, many patients with psychological morbidity are still not correctly diagnosed by junior medical staff. The HADS functioned well as a screening tool in this small study, and could be considered as a means of identifying patients who might benefit from referral.

### P137 THE EFFECT OF DEPRIVATION ON INCIDENCE, MORTALITY AND SURVIVAL FOR THE MOST COMMON CANCERS IN ENGLAND & WALES

L Melcher[1], DA Power[1], RSD Brown[1], M McCormack[1], HA Payne[1], A Brock[2], P Babb[2] and MJ Quinn[2]. [1]Middlesex Hospital WIN 8AA, [2]National Cancer Intelligence Service, Office of National Statistics SW1V 2QQ

**Aims** To examine the effect of socio-economic deprivation on the incidence, mortality and survival of the ten most common malignancies in England and Wales.

**Method** The latest national statistics were obtained from the Office of National Statistics. The effect of deprivation was assessed using the 'Carstairs Index' of material deprivation, which uses census-derived socio-economic measures.

**Results** Incidence and Mortality – The incidence of six of the ten most common malignancies was higher in the most deprived groups – lung, rectum in males only, bladder, stomach, oesophagus & pancreas. There were no socio-economic differences in incidence for cancer of the ovary, colon (in both sexes) or rectum (in females). Three cancers had higher incidence in the more affluent groups – breast, prostate & non-hodgkin's lymphomas; breast cancer incidence is about 25% higher in the most affluent groups. There were no differences in mortality for breast cancer or NHL between deprivation groups.

Survival – There were significant survival disadvantages for all the ten most common malignancies in the more deprived groups.

**Conclusion** The consistent differences in survival for adults in different socio-economic groups represents an important public health issue. Differences in survival could in theory be improved by addressing the deprivation gap. An even larger effect on mortality could be obtained by eliminating differences in incidence between socio-economic groups – principally by preventative measures including a reduction in smoking.

### P138 A RANDOMISED TRIAL OF THE IMPLEMENTATION OF COMPUTERISED GUIDELINES IN CANCER PATHOLOGY REPORTING: THE CROPS PROJECT

LK Branston[1], JM Abraham[1], S Greening[2], RG Newcombe[3], R Daoud[3], J Steward[4], C Rogers[2], NS Dallimore[5], GT Williams[3] [1]Velindre Hospital, Whitchurch, Cardiff. CF14 2TL. [2]Breast Test Wales, 18 Cathedral Road, Cardiff. CF11 9LH. [3]University of Wales College of Medicine, Health Park, Cardiff, CF14 4XN. [4]Wales Cancer Intelligence and Surveillance Unit, 14 Cathedral Road, Cardiff CF11 9LJ. [5]Llandough Hospital NHS Trust, Penarth, Cardiff

**Objectives** To determine whether reporting guidelines and computerised proformas improve the completeness of histopathological cancer data available for patient management and population cancer registration. To evaluate the acceptability of the intervention.

**Design** Randomised controlled trial with a split unit design and stratified cluster randomisation.

**Setting** Pathology laboratories in Wales.

**Subjects** 16 district general hospital laboratories employing 45 histopathologists were randomly allocated to report breast or colorectal specimens by proforma.

**Interventions** Reporting guidelines were agreed by pathologists. Participants attended a training day on the cancer site they were randomised to report. Computerised proforma reports containing the items described in the guidelines were introduced. Audit meetings were held to review individual performance.

**Main outcome measures**

- Completeness and precision of histological data available to clinicians and cancer registry.
- Acceptability of the intervention

**Results** 1020 reports of resection specimens were analysed in the study arm, 1008 in the control arm. Use of proformas led to a 28.4% (15.7%–41.2% 95% CI) increase in complete reporting of a minimum dataset required for cancer registration and a 24.5% (11.0%–38.0% 95% CI) increase in complete reporting of minimum data required for patient management. Interviews suggested pathologists had changed their working practice and generally favoured proformas, but there were some problems with implementing the computer screens. The intervention also proved acceptable to surgeons, and cancer registry.

**Conclusions** A package of training, guidelines and proforma reports significantly increases completeness of pathology reporting.

### P139 ESTABLISHING EFFECTIVE NETWORKS TO SUPPORT CLINICAL CANCER RESEARCH: THE WALES CANCER TRIALS NETWORK

L Branston[1], T Maughan[1], N Stuart[2], MD Mason[1]. [1]Wales Cancer Trials Network, Velindre Hospital, Whitchurch, Cardiff, CF14 2TL. [2]Clinical Trials Unit, Ysbyty Gwynedd, Bangor, LL57 2PW

**Background** The Wales Cancer Trials Network (WCTN) was set up in 1998 with the objective of increasing clinical cancer trials activity across Wales. 16,000 new cancer cases are diagnosed annually in Wales and historically an average of 400 patients (2.5%) entered multi-centre cancer trials each year.

**Methods** The first phase of WCTN funding has provided 4 oncology research nurses to 3 cancer centres (Cardiff, Swansea, North Wales) and 1 cancer unit (Bangor) in Wales. WCTN staff have opened a portfolio of priority trials, supported new investigators and recruited patients to studies. The WCTN has provided training and education for NHS staff in good clinical practice, informed consent and cancer therapies. The WCTN central office provides managerial, networking and statistical support to outlying trials offices and runs randomisation and data management for 4 trials regionally. WCTN has taken a regional approach to problem solving with regard to meeting the excess costs of clinical trials and making the ethics committee system work more smoothly.

**Results** Cancer trial recruitment has increased by over 50% since the introduction of the Network. This represents an increase from 490 in 1997 to 741 in 2000 (4.6% of new cancer cases). The WCTN aims to increase this to 10% of new cancer patients by 2006.

Network staff have opened 48 trials in 103 separate ethical submissions. In order to meet a 10% target we aim to broaden our portfolio of trials to include palliative and primary care and cancer genetics.

The Network has supported 21 investigators who were not previously research active.

We estimate that 1 WTE Research Nurse working in oncology trials can recruit 50–60 patients per year.

**Conclusions** A 10% recruitment target will prove exacting for the UK cancer service. Increased recruitment requires highly committed investigators particularly in cancer sites with a broad and varied portfolio of high quality studies available.

The Wales Cancer Trials Network is funded by The Cancer Research Campaign and National Assembly for Wales.

### P140 DIFFERENTIATED THYROID CARCINOMA IN THE ELDERLY.

L Vini, J Marshall, R A'Hern, C Harmer. Thyroid Unit, Royal Marsden NHS Trust, London, SW3 6JJ, UK

**Aim** This is a retrospective review aiming to assess the behaviour of thyroid cancer in elderly patients and also evaluate the results of treatment.

**Patients – Methods** Among 1448 patients with differentiated thyroid cancer treated in our Unit over the past 60 years, we identified 111 patients who were over the age of 70 at diagnosis (range 70–93 years). Follow-up ranged from 1–19 (median follow-up 9 years).

**Results** There were 28 men and 83 women (male/female ratio 1:3). The majority of patients (80%) presented with a thyroid mass. Fifty-eight tumours were papillary, 46 follicular and 7 Hurthle cell carcinomas; 55 carcinomas were well-differentiated tumours while the remaining were either grade II (22) or grade III (34). The majority of patients had locally advanced disease (70% of tumours were T3 or T4 and 45% were associated with involved lymph nodes); distant metastases at diagnosis were documented in 26 cases (25%). Surgical management included: total thyroidectomy in 52 cases, lobectomy or hemithyroidectomy in 32 and enucleation or biopsy in 25; a modified neck dissection was performed in 6 and a simple node excision in 12 cases. Radioactive iodine was administered to 80 patients, 22 received an ablation dose of 3 GBq while 58 required multiple doses (cumulative activity 6.6–29 GBq). Adjuvant external beam radiotherapy was given to 19 patients. Cause-specific survival was 75%, 50% and 50% at 5, 10 and 15 years respectively. Multivariate analysis identified age over 75 years as the single most important independent prognostic factor for survival while stage of the primary tumour had a significant effect on the risk of local recurrence.

**Conclusions** Differentiated thyroid cancer in elderly patients appears to behave more aggressively and has a less favourable prognosis than in younger adults; a significant proportion of tumours show extrathyroid spread, there is a prevalence of follicular histotype with features of poor differentiation, a higher incidence of distant metastases mainly in bones and lungs with a relatively high proportion of those being non-functioning. Early diagnosis and radical treatment i.e. total thyroidectomy plus lymphadenectomy followed by radioiodine therapy are therefore required.

### P141 SUBSEQUENT MALIGNANCIES IN PATIENTS TREATED FOR DIFFERENTIATED THYROID CANCER.

DA Wheatley, L Vini, A'Hern, C Harmer, Royal Marsden Hospital, Sutton

**Aim** This is a retrospective review to assess the risk of developing a second malignancy amongst patients who had treatment for well-differentiated thyroid cancer.

**Method** Between 1929–1999, 1448 patients with well differentiated thyroid cancer were treated in our unit. Analysis so far has been performed on 1340 patients who had a minimum follow up of one year after completion of treatment. Of these 938 patients have received radioiodine therapy, whilst 402 patients have not received radioiodine.

**Results** Overall 109 patients (8%) developed a second malignancy. The commonest second primary sites were; breast in 23 patients, gastrointestinal in 18 patients, lung in 13 patients, leukemia or myelodysplasia in 13 patients, others in 40 patients. Seventy-nine patients had been previously treated with radioiodine (25 of whom also received external beam radiotherapy). While 30 had not received radioiodine therapy (18 of those had been treated with radiotherapy). No significant difference was seen among these groups regarding the site of second primaries.

**Conclusions** In our series only 8% of patients with differentiated thyroid cancer developed a second malignancy; The incidence of second malignancies was not significantly different among the patients who had been treated with radioiodine (8.5%) compared to those not treated with iodine (7.5%).

### P142 TREATMENT OF UNRESECTABLE CHOLANGIOCARCINOMA: 8-YEARS EXPERIENCE FROM ONE CENTRE.

DA Power, M Beresford, NG Mikhaeel and MF Spittle. The Meyerstein Institute of Oncology, The Middlesex Hospital, London W1N 8AA

**Aims** To review the role of radiotherapy and chemotherapy in the management of unresectable cholangiocarcinoma.

**Methods** The management of forty consecutive inoperable cholangiocarcinoma patients between 1993 and 2000 in The Middlesex Hospital was reviewed. All patients received radiotherapy, either external beam alone or combined with high-dose-rate brachytherapy. Fourteen patients received concomitant chemotherapy. Primary end point was overall survival. The length of inpatient stay and use of stenting procedures were also reviewed.

**Results** 67 % were male and 33% female. Average age at diagnosis was 61.4 yrs (range 43–77). One patient had undergone biliary resection prior to treatment for relapse. Other patients were diagnosed radiologically (34/40) or at laparotomy (5/40). 73% (29/40) were histologically confirmed adenocarcinoma. Median survival from time of diagnosis was 9 months (range 1–31). Average inpatient stay was 39 days. 69% required external and 92% internal stenting. Average number of internal stents was 2.4 (range 1 to 8). There was no statistically significant difference in survival by the use of more intensive radiotherapy regimens (>40Gy in 2Gy or less fractions) or the addition of brachytherapy. Median survival increased with the use of concomitant chemotherapy (16 months vs 7 months) but numbers are small and the difference is not statistically significant.

**Conclusions** Cholangoiocarcinoma has a poor prognosis with a median survival of 9 months. There is no evidence, from this retrospective study, that additional treatments add to overall survival and palliation above the use of stenting procedures and randomised controlled trials with quality of life assessment are required.

### P143 TOTAL SKIN ELECTRON TREATMENT FOR MYCOSIS FUNGOIDES – THE COVENTRY EXPERIENCE, 
P Chung, NC Thorp, DC Wilson, AM Stevens, JA Mills, and RJ Grieve, Walsgrave Cancer Centre, Coventry CV2 2DX, UK

**Introduction** Total skin electron radiation is effective in the management of mycosis fungoides. Many methods have been described; the majority requires large treatment rooms with machine modification. We modified the Christie Technique; this required minimal machine and room modification. In order to achieve a more even distribution of radiation, avoiding lateral fall off, the patient lies at an angle of 30˚ to the treatment beam. Radiation is delivered using arcing beams of 6MeV electrons, arranged to have their centres of rotation above the head and above the feet. Beam overlap is optimised by immobilising the patient in VAC bags. Patients are treated in 8 sections – 4 anterior and 4 posterior quadrants – with 2 quadrants treated each day, each quadrant receives 3 fractions, to a total dose of 24 Gy over 3 weeks. A prospective audit of dosimetry for the first 20 patients treated was performed and is presented.

**Method** Data was collected prospectively. Doses were measured with TLDs placed on the top of the head, both shoulders, chest, both legs and soles, and at the match point of the arcing treatment beams.

**Results** The mean dose given to the skin surface of all the patients was 21.5Gy (SD ± 4.7%). For the first 6 patients there was relative overdose at the top of the head, shoulders and soles of the feet. For the subsequent 14 patients lead shielding was applied to these areas, producing a dose that was acceptable. Lateral doses across the patient were symmetrical. Longitudinally the mean doses even after shielding showed a statistically significant variation ($p < 0.05$); top of head 21.5Gy; shoulders 23.5Gy; anterior chest 19.0Gy; anterior matchpoint 16.0Gy; anterior legs 22.3Gy; soles 22.5Gy. Posterior doses were similar.

**Discussion** This technique was easily implemented with a standard Philips SL25 machine requiring minimal modification and conformed to AAPM Report No. 23 requirements. Patients completed the treatment but the 30˚ angle required is uncomfortable for some patients, who maintain this position for approximately 20 minutes. There can be a relative underdose at the matchpoint of the arcing beams due to self-shielding. Despite the short fraction technique, the treatment time of up to 45 minutes makes a significant impact on a busy clinical department, only allowing one patient to be treated at any one time. As few centres offer this treatment, a national waiting list has developed. We are commissioning a Betatron that will treat patients using a beam that scans along the patients' length at an angle of 30˚. This allows the patient to lie flat and be more comfortable. This system will improve the longitudinal inhomogeneity experienced with the present technique. Importantly, it will free linear accelerator time for routine work and because it will be solely dedicated for TSE treatment it will enable us to provide treatment without delay.

### P144 GESTATIONAL TROPHOBLASTIC DISEASE IN THE ASIAN POPULATION OF NORTHERN ENGLAND AND NORTH WALES 
BWL Tham, JE Everard, BW Hancock, YCR Department of Clinical Oncology and Trophoblastic Tumour Centre, Weston Park Hospital, Sheffield S10 2SJ

The aim of this study was to determine the incidence and trend of Gestational Trophoblastic Disease (GTD) in the immigrant Asian population of Northern England and North Wales.

A retrospective case note review was undertaken of 312 Asian patients who were registered with Weston Park Hospital, one of 3 UK Trophoblastic Screening Centres, from 1991 to 1999.

During the 9-year study period, the incidence of GTD in the northern part of England and North Wales averaged 1 per 714 live births. The incidence of GTD in the Asian population was 1.92 times higher than in the non-Asian population (1 per 400 live births versus 1 per 746 live births). There was an excess of molar pregnancies in the extreme maternal age groups. The incidence in these women was about 2 times higher than for the whole reproductive cohort. The frequency proportion of partial (PM) to complete (CM) hydatidiform mole also increased with the age. The ratio of PM to CM increased from 0.9 in the lower age to 3.3 in the older age group. There was a slow but rising trend in the incidence of GTD in the UK. The increase was significantly higher in the Asian than non-Asian population.

Setting up of regional or national registration centres helped to provide more accurate estimate of the true incidence of a disease. The incidence of GTD in Asian women is higher than in non-Asian and is increasing. However, there must be a move to identify ethnic groups by self-ascribed ethnicity, together with alignment and conformity in ethnic definition between centres so that meaningful results can be derived.

### P145 Ewing's Sarcoma; 5 years experience at a tertiary referral centre in Pakistan. 
AH Sadozye, K Saeed Shaukat Khanum Memorial Cancer Hospital (SKMCH) Lahore, Pakistan

**Background** Ewing's sarcoma is an uncommon malignancy in adults. The treatment is multi-modality including chemotherapy, surgery and radiotherapy.

**Materials and Methods** Case notes of all patients seen at SKMCH, between January 1995 and December 1999, with a histological diagnosis of Ewing's sarcoma or PNET, were reviewed in a retrospective manner. Attention was paid to the following; Gender, date of first diagnosis, stage, visceral mets at presentation, skeletal mets at presentation, local recurrence at presentation, chemotherapy, number of cycles of chemotherapy, definitive surgery, definitive radiotherapy (XRT), post-operative pathology, end-of-treatment (EOT) result, relapse, relapse free period, status at last follow-up.

**Results** A total of 24 patients were seen during the above-mentioned period with a diagnosis of Ewing's sarcoma/PNET. Of these 80% were male, median age was 24.5 years (range 17–44 yrs). At presentation 66% had metastatic disease. A minority (5/24; 20%) presented after definitive surgery, performed up front. Chemotherapy was administered to 14/24 (58%) patients. Of these 36% each had EVAIA and VAIA regimens whereas 14% had VACA and 14% had other regimens. A median of eight chemotherapy cycles were administered. Definitive surgery after neo-adjuvant chemotherapy was performed in 20% (5/24) patients. The pathologic complete response rate was 40% (2/5). Radiotherapy was given to 11/24 patients (45%). Only 15 patients (62%) had evaluable disease and end-of-treatment analysis shows an overall response rate of 46%. At the time of analysis, with a median follow-up of 31 weeks (average 45 wks), 3/7 responders have relapsed. Only 24% patients were disease free at their last follow-up visit.

**Conclusions** A majority of our patients presented with metastatic disease, some of them after having had erratic and maybe inappropriate treatment by physicians with little knowledge of the disease. This is reflected in our poor survival. Early diagnosis and referral to centres, with medical oncologists/surgeons and radiation oncologists all having interest and expertise in the disease, is essential and needs to be highlighted amongst the medical community in Pakistan.

### P146 THE NATURAL HISTORY OF ASYMPTOMATIC MALIGNANT MESOTHELIOMA. 
PV Murray, MER O'Brien, IE Smith, A Norton & S Ashley. Lung Unit, Department of Medicine, The Royal Marsden Hospital, Surrey, and MidKent Oncology Centre, Maidstone

Studies have shown an overall median survival in malignant mesothelioma of 4–12.5 months (1)(2). A recent trial had a 10 patient subgroup, picked up by incidental chest radiography, with a longer median survival of 21 months (3).

**Aims** To study the natural history of mesothelioma in patients with absent or mild symptoms seen at the Royal Marsden and MidKent Oncology Centre.

**Methods** 33 suitable patients with a median age of 62 years (29 male) were identified between 1994 and 2000. Of this group, 24 patients were randomised in a trial comparing best supportive care alone (n = 13) to best supportive care with SRL 172 (n = 11), a heat-treated *Mycobacterium vaccae* used as immunotherapy, with chemotherapy later if necessary. Data analysis has found no advantage in any group receiving SRL172 compared to standard. Therefore for statistical purposes all patients initially received best supportive care. Key dates and times were recorded for all patients.

**Results** The median survival in the whole group was 20 months from diagnosis. The median time to worsening symptoms was 5 months and to start of chemotherapy 9 months. Those patients with mild symptoms had a median time of 3 1/2 months of symptoms before the diagnosis was made.

**Conclusions** Our study gives further evidence that, in advanced stage mesothelioma, if relatively asymptomatic at diagnosis, prolonged survival without treatment is possible.

**Implications** This study was a natural history baseline to a new trial at The Royal Marsden looking at early vs delayed chemotherapy in mesothelioma (The MED trial) as a new approach to treatment.

1. Law MR, Hodson M.E and Turner-Warwick M (1984) *Eur. J Respir Dis* **65**: 162.
2. Ruffie P, Feld R and Minkin S et al. (1989) *J Clin Oncol* **7**: 1157.
3. Yates D.H., et al (1997) *Thorax* **52**: 507.

### P147 COMPARISON OF THE CLINICO-EPIDEMIOLOGICAL, PATHOLOGICAL AND MOLECULAR FEATURES OF ADENOCARCINOMAS AROUND THE GOJ

K Dolan[1], JK Field[2], SJ Walker[3], R Sutton[3]. [1]Department of Surgery, Leeds General Infirmary, Leeds LS1 3EX, and Departments of [2]Molecular Oncology and 3Surgery, University of Liverpool

Adenocarcinomas of the cardia resemble adenocarcinomas of the oesophagus epidemiologically in population studies based on the ICD-0 subsite classification, which classifies carcinomas of the GOJ as carcinomas of the cardia. This study was to determine if adenocarcinomas of the oesophagus and GOJ were similar in other respects with particular emphasis on molecular features.

Forty-seven adenocarcinomas arising in the proximity of the GOJ were subdivided into 20 carcinomas located exclusively within the lower third of the oesophagus (L), 9 mainly within the oesophagus but extending distally across the GOJ (L/GOJ) and 18 straddling the GOJ (GOJ). Comparison of the clinico-epidemiological (age, sex and survival), pathological (presence of Barrett's oesophagus, length, grade and stage of carcinoma) and molecular (allelic loss, microsatellite instability and TP53 mutations) features was performed.

Adenocarcinomas at each of the three subsites were similar in all of the features studied, including the presence of Barrett's oesophagus. There were no significant differences in the location or frequency of allelic loss, and direct DNA sequencing revealed identical TP53 mutations in adenocarcinomas at each of the three subsites, suggesting a common aetiology.

Adenocarcinomas located exclusively within the oesophagus and adenocarcinomas involving the GOJ are similar in all studied parameters, suggesting that they may represent the same disease. Carcinomas involving the GOJ should not be classified with carcinomas of the cardia as a gastric malignancy, but should be classified as an oesophageal carcinoma with subsite GOJ.

### P148 STENTING IN OESOPHAGEAL CANCER RELIEVES DYSPHAGIA BUT DOES NOT AMELIORATE NUTRITIONAL STATUS: RESULTS OF A RETROSPECTIVE SERIES

SY Abdallah, HL McCabe, A Early and A Maraveyas, Academic Department of Oncology, Princess Royal Hospital, Hull

**Background** Weight loss and malnutrition are common problems in oesophageal cancer (OC) patients. Weight loss leads to increased morbidity from surgery, radiotherapy and chemotherapy, and is one of the most persistent complaints in OC patients. Oesophageal stenting is used to relieve dysphagia in inoperable OC, but is not known whether it is effective in maintaining nutritional status. The aim of this retrospective study was to compare nutritional indices of patients with OC before and after uncomplicated oesophageal stent insertion, who were dysphagia-free for over 8 weeks.

**Methods** The notes of 107 patients with OC, who had undergone stent insertion in our radiology department from 1995–2000, were reviewed. Weight, serum albumin and lymphocyte count (recorded as markers of nutrition) were compared before, and 8 weeks after, stent insertion. 50 patients were excluded due to operation, PEG tube insertion, complicated stent insertion, persistent post-stent symptoms or lack of data.

**Results** Of 57 patients, 47 (82.5%) reported an improvement in dysphagia following stent insertion, 6 (10.5%) reported no improvement, and data was not available in 4 (7%). 41 patients (72%) remained symptom free for a minimum of 8 weeks. Of these 41, 29 (71%) progressively lost weight over time (>5% of body weight). Weight was stable (+/-5%) in 8 (19%), and increased (>5%) in 4 (9.7%). Serum albumin decreased by >10% in 24 of 41 patients (58%), remained stable (+/-10%) in 11 (26%), and increased (>10%) in 1 (2.4%). Albumin was not known in 5 (12%). Lymphocyte count decreased by >10% of baseline in 23 (56%), remained stable (+/-10%) in 2 (4.8%) and increased by >10% in 11 (26%). Lymphocyte count was not available in 5(12%).

**Conclusion** Oesophageal stenting relieves dysphagia in OC patients, but does not appear to ameliorate nutritional status, as evidenced by progressive weight loss and reduction in serum albumin and lymphocyte count seen in most patients. Weight loss in patients with gastrointestinal cancers is associated with more complications from chemotherapy and radiotherapy and leads to dose compromise. It is correlated with a shorter disease free survival and overall survival, decreased quality of life and performance status, compared to patients without weight loss.[1] The simple alleviation of mechanical obstruction in these patients is not an adequate treatment for this cancer-related symptom. Further efforts are required to reverse weight loss and improve nutritional status in OC patients.

1. Andreyev HJ et al. Why do patients with weight loss have a worse outcome when undergoing chemotherapy for gastrointestinal malignancies? *EJC* **98**;34(4): 503–509.

### P149 A RETROSPECTIVE ANALYSIS OF PATIENTS WITH SMALL BOWEL MALIGNANCIES SEEN AT MOUNT VERNON CANCER CENTER OVER TEN YEARS

AEF Roy, M Harrison, Mount Vernon Cancer Center, Northwood, Middlesex HA6 2RN

Primary malignancies of the small bowel are rare. Management of these cases is therefore based on limited experience and no published randomised trials. The aim of this project was to review how these malignancies had been treated at the Mount Vernon Cancer Center over the last ten years.

The Mount Vernon Cancer Database was searched for patients with primary malignancies of the small bowel. Case notes were reviewed with respect to the tumour type, location, TNM stage, surgery, chemotherapy and survival. 48 pts with small bowel primaries were seen over the last ten years, and the case notes were available on 31 patients. The average age at presentation was 66 yrs with an even sex distribution. 17 pts (55%) had adenocarcinoma; 6 pts (19%) had lymphoma, and 7 pts (23%) had carcinoid tumours. I patient had a malignant haemangioendothelioma. The adenocarcinomas were distributed evenly between the duodenum, jejunum and ileum. The lymphomas and carcinoids occurred most frequently in the ileum.

Of the 17 patients with adenocarcinoma, 12 (71%) had radical surgery. The others had palliative procedures or no surgical intervention. The mean survival in those considered suitable for radical surgery was 1 yr 8 m and only 8 mths in the others. Of those that had radical surgery, 50% had post-operative chemotherapy. The selection of those to receive chemo did not appear to depend on the TNM stage or tumour grade. 5 different regimes were used but all were 5FU based. The average survival in those that received chemotherapy was 1 yr 3 mths and 1 yr 9 mths in those who did not receive chemotherapy.

All 7 patients with carcinoid tumours had surgical resection, 2 had adjuvant radiotherapy (one of these also received De Gramont). The median survival was 8 yrs.

Of the 6 patients with lymphoma, 4 had high-grade disease and received CHOP (1 recurred and had PMitCBO). Of the 2 with low-grade 1 recurred post-surgery and received chlorambucil and radiotherapy. 5 of these patients are still alive 6–10 yrs post diagnosis, the sixth died at 6 yrs from lung cancer.

These results are comparable with other published series with regard to tumour types and distribution. The survival figures are good for lymphoma and carcinoid tumours. The numbers are too small for any statistical analysis, but there does not seem to be any survival advantage to adjuvant chemotherapy in adenocarcinoma of the small bowel.

### P150 SURVIVAL IN PATIENTS WITH ADVANCED COLORECTAL CANCER IS SIGNIFICANTLY INFLUENCED BY QUALITY OF LIFE

NR Maisey, A Norman, M Watson and D Cunningham, Department of Medicine, Royal Marsden Hospital, Downs Road, Sutton, Surrey SM2 5PT

**Background** Quality of life (QoL) evaluation is recognised as an important outcome parameter in oncology. The aim of this study was to investigate the influence of baseline QoL on survival in patients treated within clinical trials of chemotherapy for advanced colorectal cancer (CRC).

**Methods** From 1992 to 1998 4 randomised clinical trials in advanced CRC were conducted at this institution. Informed consent was obtained from all patients, and studies received local ethical approval. The EORTC-QLQ-C30 questionnaire was completed prior to the commencement of chemotherapy. An overall QoL score was calculated from all domains of the EORTC-QLQ-C30 questionnaire. Data were prospectively recorded and analyses were performed on median-dichotomised baseline QoL and clinical prognostic factors.

**Results**: Baseline QoL questionnaires were completed in 501 patients. Median age was 62 (33–82), 63% were male, 98% had PS 0–2 and 82% had metastatic disease. Median follow up was 2.84 years. All QoL domains other than financial, were significant predictors of survival in the univariate analysis. One year survival was 39.9% and 72.0% ($P<0.0001$) for patients with overall scores below and above the median (80) respectively. In the baseline Cox model, the following were significant independent predictors of survival: PS, metastatic disease, haemoglobin, weight loss, CEA and albumen. Other than emotional/cognitive functioning, constipation, diarrhoea and financial domains, all QoL scales were significant independent predictors of survival ($P<0.035$). In the final model the overall QoL score remained highly significant as an independent predictor of survival ($P<0.0001$) with a hazard ratio of 1.68 (95% CI 1.3 to 2.16).

**Conclusion** Baseline QoL is a strong independent predictor of survival in patients with advanced CRC. QoL measurements should be routinely recorded in clinical trials to stratify cohorts and aid trial comparison, and may also have relevance in the choice of initial therapy in routine clinical work.

### P151 CLINICAL IMPACT OF A NATIONAL LYMPHOMA REVIEW PANEL – A PILOT STUDY

E Toy[1], J Lester[1], I Kerby[1], T Maughan[1], C Poynton[2], S Dojcinov[3], R Attenoos[3], C O'Brien[3], A Caslin[3], [1]Velindre NHS Trust, [2]University of Wales College of Medicine, [3]All Wales Lymphoma Panel

**Background** In 1998 an All Wales Lymphoma Pathology Review Service was established. The aim was multi-fold; to assess the accuracy of lymphoma diagnosis in Wales, to identify diagnostic problem areas and to provide a higher ascertainment of diagnosis to facilitate optimal clinical management. Over 32 months, 745 cases were submitted and the overall histological diagnosis was changed in 34% of cases submitted – 230 patients.

**Method** Case notes were obtained on a random sample of 33 patients whose diagnosis had been changed. Data regarding medical history including comorbidity, examination and investigation findings was extracted and presented to an expert panel. Two hypothetical management plans were generated based on initial and revised histology reports and the individual patient data. These were then compared. Data was also collected on whether the actual management had changed due to the revision in diagnosis and whether submission to the panel resulted in a treatment delay. Patients whose specimens were initially reported as "Lymphoma NOS" were not counted as a change in management as it was assumed further steps would be taken to further categorise the disease.

**Results** 17 patients had a change in their hypothetical management.

| Change | Number of Patients |
|---|---|
| Different Chemotherapy Regimen | 8 |
| Radiotherapy to Chemotherapy | 3 |
| No treatment to chemotherapy | 3 |
| Chemotherapy to no treatment | 1 |
| Repeat biopsy to chemotherapy | 1 |
| Chemotherapy to investigation | 1 |

However, in practice, only 12 (36%) had their first line management altered. 2 died before change could be instituted, 1 was unfit for treatment and 2 who had experienced a good response to initial treatment did not have this altered. The panel diagnosis affected one 2$^{nd}$ line therapy. Panel review delayed treatment in a minority of cases (2 patients).

**Conclusions** These data suggest that a significant proportion of patients are not receiving optimal management of their lymphoma because the true diagnosis is unknown to the clinician and patient. Central review of lymphoma is essential.

### P152 RISK ADJUSTED PROGNOSTIC MODELS FOR HODGKIN'S DISEASE (HD) AND GRADE II NON HODGKIN'S LYMPHOMA (NHL): VALIDATION ON 6,728 PATIENTS.

SE Low[1], J Horsman[1], P Smith[2], D Linch[2], H Hancock[1] and BW Hancock[1]. [1]YCR Department of Clinical Oncology, Weston Park Hospital, Sheffield, S10 2SJ, [2]British, National Lymphoma Investigation, The Middlesex Hospital, Mortimer Street, London, W1N 8AA

**Background** The prognostic significance of 20 putative markers has been assessed in a consecutive series of 1,198 patients with malignant lymphoma seen by the Sheffield Lymphoma Group over three decades (Low et al., 2000. British Journal of Cancer *83* (Suppl 1): 83). Using significant factors from multivariate analyses, risk adjusted prognostic models were derived for Hodgkin's disease and NHL Grade II. Data from an additional 6,728 patients on the British National Lymphoma Investigation database was used for validation.

**Results** In 536 patients with NHL Grade II our model, based on albumin, age, ESR, LDH and stage, identified three risk groups with predicted five-year survival rates of 55.7%, 44.0%, 34.9%. Five year survival rates of 94.3%, 82.9%, 64.6%, 51.7% were identified from 315 patients in the HD model using age, albumin and lymphocyte count. These models were then applied to 4,411 patients with HD and 2,317 patients with NHL Grade II. Survival curves derived from these validation groups showed significant differences according to our risk models, with 5 year survival rates of 89.8%, 80.3%, 67.2, 46.2% for HD and 56.3%, 41.3%, 30.3% for NHL Grade II for the different risk groups ($P<0.0001$).

**Conclusions** The risk models we developed may be useful in predicting outcome and to facilitate decisions on treatment for individual patients. Whilst these models are to be tested prospectively, it is important to obtain data on new markers that will further refine the identification of appropriate prognostic groups.

### P153 NON-HODGKIN'S LYMPHOMA PRESENTING WITH SPINAL INVOLVEMENT: THE SHEFFIELD LYMPHOMA GROUP EXPERIENCE (1970–2000)

HY Ching, J Horsman, C Radstone, H Hancock and BW Hancock, YCR Department of Clinical Oncology, Weston Park Hospital, Sheffield, S10 2SJ

Spinal non-Hodgkin's lymphoma is rare. We retrospectively reviewed the clinical and histopathologic records of 39 consecutive patients referred to the Sheffield Lymphoma Group from 1970–2000 and analysed the prognostic differences between localised (Stage $I_E$ & $II_E$) and secondary (Stage III & IV) spinal non-Hodgkin's lymphomas (S-NHL) patients. 45% of all patients were over 60 years old. More patients were male (58%); presented with Stage $I_E$ and $II_E$ (63%), mostly of intermediate/high grade histology (74%); over a third had 'B' symptoms; nearly a third (11 patients) were paraplegic and 14 had sphincter dysfunction at diagnosis. The overall survival of all patients was 39% at 5 years (median 24.7 months), whilst that of localised S-NHL was 51% (median 89.7 months). Univariate analysis showed better survival for patients with good mobility status at presentation ($P<0.01$) and complete response to initial treatment ($P<0.001$). In primary S-NHL, histology ($P<0.05$) significantly influenced overall survival. In conclusion disease is frequently locally advanced at presentation with aggressive histologic grade: thorough staging should always be performed to exclude widespread disease. Good mobility status predicts for good survival outcome. Optimal treatment is still uncertain.

### P154 MANAGEMENT OF LYMPHOMA PATIENTS IN A CANCER UNIT: AN AUDIT OF 15 YEARS' EXPERIENCE IN A DISTRICT GENERAL HOSPITAL.

JD Chester[1], M Howarth[2], CJ Percy Clark, DR Goldesbrough, SE Bogle, CJ Bradley and D Parker, [1]ICRF Clinical Centre, St. James' University Hospital, Leeds; [2]Manorlands Hospice, Oxenhope, Keighley; Bradford Royal Infirmary, Duckworth Lane, Bradford

Until recently, many cases of lymphoma in the United Kingdom have been treated in specialist regional centres. The results of studies from specialist centres may not be representative of lymphoma patients as a whole. Furthermore, as a consequence of the Calman-Hine Report, there is an increasing tendency for lymphoma patients to be treated locally, in Cancer Units. Relevant data for patients treated outside specialist centres are therefore required.

We have collected data prospectively on 208 consecutive lymphoma patients presenting, on an all-comers basis, to the Medical Oncology Unit at Bradford Royal Infirmary, between 1981 and 1996. Male:female ratio was 54%:46%. Median age of patients at diagnosis was 56 years (range 17–89 years). Tumours were initially classified according to the Working Formulation, and have subsequently all been re-classified according to the REAL classification. 22% of patients presented with Hodgkin's Disease, the remainder having Non-Hodgkin's Lymphomas. Patients were treated with radiotherapy and/or chemotherapy, according to local protocols. Thirteen (6.2%) patients required further management in the Cancer Centre upon disease progression/relapse.

Five year actuarial survival was 72.7% for Hodgkin's disease and 55.7% for Non-Hodgkin's lymphoma. Age and stage of disease were the only predictors of survival in a multivariate analysis. Histological classification was not a useful predictor of survival in this analysis. Comparison of our local survival figures with those from national and international registry data suggest that survival figures comparable to those obtained nationally and across Europe are attainable in a Cancer Unit.

Multiple pathways of referral of lymphoma patients operate in our region. Our Unit treated only 22% of the patients registered in Bradford between 1986 and 1995 as having lymphoma. The vast majority of the remainder were managed by Haemtologists and/or Clinical Oncologists.

This unique data set of lymphoma survival in a District General Hospital may provide a comparator for future assessments of the success of lymphoma treatment in Cancer Units, within the new Calman-Hine structure for cancer management.

### P155 LOSS OF HETEROZYGOSITY ON CHROMOSOME 11Q24 IN COLORECTAL AND EPITHELIAL OVARIAN CANCER.

KP Watt[1], Li Li, GC Sellar[1], EP Miller[1], D Scott1, M Stewart[1], JF Smyth[1], H Gabra[1] [1]ICRF Medical Oncology Unit, Western General Hospital, Edinburgh, UK

Ten polymorphic microsatellite markers on chromosome 11q24 were amplified by PCR from normal/tumour DNA pairs from 39 patients with colorectal and 65 patients with epithelial ovarian cancer. Samples were run on the 310 ABI prism and allelotype assigned using the genescan software. LOH rates at each marker were determined. Bioinformatics resources, predominantly NIX analysis have been used to determine the order of markers within the 11q24 region. Concurrently, genes and ESTs in the region have been identified using the same approach.

In the ovarian tumours, 2 regions of LOH have been identified from within the previously defined 8.5 Mb minimum region of deletion (Cancer Res. 1996: 56 950–954). A 2 Mb region centred on the marker GATA69G01 is associated with advanced stage disease ($P = 0.026$) and adverse survival ($P = 0.013$). In multivariate analysis, LOH of the second region centred on D11S4131 is an independent adverse survival variable ($P = 0.0043$).

Two regions of LOH were also identified in the colorectal tumours. A similar region of LOH at D11S4131 was defined but showed no association with adverse survival. A separate more centromeric region of LOH between D11S912 and D11S4150 was also identified.

Candidate tumour suppressor genes from these regions are being sought and investigated. Several ESTs near the D11S4131 marker have been identified and shown to be weakly expressed by rtPCR in normal human ovarian surface epithelial cells. Expression analysis of these ESTs in cancer cell lines has yet to be performed.

This study demonstrates similarities and differences in the genetic lesions sustained by these tumour types. It narrows the region of previously documented LOH thus focusing attention on the region of chromosome 11q24 that houses the important tumour suppressor genes.

### P156 FUNCTIONAL IDENTIFICATION OF RALDH2 DYSREGULATION BY CDNA REPRESENTATIONAL DIFFERENCE ANALYSIS IN CHROMOSOME 11 MEDIATED GROWTH SUPPRESSION OF OVARIAN CANCER.

C Blenkiron[1], GC Sellar[1], E Stronach[1], GJ Rabiasz[1], EP Miller[1], DJ Porteous[2], JF Smyth[1], H Gabra[1] [1]ICRF Medical Oncology Unit, [2]Medical Genetics section, Department of Medical Sciences, University of Edinburgh, Molecular Medicine Centre, Western General Hospital, Edinburgh, EH4 2XU

Frequent loss of heterozygosity (LOH) at regions on chromosome 11p in epithelial ovarian cancer suggests the presence of tumour suppressors in these locations.

Microcell-mediated chromosome transfer (MMCT) has been used as a method to search for such genes. OVCAR3, a human ovarian cell line, contains only one copy of chromosome 11, which is fragmented and rearranged throughout. Transfer of normal chromosome 11 into OVCAR3 by MMCT produced microcell hybrids which display growth and cellular migration suppression.

Subsequently, mRNA from the parent OVCAR3 clonal line and from 11OH2.1, a growth suppressed microcell hybrid, was used in the subtractive method of cDNA-RDA.

cDNA-RDA was successfully used to enrich for transcripts that were significantly down regulated in the suppressed cell lines. cDNA-RDA of OHN, a clonal derivative of the OVCAR3 parent line, versus 11OH2.1, identified transcribed sequences that were clearly differentially regulated by RT-PCR. These included KIAA1058, HSPC307, cdc37 and 2 novel transcripts. Also among these was Retinaldehyde dehydrogenase 2 (RALDH2), a gene that has been implicated in the production of all-trans retinoic acid. RALDH2 was strongly downregulated by quantitative RT-PCR and Northern blotting in association with chromosome 11 mediated growth suppression. We have developed and transfected antisense constructs into a parental derivative cell line OH1. Clones have been derived and will be used to confirm the functional importance of RALDH2 in the context of ovarian cancer growth suppression.

### P157 IDENTIFICATION OF PUTATIVE OVARIAN CANCER TUMOUR SUPPRESSOR GENES FROM CHROMOSOME (CHR) 11 BY EXPRESSION DIFFERENCE ANALYSIS,

EA Stronach[1], GC Sellar[1], C Blenkiron[1], GJ Rabiasz[1], C Massey[1], DJ Porteous[2], JF Smyth[1], H Gabra[1], [1]ICRF, Medical Oncology Unit, [2]Medical Genetics Section, Department of Medical Sciences, University of Edinburgh Molecular Medicine Centre, Western General Hospital, Edinburgh, EH4 2XU

We and others have previously reported LOH on human chr 11 in ovarian cancer and an association has been observed between adverse clinicopathological variables and LOH on 11p15 and 11q24. Microcell hybrid transfer of chr 11 into the ovarian cancer cell line OVCAR3, which has rearrangements on both 11p and 11q, confers both growth and invasiveness suppression. The invasiveness suppressor has been localised to 11q24; the growth suppressor lies outwith this region.

In order to elucidate the pathways involved in these phenotypes several expression difference analysis methodologies have been combined. The subtractive hybridisation technique cDNA representational difference analysis (cDNA-RDA) has been used to compare the expression profiles of parental and chr 11 recipient cell lines. Sixty independent products have been identified in association with the growth suppressed phenotype. Four of these products have been shown to map to chr 11. Similarly, differential display RT-PCR (DDRT-PCR) has identified one product expressed from chr 11 in association with growth suppression. Hybridisation of three distinct Atlas 1.2 k microarrays (Clontech) have identified 36 products (four of which map to chr 11) showing 3-fold or greater upregulation of expression in chr 11 recipient cells when compared with parental cell lines. These three difference analysis methodologies have complemented each other providing a powerful strategy by which to identify potential components of the chr 11 mediated growth suppression phenotype.

Verification of differentially expressed products identified from all three techniques is underway using LightCycler (Idaho Technology) real-time quantitative RT-PCR. To date, expression differences have been validated for epidermal growth factor receptor (>3 fold), cathepsin D (>2 fold) and tissue inhibitor of metalloproteinase 2 (>2 fold). The role of these and other products verified to be upregulated in the growth suppressed phenotype will ultimately be assessed using both sense and antisense expression constructs and/or small molecule inhibitors where appropriate.

### P158 CHROMOSOMAL ABNORMALITIES IN OVARIAN CLEAR CELL CARCINOMA

Jo Dent[1], T Perren[1], N Wilkinson[1], I Richmond[2], A Markham[1], S Bell[3], [1]St James's Hospital, Leeds, UK, [2]Hull Royal Infirmary, Hull, UK; [3]University of Leeds, Leeds

Ovarian clear cell carcinoma (CCC) accounts for a small but significant proportion of all ovarian cancers and is a distinct clinical and pathological entity. It tends to be associated with poorer response rates to chemotherapy and a worse prognosis. There is little knowledge of any possible underlying genetic changes. Comparative genomic hybridisation (CGH) is a powerful analytical technique that permits identification of regions of chromosomes that have either undergone an increase or decrease in DNA causing genomic imbalance. DNA was extracted from paraffin embedded samples of 18 cases of pure ovarian CCC and analysis for genetic imbalances was carried out using CGH. 17 of the 18 cases (94%) showed genomic alterations. The mean number of changes was 15 (range 2–30) indicating a moderate level of genetic instability. Chromosome deletions were more common than amplifications. The most prominent change involved chromosome 9q deletions in 8 cases (44%) and correlates with other epithelial ovarian cancers. This deletion is currently being confirmed using microsatellite analysis and looking for loss of heterozygosity at 4 separate loci on chromosome 9 and may serve as a useful prognostic indicator. Other frequent deletions involved 10q (22%), 5p (22%), 16 (27%) and 22 (33%). Chromosome gains were most common at 3p (22%), 4q (22%), 13q (22%), 17 (33%) and 18 (33%). CGH and microsatellite marker results will be presented in detail and correlated with patient survival and other clinical parameters including age, stage at presentation and response.

### P159 LOSS OF HETEROZYGOSITY ON CHROMOSOME 11 IS A MARKER OF RECURRENCE IN TCC OF THE BLADDER CANCER.

J Edwards[1], P Duncan[1], JJ Going[2], AD Watters[1] and JMS Bartlett[1], Dept of Surgery[1]./Dept of Pathology[2], Glasgow Royal Infirmary, Glasgow, G31 2ER

Approximately two thirds of patients diagnosed with superficial transitional cell carcinoma (TCC) of the urinary bladder will recur within 2 years. It was recently reported that loss of chromosomes 9 and LOH at 9q34 in primary TCC identifies a subset of patients at high risk of recurrence (Bartlett *et al*. 1998, BJC. 77, 2193–2198 and Edwards *et al*. 2000, BJC. **83** pg 190).

This study explores genetic alterations at chromosomes 4, 8, and 11 as possible predictors of recurrence. A total of 109 tumours were investigate, tumours were obtained from 18 patients who did not recur (NR) and from 29 patients who recurred (REC). Patient DNA was microdissected and extracted from archival normal/tumour sections and analysed for loss of heterozygosity (LOH) at 10 loci. Fluorescent PCR was performed and genotyping carried out on a Perkin Elmer ABI377™ sequencer.

When the level of LOH spanning all informative markers on each chromosome was compared for NR tumours versus primary REC tumours no significant differences were found. When the level of LOH for chromosomal areas and single loci were compared it was noted that 25% (3/12) of NR tumours had LOH at D11S490 compared 81% (13/16) of REC primary tumours ($P$=0.004). An increase of 25% was also noted at FGF3, which resides on the same arm of chromosome 11 as D11S490 although this result was not significant. No other chromosomal region or loci investigated gave significant results when LOH in primary tumours were investigated.

In summary LOH at D11S490 was demonstrated to predict recurrence in a subset of TCC of the urinary bladder patients.

### P160 LOSS OF HETEROZYGOSITY AT A COMMON REGION OF DELETION ON 15q14–q15.2 IN TRANSITIONAL CELL CARCINOMAS.

Rachael Natrajan, Jari Louhelainen, Margaret A Knowles, ICRF Clinical Centre, St. James's University Hospital, Leeds, LS9 7TF

Deletions on chromosome 15 are common in many types of human tumours, including both parathyroid adenomas and colorectal carcinomas, which suggests the presence of potential tumour suppressor genes on chromosome 15. Common regions of deletion identified in these tumour types are 15q15 and 15q21.

In this study we have analysed the incidence of loss of heterozygosity (LOH) on chromosome 15 to ascertain its potential involvement in the development and progression of transitional cell carcinoma (TCC). A panel of 20 polymorphic markers, spanning 15q12-q22, were used to analyse the presence of LOH in 29 TCCs of various stages and grades. LOH was found in 62% (18/29) of informative tumours for at least one marker in the region 15q14-15q15.2. Deletion mapping has defined two minimal regions of deletion; proximally between markers D15S118 and D15S129 at 15q14; and distally between markers D15S968 and D15S659 at 15q15.1-q15.2.

These regions may contain tumour suppressor genes, whose loss, or inactivation, may be associated with TCC tumorigenesis.

### P161 ANALYSIS OF CANDIDATE TUMOUR SUPPRESSOR GENES ON CHROMOSOME 9 IN BLADDER CANCER CELL LINES

SV Williams and MA Knowles, ICRF Clinical Centre, St. James's University Hospital, Beckett Street, Leeds, LS9 7TF, U.K.

Loss of all or part of chromosome 9 in bladder cancer is a common finding, and at least four separate regions of minimal loss have been identified. We have looked at the presence and expression status of candidate tumour suppressor genes from three of these regions in a panel of bladder cancer cell lines. The main aim was to identify suitable cell lines for further functional studies of these genes. The *CDKN2* region at 9p21 was homozygously deleted in several cell lines as shown by fluorescence in situ hybridisation (FISH) and genomic PCR. In all but one cell line (T24) *CDKN2A* (p16 protein) was expressed when the gene was not homozygously deleted. No homozygous deletions of *TSC1* (tuberous sclerosis 1) at 9q34 were detected, and expression was found by RT-PCR in all cell lines. *DBCCR1* (Deleted in Bladder Cancer Candidate Region gene 1) at 9q32-33 was present by FISH and genomic PCR in all but one cell line (SHIM). As in normal urothelium, expression was at the limit of detection by RT-PCR in most cell lines, but high levels were seen in one cell line (253J). This suggests that *TSC1* and *DBCCR1* are rarely inactivated by homozygous deletion or by loss of RNA expression. SHIM, a newly described cell line, is homozygously deleted for both the *CDKN2* region and *DBCCR1*. It will therefore be useful for functional studies of these genes to elucidate their role in bladder tumorigenesis.

### P162 UNDER-REPRESENTATION OF 8p WITH A COMMON BREAKPOINT IN BLADDER CANCER.

J Adams, SV Williams and MA Knowles, ICRF Clinical Centre, St. James's University Hospital, Beckett Street, Leeds, LS9 7TF, U.K.

At least two regions of the short arm of chromosome 8 show deletions in bladder cancer. Deletions are found in 23% of bladder tumours overall and there is a significant association with muscle invasion; 56% of muscle invasive tumours have demonstrable loss of heterozygosity (LOH) in this region. 8p LOH is found in several other tumour types including prostate and colorectal carcinomas, where it is also associated with a more aggressive clinical phenotype. These findings suggest that there may be one or more tumour suppressor genes, associated with an aggressive phenotype, on 8p. To examine the genetic events that occur on proximal 8p and to refine the localisation of the regions of deletion, a panel of bladder cancer cell lines was examined, some of which are predicted to have 8p LOH. Fluorescence *in situ* hybridisation (FISH) analysis was performed using a chromosome 8 paint and a chromosome 8-specific centromeric probe combined with a series of cosmid probes which contain microsatellite markers of interest in 8p12. This has shown overall under-representation of 8p and over-representation of 8q. A common breakpoint has been identified on proximal 8p. It is not clear whether this is the location of a tumour suppressor gene, or whether the target gene is more distal and this common breakpoint is at a position prone to breakage.

### P163 IDENTIFICATION OF TRANSLOCATIONS AND ASSOCIATED EVENTS AT 14q32 IN MULTIPLE MYELOMA,

JAL Fenton, K sibley, and GJ Morgan, Department of Molecular Oncology, Algernon Firth Building, University of Leeds, Leeds LS2 9JT

Cytogenetic techniques have shown that rearrangements of the immunoglobulin heavy chain (IgH) locus at 14q32 can occur in up to 74% of cases of the haematological malignancy multiple myeloma (MM). Recurrent chromosome translocations have been identified in MM cell lines including t(11;14)(q13;q32), t(4;14) (p16;q23), t(14;16) (q32;q23) and t)6;14) (p25;q32). We have isolated and characterised one t(11;14) and six t(4;14) translocations from malignant bone marrow cells of MM patients, using a combination of long range and inverse PCR methods. The breakpoints in these reciprocal translocations are located in the IgH Sμ switch region at 14q32, and a further downstream switch region (either Sγ or Sα) with deletion of intervening DNA. The breakpoint on chromosome 11 for the t(11;14) is currently being characterised. For the six t(4;14) cases, five of the breakpoints on (der) 4 appear to 'cluster' in the promoter and 5´ region of a gene, namely MMSET, in agreement with previously published data. The t(4;14) event, in MM, is associated with fusion of the MMSET and IgH genes on der (4), producing hybrid transcripts initiating from IgH promoters. Interestingly we have isolated one further patient with a breakpoint outside this region and so apparently unable to produce such hybrid transcripts. On the reciprocal der (14) chromosome, the FGFR3 gene is brought into the proximity of strong IgH elements and as a result is over-expressed in the majority, if not all, t(4;14) MM cases. We have detected the overexpression of FGFR3 in RNA obtained from the bone marrow of 7/65 MM patients using an RT PCR approach. Such a level of overexpression (11% of MM) correlated well with several FISH studies where the t(4;14) event has been described in approximately 10% of MM cases. FGFR3 is a putative oncogene which is known to be frequently mutated in bladder and cervical carcinomas at specific hotspots, and there is also evidence of similar mutations in MM cell lines, which overexpress FGFR3. We are currently investigating whether there are any mutations in the FGFR3 positive, primary MM samples. Initial findings have identified one presentation (diagnosis) sample where FGFR3 was not mutated. Interestingly a sample from the same patient taken after post-treatment relapse was found to be mutated. This could conceivably implicate mutations of overexpressed FGFR3 in progression of MM disease.

### P164 COMPARATIVE GENOMIC HYBRIDISATION ANALYSIS OF 40 BREAST CANCERS WITH LONG TERM PATIENT SURVIVAL DATA.

RG Hislop[1], SC Stocks[1], CM Steel[2], M Sales[3], N Pratt[3], D Goudie[3], A Robertson[1], AM Thompson [4]Departments of Molecular and Cellular Pathology[1], Human Genetics[3] and Surgery and Molecular Oncology[4], University of Dundee, DD1 9SY and University of St Andrews School of Biology[2], St Andrews KY16 9TS

Little is known of the precise sequence of genetic events that define malignancy or underlie the clinical course in breast cancer. We have carried out comparative genomic hybridisation (CGH) analysis of 40 primary breast tumours for which 7 year disease recurrence and survival data were available.

Using CGH, the mean number of aberrations per cancer was 9; this comprised an average of 5.5 amplifications and 3.5 deletions per tumour. Three tumours showed no copy number changes and these patients did not suffer disease recurrence. The most common regions of amplification were 1q (67%), 8q (47.5%), 17q (32.5%) and 20q (22.5%). Most frequently deleted areas included 17p (30%), 8p (27.5%) and 19p (25%). These results are consistent with acquisition of a distinctive pattern of large scale (karyotypic) genetic change in malignant brease disease. In a proportion of cancers 8q amplification and 8p deletion co-segregated and this may represent formation of an isochromosome 8q with loss of 8p. Some of the CGH data are consistent with an underlying molecular pathology such as activation of myc (8q24). erb B2 amplification (17q12), cyclin D1 over-expression (11q13) and inactivation of tumour suppressors p53 (17p13) and E-cadherin (16q22). In other instances, such as amplification of 1q and 17q23 and deletion of 8p and 19p, strong candidate genes hve yet to be identified.

While no single change correlated with recurrence or survival, a higher mean number of deletions were seen in the group of cancers from patients who died during the follow-up period than in those from the survivors.

### P165 GENETIC PROFILING OF PRIMARY BREAST CARCINOMAS,

SV Barrett[1], TRJ Evans[1], D Hansell[2], M Rahilly[3] and R Brown[1] [1]CRC Department of Medical Oncology, University of Glasgow, Glasgow, G61 1BD, [2]Department of Surgery, Stobhill Hospital, Glasgow, G21 3UW, [3]Department of Pathology, Stobhill Hospital, Glasgow, G21 3UW

Paired frozen tumour and normal adjacent tissue samples have been collected in an ongoing study from a cohort of 75 patients with primary breast carcinoma. Matched lymphocyte and plasma has been obtained from the same group of patients before and after surgery and chemotherapy. The present aims of this study are to characterise the methylation status of specific loci, together with the presence of allelic imbalance and allelic shift of the DNA from tumours and plasma DNA. The long-term aims will be to correlate genetic alterations with available clinical data.

Using a technique known as methylation specific PCR (MSP), it is possible to investigate the methylation status of specific genes. Using this technique, it has been shown for colorectal and other cancers that a subset of tumours display a methylator phenotype, termed CpG island methylator phenotype (CIMP) in which multiple genes are methylated (1). It is known that certain genes may be methylated in breast cancer but little is known about the CIMP phenotype in breast cancer, its correlation with other genetic markers or its clinical significance. Using MSP, the methylation status of the above cohort of breast tumours is being analysed at the loci ER, BRCA1, HIC1, MINT25 and MINT31. Results so far show tumour methylation in 6/14 (43%) at ER, 1/14 (7%) at BRCA1, 7/14 (50%) at HIC1, 1/14 (7%) at MINT25 and 11/14 (79%) at MINT31.

It is known that tumour cells release DNA into the circulation and this may then be recovered from plasma (2). The tumour DNA extracted from tumour and normal adjacent tissue, lymphocytes and plasma in the above cohort of patients with breast cancer is being analysed for allelic imbalance and allelic shift using PCR with a panel of fluorescent primers. The following loci have been examined: p53, myc, APC, mfd15CA, D2S123, Bat26, Bat40, GGAA4D07, GGAA2E02 in a subset of 24 of the above patients. Results confirm the presence of allelic imbalance in the tumours analysed in up to 34% of cases at specific primers, compared with normal adjacent and lymphocyte DNA. Examples of identical changes detected in plasma DNA compared to tumour DNA have been identified at the loci p53, D2S123, GGAA2E02 and mfd15CA.

1. Toyota M., *et al*. CpG island methylator phenotype in colorectal cancer. *Proc. Natl. Acad. Sci. USA.*, **96**: 8681–8686, 1999.
2. Shaw J.A., *et al*. Microsatellite alterations in plasma DNA of primary breast cancer patients. *Clin. Cancer. Res.* **6**: 1119–1124. 2000.

### P166 CDNA MICROARRAY ANALYSIS OF TRANSFECTED BLADDER CANCER CELL LINES

J Louhelainen, J Gill, N Hornigold, E Pitt, and MA Knowles. ICRF Clinical Centre, St James's University Hospital, Beckett Street, Leeds LS9 7TF

The cDNA microarrays provide a powerful tool for studying changes in gene expression associated with the development and progression of cancer. The cDNA microarray chip technology enables parallel assessment of gene expression for thousands of genes in a single experiment. We have used this technology to compare the expression of 10,000 genes in bladder cell lines transfected with the putative tumour supressor genes *TSC1* and *DBCCR1*. Total mRNA from transfected and untransfected cell lines was isolated. Fluorescently labelled (Cy-3 and Cy-5) single-stranded cDNA targets were generated using SuperScript™ First-Strand Synthesis System and comparative hybridisation was performed with reciprocal replicates. The custom microarrays of 10000 genes were scanned using GSI Lumonics glass slide scanner. Data analysis was performed using QuantArray and GeneSpring software packages. The *TSC1* transfected cell lines had 6 upregulated and 2 downregulated genes whereas the *DBCCR1* transfected cell lines had 18 upregulated and 6 downregulated genes. Full characterisation of these is currently underway. Several of the upregulated genes of the *DBCCR1* transfected cell lines were known calcium-dependent genes. We are currently developing this method to be used with bladder tumour samples.

### P167 ALTERED GENE EXPRESSION IN CISPLATIN-RESISTANT OVARIAN CANCER AS DETECTED BY DIFFERENTIAL DISPLAY AND MICROARRAY

KG Macleod, P Mullen, LMR Gilmour, JF Smyth and SP Langdon. I.C.R.F. Medical Oncology Unit, Western General Hospital, Edinburgh, EH4 2XU

The majority of ovarian cancer patients are treated with cisplatin and development of resistance to this treatment can modify cell signalling responses. The erbB receptors are important regulators of ovarian cancer and signalling via these receptors can produce either growth stimulation or inhibition. We have developed a model system wherein cisplatin treatment has altered responsiveness to ligands of the erbB receptor family. The cisplatin-resistant ovarian carcinoma cell line PE01$^{CDDP}$ was derived from the PE01 cell line by exposure to increasing concentrations of cisplatin and demonstrates a 3-fold level of resistance. While PE01 cells are growth stimulated by $10^{-9}$ M TGFα, NRG1α and NRG1β, the PE01$^{CDDP}$ line is growth inhibited by TGFα and NRG1β but unaffected by NRG1α. TGFα increased apoptosis in PE01$^{CDDP}$ cells but decreased apoptosis in PE01 cells as measured by annexin V using flow cytometry. Comparison of the two cell lines indicated that expression levels of the erbB receptors, extent and time course of tyrosine phosphorylation at the erbB receptors and MAP kinase activation are similar for both lines and could not explain these differing functional responses.

Differential display RT-PCR was used to identify differences in mRNA expression between PE01 and PE01$^{CDDP}$ cells. Products that appeared differentially expressed were excised from acrylamide, re-amplified by PCR and subcloned into pGEM-T Easy vector. These were then sequenced and identified. Expression levels were then quantified by real-time PCR using specific primer pairs targeted to individual cDNAs. Expression of mRNAs for TCP-1, antileukoprotease and PCNA were increased at least 2-fold in PE01$^{CDDP}$ cells relative to PE01 while expression of ZXDA was decreased.

Differential mRNA expression was also analysed using the Clontech Atlas human Cancer 1.2 array. Of 1185 genes analysed, 38 increased by at least 2-fold in PE01$^{CDDP}$ relative to PE01 while 52 decreased. Confirmation of at least a 2-fold change in mRNA levels has been verified by real-time PCR for the following genes: FRA-1, E1AF, MCM2, UFO, HGIF, TRAP-1 and XRCC9 were increased in PE01$^{CDDP}$ cells relative to PE01 while IGFBP-3, PGS1, TRAM, and cytokeratins 4 and 19 were decreased.

These results indicate that growth responses to ligands of the erbB receptors can change in ovarian cancer cells after cisplatin treatment. Multiple differences in mRNA expression have been identified using differential display and a microarray and these are candidate molecules in pathways determining cisplatin-resistance and response to erbB signalling.

### P168 IS N-MYC AND CYCLIN DEPENDENT KINASE PROTEIN EXPRESSION ASSOCIATED WITH POOR PROGNOSIS IN NEUROBLASTOMA?

SL Hankin[1], M Gerrard[2], S Variend[2] & J Lawry[1], [1]Institute for Cancer Studies, University of Sheffield, Sheffield, [2]Sheffield Children's Hospital, Sheffield

Neuroblastoma is characteristically heterogeneous, displaying a great clinical variability ranging from spontaneous regression to highly malignant disease. This immunohistochemical pilot study aims to establish potential protein markers which correlate to disease stage and tumour aggressiveness, which may interpret this diverse clinical behaviour associated with neuroblastoma.

Paraffin wax embedded tissue was obtained from 17 cases of neuroblastoma (1 × stage IVs, 1 × stage I, 2 × stage II, 3 × stage III and 10 × stage IV) and were analysed with a panel of antibodies using standard immunohistochemical techniques. The antibody panel consisted of the cell cycle components CDK4 and CDK6, and apoptotic associated-proteins: Bax, Bcl-2 and CD95. All neuroblastoma cases demonstrated positive staining for the neuroblastoma marker NB84. The prognostic indicators N-Myc and CD44 were also assessed in each neuroblastoma case. This same antibody panel was then used to quantify protein expression using flow cytometry on the two human neuroblastoma cell lines Kelly and SK.N.SH.

Immunohistochemistry revealed that 82% (14/17 cases) showed positive staining for CDK4, compared to 24% (4/17 cases) for CDK6 (1 × stage III and 3 × stage IV). CDK6 positivity could potentially identify a unique sub-set of poor prognosis neuroblastoma cases.

N-Myc oncoprotein was highly expressed in both cell lines, even though SK.N.SH showed no N-Myc gene amplification determined by Fluorescent In Situ Hybridisation. N-Myc oncoprotein was evident in 94% (16/17 cases) of neuroblastoma tested by immunohistochemistry, including the patients with no recorded N-Myc gene amplification.

Immunohistochemical Staining for N-Myc Oncoprotein in Neuroblastoma

| Stage | Negative | Weak | Moderate | Strong |
|---|---|---|---|---|
| IVs, I & II | 0% (0/4) | 75% (3/4) | 25% (1/4) | 0% (0/4) |
| III & IV | 8% (1/13) | 8% (1/13) | 15% (2/13) | 69% (9/13) |

This data suggests that N-Myc protein expression, as opposed to gene amplification, could be a more valuable prognostic factor. This dual approach of both cell cycle control and apoptotic analysis could potentially explain the clinical diversity associated with this childhood neoplasm. Research is funded by Yorkshire Cancer Research.

### P169 WITHDRAWN

### P170 ELUCIDATION OF THE RELATIONSHIP BETWEEN EMAP-II, A TUMOUR-DERIVED PROINFLAMMATORY PROTEIN, AND P43, A COMPONENT OF THE MAMMALIAN MULTISYNTHASE COMPLEX,

YM Heng and JC Murray, Division of Clinical Oncology, School of Clinical Laboratory Sciences, University of Nottingham

The 20kDa proinflammatory polypeptide EMAP-II, found in tumour cell supernatants, is synthesised as a 34kDa precursor. A 1.1 kb cDNA consistent with this precursor form has been cloned. However, recently a cDNA encoding p43, the 43kDa component of the hamster multisynthase complex, was reported to have very high homology with that encoding human EMAP-II. The 1.25 kb p43 cDNA potentially encodes additional N-terminal peptide sequence. Thus, the structure of the gene(s) encoding EMAP-II/p43 remains unclear. We are currently using various genomic and RNA-based techniques to elucidate the origins of the EMAPII and p43 proteins. By database mining, we have identified a human genomic contig of ~117 kb containing the functional EMAP-II gene. The human gene appears to be ~30 kb in size and to comprise 7 exons, with an additional ~20 kb of genomic sequence upstream of the first exon contained within this contig. However, none of the genomic sequence within this contig has any similarity to the first ~170 bp of the hamster p43 cDNA. Thus, it is possible that the 5′ end sequence of the gene may have diverged between human and hamster. Alternatively, the additional N-terminal sequence of the putative p43 protein may be encoded by an additional exon not contained within this contig, but located further upstream. We have simultaneously explored the expression of EMAP-II mRNA by Northern analysis to determine the size and tissue distribution of transcripts. Of 12 normal human tissues examined, EMAP-II RNA was most abundant in the testis. Furthermore, we identified 3 hybridising transcripts of ~1.2 kb, 2.0 kb and 2.6 kb. Similar size transcripts were expressed in a range of tumour cell lines. The smallest transcript (~1.2 kb), which appears to be longer than the cloned EMAP-II cDNA (~1.1 kb), could theoretically encode the 34 kDa EMAP-II protein and potentially a novel human p43 homologue. The significance of the 2 larger hybridising transcripts is therefore unclear. These could represent alternatively or incompletely spliced transcripts. Experiments are currently under way to clone the 5′ end of the EMAP-II cDNA by the 5′ RACE method.

This work is supported by the Cancer Research Campaign

### P171 AN ABSENCE OF GENE AMPLIFICATION AT THE IBD2 LOCUS IN INFLAMMATORY BOWEL DISEASE AND GI CANCERS.

KL BRANSFIELD, PJ Hamlin, P Komolmit, GR Taylor, AF Markham and PA Robinson. Molecular Medicine Unit, University of Leeds, Clinical Sciences Building, St. James's University Hospital, Leeds LS9 7TF, UK

**Background** The inflammatory bowel diseases, Crohn's disease and ulcerative colitis, have both shown linkage to chromosome 12q13 (IBD2). Amplification of genes such as MDM2 and CDK2 at or near to this locus has also been observed in various malignancies, including GI cancers. The increased risk of neoplasia in IBD patients prompted us to determine gene dosage at this region in both IBD and GI cancers.

**Methods** DNA extracted from IBD (n=64) and GI cancer (oral n=31; oesophageal n=13; colon n=39) lesions was subjected to a quantitative multiplex fluorescent polymerase chain reaction to determine gene dosage of individual genes at the IBD2 locus. Primer pairs from two reference genes (UBE2L2 on chromosome 12q12 and CFTR on chromosome 7q31) were multiplexed with a primer pair from a test gene localised to 12q13. PCR products were analysed on an ABI 373 gene scanner and peak ratios were compared between test and control DNA. Glioma, sarcoma and breast tumor derived DNAs were used as controls.

**Results** We confirmed that gene amplification of 12q13 is a frequent event in gliomas and sarcomas. Two deletions and one amplification of the locus under investigation were detected in the IBD cohort but appeared to encompass large regions of 12q. No amplification of the 12q13 region was evident in any of the GI cancers. In contrast, amplification of the SOX4 gene on 6p was demonstrated in 38% and 23% of the same cohort of oral and oesophageal cancer samples, respectively.

**Conclusion** Our data demonstrate that amplification of the defined IBD2 locus is not observed in IBD or GI cancer lesions, in contrast to gliomas and sarcomas, and cannot therefore play a significant role in the progression of disease.

### P172 EXPRESSION OF THE RETAINED INTRON 4 SEQUENCE OF THE CONSTITUTIVELY ACTIVE CCK-2 RECEPTOR IN HUMAN COLORECTAL CANCER,

PA Clarke, DF McWilliams, S Grimes and SA Watson, Academic Unit of Cancer Studies, University Hospital, Nottingham, NG7 2UH

**Background** Recent evidence suggests that a splice variant of the human CCK-2 receptor, with retained intron 4, CCK-BRi4sv, is specifically expressed by human colon cancer when compared to normal resection mucosa (Hellmich et al, 2000, J. Biol. Chem, **275**: 32122–32128).

**Aim** To evaluate the immunoreactivity detected with two polyclonal antibodies (GRE9 and GRE10) raised against different portions of the predicted intracellular intron 4 sequence of the CCK-BRi4sv receptor.

**Methods** Antibodies were raised against two predicted peptide sequences of the retained intron of the CCK-BRi4sv receptor. Western blotting was used to assess their binding to human colorectal cancer cell lines, human colorectal specimens (carcinoma, adenoma and resection margin normal mucosa) and 3T3 cells transfected with the classical CCK-2 receptor.

Cellular localisation of the two antibodies was assessed by immuno-histochemistry. The ability of the antisera to displace$^{125}$ IG17 from 3T3 cells transfected with the CCK-2 receptor was assessed.

**Results** Western blotting with both antisera raised against intron 4 of the CCK-2 splice variant showed an immunoreactive band of approx. 82 kDa on the human colon cell lines C170HM2 and HT29, the CCK-2 transfected 3T3's expressed no specific immunoreactivity. A panel of human colorectal tumour, adenoma and normal specimens expressed a specific band of the same molecular weight with both antibodies. There was little discrimination between resection margin normal and malignancy.

Immuno-histochemical analysis revealed the over-expression of both antisera in tumour and adenoma compared to resection margin normal. The GRE10 antibody was strongly and exclusively expressed in the cytoplasm of the respective cell lines and the epithelial component of human tumour and adenoma specimens, whereas the GRE9 antibody was expressed equally intensely in the cytoplasm and nucleus. $^{125}$IG17 was not displaced by the intron 4 directed antisera.

**Conclusions** These results show the two antibodies display sequence directed specificity to the CCK-BRi4sv and provide evidence for the expression of the intron 4 sequence in the CCK-2 receptor expressed by colon cancer cells

### P173 FUNCTIONAL ANALYSIS OF TSCI IN BLADDER CANCER.

Nick Hornigold, Wendy Kennedy, Margaret A Knowles ICRF Clinical Centre, St James University Hospital, Leeds, LS9 7TF

*TSC1* (Tuberous Sclerosis Complex gene 1) lies in a region (9q34) commonly deleted in bladder transitional cell carcinoma (TCC) of all grades and stages. We have previously shown that TSC1 is mutated in some primary tumours and TCC cell lines. We report that re-introduction of functional TSC1 to a TCC cell line lacking an intact copy of the gene leads to a marked reduction in cellular proliferation. Anchorage independent growth and apoptosis rates are unaffected, but immunofluorescence studies show changes in the distribution of Tuberin, β-4 Integrin, Paxillin and Actin (mAb) within cells expressing TSC1. Transfected cells are being assayed for their tumorigenic potential in an in vivo mouse model. Further studies are being carried out to assess whether the anti-proliferative effects of TSC1 are dosage-dependent and how cell-cell and cell-matrix interactions are affected. Deletion constructs are being used to examine the role of the various protein domains of TSC1. This work will clarify the mechanisms by which TSC1 influences tumorigenesis and indicate whether TSC1 deletion is an initiating event in TCC.

### P174 ISOLATION OF FULL-LENGTH cDNAs OF DIFFERENTIALLY EXPRESSED ESTs SPECIFIC TO ADENOCARCINOMA OF LUNG.

A Yagui-Beltran[1, 2], L You[1], D Jablons[1] [1]School of Medicine, Department of Surgery and Cancer Center, University of California, San Francisco, California, USA and [2]Department of Surgery and Molecular Oncology, University of Dundee DD1 9SY

Lung cancer is the leading cause of cancer death in the United States. Lack of suitable genetic markers for detecting lung cancer at an early stage undermines the overall survival rate of lung cancer patients. We aim to develop novel markers that can be used for detecting molecular changes during the early development of lung cancer.

We have used differential mRNA display methods and high-throughput cDNA gene expression arrays to identify overexpressed mRNAs in the form of expressed sequence tags (ESTs) from stage IA adenocarcinomas in the lung.

A total of 30 ESTs were isolated, of which 27 were novel sequences. The magnitude of overexpression of ESTs was 3 to 12-fold higher in stage IA adenocarcinoma than adjacent normal lung. Five overexpressed ESTs were tested and confirmed using RNA northern blot analysis on a panel of lung cancer samples. To isolate full-length cDNA clones for the overexpressed candidate ESTs, we have used a library-free system for direct cloning. Biotinylated primers designed from EST sequences were hybridized with anchor ligated first strand cDNAs. EST-specific first strand cDNAs were isolated using streptavidin coated magnetic bead capture and amplified using high-fidelity DNA polymerase. The amplified cDNAs were cloned into plasmid vector. We have isolated three full-length cDNA clones for the five overexpressed ESTs to date. The cDNA inserts were 2.5–3.8 kb in size, respectively and the complete cDNA inserts for the clones are now undergoing sequencing. These ESTs and their full-length cDNA clones could be useful candidate gene markers for the early diagnosis of lung cancer.

### P175 CYTOCHROME P450 1B1 (CYP1B1) EXPRESSION IN HUMAN CERVICAL INTRAEPITHELIAL NEOPLASIA

TTV Hoang, MD Burke, PC Butler, A Seaton, LA Stanley, School of Pharmacy and Pharmaceutical Sciences, De Montfort University, Leicester LE1 9BH

Cervical cancer is the second most common cancer in women worldwide (Schoell *et al*., 1999). Limitations of the present screening method, the Papanicolaou (Pap) test, have lead to the requirement for a more objective method of screening.

Several studies have indicated that the cytochrome P450 isozyme, CYP1B1, may be a marker for tumourigenesis (McKay *et al*., 1995; Murray *et al*., 1997). Our studies aim to determine the significance of CYP1B1 as a marker of neoplasia and as a possible indicator of neoplastic risk in the cervix. Results from a pilot study on cervical biopsy samples indicate that there is differential expression of CYP1B1 in the different stages of cervical intraepithelial neoplasia (CIN). This communication reports on the results of this study.

A mouse $IgG_1$ monoclonal antibody (LDS101), raised against the predicted sequence of the CYP1B1 C-terminus coupled to Keyhole Limpet Haemocyanin, was used to study the expression of the CYP1B1 protein in normal and preneoplastic cervical specimens by immunohistochemistry.

In normal smears CYP1B1 expression was not detectable. In contrast, in biopsy samples diagnosed with CIN 1 to CIN 3, expression was detectable in abnormal cells within the squamous epithelium. The pattern of staining seen suggests that CYP1B1 expression corresponds with increasing stages of CIN (Table 1).

**Table 1** Summary of pilot study results showing number of samples stained positive for CYP1B1 and the type of staining pattern observed for the different stages of CIN.

| CIN Stage | Number of Samples | Number of Positive Samples | CYP1B1 Staining Pattern |
|---|---|---|---|
| CIN 1 | 9 | 8 | Staining of lower 1/3 of epithelium |
| CIN 2 | 10 | 10 | Staining of lower 2/3 to whole of epithelium |
| CIN 3 | 10 | 10 | Staining of majority to whole of epithelium |

We suggest that CYP1B1 may be a potentially useful marker for detection of CIN in routine cervical screening. A study of patients undergoing colposcopic examination for cervical abnormalities is currently underway to investigate these results further.

1. McKay, J.A. *et al*., (1995). *FEBS Letters*, **374**, 270
2. Murray G.I., *et al*., (1997). *Cancer Research*, **57**, 3026
3. Schoell W.M. *et al*., (1999). *Seminars in Surgical Oncology*, **16**, 203

### P176 IS DDX1 AN ONCOGENE? HOW DOES ITS PATTERN OF EXPRESSION RELATE TO ITS AMPLIFICATION STATUS?

JL Elsden[1], RM Kenyon[1], RE George[2], R Godbout[3], ADJ Pearson[4] and J Lunec[1], Cancer Research Unit[1] and Department of Child Health[4], University of Newcastle, Childrens Hospital, Harvard[2], University of Alberta, Canada[3]

Neuroblastoma (NB) is the third most common childhood cancer, accounting for 15% of all childhood cancer deaths. A major problem with the treatment of neuroblastoma is that diagnosis often occurs after metastasis, leading to rapid tumour progression and a fatal outcome. Once diagnosed the behaviour of neuroblastoma is unpredictable with the likelihood of tumour progression varying widely according to tumour stage and age at diagnosis.

The *MYCN* gene is amplified in 20–30% of NB tumours and this amplification is strongly correlated with poor prognosis. *DDX1* maps to the same chromosomal position as *MYCN* (2p24) and is co-amplified with *MYCN*, in approximately 50% of cases. *DDX1* is a member of the family of genes that code for proteins containing the D-E-A-D box protein motif and is a putative RNA helicase. Patients with co-amplification of both genes have a shorter post-treatment relapse period compared with patients with MYCN amplification alone.

Cloned *DDX1* cDNA, was stably introduced into NIH3T3 cells using calcium-phosphate mediated transfection methods. Initial selection for successfully transfected clones was performed using G418 resistance. From ten G418 resistant clones, four clones were isolated that showed increased levels of DDX1 protein expression by Western blotting. All four clones reproducibly showed both increased growth rates and greater ability to grow in soft agar compared with the parental cell lines or cells transfected with PCR3.1 vector alone. *DDX1* was also successfully transfected into the neuroblastoma cell line SH-EP and these cells are being tested for altered behaviour, including differentiation status and oncogenicity. Initial experiments indicates that DDX1 transfected SH-EP cells may have a faster growth rate than the negative controls. Evidence so far suggests that DDX1 has an oncogenic role in its own right.

An initial study of DDX1 and MYCN protein expression in 22 neuroblastoma tumour samples has shown considerable variation in protein expression, including increased protein levels in 5 samples, which have neither *MYCN* nor *DDX1* amplification. This indicates that DDX1 expression varies in NB and high expression can result from mechanisms other than *DDX1* amplification.

Future work will include co-transfection of both *DDX1* and *MYCN* into NIH3T3 and SH-EP cells. These cells will then be used as a model to determine the role of co-amplification of *MYCN* and *DDX1*.

### P177 IDENTIFICATION OF NOVEL CODING EXONS WITHIN THE NEUROBLASTOMA AMPLIFIED GENE, NAG.

DK Scott[1], ADJ Pearson[2], J Lunec[1]. [1]Cancer Research Unit, University of Newcastle upon Tyne, NE2 4HH, UK; [2]Dept of Child Health, Royal Victoria Infirmary, Newcastle upon Tyne, UK

Amplification of the *MYCN* oncogene in neuroblastoma tumours is predictive of a poor outcome. However, within this subset of tumours there is heterogeneity of phenotype, with instances of individuals remaining alive and disease free with minimal or no treatment. The amplified unit of DNA varies in size, and can be up to 1 Mb long. Other genes situated at the *MYCN* locus may be coamplified with the oncogene, affecting tumour phenotype. Identification of these could ultimately lead to increased accuracy of prognosis and give targets for new therapies.

The *NAG* gene has been independently isolated in two laboratories using genome scanning techniques. The gene was subsequently found to be coamplified with *MYCN* in neuroblastoma[1,2]. Wimmer *et al* reported the presence of a 4.5 kb *NAG* gene transcript in neuroblastoma cell line RNA[1]; Fruhwald *et al*, in contrast, performed Northern blots on normal tissue RNA and obtained a signal of about 7 kb[2]. In this laboratory, Northern blots on neuroblastoma cell line RNA also indicated the presence of a 7–8 kb transcript. The aim of this research was therefore to identify and characterise the extra 3 kb of mRNA sequence.

Alignment of the 4.5 kb *NAG* cDNA sequence with genomic DNA sequence from the Human Genome Project (HGP) indicated an unusually large 150 kb gap between exons 4 and 5 of the gene. RT-PCR on IMR-32 neuroblastoma cell line RNA confirmed the presence of additional expressed sequence between exons 4 and 5. The 7.5 kb PCR product has now been cloned and is being characterised. In parallel, a *NAG* gene probe was used to identify positive clones within cDNA libraries from normal tissues. A 5 kb cDNA clone was isolated from a skeletal muscle library and the sequence obtained by automated fluorescent sequencing. Comparison of this with the HGP genomic clones indicated the presence of 9 novel exons within the gap between the previously designated exons 4 and 5. Northern blots show the presence of a 7 kb transcript in skeletal muscle[2]. Work continues to establish the biological role of the full-length *NAG* gene.

1. Wimmer K, Zhu XX, Lamb BJ, Kuick R *et al* (1999) *Oncogene* **18**: 233
2. Fruhwald MC, O'Dorisio MS *et al* Rush LJ (2000) *J Med Genet* **37**: 501

### P178 ECTOPIC EXPRESSION OF REG AND ITS PUTATIVE RECEPTOR EXTR-1 IN COLORECTAL CANCER.

IA Hunter, SM Farmery, K Bransfield°, PA Robinson°, DS O'Riordain, PJ Guillou, Academic Department of Surgery, St. James's University Hospital, and °Molecular Medicine Unit, Leeds University

**Aims** *EXTR-1* has recently been reported to act as a receptor for REG (regenerating protein), a protein that is implicated in the control of cell proliferation and apoptosis. *REG* expression in early stage colorectal carcinoma is associated with an increased incidence of disease recurrence. The aims of this study were to investigate the expression of *EXTR-1* and *REG*, and their relationship to tumour stage, in colorectal cancer.

**Methods** RT-PCR was used to establish the presence of *EXTR-1* and *REG* transcripts in 28 colorectal cancer and matched normal mucosa specimens. Alterations of *EXTR-1* at the genomic level were analysed using quantitative fluorescent-labelled multiplex PCR of DNA. Tumour stage was determined from hospital pathology records.

**Results** *REG* expression was in keeping with previously reported levels being detected in 18 of 28 (64%) cancers versus 8 of 28 (28%) matched normal mucosa specimens ($\chi^2$ $P$<0.05). *EXTR-1* transcripts were also more frequently present in tumour tissue, being detected in 23 of 28 (82%) cancers versus 9 of 28 (32%) matched mucosa specimens ($\chi^2$ $P$<0.05). Of the 18 *REG* positive tumours 16 also expressed *EXTR-1*, although this relationship was not significant. There was no relationship between expression of either gene and tumour stage. Genomic analysis of *EXTR-1* detected no amplification or deletion of the gene suggesting that increased expression is regulated at the transcriptional level.

**Conclusion** Expression of both *REG* and its putative receptor *EXTR-1* are increased in colorectal cancers. EXTR-1-REG interactions may contribute to the development of disease recurrence associated with *REG* expression in early stage disease.

**Abbreviations** *EXTR-1*, Exostoses related gene 1; *REG*, regenerating gene; RT-PCR, reverse transcription and polymerase chain reaction amplification; $\chi^2$, Yates' corrected Chi-squared test.

### P179 COEXPRESSION OF PAX6 AND GLI1 IN DESMOPLASTIC MEDULLOBLASTOMA

R Przkora[1], JC Tonn[2], N Sörensen[2], OD Wiestler[1], & T Pietsch[1], [1]Dept of Neuropathology, University of Bonn, [2]Dept of Neurosurgery, University of Würzburg

Medulloblastoma is the most frequent malignant brain tumor of childhood. The *classic* and the *desmoplastic* variant display markers of different cerebellar cell lineages and show a distinct pattern of localization in the cerebellum. Neuronal precursor cell proliferation and differentiation in the cerebellum is controlled by *Sonic hedgehog* and homeobox genes.

**Procedures** In this study we analyzed the mRNA transcripts of *PAX5*, *PAX6* and *GLI1* as target gene of *SHH* in 20 *classic* MBs (cMB), 13 *desmoplastic* MBs (dMB), 2 *medullomyoblastomas* (MMB), 2 adult and 2 fetal cerebelli. A competitive RT-PCR with external *PAX5*, *PAX6* and *GLI1* gene standards was established for quantitative mRNA analysis compared to *$\beta$2-microglobulin*.

**Major findings** We detected *PAX5*, *PAX6* and *GLI1* transcripts in all variants, including the adult and fetal cerebelli. The dMB exhibited significantly higher *PAX6* (Kruskal-Wallis, $P$ =0.0032) and *GLI1* ($P$=0.0005, K-W) m-RNA levels. The expression signal of *PAX6* was significantly lower in the 8 MB cell lines compared to the tumor samples ($P$ = 0.001, Kruskal-Wallis). There was a significant correlation between the *PAX6* and *GLI1* signal in dMB (Spearman-Rank, $P$ = 0.009).

**Conclusion** Our results are supporting the hypothesis, that the dMB arises from the external granule cell layer (EGL). The correlation of *PAX6* and *GLI1* as target of the *SHH* signal transduction cascade in the desmoplastic variant suggests an interaction of these developmental control genes in the pathogenesis of dMB.

### P180 MUTATION OF FGFR3 IN TRANSITIONAL CELL CARCINOMA

Kathryn Sibley[1,2], Wendy Kennedy[1] and Margaret Knowles[1]. [1]ICRF Clinical Centre, St James University Hospital, Leeds, LS9 7TF, [2]Department of Haematology and Oncology, University of Leeds, Leeds, LS2 9JT

4p16.3 has previously been identified as a region of non-random loss of heterozygosity (LOH) in transitional cell carcinoma (TCC), suggesting the presence of a tumour suppressor gene. One candidate within this region is fibroblast growth factor receptor 3 (*FGFR3*). We investigated the frequency and nature of *FGFR3* mutations in a panel of TCCs and cell lines and studied the possible link between mutation and LOH on 4p16.3.

*FGFR3* coding sequence from 63 TCCs of various stages and grades, and 18 cell lines was analysed by fluorescent SSCP. 31/63 tumours had previously been assessed to have LOH at 4p16.3. 26/63 tumours (41%) and 4/18 (22%) of the cell lines had missense mutations in *FGFR3*. All mutations detected in our panel have been reported in the germline where all apart from one cause lethal conditions. Tumours with and without LOH at 4p16.3 had mutations in *FGFR3* suggesting that these two events are not causally linked.

Subsequently, we investigated the frequency of *FGFR3* mutation in a panel of 125 tumours and 13 cell lines from various other organs (stomach, rectum, colon, prostate, ovarian, breast, brain, renal or cervical tumours). We found only one mutation in 1/28 cervical carcinomas analysed suggesting that these mutations are important only in the development of certain types of tumour.

In addition to mutations of *FGFR3*, we have also detected alterations in the expression of *FGFR3* splice variants (IIIb and IIIc) at the mRNA level in TCC cell lines. It is possible therefore that both mutation and alterations of *FGFR3* expression contribute to bladder tumour development.

### P181 A GERMLINE MUTATION IN EXON 5 OF p53; DISEASE-CAUSING OR NOT?

Jodie Rutherford[1], Diana Barnes[2], Diana Eccles[3], Paula Duddy[1], Richard Camplejohn[1]: [1]Richard Dimbleby Dept., St Thomas' Hosp., SE1 7EH, 2ICRF Clin Oncol. Unit, Guy's Hosp., SE1 9RT; 3Wessex Clin. Genetics Service. Princess Ann Hosp., Southampton, SO16 5YA

The *p53* gene from a patient with Li-Fraumeni like syndrome was sequenced and a germline mutation was found in exon 5. This was a point mutation resulting in an amino acid change from arginine to histidine. Initially two functional assays were carried out using peripheral blood lymphocytes from the patient, the apoptotic assay (Camplejohn et al. *Br J Cancer* 72: 654, 1995) and the FASAY (Flaman et al. *PNAS* 92: 3963, 1995). The apoptotic assay measures the apoptotic response of PBL after treatment with 4 Gy irradiation and the FASAY is a yeast-based assay investigating transactivational ability of p53. Both of these assays yielded wildtype results, the apoptotic assay giving a 41% increase in apoptosis after irradiation and the FASAY giving only 2% red colonies (any value under 10% is considered wildtype). The mutation was manufactured by site directed mutagenesis and used in four different FASAYs, investigating the ability to transactivate *bax, p21, PIG3* and *RGC*. The results for all these experiments were wildtype i.e. between 2 to 4% red colonies. The mutation was cloned into a mammalian expression vector, pC53-SN3 and transfected into Saos-2 cells to investigate induction of apoptosis, suppression of colony growth and transrepression. The mutant p53 had 90% of the ability of wildtype p53 to induce apoptosis and the suppression of colony growth assay showed that the mutant and wildtype p53s had similar abilities to prevent the growth of Saos-2 colonies. For transrepression, the mutant protein had 70% of the ability of wildtype to repress the SV40 promoter, which may represent a deficiency in transrepression. Despite this finding, the functional assays used so far have failed to detect a clear defect in the mutant protein's biological activities. The induction of apoptosis experiments were carried out for a second time, using only small amounts of plasmid DNA for transfection, in order to investigate the effect of lower level expression of the mutant protein. Even at these lower levels, this mutant could still induce apoptosis at levels that were not significantly different to wildtype p53. Immunoprecipitations and the yeast-2-hybrid assay showed the mutant could bind to Mdm-2 protein. Therefore, no clear functional defects have been found in this protein, although the mutation appears to segregate with disease in the patient's family (five breast tumours, a childhood ALL, a lymphosarcoma and an adenocarcinoma in three generations) and has been reported in another LFS family. An inducible expression system has been set up with this mutant and wildtype p53 in Saos-2 cells. This system is being used along with microarray technology to try to confirm the presence or absence of a transrepression defect.

### P182 THE YEAST FUNCTIONAL ASSAY FOR P53 MUTATION: COMPARISON WITH THE HOT TECHNIQUE AND CLINICAL/PATHOLOGICAL PARAMETERS

A Yagui-Beltran[1], TR Hupp[2], AM Thompson[1], Departments of [1]Surgery and Molecular Oncology and [2]Molecular and Cellular Pathology, University of Dundee DD1 9SY

The assessment of p53 function in breast cancer is controversial. Mutational studies of p53 (usually examining only part of the gene) have produced a wide range of p53 mutation frequencies; expression studies, particularly using immuno-histochemistry, are open to varied interpretation. We therefore sought to compare a comparatively new functional assay of p53 with an established technique for mutation detection.

Total ribonucleic acid (RNA) was extracted from 16 primary breast cancers and 2 benign breast tissue samples from unrelated patients. Using reverse transcription polymerase chain reaction (RT PCR) cDNA was amplified and transfected into S cerevisiae and grown on selectable media. The human p53 inserted into the yeast activates a cotransfected ADE2 gene giving white colonies (wild type p53) or red colonies (mutant p53) if the p53 is functionally inactive. A spontaneous mutation rate due to PCR infidelity of 5% may occur, producing some red colonies even for wild type tissue. A cut off of 10% was therefore used to differentiate between mutant (>10%) and wild type (<10%) p53. The well established Hydroxylamine Osmium Tetroxide (HOT) technique was used to examine exons 5–9 of the p53 gene in the same 18 samples.

Fifteen of the sixteen cancers showed mutation according to the Yeast Functional Assay; both benign samples expressed wild type p53. Four of the cancers exhibited mutation using the HOT technique, cancers which grew 24%, 36%, 50% and 84% red colonies respectively.

There was no association between the percentage of red colonies using the functional yeast assay and clinical or pathological parameters. These data suggest that the functional yeast assay may indeed be the key p53 assay for future breast cancer trials such as EORTC-10994.

### P183 THE DEPENDENCE OF P53 INDUCTION AND ACTIVITY ON POLY (ADP-RIBOSE) POLYMERASE-1

PA Jowsey, G Farnie, BW Durkacz, and J Lunec, Cancer Research Unit, University of Newcastle upon Tyne, UK

PARP-1 is an enzyme involved in the immediate cellular response to DNA damage. Studies have suggested a functional relationship between PARP-1 and another DNA damage response protein, p53. This study investigated the possible function of PARP-1 as an upstream regulator of p53 and mdm2. PARP-1 +/+ and PARP-1 –/– mouse embryo fibroblasts (MEFs) were established from mouse colonies obtained from Gilbert de Murcia (Ecole Superieure be Biotechnologie de Strasbourg). The genotype and PARP-1 expression in these cells was confirmed using Southern blotting and Western blotting, respectively. The PARP-1 +/+ MEFs were found to contain constitutively high levels of p53, which was not induced in response to DNA damage. cDNA sequencing revealed that the PARP-1 +/+ cells contained a mutant p53, with an Asp-Glu substitution at codon 278. This mutation is in a conserved region of the DNA binding domain of p53 and is consistent with the inability to induce mdm2 or $p21^{waf-1}$. However, the PARP-1 –/– cells did exhibit a p53 induction, which in turn induced mdm2 and $p21^{waf-1}$ 3–6 hours after IR or UV treatment. Permeabilised cell assays measuring PARP activity by the incorporation of radiolabel from [$^{32}$P NAD] into ADP-ribose polymers showed that PARP-1 –/– cell lines possessed 5% residual PARP activity which cannot be attributed to PARP-1. This activity was independent of DNA ends, present as synthetic oligonucleotides to simulate DNA breaks and could be completely abolished by treatment of cells with a potent PARP inhibitor (PI) with a $k_i$ <6nM. In contrast PARP-1 +/+ cells exhibited PARP activity that was dependent upon DNA ends. The PI also drastically reduced luciferase production from a p53 dependent promoter (*MDM2* P2) in a luciferase gene reporter system in HCT 116 human colorectal cells. This suggests a role for PARP in p53 transcriptional activity. Work is in progress to identify the PARP protein responsible for the activity in the PARP-1 –/– MEFs and, using both PARP inhibitors and the PARP-1 –/– cells, to investigate potential roles for alternative PARPs in the regulation of p53 and mdm2.

### P184 GENE DOSAGE OF THE CDKN2 REGION USING REAL TIME QUANTITATIVE PCR IN BLADDER CANCER

Joanne S Aveyard, Emma J Chapman and Margaret A Knowles, ICRF Clinical Centre, St James University Hospital, Leeds, LS9 7TF

Homozygous deletions have been identified at 9p21 in bladder cancer cell lines and tumours. Previously we have shown that CDKN2A (p16) or both p16 and CDKN2B (p15) were deleted in many cases. Subsequently, p14ARF has been identified as an additional transcript from this region with its first exon between p15 and p16. The individual contribution of these three genes to bladder tumour development has not yet been fully investigated.

Recently, it has been discovered in a melanoma-astrocytoma syndrome family, using real time quantitative PCR, that both copies of p16 are retained (gene dosage ratio = 1) and one copy of p14ARF is deleted (gene dosage ratio = 0.5). This suggest that deletion of p14 ARF may be critical in some cases and not p16. We have therefore, carried out high density homozygous deletion mapping of this region in a series of bladder tumour cell lines including 10 newly established cell lines.

Homozygous deletions are difficult to detect by conventional PCR in tumour samples due to the presence of contaminating normal cells. We have used Real time quantitative PCR to work out gene dosage ratios and improve the sensitivity of detection of homozygous deletions of p16, p15 and p14ARF in TCC cell lines and tumours. For the first time this allows the precise mapping of homozygous deletion breakpoints at 9p21 in DNA samples from primary tumours.

### P185 RELATIONSHIP BETWEEN ACTIVE MMP-2 AT THE PROTEIN LEVEL AND A DECREASE IN MMP-2 EXPRESSION AT THE GENE LEVEL IN VITRO AND IN VIVO

R Asher-Dean*, HM Collins, WJ Speake and SA Watson, Academic Unit of Cancer Studies, University Hospital, Nottingham, NG7 2UH

**Background & Aims** Over expression of various matrix metalloproteinases (MMPs) have previously been speculated to correlate with tumour progression in a variety of cancers. The aim of this study was to investigate the activation of proMMP-2 at the protein level and correlate this with the expression of MMP-2 and MT1-MMP at the gene level in a human fibrosarcoma cell line transfected with MT1-MMP and three colorectal adenoma cell lines, in vitro and in vivo.

**Methods** Gelatin zymography, western blotting and real-time PCR were used to investigate the protein and gene expression of MMP-2 and MT1-MMP in vitro and in vivo in two human fibrosarcoma cell lines: HT1080 wild type; HT1080 transfected with MT1-MMP, and three adenoma cell lines: RGC2/42 (non tumourigenic, full length APC); AA/C1 (non-tumourigenic, truncated APC, heterozygous for codon 12 mutation in *K-ras*); AA/C1/SB10C (tumourigenic, truncated APC, homozygous codon 12 mutation in *K-ras*), obtained from Professor C. Paraskeva.

**Results** The human fibrosarcoma cell line transfected with MT1-MMP produced a significant 1.5 fold increase in active MMP-2 (62kDa) and a 6 fold decrease in proMMP-2 (72kDa) compared with that of the HT1080 wild type cells at the protein level in vitro. This increase in activation was also observed by western blotting, with an 8 fold increase in the proteolytic form of MT1-MMP (43kDa) which is associated with MMP-2 activation in the transfected cell line. This cell line showed a significant decrease in MMP-2 at the mRNA level ($P<0.01$) and an increase in MT1-MMP expression ($P<0.01$). In vivo, both cell lines produced active MMP-2 at the protein level and this correlated with a decrease in mRNA expression of MMP-2 in the wild type cells ($P<0.01$). The two APC truncated cell lines (AA/C1 and AA/C1/SB10C) had significantly higher levels of MT1-MMP mRNA expression ($P<0.01$) in vitro compared with that of the full length APC cell line (RGC2/42) but only AAC1 had high levels of MMP-2 ($P<0.01$). Zymography showed slight activation of MMP-2 in all three cell lines but western blotting only detected the proteolytic fragment of MT1-MMP in the tumourigenic AA/C1/SB10C. When grown in vivo, *AA/C1/SB10C* had low levels of MMP-2 gene expression and high levels of active MMP-2 at the protein level.

**Conclusion** We have shown that an increase in MT1-MMP mRNA correlates with an increase in active MMP-2 at the protein level in human fibrosarcoma cells in vitro. This increase in active MMP-2 produces a decrease in MMP-2 expression at the gene level in vitro and in vivo, suggesting that there is some form of negative feedback occurring. This decrease in MMP-2 mRNA was also observed in vivo with the tumourigenic adenoma cell line AA/C1/SB10C. The lack of the 43kDa fragment of MT1-MMP in the AA/C1 compared with that of the tumourigenic cell line suggests a different intracellular processing of MT1-MMP.

### P186 INVESTIGATION OF THE REGULATION OF CYCLIN D1 DEGRADATION BY A PHOSPHORYLATION DEPENDENT MECHANISM

Simon R Stockwell, Craig McAndrew, Michelle D Garrett, Institute of Cancer Research, 15 Cotswold Road, Sutton, Surrey SM2 5NG

Over-expression of cyclin D1 has been observed in numerous examples of cancers, particularly those of the head, neck and breast. In the light of the recent reports connecting cyclin D1 over-expression with oncogenesis, the post-transcriptional mechanisms regulating the destruction of this protein are the focus of this study.

Phosphorylation of cyclin D1 at threonine 286 (T286) has recently been shown to initiate its ubiquitin-dependent degradation. Although glycogen synthase kinase-3 beta (GSK3β) immunoprecipitated from mouse NIH3T3 cells has been found to be capable of this modification in vitro, it has yet to be shown if it is the sole activity that can perform this phosphorylation.

To detect such an activity in human cells a novel rabbit polyclonal antibody has been raised against T286 of human cyclin D1 in its phosphorylated state. Specificity of the affinity-purified phospho-antibody has been demonstrated by ELISA and phospho-peptide competition experiments. This novel reagent has been successfully employed in conjunction with a non-radioactive kinase assay to detect T286-kinase activity in crude cell extracts from several human cancer cell lines (HeLa, MCF7, U20S).

Although GSK3β has been detected in all these cell lines, the identity of the T286-kinase(s) has yet to be shown. A comparison of the sensitivity of lysate-based T286-kinase activity and recombinant GSK3β to lithium and hymenialdisine (GSK3β inhibitors) has been performed in an attempt to rule out endogenous GSK3β. This has hinted at a differential sensitivity in the lysates when compared to purified GSK3β. We are now in the process of fractionating the putative T286 kinase activity(s) by column chromatography with a view to identification.

### P187 THE PROGNOSTIC INFLUENCE OF BCL-2 IN MALIGNANT GLIOMA; A MULTIVARIATE ANALYSIS

FE McDonald[1], JW Ironside[2], A Gregor[2], E Wyatt[2], M Stewart[3], R Rye[3], J Hadley[4], H Potts[4], [1]Northern Centre for Cancer Treatment, Newcastle General Hospital, Westgate Road, Newcastle upon Tyne. NE4 6BE, [2]Departments of Pathology and Clinical Oncology, Western General Hospital, Crewe Road, Edinburgh. EH4 2XU, [3]ICRF Trials Office, Western General Hospital, Crewe Road, Edinburgh EH4 2XU, [4]ICRF Medical Statistics Group, Institute of Health Sciences, PO Box 777, Headington, Oxford, OX3 7LF

The bcl-2 gene is one of a complex group of genes which contrl programmed cell death. Bcl-2 acts to extend cell survival by blocking apoptosis, and thereby may influence tumour prognosis. This study of 187 high grade gliomas reviews clinicopathological prognostic features and the relationship to bcl-2 expression. Bcl-2 immunostaining was assessed in 159 specimens from these patients, by scoring systems of 0 to 3 for intensity of scoring and proportion of cells staining. Age, histology, pre and post-operative performance status were found to be strongly predictive of survival (logrank test $P<0.0001$). The type of surgery performed did not influence survival in this group of patients. The expression of bcl-2 had a significant relationship with survival (univariate Cox model $P=0.0302$, hazard ratio 0.8, 95% confidence interval 0.65–0.98), with increased staining associated with improved survival. Multivariate analysis showed performance status, histology and proportion of cells staining for bcl-2 to be independently predictive of survival. Bcl-2 staining was not related to histological grade of tumours.

**Keywords:** high grade glioma; bcl-2; multivariate analysis

### P188 INHIBITING THE MDM2-P53 INTERACTION USING LOW MOLECULAR WEIGHT COMPOUNDS

G Farnie[1], H Atkins[1], AH Calvert[1], NJ Curtin[1], BT Golding[2], RJ Griffin[2], I Hardcastle[2], M Kitching[2], DR Newell[1], J Northen[2], R Reid[2], J Lunec[1], Cancer research Unit[1], Dept Chemistry[2], University of Newcastle upon Tyne, UK

Levels of p53 within normal cells are regulated via an autoregulatory feed back loop involving MDM2 and p53, in which p53 both activates the transcription of *MDM2* and is also subject to inactivation and downregulation by binding of the MDM2 protein. Prevention of the interaction between wild type p53 and MDM2 has been shown to release p53 activity, resulting in cell cycle arrest or apoptosis. Libraries of low molecular weight compounds have been screened for antagonists of p53-MDM2 binding, using cell free binding assays and both functional and cytotoxicity studies with intact cells.

A gene reporter assay was developed using a luciferase reporter gene plasmid construct (pGL3-P2) with the p53-dependent *MDM2* P2 promoter inserted. Isogenic paired cell lines, A2780 ovarian (wild type p53) and CP70 (cisplatin resistant p53 heterozygous mutant), colorectal HCT 116 (wild type p53) and HCT 116 N7 (E6 degraded), and the SaOs-2 (p53 null) osteosarcoma cell line, were transiently transfected with pGL3-P2. Results with A2780 and HCT 116 wild type cell lines showed that the P2 region had strong basal promoter activity, 500 and 780 fold increased from pGL3 vector only controls, respectively. P2 promoter activities were 150 and 390 fold lower in the mutant/degraded p53 cell lines CP70 and HCT116, compared to the wild-type p53 cell lines, but not completely abolished. No significant difference in promoter activity was observed compared with vector only negative controls in p53-null SAOS cell line. In addition after exposure to γ-irradiation (6.3 Gy) p53 was induced in transfected wild-type p53 cell lines with a corresponding increase in luciferase/P2 activity with time. These results showed that the P2 promoter activity was being driven in a p53 dependent manner. The effects of potential inhibitors of the MDM2-p53 interaction were studied using the gene reporter assay.

From the initial series screened, three compounds (NU8001, NU8006 & NU8009) showed the highest levels of activity in both binding and cytotoxicity assays and NU8001 induced p53-dependent luciferase activity in the reporter assay. Growth inhibition of the A2780 (wild type p53) cell line using NU8001, NU8006 and NU8009 was also greater than for the CP70 (heterozygous mutant) cell line (e.g. for NU8001, $Gl_{50}$ = 22μM and 34μM, respectively). Further compounds are being tested to determine structure activity relationships for antagonism of the MDM2-p53 interaction.

### P189 MDM2 BLOCKS MTBP INDUCED GROWTH ARREST BY RELOCALISING MTBP TO THE CYTOPLASM

D Marcus Gore[1], Nikolina Vlatkovic[1], David Spiller[3], Michael White[3], Andrew Dodson[2], Robert Sutton[1] and Mark T Boyd[1], [1]Depts of Surgery, [2]Pathology and [3]School of Biological Sciences, University of Liverpool, Liverpool L69 3GA

The MDM2 protein, through its interaction with p53, plays an important role in the regulation of the $G_1$ checkpoint of the cell cycle. In addition to binding to and inhibiting the transcriptional activation function of the p53 protein, evidence is accumulating for the existence of p53 independent or downstream activities of MDM2. In an attempt to investigate these MDM2 pathway/s we have continued our studies of a novel MDM2 binding protein *M*dm *T*wo *B*inding *P*rotein (MTBP) that we have identified. MTBP induces a p53 independent $G_1$ arrest and this is abrogated by ectopic expression of MDM2. Our published work (Boyd et al. (2000) J. Biol. Chem. 275; 31883–31890) has suggested that this might be the result of direct binding of MDM2 to MTBP but further studies were needed to determine this.

Using a polyclonal anti-MTBP peptide serum we have found that MTBP is a predominantly nuclear protein that co-localises with MDM2. Moreover, under conditions in which MDM2 blocks the cell cycle arrest induced by MTBP, we find that MTBP is re-localised with MDM2 to the cytoplasm. This clearly supports a hypothesis in which MDM2 regulates the activity of MTBP by modulating the localisation of MTBP. Using an inhibitor of nuclear export, leptomycin B, we have found that a complex containing MDM2 and MTBP can be retained in the nucleus and thus we conclude that binding of MDM2 to MTBP in the nucleus (most likely) leads to re-localisation of this complex to the cytoplasm.

Interestingly, we have observed that MTBP may also re-localise MDM2 from the nucleus to the cytoplasm and thus the actual physiological nature of this interaction might be to regulate MDM2's ability to control p53 activity. To investigate the physiological context in which this interaction may normally act we have studied the expression of MTBP by immuno-histochemistry. The results of these studies will also be presented.

Our results support our earlier investigation in suggesting that MTBP can be added to the list of growth regulatory proteins that are genuine targets of MDM2 and moreover, that are re-localised to the cytoplasm by MDM2.

### P190 EXPRESSION OF THE GASTRIN/CHOLECYSTOKININ-B RECEPTOR IN RELATION TO THE CELL CYCLE,

SA Evans[1], DF McWilliams[1], RA Robins[2] and SA Watson[1], [1]Cancer Studies Unit, [2]Dept of Immunology, Queen's Medical Centre, Nottingham NG7 2UH

**Background & Aims** Gastrin has growth promoting effects on both normal and malignant tissues of the gastrointestinal (G1) tract. However, the extent of its role in cancer development remains controversial. The expression of the gastrin/CCK-B receptor (CCKBR) has been investigated in various G1 cell lines and in relation to the cell cycle stage in synchronised cells.

**Methods** Total RNA was extracted from human stomach, gastrointestinal (G1) cell lines and NIH3T3 fibroblasts transfected with the gastrin receptor (NIH3T3-CCKBR, obtained from Prof Matsui, Kobe, Japan) using RNAzol-B (Biogenesis, UK). Reverse transcription was performed with using random primers. Fluorescent real time RT-PCR was used to quantitatively determine relative gene expression levels of CCKBR in all of the samples. The PCR products were detected using the minor groove-binding dye, Sybr Green, and the fluorescence of the amplification reactions were monitored in real time using the ABI 5700 Sequence Detection System (Applied Biosystems, UK). The cycle number when the accumulated fluorescence crossed an arbitrary threshold was recorded (termed the ct value) for CCKBR and GAPDH PCR's. The relative gene expression levels, the ΔΔct values, of sample cDNA's were calculated according to manufacturer's instructions. The G1 cell lines were assessed along with cell populations that had been thymidine synchronised and then released. Parallel samples were fixed and stained with propidium iodide for determination of the cell cycle stage by flow cytometry.

**Results** Real time PCR showed CCKBR expression in each of the cDNA's from NIH3T3-CCKBR, GI cell lines and the human stomach. The human stomach showed statistically higher gastrin receptor mRNA levels than the CCKBR transfectants ($P<0.05$, Mann-Whitney). Both stomach and NIH3T3-CCKBR transfectants expressed significantly higher levels of gastrin receptor mRNA than G1 carcinoma cell lines ($P<0.01$, Mann-Whitney). The NIH3T3-CCKBR cell line was the focus of synchronisation studies. Flow cytometry results indicated that following release from synchronisation, the cells passed from G1, through S-phase and into G2 in approximately 10 hours. Real-time PCR results indicated that the expression of CCKBR mRNA increased steadily after release from synchronisation, peaking at 6 hours, at a level higher than unsynchronised control cells. This time point corresponds to when most cells are in S-phase.

**Conclusion** These results indicate that there is a correlation between the cell cycle stage and the level of receptor expression. The influence of the gastrin ligand on this relationship has been investigated. The stages of the cell cycle at which high and low expression occur may have implications for coupling to receptor signalling pathways and the stimulatory effects of gastrin.

### P191 DIFFERENTIATION OF HUMAN MELANOMA CELLS THROUGH p38 MAP KINASE IS ASSOCIATED WITH DECREASED RETINOBLASTOMA PROTEIN PHOSPHORYLATION AND CELL CYCLE ARREST

KSM Smalley and T Eisen. Institute of Cancer Research, Chester Beatty Labs, Fulham Road, London, UK

The 13-amino acid peptide α-melanocyte stimulating hormone (α-MSH) induces differentiation of B16 murine melanoma cells through activation of p38 MAP kinase (Smalley & Eisen, *FEBS Lett* **476**, 198). Recent studies have also highlighted the importance of the the retinoblastoma protein (pRB) in the process of terminal differentiation (Dyson, *Genes Dev*. **12**, 2245). The aim of the present study was to investigate whether α-MSH-induced differentiation of human COLO 853 melanotic melanoma cells proceeds via a p38 MAP kinase pathway and whether this was linked to the downstream modulation of pRB.

Treatment of COLO 853 cells with α-MSH induced time (0–8 hours) and concentration (0.01–100 nM)-dependent increases in the phosphorylation of p38 MAP kinase. This corresponded with the ability of α-MSH (10 nM, 72 hours) to induce differentiation, as characterised by increased accumulation of extracellular melanin (319 ± 20% basal melanin accumulation per cell) and reduced cell growth (51.0 ± 4.6% of control cell growth). Pre-treatment of cells with the p38 MAP kinase inhibitor, SB 203580 (10 μM), reversed the effects of α-MSH on both melanin accumulation and cell growth; implicating p38 MAP kinase in the α-MSH induced differentiation of COLO 853 cells.

Differentiation is often associated with cell cycle arrest. Flow cytometric analysis revealed that treatment with α-MSH (72 hours, 10 nM) increased the proportion of cells in the G1 phase of the cell cycle, from 52.9 ± 1.3% (in control cells) to 67.4 ± 2.1%, with a corresponding decrease in the proportion of cells in the G2/M phase. Incubation of cells with SB 203580 (10 μM) prior to α-MSH treatment, reduced the proportion of cells in the G1 phase to 59.9 ± 1.3%. Treatment of COLO 853 cells with α-MSH (10 nM, 72 hours) led to a dramatic decrease in the levels of phosphorylated pRB protein, with no change in levels of total pRB. The role of p38 MAP kinase in the α-MSH-mediated decrease in pRB phosphorylation was demonstrated by the finding that SB 203580 pre-treatment reversed this effect.

The fact that pRB protein is able to directly interact with the melanocyte transcription factor, micropthalmia gene product (Yavuzer et al. *Oncogene* **10**, 123), suggests that α-MSH may regulate melanocyte specific gene transcription via activation of p38 MAP kinase and modulation of pRB activity.

### P192 MODULATION OF THE CALPAIN CALPASTATIN PROTEOLYTIC SYSTEM CONTRIBUTES TO SRC-REGULATED TRANSFORMATION

NO Carragher, VJ Fincham and MC Frame, Beatson Institute for Cancer Research, Cancer Research Campaign Beatson Laboratories, Glasgow, G61 1BD

Integrin associated focal adhesion complexes provide the main adhesive links between the cellular actin cytoskeleton and the surrounding extracellular matrix (ECM) environment. In vitro studies provide evidence that motile cells utilize a complex spatial and temporally regulated mechanism of focal adhesion assembly at the leading edge of the cell co-ordinated with focal adhesion disassembly at the trailing edge to permit cell migration. Recent studies indicate that the calpain family of proteolytic enzymes can promote limited proteolytic cleavage of several components of the focal adhesion complex leading to disassembly of these complexes followed by decreased substrate adhesion and increased cell migration. Such mechanisms that regulate migration of normal cells may be deregulated under pathological conditions characterized by increased cell motility such as tumour invasion. v-Src induced oncogenic transformation is associated with loss of focal adhesion structures and transition to a less adherent more motile phenotype. To further elucidate the signalling pathways downstream of v-Src that promote the transformed cell phenotype, we have investigated the role the calpain-calpastatin proteolytic system plays during oncogenic transformation induced by v-Src. Our studies have characterized a positive feedback loop mechanism describing an increase in protein synthesis of calpain following v-Src activation that leads to proteolytic degradation of the endogenous calpain inhibitor, calpastatin, thereby enhancing calpain activity in transformed cells. We further demonstrate that v-Src induced disassembly of focal adhesions and cell transformation is accompanied by calpain mediated cleavage of the focal adhesion kinase (FAK). Overexpression of calpastatin or treatment with calpain inhibitors repress, FAK cleavage, focal adhesion disassembly, cell migration and also cell cycle progression in v-Src transforming cells. This data suggests that v-Src induced modulation of the calpain-calpastatin proteolytic system contributes significantly to the process of oncogenic transformation. Thus, manipulation of the calpain-calpastatin proteolytic system may represent a novel therapeutic approach for inhibition of tumour growth and invasion.

### P193 CHANGES IN GENE EXPRESSION ASSOCIATED WITH BREAST CANCER INVASION

G Zhu[1], T Crnogorac-Jurcevic[2] and IR Hart[1], [1]Richard Dimbleby Department of Cancer Research/ ICRF Labs, St Thomas' Hospital, London SE1 7EH, [2]ICRF Molecular Oncology Unit, Hammersmith Hospital, London W12 0HS

Gene expression changes may play an important role in cancer invasion. To evaluate this possibility, the PALM micro-laser dissection and capture system was employed to isolate pure populations of tumour cells from ductal carcinoma in situ (DCIS) which then were used for molecular genetic analysis. We dissected the central and peripheral areas of DCIS lesions separately and pooled tissue from 10 sections which were obtained from 5 different patients. By using the TRZoL kit, total RNA was extracted from microdissected tissue. The Atlas SMART™ Probe Amplification Kit was used to synthesise and amplify cDNA. Probes from the margin and from the centre were hybridised to duplicates of Atlas Human Cancer 1.2 Arrays which contain 1,176 known genes. The absolute intensity of each individual cDNA spot was determined by phosphorimager scanning. After comparing images by AtlasImage™ 2.0, we have found that 32 genes changed their expression level in the periphery of tumour lesions relative to the central regions of these tumours. 26 genes are up-regulated, and 6 genes are down-regulated (arbitrary level of 2-fold or greater). Verification that this procedure was identifying genes of importance in tumour invasion was provided by the observed down-regulation of the metalloprotease inhibitor 1, TIMP 1, at the periphery, upregulation of the mesenchymal marker vimentin at the periphery and up-regulation of the hyaluronan receptor, RHAMM, a protein already shown by us to be increased at the invading edge of breast cancers (Assmann et al, In Press). Such changes in DCIS appear to indicate that the rim of these precursor regions contain cells better able to become more invasive.

This method enables us to compare gene expression in various regions of a pure tumour cell population and to identify changes in gene expression associated with varying anatomical locations within the developing tumour mass.

### P194 MECHANISMS OF ADHESION-DEPENDENCE IN MOUSE MELANOCYTE-DERIVED CELL LINES

Ruth Gilchrist, Helen Stafford, Paula M Duddy, John F Marshall, Richard S Camplejohn, Ian R Hart, Richard Dimbleby Department of Cancer Research/ICRF Laboratory, Rayne Institute, St Thomas' Hospital, Lambeth Palace Road, London, SE1 7EH

We previously characterised a panel of C57/B1 mouse melanocyte-derived cell lines with regard to cell cycle alterations involved in adhesion-dependent and adhesion-independent growth (Br.J.Cancer, **80**(2), 25, 1999). Using BrdUrd labelling techniques we reported that, in suspension culture, Melan-a (immortal melanocyte) cells arrest immediately in $G_1$. Melan-a cells are non-tumourigenic and require supplements of TPA and Cholera Toxin to proliferate. When maintained in culture for more than thirty passages, Melan-a cells spontaneously acquire the ability to proliferate in suspension culture; these cells were termed Mel* (at 24 hrs of anchorage-independent growth, S-phase Melan-a <1%, S-phase Mel* >15%). When injected into C57/B1 mice, the Mel* cell line remained non-tumourigenic (n = 10 animals, $1 \times 10^6$ cells, no tumour growth by six months). Growth curve data also show that Mel* retained growth-factor dependency. Thus Mel* cells resembled Melan-a cells phenotypically except in terms of adhesion dependence. Protein lysates were obtained from both cell lines 24 hrs after plating on serum-coated or polyheme-coated plastic. Analysis of $G_1$ cell cycle control proteins by Western blotting showed that Mel* cells maintain Cyclin D1 expression when grown in suspension, unlike Melan-a cells which rapidly down regulated Cyclin D1 levels. The ability to maintain Cyclin D1 levels in turn maintained levels of hyperphosphorylated Rb as determined by Western blotting. To determine whether overexpression of Cyclin D1 in Melan-a cells was sufficient to induce an adhesion-independent phenotype, cells were infected with FLAG-tagged Cyclin D1 (gifted from Gordon Peters, ICRF, London). To date, such infected cells remain adhesion-dependent. Elucidation of the additional biochemical events involved in conversion from monolayer to suspension growth will shed light on the step-wise tumourigenic conversion of cells of the melanocyte lineage.

### P195 MMP-7 EXPRESSION: RELATIONSHIP TO APC/b-CATENIN STATUS

HM Collins[2], JH Scholefield[2], SA Watson[1], [1]Academic Unit of Cancer Studies, [2]Division of GI Surgery, University Hospital, Nottingham, NG7 2UH

**Introduction** Matrix metalloproteinases are a family of proteolytic enzymes which are capable of degrading the extracellular matrix. MMP-7 is predominantly expressed by epithelial cells and is overexpressed at an early stage in colorectal cancer. The roles of APC/β-catenin mutations have been investigated as possible mediators for transcriptional induction of MMP-7.

**Methods** We examined MMP-7 mRNA expression (i) following induction of wild type APC in the colorectal cancer cell line HT29 which has a zinc inducible APC gene, and (ii) in two adenoma cell lines grown in vitro (with different APC mutations). The HT29 cell line was grown under standard tissue culture conditions with various concentrations of zinc. Total RNA was extracted using RNAzol B and reverse transcribed to cDNA. MMP-7 gene expression was quantified using real time PCR and the levels normalised to both GAPDH and baseline MMP-7 expression. All assays were controlled by the inclusion of wild type HT29 and a vector control cell line. β-catenin was examined in all cell lines using western blotting.

**Results** MMP-7 mRNA is expressed in all cell lines examined, this was irrespective of the induction of wild type APC. In HT29 induced to express wild type APC there was a decrease in MMP-7 mRNA expression compared to the control cell lines, this corresponded to a decrease in β-catenin protein levels. MMP-7 is expressed by the adenoma cell lines examined. RGC2/42 which has full length *APC* expresses MMP-7, albeit at a lower level than AA/C1. RGC2/42 has a truncated β-catenin protein as determined using western blotting.

**Conclusions** β-catenin status is important in the regulation of MMP-7 expression. Expression may be related to the breakdown of the interaction between APC and β-catenin. MMP-7 mRNA expression is generally increased by the addition of zinc, however this is reversed following restoration of wild type APC. Wild type APC sequesters β-catenin in the cytoplasm for subsequent proteasomal degradation, therefore it is not available for transcription. Studies are ongoing to establish the role of β-catenin in controlling MMP-7 transcription.

### P196 OVEREXPRESSION OF Rho-A IN COLORECTAL CARCINOMA CELL LINES AND ITS EFFECTS ON ADHESION, INVASION AND MOTILITY

O Dewhurst, D Jayne & PJ Guillou, Academic Unit of Surgery, Clinical Sciences Building, St James's University Hospital, Leeds, LS9 7TF

Members of the small Rho-GTPase family of proteins are vital for a broad range of cellular functions including adhesion, motility and invasion, which are necessary for the metastatic cascade. Our group is particularly interested in the family member, Rho A which is required for cytoskeletal dynamics.

Several human colorectal cell lines were transfected with a pcDNA3 based, CMV driven vector. The expression vectors contained wild-type RhoA and a constitutively active mutant (kind gifts from Kazuyuki Itoh, Japan). DNA was prepared and transfected into SW948 and SW480 (adherent cell lines) and COLO 320 (non-adherent cell line). As a control, sham transfectants were made where cells were transfected with vector alone. Successful transfection confers neomycin (G418) resistance to cells and was therefore used for selection of transfected cells. The expression vectors contained an N-terminus octapeptide epitope (DYKDDDDK) FLAG-tag. Transfected cells were then identified by immunofluorescence and SDS-PAGE/Western Blotting using a FLAG M5 antibody.

Overexpression of both wild-type and constitutively active forms of RhoA have resulted in increased levels of invasion through an *in vitro* Transwell based invasion assay. Invasion in transfected and control cells was totally inhibited following pre-incubation of cells for 48 hours with the RhoA specific inhibitor, *botulinum* toxin C3 exoenzyme. Little difference has so far been observed in levels of motility over 48 hours between transfected and control cells, using the colloidal gold migration assay. Glass coverslips are coated with bovine serum albumin (BSA) and colloidal gold. Cells are then added to the coated coverslips and as they ingest the BSA, particles of the collodial gold are also ingested. As the cells paths are cleared on the coverslip which are visible under dark-field illumination. We are currently in the process of developing a method using the LUCIA image analysis software to quantitate the area cleared by the cells. Using a 96well micro-titre plate adhesion assay, we have shown that SW480 cells expressing constitutively active RhoA achieve maximum attachment after 20minutes compared with 60minutes in the control cells. We have also observed altered growth patterns in adherent cell lines, whilst the non-adherent cell line, COLO320 have become adherent. We believe that through these studies and our continuing work in this area that we may further our understanding of the role of RhoA in the peritoneal metastasis of gastrointestinal carcinoma.

### P197 EXPRESSION OF HEPATOCYTE GROWTH FACTOR/SCATTER FACTOR, C-MET, HGF ACTIVATOR AND INHIBITORS IN HUMAN CANCER CELLS

C Parr, and WG Jiang, Dept. of Surgery, University of Wales College of Medicine, Cardiff CF14 4XN, UK

Hepatocyte growth factor/scatter factor (HGF/SF) elicits a number of functions that are tumourigenic and enhance the metastatic potential of cancer cells. Pro-HGF/SF becomes bio-active upon activation by the HGF/SF activator (HGFA). This study analysed the expression of HGF/SF, its receptor, cMET, HGFA and HGFA inhibitors (HAI) in various human cancer cell lines including breast, prostate, colon, pancreas, liver, and bladder, while also comparing against normal cells such as fibroblasts and endothelial cells.

Reverse-transcriptase/polymerase-chain reaction (RT-PCR) was employed to determine the degree of expression of the HGF, c-met, HGFA, HAI-I and HAI-2 genes in the cell lines examined. Results have shown that the only cell line producing a significant amount of HGF/SF was the human fibroblasts (MRC5), and that these fibroblasts also expressed c-met plus HGFA, thus allowing autocrine regulation of HGF/SF activity, and importantly there was virtually no inhibitor presence to inhibit the biological function of HGF/SF. Breast cancer cells (MDA231) expressed large amounts of both c-met and HGFA to allow maximum influence of HGF/SF and also did not express the HAI-1 gene, but in contrast did express HAI-2 to a large degree. The pattern of the inhibitors generally shows that they are not present in similar proportions in the same cell line and that although HAI-2 is present to some degree in all cell lines tested, HAI-1 was either absent or expressed at a lower level than HAI-2. Interestingly, the cell lines that displayed little or no HAI-1 expressed HGFA or HGF/SF. Liver cancer cells (PLC) only expressed the HGFA gene. The metastatic potential of a cell type (breast cancer or prostate cancer cells in study) correlates to the degree of c-met and/or HGFA expression.

In conclusion, HGF/SF, its activator and inhibitors are expressed in different patterns in cancer cells and fibroblasts. High levels of HGFA expressed in cancer cells and fibroblasts may contribute to the autocrine and paracrine activation of HGF/SF.

### P198 SUPPRESSION OF EXPRESSION OF HGF/SF IN FIBROBLASTS AND ITS RECEPTOR, c-MET, IN BREAST CANCER CELLS USING HAMMERHEAD RIBOZYMES

WG Jiang[1], D Grimshaw[1], J Lane[1], TA Martin[1], R Abounder[2], J Laterra[2], RE Mansel[1], [1]Dept of Surgery, Univ Wales College of Medicine, Cardiff, UK, [2]Dept Neurology, Kennedy Krieger Institute, Johns Hopkins Univ. School of Medicine, Baltimore, USA

HGF/SF, via its receptor cMET, has been implicated to play a pivotal role in breast cancer development and progression. This study examined a transgene consisting of a combination of U1snRNA, hammerhead ribozyme and antisense, designed to inhibit expression of HGF/SF and its receptor, c-met, and its impact on the migration and in vitro invasion of breast cancer cells.

Human breast cancer cells, MDA MB 231 and MCF-7 were transfected with the c-MET ribozyme-containing plasmids by electroporation. Human fibroblast cells, MRC5, were infected with a retroviral ribozyme containing HGF/SF antisense. Stable transfectants of breast cancer cells almost completely lost c-MET at both mRNA and protein levels, as shown by RT-PCR, Northern blotting and Western blotting, respectively. MRC5 infected with ribozyme exhibited a similar reduction of HGF/SF, as determined by RT-PCR and an HGF/SF bioassay. Met-ribozyme transfected cells exhibited a reduction of migration (determined by motion analysis) and in vitro invasiveness through extracellular matrix (Matrigel) in response to rhHGF/SF, compared with the wild type cells and cells transfected with empty plasmid. MRC5 carrying HGF/SF ribozyme also lost their ability to induce in vitro invasion of breast cancer cells.

It is concluded that targeting HGF/SF and its receptor, cMET, by way of hammerhead ribozymes encoding antisense to the molecules is an effective approach in reducing the invasiveness of breast cancer cells.

### P199 NK4, AN HGF/SF ANTAGONIST INHIBITS HGF/SF INDUCED CHANGES IN TIGHT JUNCTION PROTEINS

TA Martin, WG Jiang and RE Mansel, University Department of Surgery, University of Wales College of Medicine, Heath Park, Cardiff, CF14 4XN

Permeability of endothelial cells is governed by tight junctions, the most topical structures in the endothelium. Interaction and penetration of the endothelium by cancer cells is an important step in the formation of metastases, indicating that changes in tight junction formation will be an early and key aspect. NK4 is a variant of HGF/SF (Hepatocyte Growth Factor/Scatter Factor). We have already shown that NK4 can prevent HGF/SF induced changes in tight junction function[1,2] and that NK4 is antagonistic to HGF/SF[3].

**Purpose** This study sought to determine the effect of HGF/SF and its antagonist, NK4 on the expression of molecules involved in tight junction structure and function.

**Methods** Human umbilical vein endothelial cells (HUVEC) were co-cultured with HGF/SF (10 ng/ml) and/or NK4 (100 ng/ml) for up to 24 h in order to investigate any changes in expression of the tight junction proteins ZO-1 and occludin. Western blotting was used to ascertain changes in protein levels. mRNA was extracted from co-cultured confluent cells followed by RT-PCR to observe any changes in the tight junction proteins claudin-1, claudin-5, junctional adhesion molecule (JAM), in parallel to investigating changes in tight junction function, assayed by measuring transendothelial resistance (TER) and paracellular permeability.

**Results** Western blotting revealed that HGF/SF decreased the protein level of ZO-1 (decreased over the 24 h of culture with HGF/SF from an initial relative density of 230 to one of 175 at 24 h co-culture), but did not cause a change in level of occludin. RT-PCR revealed that addition of HGF/SF caused no change in signal for claudin-5 or JAM, but decreased the signal for claudin-1. NK4 was able to prevent the decrease in levels of ZO-1 and claudin-1 by HGF/SF. In a parallel study NK4 was able to prevent HGF/SF induced decrease and increase in TER and paracellular permeability respectively[1].

**Conclusions** We conclude that HGF/SF induces changes in tight junction function via modulation of proteins involved in tight junction function and structure. NK4 inhibits these effects of HGF/SF and may therefore have a role to play in the control of invasion of endothelium by cancer cells.

1. Martin et al (2000) *Breast Cancer Res Treat* **64**(1): 108.
2. Martin et al (2000) *Breast Cancer Res Treat* **64**(1): 133.
3. Jiang et al (1999) *Clin Cancer Res* **5**: 3695.

### P200 MODULATION OF PTEN EXPRESSION BY TGF-β1

R Crosland[1], M Wells[2], ME Blair[1], [1]BMRC, Sheffield Hallam University, Sheffield S11 WB, [2]Dept. of Pathology, Sheffield University Medical School, Sheffield S10 2JF

Genetic and biochemical studies have identified a tumour suppressor gene at 10q23.3 which has been termed PTEN for *P*hosphatase and *Ten*sin Homologue Deleted on Chromosome *Ten*. Somatic mutations in the PTEN gene have been found in many types of cancer, but most frequently in cancer of the prostate, endometrium, glioblastomas and melanomas. PTEN is currently considered to be the most frequently after p53 tumour suppressor mutated in cancer. Germ-line PTEN mutations have been also detected in three rare autosomal dominant disorders with overlapping clinical features: Cowden disease, Bannayan–Zonana syndrome and Lhermitte–Duclos disease. PTEN is a 403aa protein with homology to protein phosphatases and tensin and auxilin. It has been established, however, that in spite of homology to dual specificity protein phosphatases, PTEN is an inefficient protein phosphatase in vitro and that its main substrate is phosphatidylinositol (3,4,5) trisphosphate (PIP-3), which acts in cells as a second messenger. In this study modulation of PTEN expression by TGF-β in HEC 1B cells derived from endometrial carcinoma was investigated. For this purpose cells were grown in McCoy's modified 5A medium with reduced (1%) FCS and treated with 2ng or 5 ng/ml TGF-β1. Untreated cells were used as a control. Levels of PTEN mRNA and protein were determined by RT-PCR and Western blotting, respectively. Up-regulation of PTEN by TGF-β1 was detected. The possible significance of this for inhibition of cell proliferation in the endometrium will be discussed.

### P201 MOLECULAR MODELLING OF DNA-BINDING INHIBITORS OF HUMAN TELOMERASE

D Cairns, TC Jenkins[1] and SP Mackay[2] Institute of Pharmacy and Chemistry, University of Sunderland, Sunderland, [1]YCR Laboratory of Drug Design, University of Bradford, Bradford, [2]Dept. of Pharmaceutical Science, University of Strathclyde, Glasgow

Telomerase is an RNA-dependent DNA polymerase enzyme responsible for the elongation of repeating telomeric sequences found at the 3′-end of eukaryotic chromosomes. Telomerase is not active in healthy somatic cells but is overexpressed in almost 90% of human cancers, As a result telomerase has attracted considerable attention as a possible target for anti-cancer chemotherapy. Recently, a number of mono- and bis-substituted anthraquinones were synthesized, fully characterized and tested for their ability to inhibit telomerase and *Taq* polymerase using a modified TRAP (Telomere Repeat Amplification Protocol) assay and their toxicity determined in a panel of ovarian carcinoma cell lines (A2780, CH1 and SKOV-3) using a sulforhodamine B assay[1]. All compounds tested displayed inhibition of telomerase of up to 43% at concentrations of 10 μM. Importantly, none of our compounds inhibited the related enzyme, *Taq* polymerase, at concentrations of up to 50 μM, nor did any display significant general cell toxicity. In an attempt to explore the structural requirements for telomerase inhibition, molecular modelling studies were undertaken. Briefly, an intercalation site was introduced into the intramolecularly folded tetraplex solution NMR structure of the human telomeric DNA sequence $d[AG_3(T_2AG_3)_3]$[2] and the structure minimized using a distance dependent dielectric of 4.0. Anthraquinones were manually docked and the resulting complexes minimized to yield plausible starting conformations. Molecular dynamics simulations were then undertaken to allow the anthraquinones to explore the intercalation site. Finally, the average trajectory from the dynamics was minimized and the net binding enthalpy calculated. All simulations were carried out in an aqueous solvent environment in order to replicate as much as possible the situation *in vivo*. The modelling results show a similar order of potency to the experimental data thereby validating our model and suggesting that intercalation into the G-tetraplex associated with human telomeric sequences is an important feature of the molecular mode(s) of action of telomerase inhibitors of this type.

1. Gibson, V. Anderson, R.J., Jenkins, T.C. and Cairns, D. (1999) *Pharm. Pharmacol. Comm*. **5**, 669
2. Wang, Y. and Patel, D.J. (1993) *Structure*, 1, 263

### P202 TOWARD NON-TOXIC THERAPIES: DESIGN, SYNTHESIS AND DNA SELECTIVITY OF SECOND-GENERATION TELOMERASE INHIBITORS

PJ Perry and TC Jenkins, YCR Laboratory of Drug Design, University of Bradford, BRADFORD BD7 IDP, UK

Anthraquinone-based molecules are known to interact with a varied number of nucleic acid targets. For example, the anticancer drug mitoxantrone exerts at least a proportion of its biological effects through interaction with duplex DNA. More recently, examples of this class of molecule (e.g. **1**) have found application through interaction with higher-order DNA structural forms. Stabilisation of DNA triplexes by such molecules may facilitate highly-specific inhibition of gene expression[1] whilst interaction with guanine-tetraplex structures formed by the intramolecular folding of G-rich DNA has been shown to inhibit telomerase,[2] a tumour-specific enzyme essential for sustained cellular proliferation in tumour cells.

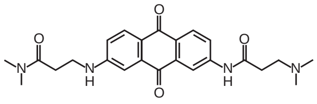

(**1**)

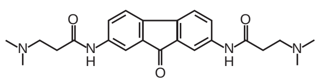

(**2**)

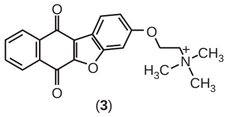

(**3**)

However, for these therapies to be effective it is essential that acute toxicity is minimised or preferably "designed-out" completely.[3] To address these issues, we have rationally designed two second-generation drug series (**2** and **3**) wherein duplex-binding, and thus cytotoxicity, is minimised yet binding to higher-order DNA triplexes and tetraplexes is retained with biological efficacy.[4, 5] Structural selectivity for nucleic acid binding is assessed using competitive dialysis and a correlation between duplex binding and cytotoxicity is established for a panel of cell lines.

1. MD Keppler et al **1999** *Eur. J. Biochem* 263: 817–825.
2. PJ Perry et al., **1998** *J. Med. Chem* 41: 4873–4884.
3. PJ Perry, TC Jenkins **1999** *Expert Opin. Invest. Drugs*, 8: 1981–2008.
4. PJ Perry et al., **1999** *J Med. Chem*., 42: 2679–2684.
5. PJ Perry et al., **1999** *Anti-Cancer Drug Des*., 14: 373–382.

### P203 HTERT EXPRESSION IS SIGNIFICANTLY HIGHER IN BREAST CANCER TISSUE THAN IN ADJACENT NON-CANCEROUS TISSUE

KL Kirkpatrick[1], R Carpenter[1], W Ongunkolade[1], C Laban[1], A Elkak[1], M Ghilchick[2], S Bustin[1], PJenkins[1], K Mokbel[3], [1]Barts and The London NHS Trust, [2]Central Middlesex Hospital, [3]St George's Hospital

**Introduction** Telomerase is a ribonucleoprotein responsible for synthesising the repetitive nucleotide sequence which makes up telomeres at the end of chromosomes. Without telomerase activity each round of cellular division results in shortening of the telomeres. This can lead to chromosomal instability and cell senescence, whereas continued telomerase activity allows the possibility of cellular immortality. Telomerase is active in 85–90% of human cancers but is undetectable in most normal somatic cells. Three components have been identified, of which hTERT (human telomerase reverse transcriptase) is the one which appears to confer enzyme activity.

**Aims** Our aim in this study was to investigate the expression of hTERT in human breast cancer and adjacent non-cancerous tissue (ANCT) using quantitative RT-PCR.

**Materials and Methods** Samples of breast tumour and macroscopically normal breast tissue 1 cm from the tumour were taken from 32 patients undergoing breast cancer surgery. Total cellular RNA was extracted using the RNeasy (Qiagen) kit. Following quantification of RNA (Ribogreen reagent – Molecular Probes Europe BV) RT-PCR was performed in duplicate for each sample using the ABI PRISM 7700 Sequence Detector (Perkin-Elmer Applied Biosystems).

**Results** For adjacent non-cancerous tissue the median copy number for hTERT mRNA was 12.1 copies per ηg of RNA (range 0–9850). Of 32 specimens 30 (94%) had less than 1000 copies of hTERT RNA per ηg of total RNA.

For breast cancer tissue the median copy number for hTERT mRNA was 152,000 (range 6.55 to 7,910,000). Of 32 specimens 26 (81%) expressed more than 1000 copies of hTERT per ηg of total RNA. Statistical analysis using the $\chi^2$ test gave a value of 51 ($P<0.001$).

**Conclusions** These results show that there is a significant difference in the expression of hTERT mRNA between cancerous and non-cancerous breast tissue. There is potentially a use for hTERT as a diagnostic marker for breast malignancy, particularly using a cut-off point for significant levels of expression.

### P204 T ELOMERASE ACTIVITY IN IRANIAN PATIENTS WITH ESOPHAGEAL SQUAMOUS CELL CARCINOMA

S Kazemi Noureini*, AA Ziaee, M Yazdanbod⁺ *Institute of Biochemistry and Biophysics, University of Tehran, Iran; ⁺Dept. of Surgery, Shariati Hospital, Medical Science University of Tehran

Telomerase is one of the tools which immortalize stem or cancerous cells. This special reverse transcriptase elongates telomeres and prevents telomere erosion naturally ocurr in every cell cycle. In about 80–90% of the different types of cancer cells telomerase is activated, while in most somatic cells this enzyme is switched off. Therefore telomerase can be a good tumor marker and also convenient for cancer therapy. Esophageal cancer is the fifth most ferequent cause of cancer death worldwide and esophageal sqoamous cell carcinoma (ESCC) is highly associated with cultural habits, environmental chemicals and nutrients. In the persent work telomerase activity in Iranian patients suffered from ESCC was observed applying Telomerase Repeat Amplification Protocol (TRAP) according to Kim, N. et al 1994 with some modifications. In more than 90% of the 22 samples of different stages of ESCC telomerase activity was detected, therefore this suggests that telomerase activation happens in an initial step of carcinogenesis pathway of ESCC. However further studies should be done for clarifying the exact time of this enzyme activation. Anyway activation of telomerase precede mutation/loss of heterozigosity of p53. Although telomerase activity could be considered as a good tumor marker in ESCC, there is no correlation between the amount of telomerase activity and the progress of this kind of cancer. On the other hand, a very slight activity was observed in normal epithelial tissues adjacent tumor that can be due to the presence of epithelial stem cells or some small contamination with cancerous cells. It is also possible that ESCC could be resulted from incomplete differentiation or a failure in telomerase gene switching off which normally ocurres in normal differentiation.

### P205 TRAP ASSAY ANALYSIS OF TELOMERASE ACTIVITY IN BARRETT'S OESOPHAGUS

AJ Fletcher-Monaghan[1], JJ Going[2], R Stuart[3] and WN Keith[1], [1]CRC Dept Medical Oncology, University of Glasgow, CRC Beatson Labs., Glasgow G61 1BD, [2]University Dept Surgery & [3]University Dept Pathology, Glasgow Royal Infirmary

**Background** Barrett's oesophagus is a pre-neoplastic lesion of the oesophagus. The squamous epithelium of the lower oesophagus is replaced by a metaplastic columnar epithelium due to severe tissue injury caused by acid reflux. Most cases of oesophageal adenocarcinoma arise in a pre-existing area of Barrett's oesophagus and the cancer risk is approximately 30 fold that of the general population. However, less than 10% ever develop cancer. Telomerase is a ribonucleoprotein, which replicates the terminal sequences of eukaryotic chromosomes and is active in germ line tissues. It is absent from most normal somatic cells but is expressed in 85% of cancers.

**Aim** To investigate the differential expression of telomerase in normal mucosa of oesophagus and stomach compared to Barrett's oesophagus with a view to identifying a biomarker for disease progression.

**Methods** Biopsies were taken (between 4 and 11) from each patient along the length of the GI tract. We determined the telomerase activity of 344 biopsies from 49 patients (31 with Barrett's oesophagus, 13 with gastric cancer, 3 with oesophageal cancer and 2 with cancer of the oesophago-gastric junction) using the telomeric repeat amplification protocol (TRAP) assay.

**Results** 56% of patients were found to have at least one Barrett's biopsy positive for telomerase. Less than 1% of normal stomach (antrum and body) biopsies were found to be positive. 87% of patients were found to have normal squamous oesophagus biopsies positive for telomerase.

**Conclusions** There is a significant difference in the percentage of patients with a Barrett's biopsy positive for telomerase compared with normal stomach. This may be an indication of which patients will progress to invasive disease and will be monitored in an ongoing study. It should be noted however, that multiple samples were taken from each patient to enable us to identify those patients who had a Barrett's biopsy positive for telomerase. Telomerase is believed to be constitutively expressed in permanently renewing epithelia. Consequently, normal squamous oesophagus should not be used as a negative control.

### P206 IN VITRO MODELLING OF THE PROSTATE: 3D CULTURE OF PROSTATIC CELLS IN MATRIGEL

SH Lang, M Stark[1], J Hall, K Hyde, M Stower[2] and NJ Maitland YCR Cancer Research Unit, Dept. of Biology[1], University of York and York District Hospital[2], York, YO10 5YW, UK

The aim of our work is to develop a three dimensional in vitro model of the prostate which reflects both architectural and functional aspects of the prostate and incorporates both stromal and epithelial cells.

Epithelial and stromal cultures were derived from prostatic tissue of patients undergoing surgery for non-malignant disease. Epithelia were seeded into Matrigel (a commercially available basement membrane) in the presence or absence of stroma and prostatic hormones. Epithelial spheroids were grown for up to three weeks and then analysed by electron microscopy and immunohistochemistry.

Spheroids contained 1–2 epithelial cell layers which were positive by immunohistochemistry for epithelial (cytokeratin, E-cadherin) and prostatic markers (PSA, PSMA). In the presence of stroma, serum and hormones the epithelial cells became more columnar and polarised (demonstrated by confocal and transmission electron microscopy). Microvilli, secretory vesicles, Golgi bodies and PSA expression were consistently luminal whilst beta 1 integrin expression was basal. The presence of stromal cells also increased spheroid forming efficiency.

Primary epithelium was compared to epithelial cell lines. We cultured both PC-3 (metastatic) and PNT2-C2 (normal) prostatic cell lines in Matrigel. PC-3 cells formed hollow spheroids, with predominantly one cell layer and evidence of polarisation, whilst PNT2-C2 cells formed spheroids of solid cells.

We have produced a three dimensional model capable of reflecting prostatic acini architecture and function. Such a model will allow us to study (and manipulate) cellular interactions and how they change with progression of prostate and provide a relevant model to test new drugs and treatments prior to human clinical trials.

### P207 IN VITRO MODELLING OF THE PROSTATE: 3D CULTURE OF PRIMARY PROSTATE CELLS IN COLLAGEN GELS

J Hall, M Stower[2] and NJ Maitland. YCR Cancer Research Unit, Dept. of Biology[1], University of York and York District Hospital[2], York, YO10 5YW, UK

**Introduction** Despite the prevalence of prostate cancer (CaP) there are few good in vitro models with which to predict why some cancers metastasise while others remain organ confined. Alterations in stromal epithelial interactions play an important role in advancement to a malignant phenotype. Our model aims to shed light on conflicting evidence relating to the effects that stromal cells have on prostate epithelial cell growth, motility and invasion: i.e. do they stimulate or inhibit and does this change with disease progression?

**Methods** Fibroblasts derived either from BPH tissue (BPHFb) or from CaP tissue (TFb) were seeded into a collagen type 1 gel and allowed to form a matrix. PEC derived from human BPH tissue or Cap tissue were cultured on the surface of the fibroblast-populated collagen gels for 15 days. Cultures were examined morphologically, after frozen sectioning, using light microscopy and immunohistochemical analysis with markers for prostate specificity, cellular differentiation and cell adhesion.

**Results** A difference was found in the invasion of PEC derived from CaP tissue and BPH tissue when grown on BPHFb or TFb gels. On TFb gels PEC remained at the surface of the gel whereas on BPHFb gels PEC invaded into the gel. In the case of PEC derived from BPH tissue they formed acinus like structures which stained strongly for basal CK and less strongly for luminal Ck. They also stained positively for the prostate specific markers PSMA and PSA. Strong cell to cell E-cadherin staining showed that the cells were adherant.

**Conclusion** Our in vitro 3D culture model, based on a primary prostate fibroblast populated type 1 collagen lattice, allows us to move closer to the in vivo situation to study stromal epithelial interactions in the prostate and indicates distinct differences between normal and malignant tissues.

### P208 MOTOGEN INDUCED DISSOCIATION OF THE E-CADHERIN/CATENIN COMPLEX AND ASSOCIATION OF β-CATENIN WITH SIGNAL TRANSDUCTION PATHWAYS IN PROSTATE CANCER CELLS,

G Davies*[1], W Jiang[1] and M Mason[2], [1]Depts. of Surgery and [2]Clinical Oncology, University of Wales College of Medicine, Cardiff, CF14 4XN

The effect of Hepatocyte Growth Factor/Scatter Factor (HGF/SF) was investigated on its tyrosine kinase receptor c-Met, on E-cadherin/β-catenin function and on the β-catenin/APC/GSK3β complex (Axin complex) using an E-cadherin positive (LNCapFGC), or E-cadherin negative (PC-3) prostate cancer cell line. Incubation with the motogen HGF/SF, showed no change in the co-precipitation status of APC with either GSK3β, or β-catenin in both prostate cell lines using immunoprecipitation. In contrast, co-precipitation between GSK3β and β-catenin was found to be increased upon continued exposure to HGF/SF in LNCapFGC cells. Furthermore, continued exposure to HGF/SF increased the level of co-precipitations between the E-cadherin/β-catenin complex with c-Met, and also increased tyrosine phosphorylation of c-Met. Using immunofluorescence, continued exposure to HGF/SF decreased the level of co-localised peripheral staining between the E-cadherin/β-catenin complex with the c-Met receptor, and increased the level of co-localised cytoplasmic staining between β-catenin and GSK3β. RT-PCR revealed that there were no mutations within the binding regions between β-catenin and GSK3β. In conclusion, uncomplexed cytoplasmic pools of β-catenin associate more readily with the Axin complex in the absence of E-cadherin. Whereas, in the presence of E-cadherin, β-catenin is stabilised by forming tight cell-cell contacts. Furthermore, the association between the E-cadherin/β-catenin complex with the HGF/SF receptor c-Met, may regulate intercellular adhesion in prostate cancer following stimulation by HGF/SF.

### P209 INHIBITION OF HGF/SF-INDUCED CELL-MATRIX ADHESION, INVASION, MIGRATION & PAXILLIN PHOSPHORYLATION, IN PROSTATE CANCER BY THE HGF/SF ANTAGONIST, NK4

C Parr[1], G Davies[1], S Hiscox[1], T Nakamura[2], K Matsumoto[2], and WG Jiang[1], RE Mansel[1], [1]Dept. of Surgery, University of Wales College of Medicine, Cardiff CF14 4XN, UK, and [2]Dept. of Biochemistry, University of Osaka, Osaka, Japan

Hepatocyte growth factor/scatter factor (HGF/SF) plays a key role in regulation of migration, cell-matrix adhesion, invasion and angiogenesis in cancer, through the phosphorylation of its specific receptor, the c-met tyrosine kinase. This study sought to examine the effects of the recently discovered HGF/SF variant, NK4, on the influence of HGF/SF in prostate cancer cells.

Human prostate cancer cells PC-3 and DU145 were tested. Tumour cell adhesion to the extracellular matrix (Matrigel) was significantly increased by HGF/SF (number of adherent cells 94.1±7.6 with HGF/SF vs 51.3±4.9 in control, $P<0.01$). However, this increase was reduced in the presence of NK4 (50.2±4.49, $P=0.01$). In an in vitro invasion assay, NK4 was also found to suppress the invasion induced by HGF/SF (invasion index being 73.1±2.0 with HGF/SF and 52.8±4.2 with HGF/SF plus NK4, $P<0.01$). In a real time cellular motion analysis, NK4 was also found to significantly reduce the migration of these cells (migration distance over 100 mins being 18.7±6.5 in a control, 34.6±10.7 with HGF/SF and 25.5±6.5 in a combination of HGF/SF and NK4). It was further shown that HGF/SF stimulation of the cells resulted in an increase in the degree of phosphorylation of paxillin (immunoprecipitation and Western blotting) and also an increase in the immunofluorescent staining of paxillin in the focal adhesion complexes. Once again, the influence of HGF/SF on paxillin phosphorylation was antagonised in the presence of NK4.

In conclusion, this study shows that HGF/SF stimulates cell-matrix interactions, tumour cell motility and matrix invasion. These properties, required for metastatic spread, can be inhibited by NK4.

### P210 SOLUBLE FIBROBLAST GROWTH FACTOR RECEPTOR AS A STRATEGY TO INHIBIT PROSTATE TUMORIGENESIS,

AF West, DE Neal and HY Leung. Prostate Research Group, Dept of Surgery, University of Newcastle upon Tyne, Framlington Place, Newcastle upon Tyne NE2 4HH

**Introduction** Carcinoma of the prostate remains a major clinical problem. The family of fibroblast growth factors (FGFs), and their high affinity membrane tyrosine kinase receptors (FGFRs), play key roles in development and progression of this cancer. A variety of approaches have been employed to bring about inhibition or down-regulation of growth factors and/or their receptors, with beneficial effects against human malignancies. Using the strategy of dominant-negative inhibition, Celli and colleagues expressed a soluble form of the FGFR (sFGFR) in a transgenic mouse model and demonstrated potent inhibition of FGF-mediated organogenesis [1]. We hypothesised that the expression of such sFGFR inhibits proliferation of prostate cancer cells.

**Materials and Methods** The full-length cDNA for FGFR1-IIIc was used as template in a polymerase chain reaction, using primers for the extracellular domain of the receptor. The construct obtained was subcloned into a selectable adenoviral expression system with a green fluorescent protein (GFP) reporter [2]. Western blot analysis was used to verify expression of the sFGFR1 in 293 cells and human prostate cancer DU145 cells infected with the generated recombinant adenovirus (Ad-IIIcR1), and to examine the effect of sFGFR1 on FGF-induced MAP kinase phosphorylation. The effect of sFGFR1 on proliferation of DU145 cells was assessed by WST-1 assay.

**Results** Expression of the activated MAP kinase, pERK1, induced by exogenous FGF-1/heparin was almost completely abolished in Ad-IIIcR1-infected DU145 cells in contrast. Control-infected cells showed no such effect. Proliferation of Ad-IIIcR1-infected DU145 cells maintained in full culture media was reduced by almost 50% in comparison with controls.

**Conclusions** We have demonstrated for the first time that expression of soluble FGFR in human prostate cancer cells may have a potential role in the development of therapy. This model will be further developed for in vivo assessment.

### REFERENCES

1. Celli *et al*., (1998) *EMBO*; **17**: 1642
2. He *et al*., (1998) *PNAS*; **95**: 2509

### P211 MATRIX METALLOPROTEINASE 9 EXPRESSION IN PROSTATE TUMOUR AND STROMAL CELLS

MJ Thompson, J Crimmins, S Ganta and PM Loadman, Cancer Research Unit, University of Bradford, Bradford, West Yorkshire BD7 1DP

Prostate cancer is the 2nd most common cancer in males in the UK with over 18,000 new cases registered per year (CRC figure for 1995). Tumour progression in prostate cancer is a common occurrence that often accompanied by metastasis and leads to a largely untreatable disease state. Tumours that lack metastasis present with a good 5 year survival rate. Past work has demonstrated that MMP9 mRNA is upregulated in the more aggressive, metastatic prostate tumours. Similarly, western blotting has shown an increase in MMP9 protein within tumour tissues. The work presented here aims to examine the cellular localisation of the MMP9 protein within prostate tumour tissue and to begin to determine the extent to which stromal cells contribute to the observed MMP9 expression.

Archival paraffin embedded tumour tissues were obtained from the Bradford Royal Infirmary NHS Trust. The tissues were paired into pre and post androgen independence for each patient and tumour areas were identified in consultation with relevant urological and pathological clinicians. Serial sections were then immunostained for MMP9 and CD68 (a macrophage specific marker) using standard methods. Scoring was carried out blind by two researchers.

MMP9 expression was examined in tumour cells and found to be positive (where >90% of tumour cells were stained) in 56% of the cases (18/32). A lower level of staining (where 80–90% of cells were positive) was observed in an additional 12.5% of cases (4/32). In a majority (90%) of cases (29/32), strong MMP9 expression was noted in CD68 positive macrophages present within the tumour tissues. This study therefore demonstrates that in this sample, immunodetectable MMP9 expression does not appear to significantly alter when prostate tumours progress from the hormone dependent to hormone independent state. The expression of MMP9 within these tumours is shown to arise from both the tumour cells and from normal, stromal macrophages. The manipulation of MMP9 activity in prostate tumour or stromal cells may therefore be a viable therapeutic strategy.

This work was supported by the Cancer Research Campaign

### P212 DIFFERENTIAL EXPRESSION OF ECE AND NEP METALLOPROTEINASE IN PROSTATE CANCER,

BA Usmani[1], NJ Maitland[2], DM Nanus[3], AJ Turner[1], [1]School of Biochemistry & Molecular Biology, University of Leeds, Leeds LS2 9JT, [2]YCR Unit, Dept. of Biology, University of York YO10 5YW, [3]Depts. of Medicine & Urology; Weill Medical College of Cornell University; NY, NY 10021

Prostate cancer is now the most common cancer and the second highest cause of cancer death in men in Western society. Recently, clinical and pre-clinical data have indicated a mitogenic role for neuropeptides in prostatic disease. Endothelin-1 (ET-1) is produced by prostatic epithelium and prostate cancer cell lines, and plasma ET concentrations are significantly elevated in men with metastatic prostate cancer. Therefore, a prospective therapeutic approach might be to block ET-1 activity in prostate cancer through the modulation of the Endothelin-Converting-Enzyme (ECE), a metalloproteinase which converts the inactive precursor (big ET) to the active ET peptide. Recently, the consequence of loss of a related cell surface peptidase, NEP, has been shown to contribute to androgen-independent progression of human prostate cancer (Papandreou, CN, Usmani, BA, *et al*. (1998) *Nature Medicine* 4:50–57). The present study aims to examine ECE expression in prostate cancer, the cellular localization of ECE, and whether changes in ECE expression may contribute to androgen-independent disease. Established prostate epithelial cell lines from transformed (PNT1a, PNT2-C2, P4E6) and tumour (LNCaP, PC-3, DU145, TsuPr1, PPC-1) origins were utilized in this study, as well as available tumour biopsy and radical prostatectomy material. Western analysis revealed that ECE-1 is abundantly expressed in all metastatic cell lines, but weakly expressed in LNCaP cells. In contrast, LNCaP cells were seen to express high levels of NEP, whilst PC-3, DU145 and other metastatic cell lines tested lacked detectable NEP activity. Primary epithelial cells of BPH origin expressed much higher levels of NEP than their malignant counterparts, but neither showed ECE expression. An absence of epithelial ECE might indicate a role for stromal interaction and paracrine production of ECE within the host. Immunohistochemical analysis does in fact reveal stromal ECE activity in primary prostate tumour tissue. In contrast, the up-regulation of ECE in metastatic epithelial cell lines in culture may further indicate a critical role for ECE in ascerting a metastatic phenotype.

In conclusion, a differential profile of NEP and ECE would allow for an abundance of mitogenic peptides contributing to the progression of prostate cancer. ECE is up-regulated in stromal cells surrounding tumour tissue. Paracrine up-regulation of ECE leads to an increase in mitogenic endothelin peptide concentration, and this may be a critical early step during the progression of androgen-independent prostate cancer.

### P213 ABNORMAL EXPRESSION OF MITOGEN-ACTIVATED PROTEIN KINASE KINASE-5 (MEK5) IN HUMAN PROSTATE CANCER,

PB Mehta, R Brown, L Thilak, AB Pulimood, CN Robson, DE Neal and HY Leung, e-mail: H.Y. Leung@ncl.ac.uk. School of Surgical and Reproductive Sciences, The Medical School, University of Newcastle upon Tyne, NE2 4HH, UK

We applied the technique of arbitrarily primed Polymerase Chain Reaction (PCR) to genotype variants of the human LNCaP prostate cancer cell line that have different metastatic phenotypes (LNCaP-Pro5 and LNCaP-LN3 (most invasive)). A DNA fragment (Prosl), lost in the LNCaP-LN3 variant, had a perfect match to the human mitogen-activated protein kinase kinase-5 (MEK5) gene.

Immunoprecipitation and western blotting confirmed the loss of MEK5 protein expression in LNCaP-LN3 cells, when compared to the parental variant LNCaP lines. Tissue in situ hybridisation (TISH) and immunohistochemistry (IHC) demonstrated abnormal expression of MEK5 in the malignant epithelium while adjacent areas of BPH were negative. Occasional signal was observed in the basal cells of some BPH cases. Data from both TISH and IHC studies suggest highest levels of MEK5 expression in about 50% of prostate cancer, with no significant difference observed among tumours of different Gleason scores. Furthermore, stromal smooth muscle express MEK5 at low levels, in keeping with a physiological role of MEK5 in the maintenance of myocyte lineage.

To further investigate its potential roles in invasion, a constitutively active form of MEK5 (MEK5D) was co-transfected with matrix metalloproteinase (MMP) promoter driven luciferase reporter constructs into COS-7 cells. The promoters for MMP-1, -2 and 9 were examined. MEK5D stimulated MMP-9 promoter the greatest, MMP-1 moderately, but had no effect on MMP-2 promoter activity. In summary, we identified *MEK5* as a gene potentially associated with increasing invasiveness in prostate cancer cells and confirmed its abnormal expression in the resected primary tumour specimens. Of interest, MEK5 may be an important signalling molecule required in the induction of MMP-1 and MMP-9 genes.

### P214 A STUDY OF SURVIVIN AND $p21^{CIP1/WAF1}$ EXPRESSION IN PROSTATE CANCER,

MJ Thompson, S Ganta & J Seargent, Cancer Research Unit, University of Bradford, Bradford, West Yorkshire BD7 1DP

Prostate cancer is a common male cancer that is increasing in both incidence and mortality. Treatment is initially successful via withdrawal of androgenic hormones, but a relapse into an androgen independent state is commonly observed and difficult to treat. The initial response of the prostatic tumours to hormonal withdrawal is via apoptosis. The novel antiapoptosis protein survivin is a member of the inhibitor of apoptosis protein (IAP) family that links apoptosis and cell cycle progression, ensuring that cells maintain viability and proper cell cycle progression. It is expressed normally in the G2/M phase of mitosis, associating with the mitotic spindle microtubules and the CDK inhibitor $p21^{cip1/waf}$ within the centrosome. Over-expression of survivin therefore appears to be a mechanism by which cells can produce an antiapoptotic environment through their cell cycle. This work aims to study whether the expression of survivin is related to prostate tumour progression from being hormone dependent to a hormone independent state and to determine whether survivin expression was associated with $p21^{cip1/waf}$ expression.

Archival paraffin embedded tumour tissues were obtained from the Bradford Royal Infirmary NHS Trust. The tissues were paired into pre and post androgen independence for each patient and tumour areas were identified in consultation with relevant urologists and pathologists. These were then immunostained for survivin and $p21^{cip1/waf}$ using standard methods. Scoring was carried out blind by two researchers. Survivin expression was found in a majority of tumours (35/38). When the paired tissues were examined, no significant difference was found between the scores for the pre (1.61 ± 0.29) and post-relapse (1.33 ± 0.26) states. Similarly, $p21^{cip1/waf}$ expression was observed in a majority of tumours (37/38) but no significant difference was observed between pre (2.17 ± 0.24) and post-relapse (1.85 ± 0.29) groups. This work demonstrates that survivin is expressed by a majority of prostate tumours but this is not associated with androgen independence or $p21^{cip1/waf}$. Manipulation of survivin function may therefore represent a relevant approach in the treatment of prostate cancers.

This work was supported by the Cancer Research Campaign

### P215 THE MOLECULAR DETECTION OF PROSTATE CANCER

K Patel[1], P Harndon[1], P Whelan[1], PJ Selby[1] and S Burchill[1]
[1]Imperial Cancer Research Fund Clinical Cancer Centre, St. James's University Hospital, Leeds, UK. e-mail: medkp@cancermed.leeds.ac.uk

Sensitive methods for the detection of prostate cancer may be useful in defining prognosis and in directing future therapeutic strategies. Reverse-transcriptase polymerase chain reaction (RT-PCR) for PSA mRNA has been extensively studied in patients with prostate cancer with variable results. This study evaluated the sensitivity and specificity of RT-PCR and compared it to microsatellite analysis for the detection of prostate cancer in the peripheral blood (PB).

RT-PCR for PSA was performed on PB from 21 patients with metastatic prostate cancer and 24 healthy volunteers' blood samples. Ten of the patients with metastatic disease already evaluated by RT-PCR were examined for microsatellite instability (MSI) and loss of heterozygosity (LOH) in their plasma using 14 polymorphic and 2 monomorphic fluorescently labelled markers. Microsatellite analysis was also performed on plasma samples from 9 healthy volunteers.

RT-PCR for PSA was very sensitive, detecting 10 prostate cancer cells spiked in 1 ml of PB, although PSA mRNA was only detected in the PB samples from 6/21 patients. Furthermore, PSA mRNA was detected in normal PB (4/24 samples). MSI and LOH were detected in 6/10 prostate tumours, but only LOH was detected in matching plasma samples from 2/10 patients. These two patients were also RT-PCR-positive for PSA mRNA in the PB.

In summary, RT-PCR for PSA is a sensitive method for the detection of prostate cancer cells, however the detection of PSA mRNA in normal PB may limit its clinical utility. Microsatellite analysis is specific for the identification of disease but detection of tumour derived DNA is low in patient plasma samples. The role of real-time quantitative RT-PCR for PSA mRNA in distinguishing between healthy and diseased populations will be evaluated and the results will be presented.

### P216 EXPRESSION OF TIP60, AN ANDROGEN RECEPTOR CO-ACTIVATOR, IN PROSTATE CANCER

K Chalkidou, VJ Gnanapragasam, ME Brady , S Cook , DE Neal , CN Robson, Dept. of Surgery, University of Newcastle Medical School, Newcastle-Upon-Tyne, NE2 4HH

Prostate cancer is currently the second leading cause of cancer deaths in males. While early treatment is by androgen ablation, there are few treatments for recurrent disease. Recent evidence has suggested that the androgen receptor (AR) continues to be active in hormone refractory cancer and one of the mechanisms may be by overactivity of steroid receptor coactivators. We have previously identified Tip60 as a coactivator of the human AR showing that it enhances AR dependent transactivation and interacts physically with the AR in vivo. The role and expression of Tip60, in clinical prostate cancer however is still unknown. We were therefore interested in investigating Tip60 expression in both benign and malignant prostatic tissue.

**Materials and Methods** ***In situ hybridisation*** In vitro transcription was performed from Tip60 cDNA to generate anti-sense and sense probes using digoxigenin labelling. *Anti-Tip60 Ab*: A rabbit polyclonal anti -Tip60 antibody was raised using a C terminal Tip60 peptide. Tip60 antiserum was then isolated and affinity purified on a peptide column. *Western blot*: Western blot was performed on COS 7 (monkey kidney) cells transfected with HA-tagged Tip60 or plasmid vector control. Plasmid expression was confirmed by probing for the HA tag. *Immuno-histochemistry*: Purified antibody was used to study the expression of Tip60 in clinical cases of paraffin embedded BPH and prostate cancer (sections obtained by TUR biopsies) using an Avidin-Biotin staining system.

**Results** In situ hybridisation showed that Tip60 was expressed at the transcript level in prostatic tissue. Malignant tissue demonstrated weak but positive expression of Tip60 mRNA in some cells. In benign tissue there was more convincing staining of glandular basal cells. In both cases stroma exhibited weak non-specific staining. In the western blot, Tip60 antibody produced a single band of the predicted size in HA-tagged Tip60 transfected COS 7 cells, but not in un-transfected cells. Expression levels correlated with a probe for the HA tag. Immunohistochemistry demonstrated that in sections of BPH, Tip60 localised weakly to epithelial cells with predominantly nuclear staining. In malignant epithelium, there was more intense staining of nuclei with scattered stains in the cytoplasm. Controls using no primary antibody exhibited no staining.

**Conclusion** We have identified Tip60 mRNA expression in prostatic tissue. We have also produced and characterised a novel Tip60 antibody and confirmed protein expression in both benign and malignant tissue. Further data will be presented of Tip60 expression in prostate cancer and correlation with stage and grade of disease.

### P217 POTENTIAL OF INTERMITTENT HORMONE THERAPY FOR M+ AND M0 PROSTATE CANCER PATIENTS

RTD Oliver[1], A Lee[2], D Farrugia[1], W Ansell[1], F Chinegwundoh[2] Dept of Medical Oncology[1], and Urology[2], St Barts & Royal London School of Medicine, London ECIA 7 UK

Increasingly animal and clinical studies suggest that intermittent therapy may improve duration of hormone dependence in patients with prostate cancer. However there remains uncertainty as to optimal duration of treatment, level of PSA before treatment is restarted and whether it is safe to use this strategy in patients with M0 disease. The aim of this study is to compare outcome in prostate cancer patients with M+ and M0 disease receiving intermittent hormone therapy.

**Patients and methods** Any patients who had achieved PSA complete remission after hormone therapy for metastatic or locally advanced prostate cancer were included in the study. Patients restarted treatment when symptoms developed or if PSA rose above pretreatment levels.

**Results** Fifty patients (M+ n = 17, M0 n = 33) entered on intermittent hormone therapy (HT) after achieving a PSA complete remission. Overall 57% remain off treatment at 12 months and the median time for restarting further hormone therapy was 14 months. 95% of patients retreated are progression free at 1 year and overall 92% are alive at 3 years. 16 patients have completed a second cycle, 12 a third cycle and 3 have begun a 4th cycle.

There was a slower progression to require treatment in M0 v M+ patients (70% v 37% continue off hormones at 1 year) Furthermore in a subset of four M0 patients with a short first cycle time off HT alone (2,7,7,13 mean 7.3 mths) radiation consolidation prolonged their second cycle off HT (19, 14, 19, 28 mean 20 months). Despite this small sub-group of early relapses, overall 53% of M0 patients treated without radiation remained off hormones at 3 years.

Prompted by these findings a randomised phase 2 study has been initiated to investigate immediate versus deferred radiation in M0 patients who received 9 months intermittent hormone therapy. To date 52 have been randomised and currently at 6 months 1 of 12 hormone alone patients versus 1 of 11 hormones plus radiation patients have achieved PSA complete response.

**Conclusion** It is clearly safe to consider patients with M0 prostate cancer for intermittent hormone therapy alone. Furthermore there is a suggestion that it may be safe to restrict radiation to consolidate response of the minority having a short duration of time off therapy in first cycle, though a randomised phase 2 trial will be required to confirm this.

### P218 ANDROGEN RECEPTOR AMPLIFICATION IN HORMONE REFRACTORY PROSTATE CANCER

J Edwards[1], MA Underwood[2], NS. Krishna[1], R. Mukherjee[2], AD. Watters[1] and JMS Bartlett[1], Dept of Surgery[1]/Dept of Urology[2], Glasgow Royal infirmary, Glasgow, G31 2ER

To aim of this study was to examine the role of AR gene amplification and aneusomy of chromosome X in the development of anti-androgen resistant prostate cancer.

Twenty patients with recurrent prostate cancer resistant to androgen deprivation therapy were selected for study. Pre and post anti androgen therapy tumours and full clinical follow-up were retrieved for each patient. AR gene amplification and X chromosome copy number were assessed by fluorescent *in situ* hybridisation (FISH) using a Spectrum Orange™ labelled probe at locus Xq11–13 for the AR gene (Vysis, UK, Ltd) and a Spectrum Green™ labelled alpha satellite probe for the X chromosome (Vysis, UK, Ltd). A minimum of 20 nuclei were scored over 3 tumour areas by 2 independent observers.

Aneusomy of chromosome X was reported in 35% (7/20) pre and 55% (11/20) post relapsed tumours and AR gene copy number was 2 or more in 35% (7/20) of pre and 65% (13/20) of post relapse tumours. AR gene amplification was found in 5% (1/20) of pre relapse tumours and 15% (3/20) of post relapsed tumours.

The rate of AR gene amplification is too low to be solely responsible for the development of anti-androgen resistant prostate cancer. Also the presence of amplified AR and cells aneusomic for the X chromosome in primary tumours that respond to androgen deprivation therapy suggests that an increase in AR gene copy number does not prevent a tumour from responding to androgen deprivation therapy. Therefore protein levels of AR and other mechanisms which could cause hormone refractory prostate cancer must be investigated before we understand why so many patients with this disease relapse with an anti-androgen resistant tumour.

### P219 PHASE I TRIAL OF *bcl-2* ANTISENSE (GENASENSE™) AND DOCETAXEL (D) IN HORMONE-REFRACTORY PROSTATE CANCER

JS de Bono, EK Rowinsky, J Kuhn, L Ochoa, G Schwartz, A Patnaik, H Fingert, S Weitman, I Thompson, AW Tolcher, Institute For Drug Development, University of Texas at San Antonio, Genta Inc, Berkeley Heights, NJ

Hormone refractory prostate cancer (HRPC) is relatively resistant to therapeutics that induce apoptosis due, in part, to the proto-oncogene *bcl-2*, which is overexpressed in the majority of HRPC and which appears to be a determinant of androgen-independence and chemoresistance. D has, however, been shown to induce PSA responses in patients with HRPC. Genasense™ (G), an antisense oligonucleotide directed to the first 6 codons of bcl-2 mRNA, downregulates Bcl-2 protein expression and enhances D chemosensitivity in prostate cancer xenografts. This rationale is the basis for this phase I pharmacokinetic (PK) and pharmacodynamic (PD) study of G3139 administered prior to treatment with D in patients with HRPC. In this study, G is administered as a continuous IV infusion for 5 days followed by D administered over 1 hr, with cycles repeated every 21 days. Sixteen patients with HRPC (median age – 66, median PS 1) have received 50 courses with escalating dose levels: G 5 to 7 mg/kg/day with D 60 to 100 mg/m$^2$. Four patients had received prior radiotherapy and 7 patients prior chemotherapy including 3 patients with prior taxane therapy. Toxicity has not been dose limiting with uncomplicated grade 4 neutropenia in 4 patients, grade 1 fever in 5 patients and grade 1 mucositis in 2 patients. No drug-drug PK interactions have been noted, as both D and G PK parameters have been similar to PKs in single-agent studies. G plasma concentrations have consistently exceeded target levels in preclinical studies (1 μg/ml) with $C_{ss}$ values averaging 3.09 and 5.36 μg/mL at the 5 and 7 mg/kg/day dose levels, respectively. Both flow-cytometric and western blot analyses indicate marked downregulation of Bcl-2 protein by day 5 in peripheral blood mononuclear cells. To date, durable PSA responses have been seen in 4 of 8 taxane-naïve patients, including a 50-fold reduction in PSA and major objective responses in liver and viscera. Genasense can be safely administered in combination with docetaxel, effectively downregulating Bcl-2 protein in vivo. The preliminary antitumor activity and safety data support expanded clinical development for patients with HRPC.

### P220 COMBINED EXTERNAL BEAM AND BRACHYTHERAPY BOOST FOR LOCALISED PROSTATE CANCER: A REPORT OF PROCEDURAL COMPLICATIONS AND ACUTE RADIATION TOXICITY

J Dickson[1], P Bownes[1], P Ostler[1] and PJ Hoskin[1], [1]Mount Vernon Cancer Centre, Rickmansworth Road, Northwood, Middlesex, HA6 2RN

**Introduction** In patients with apparently localised prostate cancer, conformal radiation therapy using combined external beam (EBT) and temporary implantation (BB) has been developed at Mount Vernon, covering potential sites of microscopic disease in the extracapsular region and seminal vesicles with 35.7Gy in 13 daily fractions EBT, followed by a fractionated HDR implant delivering 17Gy in 2 fractions. This is currently being compared with EBT alone in a randomised phase III trial. This series reports our initial experience in seventeen patients refusing randomisation who were treated off-study, but according to protocol.

**Materials and Methods** Data were collected retrospectively from patient notes using a standard pro-forma: this covered patient demographics, treatment details, procedure-related complications and acute radiation toxicities. Data were recorded on Info flex 4, with analysis using SPSS.

**Results** Results are available from seventeen patients, with a median age at treatment of 67 years (range 53–74). Eight patients (47%) were asymptomatic at diagnosis. Five patients had stage I and II stage II disease. Disease stage was unavailable in 1 patient. Median PSA at presentation was 14.2 ng/ml (range 4.6–38.7). All patients were treated radically, according to protocol. The median planning target volume, as defined by CT, was 102 cc (range 49–137). The maximum urethral dose ranged from 15–22.2Gy over two fractions (median 20.1). The median maximum anterior rectal wall dose was 12.7Gy, over two fractions. Eight patients (47%) required narcotic analgesia whilst the implant was in situ. No patient had any other procedure-related complication. Initial follow-up was at a median of 4 weeks post-treatment (range 2–6). Five patients had significant urinary symptoms at this time: 1 had dysuria and 4 had frequency during the day of every 2 hours or less. Three patients had bowel symptoms: 1 had PR bleeding and 1 had mucous rectal discharge, both intermittent. One patient required regular medication to control his bowel habit. Data concerning late toxicity was available on ten patients with a median follow-up of 12 months (range 6–20). No patient had biochemical disease relapse. Three patients had RTOG grade I/II urinary symptoms and bowel symptoms, respectively.

**Discussion** This series represents an initial overview of the procedural complications and acute radiation morbidity in 17 patients treated off-study with combined EBT and BB. The procedure is generally well tolerated. Acute radiation morbidity is acceptable when compared with standard external beam protocols. Further follow-up will define rates of tumour control and late radiation morbidity.

### P221 ANORECTAL IRRADIATION DURING RADIOTHERAPY FOR CARCINOMA OF THE PROSTATE: A COMPARISON OF HELAX PREDICTED AND DIODE MEASURED DOSES

D Hayne[1], U Johnson[2], R Brown[3], D D'Souza[2], C Hare[4]. PB Boulos[1], H Payne[3], [1]Dept of Surgery, [2]Dept of Medical Physics, [3]Meyerstein Institute of Oncology' [4]Dept. of Radiology Royal Free and University College Medical School London

**Objectives** To validate anorectal radiation doses predicted by a HELAX™ treatment planing system by comparing them to doses measured using a Scanditronix rectal probe containing 5 n-type photon-detecting diodes.

**Background** Anorectal symptoms after radiotherapy for prostate cancer are extremely common and cause significant morbidity. The degree of anal canal irradiation and its effect on subsequent symptoms has not been investigated. For this purpose an accurate assessment of anorectal dose was required.

**Methods** Ten patients were CT planned with the probe in the anorectum and HELAX™ was used to predict doses for each of the diodes and to determine the relation of each diode to the target volume (in the volume, at the edge of the volume, outside of the volume). Doses were then measured with the probe on 5 consecutive fractions and the average diode-measured doses compared with those predicted by HELAX™.

**Results** For diodes situated in the target volume, the average difference between measured and HELAX™ predicted doses was 6%. Large differences were found between measured and predicted doses in diodes at the edge and outside of the target volume (average variation compared to HELAX™ predicted dose was 55% and 40% respectively). Diodes outside the target volume were higher than the HELAX™ predicted doses, to a statistically significant degree ($P < 0.022$).

**Conclusions** HELAX™ predicted doses appear accurate within the confines of the target volume. Probe movement, set-up error and internal organ movement may explain the large variation between measured and predicted doses for diodes not within the target volume. Due to the averaging of doses on five consecutive fractions, measured doses are likely to represent a more accurate reflection of anorectal irradiation over an entire treatment period.

### P222 USE OF $^{14}$C METHYL CHOLINE TO MONITOR INHIBITION OF THE EXTRACELLULAR SIGNAL-REGULATED KINASE CASCADE: A NOVEL APPROACH TOWARDS IMAGING OF SIGNAL-TRANSDUCTION INHIBITION

OC Hutchinson[1], D Liu[1], S Osman[2], P Price[1] P Workman[3] and EO Aboagye[1], [1]PET Oncology Group, Imperial College School of Medicine, London, [2]MRC Cyclotron Unit, Hammersmith Hospital, London; [3]CRC Centre for Cancer Therapeutics, Institute of Cancer Research, Sutton

**Background** Phosphocholine levels are found to increase with malignant transformation of mammary epithelial cells in vitro. There is evidence to suggest that the phosphocholine production is regulated by the receptor-Ras-Raf-MAPK-kinase (MEK)-Mitogen Activated Protein Kinase (MAPK) cascade. In this study, the regulation of choline phosphorylation by the MAPK pathway was investigated by using signal transduction inhibitors. The ultimate aim is to develop and validate [methyl-$^{11}$C]choline as a positron emission tomography (PET) probe for quantifying the pharmacodynamics of signal transduction inhibition.

**Methods** We studied the effects of Geldanamycin and 17 AAG (Raf-1 inhibitors), U0126 (specific MEK inhibitor), Roscovitine (cyclin-dependent kinase-2 inhibitor), and LY294002 (PI3K Inhibitor) on choline uptake and phosphocholine production. In parallel experiments, the effects of these drugs on the phosphorylation of extracellular signal-regulated kinase (ERK-1/2), and cell proliferation were also assessed.

**Results** In exponentially growing HT29 cells, inhibition of ERK-1/2 phosphorylation by Geldanamycin and U0126 was associated with a concentration-dependent reduction in choline uptake, phosphorylation production and cell proliferation. Brief exposure to high concentrations (100 μM) of U0126 inhibited phosphocholine production to the same extent as Hemicholinium-3 (1 mM), an inhibitor of choline transport and choline kinase activity. In contrast to these inhibitors which act upstream of MAPK, Roscovitine (1,10 μM) failed to suppress phosphocholine production. Furthermore, LY294002 (5,50 μM) also failed to suppress phosphocholine production.

**Conclusion** Choline phosphorylation is regulated by the MAPK cascade. This suggests that using [methyl-$^{11}$C]choline maybe useful as a PET probe for the in vivo quantification of pharmacodynamics of signal transduction inhibitors that act on the receptor-MAPK cascade.

### P223 DIAGNOSTIC ACCURACY OF DIFFUSION WEIGHTED IMAGING IN THE PROSTATE

P Gibbs, DJ Tozer, LW Turnbull MRI Centre, Hull Royal Infirmary, Anlaby Road, Hull HU3 2JZ

**Introduction** Over the past few years MR imaging of the human prostate has become more prevalent. However, due to the similarity in signal intensity between prostatic carcinoma (PCa) and benign prostatic hyperplasia (BPH) on T2 weighted imaging, the usefulness of conventional MRI is reduced. An alternative approach may involve the utilisation of diffusion weighted imaging, which has seen increasing clinical relevance in the brain. Diffusion weighting involves sensitising the imaging sequence to the random motion of extracellular water. Variations in apparent diffusion coefficient (ADC) in malignant tissue are often attributed to changes in the extracellular volume fraction and increased tortuosity of the extracellular space.

**Methods** All imaging was performed using an IGE 1.5 T scanner and a commerical pelvic phased array coil. Seventy-six patients with transrectal ultrasound guided biopsy proven prostatic carcinoma aged between 51 and 83 years (mean 68 years) were scanned. Diffusion weighted images of a single axial slice through the prostate were acquired. Data was obtained with diffusion gradients applied along each of the three main axes in turn. For each direction 8 images were obtained at different diffusion weightings to enable accurate quantification of the ADC. The total scan time was just under 27 minutes. After acquisition regions of interest were drawn on areas of PCa, BPH, and normal peripheral zone (PZ) tissue where available. Calculated ADC values were then compared across tissue types.

**Results** Significant differences were noted between all tissue types in the x- and y-directions ($P < 0.001$ for all comparisons). In the z-direction a significant difference was noted between PCa and PZ ($P < 0.0001$). The results are presented in the accompanying table.

| Tissue | $ADC_x$ | $ADC_y$ | $ADC_z$ |
|---|---|---|---|
| PCa | 2.23±1.05 | 2.32±0.91 | 4.16±1.63 |
| BPH | 2.39±0.81 | 2.65±0.91 | 4.33±1.59 |
| PZ | 2.95±1.09 | 3.02±1.16 | 4.84±1.78 |

ADC values were noted to be significantly higher in the z-direction for all tissue types indicating that diffusion anisotropy may be important in the prostate.

**Conclusions** Diffusion weighted imaging of the prostate may offer improved diagnostic accuracy compared to conventional MR imaging.

### P224 ROLE OF MR IMAGING IN PROSTATE CANCER: CORRELATION WITH PSA AND GLEASON GRADE.

B Wang, M Lowry & L W Turnbull, Centre for MR Investigations, Hull Royal Infirmary, Anlaby Road, Hull HU3 2JZ

**Introduction** Prostate cancer is the second most common cause of cancer-related death in the UK. Although up to 40% of men will have prostate cancer by the age of 60 years, not all will develop progressive or metastatic disease. The characteristics of lesions associated with a poor prognosis are urgently being sought with a view to optimising patient management. Tumours grow by induction of neoangiogenesis. Pharmacokinetic modelling of dynamic contrast-enhanced magnetic resonance imaging (DCE-MRI) allows analysis of amplitude of contrast uptake, estimation of permeability (ER) and the distribution volume (DV). Empirical techniques provide a measurement of maximum enhancement index (EI), a parameter related to blood flow. The objective of this study is to determine if a relationship exists between MR-determined parameters of neoangiogenesis and tumour stage (T-stage), size, Gleason grade, PSA level and presence of bone metastases at initial presentation.

**Methods** 78 patients (aged 48–82 yrs; mean 65) underwent MRI at 1.5T using a pelvic phased array coil for signal reception. Thin-slice T2-weighted fast-spin echo images were acquired for T-staging and tumour localisation, estimation of maximum tumour diameter and volume. DCE-MRI was carried out at four slice locations using an FSPGR sequence (temporal resolution 11.2 secs) and Gd-DTPA administered at 0.1 mmol/kg body weight. Regions-of-interest were drawn manually on a Sun Workstation to encompass tumour, whilst excluding areas of benign prostatic hyperplasia and venous plexus. Data analysis was performed using a 2-compartment pharmacokinetic model. Maximum EI was obtained empirically. Gleason grade (GG), prostate specific antigen (PSA) level and radionuclide bone scanning were obtained 2–5 weeks prior to MRI.

**Results** PSA values ranged from 0.6–292 ng/ml (mean 42) and GG from 2–10 (mean 6). Strong correlations were obtained between T-stage and both GG and PSA ($P < 0.000$ and 0.002) and between tumour diameter/ volume and GG and PSA ($P <$ 0.000 & 0.002; 0.028 & 0.037 respectively). No significant correlations were demonstrated between GG, PSA or T-staging and MR parameters of angiogenesis. Moderate correlations were obtained between maximum tumour diameter and EI and DV ($P = 0.05$ and 0.03 respectively) for patients with no bone metastases, but no correlation found for those with bone metastases.

**Conclusions** Strong correlations have been demonstrated between MR-defined tumour size, T-stage, GG and PSA at presentation, but MR-defined parameters of neoangiogenesis do not appear to be useful determinants of future potential for progressive disease.

### P225 MONITORING CHEMOTHERAPY IN BREAST CANCER USING QUANTITATIVE MR IMAGING

DJ Manton[1], D Tozer[1], A Maraveyas[1], A Chaturvedi[2], J Greenman[1], L Cawkwell[1], A Hubbard[3], A Modi[4], M Lind[1], & LW Turnbull[1], [1]Faculty of Health, University of Hull, East Riding Campus, Willerby, HU10 6NS;[2]Oncology Dept., Princess Royal Hospital, Salthouse Rd., Hull, HU8 9HE, [3]Breast Screening Unit, Castle Hill Hospital, Castle Rd., Cottingham, HU16 5JQ,[4]Consultant breast surgeon, Castle Hill Hospital, Castle Rd., Cottingham, HU16 5JQ

Change in tumour volume may be a relatively late manifestation of response to chemotherapy in patients with inoperable primary breast cancer. Recent studies[1,2] have suggested that using MRI to quantify water apparent diffusion coefficient (ADC) or microvessel permeability, or alternatively using proton MR spectroscopy (MRS) to quantify the relative water and fat signal magnitudes may be able to provide an early indication of ultimate treatment response. Therefore a study was designed to compare the reliability of these three methods in women undergoing neoadjuvant chemotherapy for inoperable breast cancer.

Five women have so far been recruited into the study who have fully completed their course of treatment. All women received a standard dosage chemotherapy regime involving intravenous administration of 5-Fluorouracil, Epirubicin and Cyclophosphamide. MRI and MRS was carried out prior to chemotherapy, between the second and third courses (TP2) and shortly after the final (fourth) course. ADC was measured using an EPI sequence producing eight images with diffusion gradient weightings up to 680 s/mm$^2$. Microvessel permeability was measured using a 7.5 minute long $T_1$-weighted fast spoiled gradient echo (FSPGR) sequence (temporal resolution of 13 s) combined with a 3 compartment pharmacokinetic model. The proportion of MRS signal arising from water (i.e. PW = water / {water+ fat}) was measured using a single voxel STEAM sequence (TE 135 ms) combined with phase encoding to give seven 0.25 ml voxels in a column through each tumour. Tumour volume was measured using manually traced regions of interest drawn on high resolution 3D, post-contrast, fat-suppressed FSPGR images.

Preliminary results demonstrated that all 5 tumours responded (median final:baseline volume ratio = 12%; range = 5% to 32%). Both the TP2:baseline volume ratio (44%; 9% to 64%) and the corresponding permeability ratio (25%; 5% to 56%) accurately predicted response (by being less than 100%) in all 5 cases. The corresponding ADC (115%; 49% to 147%) and PW (97%; 89% to 110%) ratios predicted response (by being > 100% and < 100% respectively) in only 3/5 and 4/5 cases suggesting that they may be less reliable indicators of final treatment efficacy.

1. M Zhao, *et al* (1996) *Br. J. Cancer* **73**: 61
2. N Tsuboi, *et al* (1999) *Oncology Reports* **6**: 727
3. NR Jagannathan, *et al*. (1998) *NMR in Biomedicine* **11**: 414

### P226 THE ROLE OF FDG WHOLE BODY POSITRON EMISSION TOMOGRAPHY IN METASTATIC CARCINOMA OF UNKNOWN PRIMARY SITE

M Osborne, A Makris, PJ Hoskin, J Lowe, J Emmott, WL Wong, Mount Vernon Hospital, Northwood HA6 2RN, UK

**Aim** 5–6% of new cancer cases present as cancer of unknown primary site. They represent a heterogeneous group of tumours, often requiring extensive investigation. The subgroup of patients presenting with extracervical metastases tend to have a worse prognosis and whole body imaging with a non-invasive modality in this group is appealing. The aim of this study was to evaluate the role of FDG PET in establishing a primary tumour in this group and assess its influence on their management.

**Patients and Methods** 25 patients (13 male, 12 female), mean age 58 years (range 35–86), with histologically (17) or cytologically (8) confirmed metastatic carcinoma were imaged with whole body PET following conventional work up which failed to reveal the primary origin. Sites of presenting metastases were liver (8 patients), bone (5), nodes (4), ascites (2), and mediastinum, scalp, brain, pelvis, chest wall, abdominal wall (1 each). Investigations prior to PET scanning included CT scanning in all cases and mammography, endoscopy, MRI, serum tumour markers and immunohistochemistry according to clinical suspicion of primary tumour site. Whole body scan was performed at one-hour post injection of 350MBq of FDG.

**Results** In all cases the known sites of metastases were confirmed on PET and in 68% patients other metastatic sites were also identified. 14/25 (56%) PET scans suggested a possible primary site – lung (4), pancreas (3), breast (1), renal (1), oesophagus (1), caecum (1), colon (2), stomach (1). Of these, 1 primary was subsequently confirmed. In one case a primary not visible on the original PET scan was subsequently identified and confirmed on biopsy in the pancreas. 9/25 (36%) PET scans had some influence on management-8/25 (32%) supported planned treatment, but only 1/25 (4%) actually altered planned treatment.

**Conclusion** In this group of patients with metastatic carcinoma of unknown primary origin PET identified the known metastases in all cases. Although a possible primary was suggested in 56%, this could not be confirmed in the majority of cases. The influence on patient management was limited.

### P227 COBALT COMPLEXES WITH A TETRADENTATE SCHIFF BASE AS PERSPECTIVE ANTICANCER AGENTS

S Osinsky[1], I Levitin[2], L Bubnovskaya[1], A Sigan[2] and I Ganusevich[1], [1]Inst. exp. Pathol. Oncol., Kiev-03022, Ukraine, [2]Inst. Organoelement Compounds, Moscow, Russia

**Aims** examination of antitumor activity of Co(III) complexes with both a tetradentate Schiff base and a few native nitrogenous ligands in experiments *in vivo*.

**Introduction** It is known that biogeneous ligands (amino acids, nucleic bases, etc.) may substantially enhance or modify physiological activity of metal complexes. We have been studying the influence of extra biogeneous ligands and their close synthetic analogs on the anticancer activity of Co(III) chelates with tetradentate Schiff bases. A moderate anitumor effect of parent compounds lacking such extra ligands was reported earlier (Dori & Gershon, US-Patent, Pat. No5258403; Date of Pat. Nov. 2, 1993).

**Materials & Method** Novel complexes of the common formula $[Co(acac_2en)L_2]X$ (**1**) where $acac_2en$, L and X are (*i*) dianion residue of the Schiff base from acethylacetone and ethylenediamine, (*ii*) nicotinamide (nic), isonicotinamide (i-nic), or histidine, and (*iii*) counter-ion, respectively were synthesized and thoroughly characterized. Such complexes may be reversibly reduced under hypoxia, giving rise to a catalytic autooxidation process involving generation of reactive oxygen species (ROS). The biomedical examinations were carried out using both rat (Guerin carcinoma) and mice [Lewis lung carcinoma (3LL), melanoma B16, adenocarcinoma Ca755] tumors, and **1a** ($NH_3$), **1b** (nic) and **1c** (i-nic) complexes. Level of lipid peroxidation and activity of glutathione system was estimated by biochemical techniques. $^{31}P$ NMR spectra of tissue perchloric extracts were obtained using a Mercury 300 BB *Varian* spectrometer. All experiments has been approved by the regional animal ethics committee.

**Results** The inhibition of primary tumor growth ranged from 50 to 80%. Anticancer effects generally increased along the series **1a** < **1c** < **1b**. **1b** and **1c** displayed very high antimetastatic effect. Namely, inhibition of metastases (IM) by their number (n) and volume (v) was as follows: in the case of 3LL, IM was 67, 84 and 84% (n), and 80, 99 and 95% (v), and in the case of B16, 75, 71 and 65% (n) and 76, 99 and 85% (v) on treatment with **1a, 1b** and **1c**, respectively. Malonyldialdehyde concentration rose by a factor of 3.5 in tumor after **1b** injection, while the increase in liver and kidney was only 1.2- and 2-fold, respectively. Activity of glutathione system decreased by a factor of 2 in tumor without significant changes in normal tissues. It was also shown that **1b** selectively and significantly decreased the bioenergetic status of the tumor.

**Conclusions** (1) selective effects of the complexes on lipid peroxidation and bioenergetic status of tumor support our assumption that they are reduced in tumors containing significant hypoxic regions, which leads to formation of ROS; (2) tested complexes can be regarded as prospective selective anticancer agents; (3) antimetastatic activity of complexes may be also explained by complexes affecting factors

**P227 Cont'd**

controlling metastases, in particular matrix mettaloproteinases and some other ones associated with the tumor microphysiology which is modifyied by complexes as shown earlier.

### P228 17-ALLYLAMINO-17-DEMETHOXYGELDANAMYCIN (17-AAG) DECREASES NPM-ALK STEADY-STATE AND PERTURBS NPM-ALK-DEPENDENT TYROSINE PHOSPHORYLATION BY INHIBITING NPM-ALK/HSP90 COMPLEX

P Bonvini, T Gastaldi & A Rosolen. Clinica di Oncologia Pediatrica, Azienda Ospedaliera di Padova, Padova, 35128, Italy

Anaplastic Large Cell Lymphoma (ALCL) represent a well recognized subgroup of non-Hodgkin Lymphomas, characterized by the expression of the membrane receptor Ki-1/CD30, a member of the tumor necrosis factor (TNF) ligand family. About 40 to 50% of ALCLs posses a specific chromosomal translocation, t(2;5)(p23;q35), that fuses the N-terminal portion of the NPM (or nucleophosmin) gene to the C-terminal portion of the ALK (Anaplastic Lymphoma Kinase) gene, a novel orphan receptor of the insulin tyrosine kinase receptor subfamily. This translocation originates the soluble chimeric protein NPM-ALK, or p80, localized both in the nucleus and in the cytoplasm. NPM-ALK shows a constitutive tyrosine kinase activity, which can induce malignant transformation both in vitro and in vivo. However, little is known about the NPM-ALK transduction pathway. In this study, we analyzed the stability and tyrosine phosphorylation state of NPM-ALK, as well as NPM-ALK-dependent tyrosine phosphorylation, in ALCL cell lines treated with the novel hsp90 antagonist 17-AAG. We found that 17-AAG causes NPM-ALK depletion in a dose-and time-dependent manner, but it does not affect the steady-state level of the NPM-ALK-associated proteins Shc, IRS-1, Grb2 or PLCγl. Exposure to 0.5 μM 17-AAG for 24 hours, decreases NPM-ALK steady-state level up to 10–15% of the control level, both in the cytosolic and nuclear compartment, and the protein becomes undetectable with longer exposure to the drug. Furthermore, 17-AAG stimulates NPM-ALK tyrosine dephosphorylation and inhibits NPM-ALK-dependent Shc and PLCγ1 phosphorylation, by inducing loss of NPM-ALK/Shc or NPM-ALK/PLCγ1 association. Interestingly, time-course experiments have shown that the tyrosine phosphorylation level of NPM-ALK declines more rapidly than its total amount, suggesting that NPM-ALK regulation occurs at a postranscriptional level. To further confirm that 17-AAG specifically causes NPM-ALK destabilization, we found that NPM-ALK associates with the molecular chaperone hsp90, and 17-AAG treatment completely down-regulates NPM-ALK/hsp90 complex, as shown by co-immunoprecipitation experiments. Finally, we found that NPM-ALK complexes with the co-chaperone hsc70 as well. However, differently from the NPM-ALK/hsp90 association, the level of hsc70-associated NPM-ALK increases markedly right after 4 hours of AAG treatment, suggesting an hsc70 involvement in NPM-ALK degradation Thus, our findings reveals NPM-ALK as a novel hsp90 client protein and underline the role of this benzoquinone anasamycin in regulating NPM-ALK activity.

### P229 COMPARISON OF THE PLASMA PHARMACOKINETICS OF 17-ALLYLAMINO-17-DEMETHOXYGELDAMYCIN ANALOGUES FOLLOWING CASSETTE AND SINGLE DOSING

NF Smith, FL Raynaud, PM Goddard and P Workman, CRC Centre for Cancer Therapeutics, Institute of Cancer Research, 15 Cotswold Road, Sutton, Surrey, SM2 5NG, UK

17AAG is a benzoquinone ansamycin which binds to and inhibits the molecular chaperone heat shock protein 90, leading to depletion of several oncogenic proteins. Phase I clinical trials of 17AAG as an anticancer agent are currently ongoing. *In vitro* studies have shown that the growth inhibition properties of 17AAG depend upon length of exposure. The aim of this study was to evaluate cassette dosing, the administration of several compounds as a mixture to a single animal, in a series of derivatives of 17AAG by comparing the pharmacokinetic parameters of the same compounds dosed both alone and in combination. 17AAG and four structural analogues differing at positions 11, 17 and 19 of the ansamycin ring (NSC 255110; NSC D682300; NSC D683661; NSC D683663) were administered intravenously to Balb $C^-$ mice as single agents at the maximum tolerated doses and at 5 mg/kg, and as a mixture at 5 mg/kg each. Plasma concentrations were determined by HPLC-MS/MS with multiple reaction monitoring. Pharmacokinetic parameters were evaluated by non-compartmental analysis. 17AAG displayed non-linear pharmacokinetics, with the area under curve (AUC) increasing from 1.2 to 33.1 hr*nmol/mL with a 10-fold increase in dose from 5 to 50 mg/kg. The AUCs were up to 5-fold higher following cassette administration compared to single administration for 17AAG, NSC 255110 and NSC D683661. When administered in combination, none of the analogues displayed a higher AUC than 17AAG (4.9 hr*nmol/mL). However, following administration at the maximum tolerated doses NSC D683663 displayed the highest AUC (44.6 hr*μmol/mL) of the five compounds. The half life and AUC of NSC D683663 were 10-fold higher following single dosing compared to cassette dosing. With the exception of NSC D683663, the ranking of four of the compounds from highest to lowest AUC remained similar whether they were given alone at the maximum tolerated doses or in combination as a cassette (17AAG > NSC 255110 > NSC D683661 > NSC D682300). The observed differences between cassette and single dosing could be due to non-linear pharmacokinetics and/or drug-drug interaction. These issues will be investigated in further studies. In conclusion, cassette dosing has a number of advantages for use in drug development, ie. it increases sample throughput whilst reducing animal usage, but is probably unsuitable for this compound series.

This work was supported by the Cancer Research Campaign

### P230 SYNTHESIS OF MOLECULAR TWIST COMPOUNDS AND EVALUATION OF THEIR INTERACTIONS WITH HIGHER-ORDER DNA STRUCTURES

PM Murphy[1], TC Jenkins[2], RT Wheelhouse[1], [1]School of Pharmacy and 2YCR Laboratory of Drug Design, University of Bradford, BRADFORD, BD7 1DP, UK

Compounds based on un-fused polyaromatic ring systems (e.g. **1**) have been shown to intercalate double stranded DNA. Normally bi-phenyl ring systems cannot adopt a planar conformation; however, when N was substituted for methine in the central ring, the diphenylpyrimidine, **1**, was found to be able to overcome the small steric interactions involved to become planar when an external stacking constraint (crystal packing or DNA intercalation) was applied.[1] The threading distance of the intercalator, estimated from a model built using the crystal structure to be 13.2 Å, is comparable with ethidium, anthraquinones and porphyrins, and is therefore predicted to be a closer match to the larger cross-sections of triplex and tetraplex DNAs than to duplex DNA. Tetraplex structures formed in G-rich DNA by guanine tetrads have been shown to be important in the regulation of cellular replication, whilst triplex DNA formation has been postulated as a specific means to target and thereby inhibit gene expression.

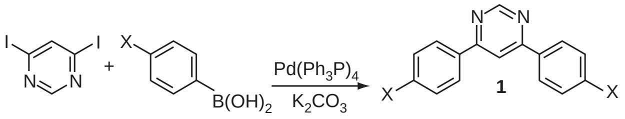

A novel and versatile synthesis utilising a Suzuki coupling reaction has been developed to prepare this class of compounds in greatly improved yields (Scheme). Investigations of their binding interactions with several DNA types will be reported. Structural preferences for DNA binding have been established using equilibrium competition dialysis; binding stoichiometries and thermodynamic parameters have been determined using isothermal titration calorimetry and UV spectrophotometry. These data have been used to inform molecular modelling of the complexes between the ligands and duplex, triplex and tetraplex DNA which may be used as the basis for the design of potential drug compounds to target higher-order DNA structures.

1. W.D Wilson, et al, (1988) *J. Am. Chem. Soc.* **110**, 8292.

### P231 DESIGN OF NEW TOPOISOMERASE I INHIBITORS: SYNTHESIS AND IN VITRO ACTIVITY.

DJ Minchera, G Kaya, S Petterssona, A Turnbull[a] and MC Bibby[b], [a]School of Life Sciences, Napier University, Edinburgh, EH10 5DT, UK, [b]Cancer Research Unit, University of Bradford, Bradford BD7 1DP, UK

We have shown that anthraquinone-amino acid conjugates afford a library of novel topoisomerase inhibitors with broad spectrum anti-tumour activity both in vitro and in vivo. [D.J. Mincher, G. Kay, J.E.L. McDonald, A. Turnbull, M.C. Bibby, J.A. Double, (2000), Clin. Cancer Res., **6**, 11:70]. The type and conformation of a spacer group separating the anthraquinone chromophore from the amino acid/peptide motif modulates relative inhibition of topo I versus the individual α and β isoforms of topo II. We now demonstrate that topo I-selective agents can be produced by insertion of **conformationally rigid** 1,4-diaminocyclohexane or piperizinyl spacer groups. In common with the comparator compound, camptothecin, an L-phenylalanine conjugate and its 2- and 4- chloro derivatives constrained by rigid spacer groups, failed to inhibit topo I mediated relaxation at concentrations up to 100 μM. Like camptothecin these conjugates stabilised topo I cleavable complexes resulting in 40–50% nicked pBR322 plasmid DNA in vitro. The conjugates were actively cytotoxic towards the refractory MAC15A tumour cell line with $IC_{50}$ values of 3.5, 3.0 and 1.0 μM respectively (camptothecin 1.7 μM). Molecular modeling studies show that the novel conjugates can mimic the angular structure of camptothecin and account for the reduced DNA-binding affinity compared to conformationally unrestrained analogues possessing identical peptide motifs.

Evidence for the involvement of drug-stabilised topo I cleavable complexes in the mechanism of cell kill by the conjugates is given by the observed immunoband depletion of topo I in human HL60 cells. For example, the extent of immunoband depletion by the L-phe conjugate at 100 μM was comparable to that observed for camptothecin at 50 μM.

The conjugates constitute a new structural class of topo I inhibitor and exhibit mechanisms of inhibition analogous to the structurally labile camptothecins and merit further pre-clinical evaluation.

### P232 IN VITRO AND IN VIVO-ACTIVE ANTHRAQUINONE PEPTIDE CONJUGATES: A PUTATIVE PHARMACOPHORE FOR COLON TUMOUR SPECIFICITY

S Pettersson[a], A Turnbull[a], G Kay[a], MC Bibby[b] JA Double[b] and DJ Mincher[a*]. [a]School of Life Sciences, Napier University, Edinburgh, EH10 5DT, UK. [b]Cancer Research Unit, University of Bradford, Bradford BD7 1DP, UK

The novel anthraquinonyl L-proline conjugate NU:UK 31, active against the refractory murine MAC15A adenocarcinoma of the colon *in vivo* [S. Jackson, J.A. Double, D.J. Mincher, A. Turnbull, M.C. Bibby, (2000), Clin. Cancer Res., **6**, 11:93] has been shown to inhibit both DNA topoisomerase I and topoisomerase II (α and β isoforms). This dual inhibitory activity may permit circumvention of resistance mechanisms arising from altered expression of an individual enzyme. We now report that analogues of NU:UB 31 have been synthesised and evaluated against a broad panel of human colon cancer cell lines in an attempt to define the structural requirements for aliphatic hydrophobic α-side chains including Val, D-Val, Leu and the non-proteinogenic amino acids Abu and Cha that show high sensitivity to all cell lines in the colon sub-panel of the NCI 60 cell line screen as measured by $GI_{50}$, TGI and $LC_{50}$ parameters. The pattern of sensitivity/resistance for these conjugates is in stark contrast to those of the established topo II inhibitors, doxorubicin, mitoxantrone and etoposide or the topo I inhibitor camptothecin. Colon selectivity is optimum when a three carbon or four carbon spacer moiety separates the peptide motif from the anthraquinone; colon selectivity is clearly abolished if the hydrophobic side chain is substituted by a polar group. Extending the length of the contiguous peptide motif to a tripeptide additionally confers colon resistance. Cyclic hydrophobic residues, notably proline and cyclohexylalanine (Cha) show the greatest colon selectivity. We now show that the proposed putative pharmacophore for colon selectivity also modulates the ability of the conjugates to circumvent resistance mechanisms in a panel of human and animal cell lines of well defined topoisomerase status. Evidence for the involvement of drug stablised topoisomerase cleavable complex formation in intact cells by L-Pro and L-Cha conjugates is given by immunoband depletion experiments in human HCT116 colon cells and is correlated to increased expression of p21.

A putative pharmacophore for colon selectivity in vitro is thus proposed for this atypical class of novel dual topoisomerase inhibitor and merits validation in vivo.

### P233 USE OF A NOVEL DNA MICROARRAY APPROACH TO IDENTIFY THE LOCUS OF ACTION OF A CLASS OF QUINAZOLINE-BASED ANTI-TUMOUR AGENTS

B Allan[1], LA Skelton[1], JP Clark[2], F Yafai[1], S Edwards[2], CS Cooper[2], AL Jackman[12], [1]CRC Centre for Cancer Therapeutics and [2]Molecular Carcinogenesis, The Institute of Cancer Research, Sutton, Surrey SM2 5NG

A compound development programme serendipitiously identified a class of highly potent (growth inhibitory $IC_{50}$ ~ 1nM) quinazoline-based analogues of folic acid with an unknown mechanism of action. They induce a delayed, non-phase specific cell-cycle arrest and are COMPARE negative in the NCI anti-tumour cell line screen. An ~300 fold resistant cell line (W1L2:R865) was raised by stepwise selection with one analogue, CB30865. This cell line is cross-resistant to all analogues in the class but not to other known anti-tumour agents. In an attempt to identify the *in vitro* target a novel DNA microarray approach was employed.

A cDNA library of ~7000 clones was generated with RNA from the W1L2:R865 line and used to produce microarray slides. These were probed with differentially labelled reverse-transcribed RNA extracted from W1L2:R865 cells and the parent line W1L2 cells. 26 distinct cDNAs were identified as over-expressed by a factor of three or more in the resistant cell line. Sequencing and BLAST database analysis of these clones revealed 50% map to chromosome 7, previously found by CGH to contain a large amplicon. Eight of the cDNAs from chromosome 7 represent known genes and the rest are of unknown function. Of particular interest are 9 genes (6 known, 3 unknown) located in 7q22, the region of maximum amplification.

Microarray amplification was confirmed by northern blot analysis of RNA from a panel of W1L2-derived cell lines with varying resistance levels to CB30865 or one of its more water soluble analogues. Primary candidates for further investigation were selected based on appropriate variation in the expression. There are six genes on the primary candidate list, four of which are from chromosome 7q22 and as such have been prioritised for further investigation. Preparations are underway to transfect these genes into the susceptible parent cell line in an attempt to confer resistance.

Tandem investigative approaches were also employed based on the microarray results. One gene identified is a component of the 26S proteasome, the multi-catalytic protease responsible for ~85% of all non-lysosomal protein degradation in cells, including many key cell cycle regulatory proteins. The W1L2:R865 cell line was shown to exhibit a low level of cross-resistance to the proteasome inhibitor, Lovastatin pro-drug. Furthermore, example compounds from the quinazoline series were shown to this is the growth rate limiting locus for these compounds.

Sponsored by the Cancer Research Campaign.

### P234 THE SINGLE LXXLL MOTIF IN TIP60 MEDIATES INTERACTION WITH CLASS I NUCLEAR HORMONE RECEPTORS

L Gaughan, ME Brady, DE Neal and CN Robson, Prostate Research Group, School of Surgical Sciences, University of Newcastle upon Tyne, Medical School, Framlington Place, Newcastle upon Tyne, NE2 4HH

The androgen receptor (AR), a member of the nuclear hormone receptor superfamily, plays a prominent role in prostate development and malignant transformation of prostate cells. Upon binding to androgen, the androgen receptor functions as a transcription factor, enhancing expression of target genes. A pre-requisite for nuclear hormone receptor activity is the binding of co-activator molecules to the activation function-2 (AF-2) domain of the receptor. The LXXLL motif/ NR-box, contained within a number of co-activator molecules, mediates the protein-protein interaction with nuclear hormone receptors.

We have previously demonstrated that Tip60 (*T*at-*i*nteractive *p*rotein-60kDa) enhances the transcriptional activity of the AR in LnCaP cells in a hormone-dependent manner. Tip60 contains one NR-box at its extreme C-terminus, which we hypothesised to mediate the interaction with the AR.

We show that interaction between the AR and Tip60 is hormone-dependent and requires the single NR-box of Tip60. Removal of the C-terminal fragment of Tip60, containing the LXXLL motif, abolishes AR-binding. We demonstrate that substitution of individual leucine residues of the LXXLL motif prevent AR interaction and also abolishes Tip60-mediated enhancement of AR activity, suggesting that inhibiting the formation of the Tip60-AR complex may be a possible target for reducing AR activity in the physiological context. We also demonstrate that Tip60 interacts in a hormone-dependent manner with AR, estrogen receptor-α, estrogen receptor-β and glucocorticoid receptor, but fails to bind to thyroid hormone receptor, vitamin-D receptor and retinoid-x-receptor implying that Tip60 may be principally involved in regulating class I nuclear hormone receptors, giving an indication as to how specific receptor activation can be achieved.

### P235 INFLUENCE OF RETINOID ISOMERISATION ON THE CELLULAR ACTIVITY OF 13-CIS RETINOIC ACID IN NEUROBLASTOMA

GJ Veal[1], J Errington[1], CPF Redfern[2], ADJ Pearson[3] and AV Boddy[1]. [1]Cancer Research Unit and Departments of [2]Medicine and [3]Child Health, University of Newcastle upon Tyne, Newcastle upon Tyne NE2 4HH

Retinoids can inhibit cell proliferation and induce differentiation and apoptosis in many cell types during normal development as well as in cancer cells propagated in tissue culture. Two of the most clinically useful retinoids in oncology are 13-cis retinoic acid (13-cis RA) and all-trans retinoic acid (ATRA). These isomers exhibit contrasting efficacy, toxicity and pharmacokinetics in clinical studies and have both been used in clinical trials for the treatment of neuroblastoma (NB). Recently published data have shown a significant clinical benefit of 13-cis RA in high-risk NB patients. Experiments were carried out to determine the extent of extra-and intracellular isomerisation of 13-cis RA and all-trans retinoic acid (ATRA) in neuroblastoma cell lines and to investigate the influence of isomerisation on the growth inhibitory effects of 13-cis RA and ATRA and on the regulation of expression of CRABP II and RAR-β, commonly used markers of retinoid activity.

We have quantified extra- and intracellular levels of RA isomers after incubation of a panel of NB cell lines with 13-cis RA or ATRA (10 μM) *in vitro*. Limited isomerisation of 13-cis RA or ATRA was observed in the extracellular medium, with less than 20% conversion measured over a 72 h period after retinoid incubation. By contrast, intracellular levels of ATRA determined over a 72 h period after incubation with 13-cis RA, accounted for up to 45% of the total intracellular retinoids and levels of ATRA actually exceeded those of 13-cis RA at 48 and 72 h in IMR32 cells, despite extracellular levels of 13-cis RA being 4-fold greater than those of ATRA. Comparable levels of intracellular isomerisation were not observed after ATRA incubations. Parallel cytotoxicity studies showed no differences in the sensitivity of three N-type NB cell lines to either 13-cis RA ($IC_{50}$: 11.2–13.9 μM) or ATRA ($IC_{50}$:12.9–14.4μM), despite significant differences in intracellular retinoid levels. However, a significant decrease in sensitivity to 13-cis RA ($IC_{50}$ = 137 μM), as compared to ATRA ($IC_{50}$ = 41 μM), was observed in the S-type cell line SH-S-EP. RAR-β was induced in a dose-dependent manner in SH SY 5Y cells with ATRA resulted in a greater induction of CRABP II than incubation with 13-cis RA. These results indicate either an intracellular conversion of 13-cis RA to ATRA or a selective uptake of ATRA in neuroblastoma cells and suggest that this may mediate the differential activity of 13-cis RA in neuroblastoma cell subtypes.

### P236 THE ROLE OF β3-ADRENERGIC RECEPTORS IN CANCER CACHEXIA

ST Russell[1], K Hirai[2] and MJ Tidale[1], [1]Pharmaceutical Sciences Research Institute, Aston University, Birmingham, B4 7ET, [2]Department of Obstetrics and Gynaecology, Osaka City University Medical School, Osaka 545–8585, Japan

A major contributor to the weight loss observed in cancer cachexia is the production of alipid mobilizing factor (LMF) a 43KDa glycoprotein, which has been shown to be excreted in the urine of cachectic cancer patients and induce lipolysis in isolated murine adipocytes membranes. This lipolytic activity was attenuated by low concentrations ($10^{-9}$–$10^{-7}$M) of the specific β3-adrenoceptor (β3-AR) antagonist SR59230A. LMF (250nM) was shown to produce comparable increases in intracellular cyclic AMP in CHOK1 cells transfected with the human β3-AR to that obtained with isoprenaline (1 nM). Also non-linear regression analysis of the binding of LMF to the β3-AR showed a high affinity binding site with a Kd value 78 ± 45 nM and a Bmax value (282±1 fmole mg protein$^{-1}$) comparable with other β3-AR agonists. These results suggest that LMF induces lipolysis through the β3-AR. LMF does not only induce lipolysis in white adipose tissue, but also causes the up regulation of uncoupling proteins-1 and -2 in brown adipose tissue, which have been shown to be induced by β3-AR agonists. This suggests that LMF possibly plays a role in the increased energy expenditure in cachectic cancer patients.

### P237 A COMPARISON OF THE EFFECTS OF THE ANTI-TUMOUR AND ANTI-CACHECTIC AGENT EICOSAPENTAENOIC ACID IN CANCER CACHEXIA AND ACUTE STARVATION.

AS Whitehouse and MJ Tisdale, Pharmaceutical Sciences Research Institute, Aston University, Birmingham, B4 7ET, United Kingdom

Cancer cachexia is the major cause of death in cancer patients. Muscle wasting is associated with an upregulation of the ubiquitin-proteasome system in skeletal muscle, as occurs in acute starvation The polyunsaturated fatty acid Eicosapentaenoic Acid (EPA) is effective in attenuating the increased protein degradation seen in skeletal muscle during cancer cachexia, possibly by inhibiting formation of 15-hydroxyeicosatetraenoic acid (15-HETE). To determine if similar pathways are involved in different catabolic conditions, the effect of EPA on muscle protein degradation and activation of the ubiquitin-proteasome pathway has been examined during cancer cachexia and acute fasting in mice.

Daily oral administration of EPA (2.5 g/kg) to animals bearing an experimental model of cachexia (MAC16 colon adenocarcinoma) inhibited weight loss ($P$ = 0.0016), tumour growth ($P$ = 0.0056) and proteolysis in muscle. This was correlated to a preservation of myosin ($P$ = 0.0029), inhibition of both the ‘chymotrypsin like’ proteasome activity and expression of several proteasomal subunits including 20s ($P$ = 0.0001), 19s($P$ = 0.0099) and P42 ($P$ = 0.001), but not the ubiquitin conjugating enzyme-$E2_{14k}$ ($P$ = 0.4313).

When non-tumour bearing, fasted animals receiving either nothing, vehicle (olive oil) or EPA (2.5 g/kg) were compared to non-tumour bearing non fasted controls, there was a significant reduction in total muscle breakdown as measured by tyrosine release in the soleus muscle of mice treated with EPA compared with the other two starvation groups ($P$ > 0.02 from control). The predominant proteolytic (chymotrypsin-like) activity of the β-subunits of the proteasome was elevated in gastrocnemius muscles of fasted animals with or without olive oil, but was completely attenuated in animals pretreated with EPA. Proteasome subunit expression was determined in gastrocnemius muscle by Western blotting using MCP231 antibody, which detected three α-type subunits of the 20S proteasome. There was an increase in expression of all three subunits in starved non-treated and vehicle treated controls, but proteasome expression was reduced down to that of non-fasted animals in starved animals pretreated with EPA. A similar effect was observed with anti-MSSI directed to the 19S cap of the proteasome, and P42 an ATPase subunit of the 19S regulator. However again, there was no change in expression of the ubiquitin-conjugating enzyme $E_{214k}$

These results suggest that protein catabolism in cancer cachexia and starvation is mediated by a common pathway which is inhibited by EPA. That EPA is an effective anti-tumour and anti-cachectic agent has been shown. However these results further suggest that EPA may also be effective in the attenuation of protein catabolism in other catabolic conditions.

### P238 PHASE I STUDY OF THE L-STEREOISOMER NUCLEOSIDE ANALOGUE TROXACITABINE

JS de Bono, SD Baker, J Stephenson Jnr, C Simmons, A Goetz, L Prouix, J Jolivet, M Hidalgo, DD Von Hoff, and EK Rowinsky, Institute For Drug Development, University of Texas at San Antonio, BioChem Pharma Inc. Laval (Quebec), Canada

**Purpose** To investigate the safety, toxicity profile, pharmacokinetics and activity of troxacitabine (BCH-4556), a unique L-stereoisomeric nucleoside analogue that is not a candidate for the nucleoside catabolic enzyme deoxycytidine deaminase, on a 30 minute infusion daily for five days schedule, and to recommend a dose for subsequent disease-directed studies in both minimally pretreated (MP) and heavily pretreated (HP) patients with advanced solid malignancies.

**Patients and methods** Troxacitabine was administered by the most active preclinical schedule, daily for 5 consecutive days as a 30 minute intravenous (IV) infusion every 3–4 weeks. The starting dose was 0.12 mg/m$^2$. Dose escalation with each consecutive cohort to 0.24, 0.48, 0.72 and 0.96 mg/m$^2$ was undertaken. Further dose escalation was undertaken to 1.2 and 1.5 mg/m$^2$ for HP patients and to 1.5 and 1.8 mg/m$^2$ for MP patients. Troxacitabine concentrations were analysed and pertinent pharmacokinetic (PK) parameters were related to the principal toxicities in pharmacodynamic (PD) analyses.

**Results** 39 patients received 123 courses of troxacitabine. Neutropenia and dermatologic toxicity characterised by pruritic, erythematous and generalised maculopapular-rashes precluded dose escalation above 1.8 mg/m$^2$. There was an unacceptably high incidence of cumulative severe myelosuppression at troxacitabine doses exceeding 1.5 mg/m$^2$ in HP patients and 1.8 mg/m$^2$ in MP patients. Palmoplantar erythrodysesthesia, distinct from the maculopapular rash, was observed in 4 patients. The PK behaviour of troxacitabine was linear over the dose range tested. On day 1, the disposition of troxacitabine was characterized by mean (SD) values for Vss and Cls of 60 (32) L and 161 (33) mL/min, respectively. Following the fifth day of treatment, the mean (SD) t1/2 value was 39 (63) hours and Cls was reduced by approximately 20% with a mean (SD) value of 127 (27) mL/min. For all dose levels combined, the mean (SD) accumulation ratio was 1.29 (0.29). A mean of 50% and 61% of the administered troxacitabine dose was excreted as unchanged drug in the urine during the first 24 hours following treatment on days 1 and 5, respectively. An additional 16% of troxacitabine was excreted between 24 to 48 hours after administration of the fifth dose. A significant and linear correlation was observed between estimated creatinine clearance and troxacitabine systemic clearance on day 1 ($R^2 = 0.318$, $P = 0.0002$) and day 5 ($R^2 = 0.3017$, $P = 0.0003$). More severe myelosuppression and dermatologic toxicities were associated with higher troxacitabine exposure. One partial remission was observed in a patient with metastatic choroidal melanoma.

**Conclusion** Troxacitabine can be safely administered using this schedule. Combination and phase II studies to evaluate this unique L-stereoisomeric nucleoside's anti-tumor efficacy have been initiated.

### P239 PHASE I CLINICAL AND PHARMACOKINETIC TRIAL OF VINFLUNINE: A NOVEL FLUORINATED VINCA ALKALOID GIVEN ON DAY 1 EVERY 21 DAYS.

P Fumoleau[1]; J Bennouna[1], J-P Armand[2], G Blanchot[3], F-M Delgado[3], and M Marty[4,1], René Gauducheau Cancer Centre (Nantes-F)[2]; Institut Gustave Roussy (Villejuif-F), [3]Pierre Fabre Medicament (Boulogne-F), [4]St Louis Hospital (Paris-F)

Vinflunine (VFL), is a novel vinca alkaloid obtained by semi-synthesis using superacidic chemistry. The most important structural modification is the selective introduction of two fluorine atoms at the 20′ position of vinorelbine. In human tumours xenografts, VFL showed definite antitumour activity (high/moderate) in 64% (7/11) of xenografts tested versus moderate activity with vinorelbine in 27% (3/11). This first phase 1 study was performed to determine the toxicity and PK of escalating doses (accelerated method) of VFL administered intravenously as 10 minute infusion once every 21 days. Between 12/98 and 03/2000, 31 patients with a variety of cancers (6 renal carcinoma (ca), 6 colorectal ca, 4 other gastro-intestinal ca, 4 unknown primary ca, 4 sarcoma, 3 advanced breast ca, 2 gynaecological ca, 2 NSCLC) were treated at 9 different dose levels from 30 to 400 mg/m$^2$. 3/5 patients experienced dose limiting toxicity (DLT) at 400 mg / m$^2$ (at cycle 1): 1 patient with grade (G) 4 abdominal pain, 1 patient G3 constipation and 1 transient and reversible G3 heart-failure. Other G-3–4 non-DLT were: grade 4 neutropenia (3 patients). The maximal tolerated dose was achieved at 400 mg/m$^2$ every 3 weeks. A new dose level was tested at 350 mg/m$^2$ in which I heavily pre-treated pt developed febrile neutropenia and sepsis. At the same level another patient experienced G3 dyspnoea and hypertension 5 days after the drug administration.

Pharmacokinetics was available in 30 patients. A dose proportional increase in VFL AUC was observed. Apparent elimination half life was 25.5 ± 3.9 h. Inter-individual variability was moderate (< 35%). Pharmacokinetic comparison at different cycles supported a good reproducibility on blood exposure. Pharmacokinetic-pharmacodynamic relationship was demonstrated on WBC and neutrophils. Therefore the recommended dose for further phase II development of VFL is established at 350 mg/m$^2$ every 3 weeks. VFL is a promising new agent, 3 partial responses were observed: 2 patients with advanced breast cancer (1 pt with liver involvement) and 1 pt with renal cell carcinoma. Phase II trials are already ongoing.

### P240 A PHASE I AND PHARMACOKINETIC STUDY OF VINFLUNINE GIVEN ON A WEEKLY SCHEDULE

J-B Vermorken[1], R Stupp[2], M-C Pinel[3], R Bugat[4], J-P Delord[4] and P Variol[3], [1]University Hospital, Antwerpen, Belgium, [2]Centre Pluridisciplinaire d'Oncologie, Lausanne, Switzerland, [3]Institute de Recherche Pierre Fabre, Boulogne-Billancourt, France, [4]Institut Claudius Regaud, Toulouse, France

Vinflunine (VFL) is derived from vinorebine (VRL) by novel semi-synthetic technic which result in the insertion of two fluorine atomes. Pre-clinical activity in human xenograft models has shown potential for efficacy in a panel of eleven human tumours. The current phase I study was aimed at determining the tolerability and the pharmacokinetics of a weekly dose regimen of VFL. VFL was administered as a 10 minutes infusion every week for at least 4 weeks.

A pharmacokinetic study of VFL administered on a weekly schedule was undertaken in 14 previously treated patients (melanoma: 3, mesothelioma: 2, digestive tract cancer: 3, other tumours: 6).

The MTD was reached at the entry dose level (190 mg/m$^2$) with 2/3 patients experiencing dose limiting infection (grade 4) or raised transaminases: (grade 3); an initial reduction to 150 mg/m$^2$ was still associated with dose-limiting toxicity: in 3/5 patients (neutropenia: grade 4, febrile neutropenia). At 120 mg/m$^2$, 0/6 patients experienced severe toxicity. One patient (renal cell carcinoma) entered at the second dose level achieved a minor response.

Dose proportional increases in blood concentration of VFL and its 3 metabolite peaks were seen over the range of 120–190 mg/m$^2$. While two of these metabolite peaks were eliminated between each weekly administration, $P9_{VFL}$ (4-O-deacetyl-vinflunine) was measurable at pre-dose sampling and accumulated over courses. A moderate accumulation was also observed for parent compound.

In conclusion, the MTD in previously heavily treated patients was 150 mg/m$^2$. DLT were haematological toxicities. Pharmacokinetic behaviour of parent compound and the $P9_{VFL}$ active metabolite might contribute to toxicities through its accumulation on this weekly VFL dose regimen.

The study is still ongoing in chemonaive patients in order to determine the MTD of weekly vinflunine in this sub-set of patients.

### P241 A PHASE I AND PHARMACOKINETIC STUDY OF VINFLUNINE GIVEN ON DAYS 1 AND 8 EVERY 3 WEEKS

P Johnson[1], I Judson[2], C Ottensmeier[1], A O'Donnell[2], M-C Pinel[3], C. Puozzo[3] and P Fumoleau[4], [1]Cancer Sciences Division, Southampton University School of Medicine, United-Kingdom, [2]Royal Marsden Hospital, Sutton, United-Kingdom, [3]Institute de Recherche Pierre Fabre, Boulogne-Billancourt, France, [4]Centre René Gauducheau, Nantes, France

Vinflunine (VFL) is a novel *vinca* alkaloïd produced by semi-synthetic insertion of two fluorine atoms into vinorelbine (VRL); it shows significant evidence of pre-clinical activity in human tumour xenografts.

A phase I study using treatment on D1+D8 q 3 weeks was conducted in order to evaluate the tolerability and pharmacokinetics of this dose regimen of Vinflunine.

A starting dose of 210 mg/m$^2$ was chosen on the basis of the first phase I study of a 21 day regimen, and previous experience with VRL. Sixteen patients were treated at dose levels of VFL of 210 mg/m$^2$, 190 mg/m$^2$ and 170 mg/m$^2$. The clinical results of treatment in this typical phase I patient population (primary sites: colorectal: 4, mesothelioma: 3, cervix: 2, sarcoma: 2, melanoma: 2, other tumours: 3) showed that the MTD was reached with grade 3/4 toxicity in 3/6 patients at the starting dose-level (210 mg/m2) necessitating two successive dose-reductions. Dose-limiting toxicity consisted of severe constipation and myalgia, and at the first dose reduction (190 mg/m$^2$) febrile neutropenia and constipation were recorded. At 170 mg/m$^2$, the same effects were still seen grade 3/4 in 1 of 6 patients and another patient experienced grade 3 chest pain.

The pharmacokinetics observed during this study were very similar to those seen with the 21 day regimen, except for the VFL elimination half-life. With pre-dose sampling on D8 (i.e. $T_0$ + 168 h from D1) a more accurate half-life of 39 ± 6 h was obtained. Several peaks of metabolites were observed which were rapidly cleared except $P_9$VFL, with a half-life estimated between 3 and 8 days.

In conclusion, DLT were neutropenia, constipation and myalgia. A dose level of 170 mg/m2 D1–D8 may be deliverable, but even at this level there is appreciable toxicity, and the D1–D21 schedule may be preferable for phase II trials.

### P242 VINORELBINE AND OXALIPLATIN: A NEW SCHEDULE FOR MESOTHELIOMA AND OTHER SOLID TUMOURS

PC Jeremy Steele, Jonathan Shamash, Marie T Evans, Maria L Nystrom, Nicole H Gower, Robin M Rudd, Lung and Mesothelioma Unit, Department of Medical Oncology, St Bartholomew's Hospital, London EC1

**Introduction** The incidence of malignant mesothelioma (MM) is increasing. Treatment options are limited, though recent data suggest that chemotherapy can provide effective palliation. We reported a response rate of 24% for vinorelbine 30 mg/m$^2$ weekly (J Clin Oncol 18:3912–17, 2000). Oxaliplatin has not been evaluated in MM or in combination with vinorelbine in other tumour groups. We conducted a phase II trial of vinorelbine 30 mg/m$^2$ days 1 and 8 and oxaliplatin 130 mg/m$^2$ on day 1 each 21 days in patients (pts) with MM. The major endpoint was response with secondary endpoints of toxicity, quality of life and survival.

**Results** From November 1999, 26 pts with histologically-proven MM have been treated. Pt characteristics: male 21 pts, female 5; median age 60 y (range 44–72); 13 epithelioid, 5 sarcomatous, 7 mixed, one pt awaiting subtype review. There were 6 PRs; 17 pts with SD; 3 pts progressed through therapy: response rate = 23% [95% CI = 9% to 44%]. The median number of cycles delivered = 3 (range 1–6). Grade 3/4 toxicities: neutropenia 18%; phlebitis 12%; malaise 12%; anorexia 12%; nausea and vomiting 12%; constipation 6%. Median survival has not yet been reached.

**Summary** The combination of vinorelbine and oxaliplatin has activity in MM. The toxicity is greater than that with single-agent vinorelbine. Survival data will indicate whether this combination is worth evaluating in phase III trials in MM. The doses and schedule of vinorelbine and oxaliplatin described here appears maximal and could be tested in other tumour groups.

### P243 SAFETY PROFILE OF ZD0473 IN PHASE II TRIALS OF PATIENTS WITH ADVANCED CANCERS

G Hoctin-Boes[1], J Cosaert[1], M Koehler[2], M Smith[1], [1]AstraZeneca, Alderley Park, Cheshire, UK, [2]AstraZeneca, Wilmington, DE, USA

Current platinum anticancer drug development is principally focused on circumventing tumour resistance to cisplatin/carboplatin while optimising tolerability. ZD0473 (*cis*-amminedichloro [2-methylpyridine] platinum [II]) is a new generation platinum drug that shows evidence of an extended spectrum of antitumour activity and overcomes platinum resistance mechanisms. After modification of the starting dose from 120 mg/m$^2$ to 150 mg/m$^2$, every 3 weeks in the Phase II clinical trial programme, a dose, safety and efficacy review was performed on 99 patients in 5 Phase II trials. Patients with a range of tumours were included (previously treated ovarian cancer [29], NSCLC [29], SCLC [25], or mesothelioma [10], and untreated NSCLC [6]). Overall, 278 cycles of treatment were given and 28 patients received ≥4 cycles. ZD0473 had a manageable safety profile. Consistent with Phase II data, haematological toxicity was dose limiting. Grade 3/4 haematological toxicity consisted of: thrombocytopenia (40% of patients), neutropenia (28%) and anaemia (19%). Haematological toxicity was generally more frequent and of a higher grade in ovarian cancer patients. ZD0473 had an acceptable non-haematological toxicity profile. There was no evidence of clinically relevant neuro- nephro- or ototoxicity, and no drug related deaths have occurred. Overall, this safety review demonstrates that ZD0473 has a manageable toxicity profile.

AstraZeneca acknowledges the contribution of the investigators to the Phase II clinical trial programme.

### P244 THE HUMAN UROPLAKIN IB GENE PROMOTER

J Olsburgh[1], P Selby[1], J Southgate[2], [1]ICRF Clinical Cancer Centre, Leeds LS9 7TF, [2]Dept of Biology, University of York, York YO10 5YW

The uroplakins (UP) are 4 distinct genes whose products form plaques that are unique to the luminal membrane of terminally-differentiated bladder epithelial (urothelial) cells. The UPIb gene is highly expressed in around 50% of muscle-invasive bladder cancer primaries and lymph node metastases[1]. Thus, the UPIb promoter could provide a means to target therapeutic gene expression in bladder cancer and the aim of this study was to characterise the promoter of the human UPIb gene.

A human PAC library was screened for the UPIb coding sequence. The full-length 5´UTR and the transcription start site were mapped using 5'RACE and ribonuclease protection assays. Functional characterisation of 5'flanking genomic DNA was performed by transient transfection of UPIb-luciferase reporter constructs into UPIb-expressing and non-expressing urothelial and non-urothelial cell lines of normal and tumour derivation. Co-transfection with an HSV-tk promoter construct was used in a dual luciferase assay to correct for transfection efficiencies.

Results showed that the UPIb proximal promoter is TATA-less, with multiple transcription initiation sites lying within a short (69 bp) non-coding exon 1. A short region (150 bp), located 200 bp upstream of UPIb exon 1, conferred strong transcriptional activity in a range of bladder cancer-derived cell lines. This activity was of comparable intensity to the control SV40 promoter. The same UPIb construct had weaker transcriptional activity in cultured primary human skin fibroblasts. Both longer and shorter UPIb constructs generated lower luciferase activity in all cell types.

This work suggests that a strong functional human UPIb proximal promoter has been identified that shows differential transcriptional activity in urothelial and fibroblast cell types. The definition of urothelium-specific transcription regulatory elements should find application in targeting gene therapy strategies for bladder cancer.

1. Lobban ED, Smith BA, Hall GD, Harnden P, Roberts P, Selby PJ, Trejdosiewicz LK, Southgate J. Uroplakin gene expression by normal and neoplastic human urothelium. *Am J Pathol* 1998; **153**: 1957–1967

### P245 CYTOTOXIC DRUGS COMBINED WITH ADENOVIRAL-MEDIATED INTRODUCTION OF TUMOUR SUPPRESSOR GENES ACT SYNERGISTICALLY TO DECREASE PANCREATIC CANCER CELL VIABILITY

CM Halloran, P Ghaneh, JP Neoptolemos and E Costello, Department of Surgery, Royal Liverpool University Hospital, 5th floor UCD Building, L69 3GA, Liverpool, UK

Pancreatic ductal adenocarcinoma has become a common cause of cancer related deaths in Europe and the USA. The illness has a relatively symptom free progression and by diagnosis the majority of patients have advanced incurable disease. At present surgery offers the only prospect of a long-term survival, but is feasible in few patients (around 20%). Chemotherapy increases survival for a few months only and recent trials have shown radiotherapy may be deterimental to survival in the medium term. As pancreatic cancer has a typical footprint of genetic mutations, including loss of function of the tumour suppressor genes p53 and p16$^{INK4a}$, gene therapy has been suggested as an additional treatment option. It has been demonstrated previously, by our group, that adenoviral re-introduction of wild type p53 and p16$^{INK4a}$ into pancreatic adenocarcinoma cell lines caused growth arrest and apoptosis, and reduction in tumour growth in subcutaneous tumours in nude mice. In this study we have investigated the results of combining standard chemotherapeutic drugs used for pancreatic cancer, 5-flurouracil (5-FU) and gemcitabine, with adenoviral introduction of p53 (Adp53) and p16$^{INK4a}$ (Adp16). Pancreatic cancer cell lines, mutant for p53 and p16$^{INK4a}$, were treated with either drug followed by vector or vector followed by drug. In all cases, the doses of 5-FU and gemcitabine used were singularly ineffective at reducing cancer cell number. Likewise, Adp53 or Adp16, at the multiplicities of infection (MOIs) used had sub-optimal effects. Cell viability was assessed by MTT assay. 5-FU and gemcitabine are both agents that predominantly affect 'S' phase of the cell cycle. Hence any pre-treatment of cancer cells with Adp53 or Adp16 which prematurely arrest the cell cycle in 'G1' might be expected to prevent co-operative action. Interestingly we have demonstrated that pancreatic cancer cells treated with either Adp53 or Adp16 followed by either 5-FU or gemcitabine showed a *reduction* in cell viability in keeping with an *additive* effect from the vector and drug combination. When pancreatic cancer cells were treated with 5-FU or gemcitabine and followed by Adp53 a similar *additive* effect was seen between the combinations. Surprisingly, when cells were treated with 5-FU or gemcitabine and subsequently treated with Adp16, there was an *enhanced reduction* in cell number suggesting that a *synergistic action* exists between these combinations. This was statistically significant ($P$ = 0.04 for 5-FU/Adp16 and $P$ = 0.02 for gemcitabine/Adp16). In addition, pancreatic cancer cells were treated with drug and vector combinations simultaneously: Adp16 plus 5-FU, Adp53 plus 5-FU and Adp16 plus gemcitabine all showed *synergistic* reduction in cell number after 5 days treatment ($P <$ 0.05). We have shown that all combinations of 5-FU or gemcitabine with Adp53 or Adp16 are beneficial in terms of reducing pancreatic cancer cell viability. The most crucially beneficial combinations showed synergistic co-operation in reducing cancer cell viability. It is envisaged that such use of combining standard chemotherapy with adenoviral re-introduction of wild type copies of mutated genes may become an important adjuvant treatment for pancreatic adenocarcinoma.

### P246 ISOLATION OF PEPTIDES RELATED TO CLASS I FROM TUMOUR CELL LINE WHICH HAS BEEN CORRECTED FOR MISSING CLASS I ANTIGENS VIA β2-M GENE TRANSFACTION

N Torabi-Pour, AME Nouri, R Saffie and RTD Oliver, Department of Medical Oncology, The Bart's and London Trust, London, UK

Given the difficulties of isolating tumour cytolytic T cells (CTL), and the fact that tumour specific peptide(s) may prove to be critical for enhancing anti-tumour immunity in some cancer patients we used immuno-bead purification technique in combination with high performance liquid chromatography (HPLC) approach to isolate peptide(s) from the groove of class I antigens from various biological specimens.

The results showed that:

1. Pure class I complex molecules could be prepared from extract of tumour cell lines, peripheral mononuclear cells as well as kidney and bladder tumour tissue biopsies.
2. The HPLC peptide profile differed for different specimens.
3. In the case of a bladder tumour cell line which lacked class I antigens because of a defective β2-m molecule, fully assembled class I antigens were re-expressed following the insertion of cells with the normal β2-m gene. At least 22 different peptides were isolated from the class I antigens of these cells. Tandem mass spectrometry analysis of HPLC purified peptides of these cells showed at least three identifiable peptides one octomer and two nanomers with the following amino acid sequences; L,T,P', H,L,L,S,Y and V,T,D,P,G,N,L,L,Y and L,T,D,L,G,F,L,V,Y. Prediction of class I alleles, showed that the eluted peptides had motifs of HLA-A1 and resembled the MAGE1 HLA-A1 peptide EADPTGHSY in having a D at position 3 and/or a P at position 4 and a Y at the C-terminus of the sequence. The first peptide (being an 8-mer) may have been trimmed in the preparative portion of the purification protocol (and could have started life as a 9-mer) whose putative source peptides are unknown.

These data demonstrated the feasibility of isolating and identifying class I-associated peptides from tumour specimens. Further simplification of these approaches will prove to be helpful for the future of tumour vaccine therapy for induction of anti-tumour immunity post de-bulking of accessible tumour in cancer patients following surgery.

### P247 BEHAVIOUR OF HLA RELATED PEPTIDES ISOLATED FROM A BLADDER TUMOUR CELL LINE: A FURTHER STEP TOWARD THE FUTURE OF PEPTIDE VACCINE THERAPY IN BLADDER CANCER PATIENTS

N Torabi-Pour, R Saffie and RTD Oliver, Departments of Medical Oncology, Bart's and London NHS Trust, London. UK

Results from the use of BCG for treatment of superficial bladder cancer suggest that this tumour is one of the most immunogenic tumours in man, though little is known about the antigenic determinants responsible for immune rejection. Part of the problem in identifying these determinants is the lack of T cell cellular immunoassay.

The research goal of this study is to define the biochemical and functional nature of antigenic peptides presented in the context of HLA (human leukocyte antigen) class I antigens.

The HPLC profile of nano peptides was eluted from purified HLA molecules from an in house bladder tumour cell line. Chemical sequencing of eluted nanopeptides was performed by Tandem mass spectrometry and the nanopeptides were characterised as follows: NAT 1 (VTDPGNLLY), NAT2 (LTDLGFLVY) and NAT 3 (LTDPHLLSY).

Predictions of class I alleles based on the NCBI GenBank show that eluted peptides are all motif peptides of HLA-A1. NAT 2 and NAT 3 resemble the MAGE-1A1 and human Herpes simplex virus 1 presented peptides. The putative source peptide of the first peptide (NAT 1) is the Hepatitis B virus. By monitoring the incorporation of $^{125}$I β2-m into the class I complex, it was found that the percent specific binding for dissociation of $\beta_2$-m at 37˚C was more than 40 h for HLA-A1 complexes containing the peptides NAT 1 & 3, whereas it was less than 40 h for the NAT 2.

Experimental measuring half-life ($t_{1/2}$) of $\beta_2$-m dissociation at 37˚C was about 3140/720 min for the corresponding complexes containing NAT 3 and NAT 1 respectively and about 173 min for NAT 2. These results resembled the theoretical $t_{1/2}$ measurement (Fortan program) predicting that both NAT 3 and NAT 1 should dissociate from HLA-A1 with a half-life ($t_{1/2}$) of 3125 and 810 min respectively, versus 312 min for NAT 2. Since MAGE 3 peptide (was used as a negative control) did not display enhanced binding affinity for HLA-A1 suggest that the importance role play with the specific peptides to increase the relative binding strengths of HLA-A complexes.

The data presented in this investigation demonstrate the feasibility of isolating and identifying peptides from class I antigens of a bladder tumour cell line. Although we have applied this approach on a relatively small scale in this study, broader applications can be foreseen.

### P248 A HETEROLOGOUS VACCINATION PROTOCOL STIMULATES BOTH HUMORAL AND CELL MEDIATED IMMUNE RESPONSES

A Conn, V Potter, I Spendlove, J Ramage, L Durrant and Professor J Carmichael, CRC Academic Unit of Clinical Oncology, Nottingham City Hospital, Nottingham NG5 2PB

**Aims** 105AD7 is a human anti-idiotypic antibody that mimics the tumour associated antigen CD55, which is over-expressed on colorectal carcinoma. 105AD7 has been constructed as a DNA vaccine in the form of a dimeric single chain Fv (scFv). Immune responses generated by a DNA prime and protein boost vaccination protocol have been studied in a mouse model.

**Procedures** Three 6–8 week old female balb/c mice were immunised in the quadriceps muscle with 100 μg of 105AD7 scFv DNA and CpG at weeks 0 and 2. Antisera were obtained at week 4 from tailbleeds. At week 5 mice were immunised subcutaneously with 100 μg of 105AD7 protein in incomplete freunds adjuvant. Five days later spleens were harvested and antisera obtained. Antisera were assessed by ELISA for the production of anti-anti-idiotypic antibodies against 105AD7 scFv. Splenocyte cultures were assessed for the presence of cytotoxic T cells, and CD8 positive cells were quantified by flow cytometry.

**Major Findings** There was a significant increase in anti-anti-idiotype antibody production at a 1:100 dilution for all three mice when compared to un-immunised controls. The cytotoxic T cell assay for one of the three mice showed a 30% increase in the specific lysis of P815 target cells pulsed with the $H^2K^d$ and the CDRH3 of 105AD7. Flow cytometric analysis showed there to be at least 19% CD8 positive T cells in splenocyte cultures, and this was increased by 8% in one mouse following culture with the $H^2K^d$.

**Conclusion** Stimulation of both humoral and cell mediated immune responses have been shown by a 105AD7 scFv DNA prime and protein boost vaccination protocol in balb/c mice. This heterologous vaccination protocol could be translated into a clinical trial in the therapy of advanced or primary colorectal carcinoma.

### P249 MUCI-BASED cDNA VACCINES IN MURINE TUMOUR MODEL SYSTEMS

TA Plunkett, I Correa, RA Graham, J Taylor-Papadimitriou & DW Miles, ICRF Breast Cancer Biology Group, Guy's Hospital, London SE1 9RT

MUCI has been proposed as a target for tumour immunotherapy. We have developed murine tumour models to assess the potential usefulness of MUCI-based vaccines. In addition, both C57B16 and BalbC mice transgenic for human MUCI have been developed. In transgenic mice human MUCI is a self-antigen, allowing the study of both immunological tolerance and auto-immunity. Three intramuscular injections at 3 week intervals with at least 50mcg of MUCIcDNA resulted in partial protection against subsequent challenge with MUCI-expressing tumour cells in both wild type C57B16 (80% of mice alive and tumour-free at 6 weeks vs 0% controls) and BalbC mice (25% of mice alive at 6 weeks vs 0% controls). Cellular depletion studies following cDNA immunisation demonstrated that the tumour protection was dependent upon both CD4+ and CD8+ T cells. However, no MUC1-specific cytotoxic T cells (CTL) could be demonstrated prior to tumour challenge. These results suggested that CTL activation was sub-optimal following cDNA immunisation with MUCI cDNA. Recent work has demonstrated the primacy of CD4 T cells in effective tumour-specific CTL activation. We reasoned that increasing MHC class II presentation of MUCI-derived epitopes might translate into more effective MUCI-specific CTL activation and thereby more effective tumour protection. We developed a chimeric construct encoding the extracellular domain of MUCI and the transmembrane and cytoplasmic tail of Lamp1. Lamp1 is found in the MIIC, the putative site of MHC class II-loading. The MUC1/Lamp1 chimeric construct localised to vesicular intracellular compartments that also expressed wild type Lamp1. In proliferation studies using antigen-presenting cells transfected with either wild type MUCI or the MUC1/Lamp1 construct, the MUC1/Lamp1 construct generated significantly greater responses suggesting more effective MHC class II processing and presentation. However, MUCI-specific CTL could not be demonstrated in mice immunised with the MUC1/Lamp1 construct. In tumour protection studies, the MUC1/Lamp1 construct was not more effective than the wild type MUC1 cDNA. Studies were also performed in mice transgenic for human MUC1; injection with up to 100mcg cDNA at 3 week intervals did not result in tumour protection. In this study the chimeric MUC1/Lamp1 construct was not more effective than the wild type cDNA. These studies also demonstrate that although MUC1-based cDNA vaccines can protect against tumour challenge in wild type mice, they do not elicit a protective immune response in mice transgenic for human MUC1. These data suggest that alternative immunisation strategies are required to overcome immunological tolerance to MUC1.

### P250 GENETIC MODIFICATION OF HUMAN NATURAL KILLER CELLS: IMPLICATIONS FOR CANCER IMMUNOTHERAPY

CS Naylor, DE Gilham, RE Hawkins, CRC Department of Medical Oncology, University of Manchester, Paterson Institute for Cancer Research, Manchester M20 4BX

Natural Killer (NK) cells provide a cellular defence which compliments that of T-cells in so much as NK cells are activated in the absence of MHC Class I molecules on the surface of target cells. As many tumour cells down regulate MHC Class I in order to avoid T-cell immune surveillance it would be expected that these cells become targets for NK activity. Indeed, there is a growing body of evidence that supports the view that NK cells play a critical role in controlling the growth and metastatic spread of a variety of tumours. It could be predicted therefore that enhancing NK activity would be advantageous to the patient. One strategy that may maintain or enhance NK activity *in vivo*, is to use them as targets for gene therapy. Many immune effector cells have been targets for retroviral gene transfer and expression of transgenes such as cytokines and chimeric immune receptors have shown to be effective in tumour targeting experiments. However, for reasons unknown, NK cells appear refractory to retroviral uptake.

In this study we report the optimisation of retroviral transduction in freshly isolated human Natural Killer cells. Transduction efficiency was based on EGFP transgene expression and NK cell viability post-transduction. An optimised spin infection and a retronectin-aided supernatant infection were demonstrated to be more efficient at transducing isolated NK cells, whilst maintaining optimal viability. GFP expression in viable CD56+, CD3- NK cells was routinely over 20%, with individual donors ranging from 6–40%. The manipulation of primary NK cells *in vitro* is hampered by difficulties encountered in their long-term culture. NK cell proliferation peaked between 11 and 14 days with viability declining considerably by 3 weeks post-harvest. We therefore studied mechanisms of maintaining the survival of transduced NK cells *in vitro*. Reintroduction of transduced cells into an NK-depleted autologous feeder population allowed the survival of the NK cells to be extended for a further 2–3 weeks. Upon re-isolation, their cell surface phenotype and transgene expression remains largely unchanged. Although their viability can be extended, the NK population were not found to proliferate whilst being maintained in the feeder cells.

Subsequently NK cells have been transduced with a retrovirus containing a chimeric immune receptor consisting of a ScFv for a specific Tumour Associated Antigen, fused to the CD3ζ domain. FACs analysis and Western Blotting confirm successful transduction of NK cells with the chimeric immune-receptor. By reintroduction into a feeder population, viability of transduced cells can, again, be prolonged. Work is continuing to assess the functionality of the transduced cells in redirected killing assays.

**P250 Cont'd**

Although it has been demonstrated here that NK cells can be transduced and maintained for up to 6 weeks, additional factors other than cytokines, such as IL-2, and feeder cells may be required to enhance NK cells expansion in vitro. The lack of proliferation in isolated NK populations will have serious implications for their potential as targets for cancer immunotherapy. These difficulties must be overcome before NK cells, and their properties can be exploited for clinical therapies.

### P251 IMPROVING THE SURVIVAL OF GENE MODIFIED LYMPHOCYTES FOR ADOPTIVE CELLULAR THERAPY USING ANTI-APOPTOSIS GENES

JD Eaton, DE Gilham, A O'Neil & RE Hawkins. CRC Department of Medical Oncology, Paterson Institute for Cancer Research, University of Manchester

For a gene-modified adoptive cellular immunotherapy approach to be successful the prolonged in vivo survival of genetically modified effector cells is crucial. Detection of circulating gene-modified T cells following re-infusion into patients has so far required highly sensitive techniques. Whilst the exact fate of these T cells is not known, the genetic modification process, or the local tumour environment in vivo could be expected to lead to activation-induced apoptosis.

In vitro studies of CD 28 co-stimulation have shown that up-regulation of certain anti-apoptosis genes, in particular Bcl-$x_L$, promote T cell survival. We have attempted to modulate this resistance to apoptosis and improve cell survival by using retroviral genetic modification to increase expression of Bcl-$x_L$ in human peripheral blood lymphocytes. Modified cells have been identified by linking Bcl-$x_L$ expression via an internal ribosome entry site to the marker green fluorescent protein.

Jurkat cells transduced with Bcl-$x_L$ were partially resistant to Fas (CD95) antibody induced apoptosis. Subsequent in vitro assays with transduced primary human lymphocytes demonstrated that over-expression of Bcl-$x_L$ promoted the survival of lymphocytes cultured in the absence of interleukin-2, either with or without the anti-CD3. antibody, OKT3.

Furthermore, Bcl-$x_L$ over-expression in human lymphocytes delayed the onset of apoptosis induced by long-term co-culture with the HeLa and 293T tumour cell lines. These results indicate that co-expression of Bcl-$x_L$ in donor lymphocyte infusions or in conjunction with a therapeutic gene might enable the long-term survival and persistence of transduced cells in vivo thereby potentially enhancing the clinical outcome of gene-modified adoptive cellular therapy.

### P252 THE MODIFICATION OF T LYMPHOCYTES FOR THE GENE THERAPY OF CANCER

DE Gilham, A O'Neill, C Hughes, R Guest, M Lehane, N Kirillova, RE Hawkins, CRC Department of Medical Oncology, Paterson Institute, Christie Hospital, Manchester M20 9BX

The aim of this project is to produce large numbers of tumour specific lymphocytes through the introduction of constructs encoding chimeric immune receptors into primary human T lymphocytes. These receptors consist of the CD3ζ protein fused to an antigen binding domain consisting of single chain antibody fragments (scFv) specific for tumour associated antigens (TAA's). The scFv's used were specific for the carcino-embryonic antigen (CEA) and neuro-cell adhesion molecule (NCAM). Ligation of the scFv by antigen results in the clustering of the CD3ζ signalling domains resulting in downstream activation of the modified lymphocyte. In this manner, it is anticipated that the gene modified lymphocytes will target and be activated by tumours expressing the relevant TAA in the absence of the usual T cell requirement for peptide-MHC presentation on the target cell

Retroviral gene transfer methods have been used to transduce primary activated polyclonal T lymphocytes from normal donors. High level transduction was identified by the use of an EGFP marker gene expressed under the control of an IRES element downstream of the scFv:CD3ζ construct. These cells were expanded in the presence of IL-2 and the expression of the recombinant receptors confirmed by western blot. The chimeric receptors were found to homodimerise and also to heterodimerise with wild-type CD3ζ protein.

The modified lymphocytes were induced to produce interferon γ (IFNγ) when cultured in the presence of the correct protein antigen either on target cells or on purified protein. However, no IFNγ was detected when control or modified lymphocytes were cultured on target cells or antigen not recognised by the individual scFv. Furthermore, specific proliferation was observed when the anti-CEA:CD3ζ modified lymphocytes, but not the anti-NCAM:CD3ζ modified cells, were cultured on immobilised purified CEA.

Encouragingly, these modified cells also displayed specific cytotoxicity against cell lines expressing the relevant target antigen. Anti-CEA:CD3ζ expressing lymphocytes killed CEA-positive colo-rectal lines including MKN45K while the anti-NCAM:CD3ζ lymphocytes killed the neuroblastoma line SK-N-BE in a dose-dependent manner. However, further studies have suggested that signalling through the scFv:CD3ζ receptor may result in activation-induced cell death (AICD) and that the modified effectors themselves may be subject to target cell induced death. AICD can be avoided to some extent by the inclusion of anti-CD28 Mabs. These studies indicate that gene modified T lymphocytes can successfully target and eliminate tumour cells. However, further critical issues, including the survival of modified cell during activity, need to be addressed for this therapy to approach a clinical setting.

### P253 DNA TRANSFER TO TUMOUR CELLS IN VITRO AND IN VIVO FOR GENE DIRECTED ENZYME PRODRUG THERAPY.

J Tupper, O Greco, M Cemazar, GM Tozer, GU Dachs, Tumour Microcirculation Group, Gray Laboratory Cancer Research Trust, Mount Vernon Hospital, Middlesex, HA6 2JR, UK

Cancer treatment is the main application of gene therapy, initially devised to correct inherited monogenic disorders. Gene directed enzyme prodrug therapy (GDEPT) involves the transfer of a gene encoding an enzyme to cells, and the subsequent administration of a prodrug, which is converted to a cytotoxin by the enzyme. Several GDEPT systems have been devised, including the novel horseradish peroxidase/indole-3-acetic acid (HRP/IAA) combination (Greco *et al.*, 2000, Cancer Gene Ther. 7, 1414). When expressed in mammalian cells, the plant enzyme HRP is able to convert IAA, a plant hormone, to a cytotoxin.

One of the main limitations of gene therapy is the efficient delivery of the DNA to target cells. Viral vectors are currently the most efficient transfection method, however they may activate oncogenes and elicit immune responses often preventing repeat administration.

The aim of this study was to assess transfection of FaDu – human nasopharyngeal squamous carcinoma – cells in vitro and in vivo with relation to the HRP/IAA GDEPT system. The marker green fluorescent protein (GFP) was encapsulated in lipofectin vesicles targeted with integrin binding peptide, and incubated with cell monolayers (Hart *et al.*, 1998, Human Gene Ther. 9, 575). This gave a transient transfection efficiency of 36–40%. Application of high voltage direct current pulses to cells and tissues can be used to facilitate DNA entry. In this case percutaneous square wave pulses significantly increased transfection efficiency in subcutaneous tumours when compared with DNA alone or in lipofectin vesicles. However, transfection levels were still below 1%.

Transfection of cell cultures with plasmid containing the HRP gene resulted in 10–14% of cells expressing the enzyme. This level of expression was sufficient to result in significant cell kill after exposure to IAA, and increased sensitivity 55-fold compared to GFP transfectants.

The HRP/IAA combination is a potentially efficient GDEPT strategy. Whilst electroporation significantly increases in vivo DNA delivery, the levels of expression obtained need to be optimised for therapeutic use.

### P254 PHYSIOLOGICALLY REGULATED GENE THERAPY FOR THE TREATMENT OF CANCER

K Binley, L Griffiths, O Kan, S Iqball, H Spearman, S Kingsman, S Naylor. Oxford BioMedica, Oxford Science Park, Oxford, OX4 4GA

Targeted transgene expression is an important feature for gene-based therapies. Although tissue-specific regulatory elements have been used for transcriptional targeting often the level of transgene expression is relatively low. Hypoxia is a common feature of solid tumours and triggers a cascade that is mediated by transcription factors binding to the Hypoxia Response Element (HRE). We have developed a novel adenoviral vector (AdOBHRE) that targets gene expression to areas of low oxygen tension (hypoxia) through an optimised HRE.

We have previously shown AdOBHRE displays hypoxia regulated reporter gene expression in a range of tumour cell lines[1] and primary cell types including macrophages[2]. The reporter gene was replaced with a therapeutic gene encoding human cytochrome P4502B6 (CYP2B6) to generate Ad.OBHREP450. Macrophages transduced with Ad.OBHREP450 demonstrate induced gene expression when infiltrated into a tumour spheroid and a significant reduction in tumour spheroid volume upon exposure to the prodrug cyclophosphamide[2].

We have assessed the potency of AdOBHREP450 by direct injection into established MDA231 human tumour xenografts on nude mice. We demonstrate significant tumour growth delay only in the presence of cyclophosphamide via activation of P4502B6 in the hypoxic tumour environment. The work presented will demonstrate the potential of targeting gene expression to ischemia/hypoxic regions using the OBHRE promoter in the context of an adenoviral vector.

1. Binley K *et al.*, Gene Therapy (1999) **6**: 1721–1727
2. Griffiths *et al.*, Gene Therapy (2000) **7**: 255–262

### P255 NITROREDUCTASES FROM *Bacillus subtilis* FOR PRODRUG ACTIVATION IN CANCER GENE THERAPY

GM Anlezark[1], T Vaughan[2], NP Michael[3], H Murdoch[1], MA Sims[1], E Fashola-Stone[1], NP Minton.[1] [1]Centre for Applied Microbiology and Research, Porton Down, Salisbury SP4 0JG; Present addresses: [2]Dept of Biology, Univ of York YO10 5YW, [3]Nycomed-Amersham, Cardiff Laboratories CF4 7YT

Nitroreductases activate diphenylcarboxamide prodrugs to form alkylating agents which crosslink DNA and kill tumour cells. This property was first demonstrated for a rat DT diaphorase with the prodrug CB 1954, and subsequently, cytotoxicity has been demonstrated *in vitro* and *in vivo* using transfected cells expressing *E. coli* B nitroreductase (NfnB) when CB 1954 and its analogues are administered. Using the sequences of NfnB and YwrO of *Bacillus amyloliquefaciens* (YwrO BAM), a novel protein that reduces CB 1954, we have identified 5 sequences encoding novel putative nitroreductases in the *B. subtilis* genome. The genes were obtained as PCR products by reverse genetics, cloned and the proteins expressed in an *E. coli* host. The recombinant proteins were purified to homogeneity by ion exchange chromatography and characterised. The proteins more highly related in sequence to YwrO BAM (YrkL, YdeQ and YwrO BS) were either inactive or very slowly active with CB 1954 and showed no activity with its mustard analogue SN 23862. However they used flavins, menadione and azodyes as substrates. In contrast, the two proteins related in sequence to NfnB (YodC and YdgI) rapidly reduced both CB 1954 and SN 23862. HPLC analysis of the reduction products of CB 1954 showed that both enzymes form the 4-hydroxylamine cytotoxic product only. Both YodC and YdgI are flavoproteins and are flavin, menadione and azoreductases, although they differ in kinetic parameters and cofactor specificity. In in vitro cytotoxicity tests enhanced cytotoxicity of CB 1954 and SN 23862 was demonstrated when V79 cells were incubated with prodrug, NAD(P)H and YodC or YdgI. The NfnB family of bacterial proteins is likely to provide further new candidates for effective nitroreductive prodrug activation by transfected tumour cells for DEPT (Directed Enzyme Prodrug Therapy) applications.

### P256 IMMUNOGENICITY OF CARBOXYPEPTIDASE G2 (CPG2) ENZYME IN ADEPT

SK Sharma, J Bhatia, RB Pedley, DIR Spencer, [1]N Minton, KA Chester, and RHJ Begent, CRC Targeting & Imaging Group, Dept. of Oncology, RF & UCMS, UCL, Royal Free Campus, Rowland Hill Street, London NW3 2PF.[1] CAMR, Porton Down, Salisbury

Pilot clinical trials with chemical conjugates of carboxypeptidase G2 (CPG2) and anti-CEA F(ab′)2 have shown that CPG2 mediated Antibody Directed Enzyme Prodrug Therapy (ADEPT) is effective, but limited by formation of human antibodies to CPG2 (HACA).

We have engineered a recombinant fusion protein of anti-CEA single chain Fv (MFE-23) fused to CPG2. This fusion protein (MFE-CP) has been expressed in bacteria (*E. coli*) and yeast (*Pichia pastoris*) with C-terminal hexa-histidine (his) or myc-his tags. Both expression systems resulted in production of stable and biologically active molecules but the *Pichia* product gave 100-fold greater yields and was glycosylated. Immunogenicity of these molecules was studied by injecting Balb/c mice with MFE-CP protein (50–100 ug/mouse, ip.) at 14–28 day intervals. Mouse anti-CPG2 antibodies (MACA) in serum were measured, by ELISA, 10 days after each immunisation. Results showed that the presence of the C-terminal tags appeared to reduce MACA but glycosylation did not. In addition, a functional mutant of MFE-CP[1] has shown a significant reduction (range 10.2–65.3%, median 45.1%) in reactivity with sera of 15 patients with post-therapy HACA.

These studies demonstrate how molecular and structural biology may be applied to reduce immunogenicity of protein therapeutics for cancer.

1. Spencer DIR, Purdy D, Minton N, Whitelegg NR, Rees AR, Begent RHJ, Chester KA (2000) Mutating ADEPT enzyme CPG2 to reduce its immunogenicity. *British Journal of Cancer* **83**: 71

Supported by the Cancer Research Campaign

### P257 BIODISTRIBUTION OF $^{123}$I-LABELLED MFE-CP FUSION PROTEIN IN LS174T XENOGRAFTED NUDE MICE

SK Sharma, H Irwin, RB Pedley, J Bhatia, J Dearling, A Flynn, KA Chester and RHJ Begent, CRC Targeting & Imaging Group, Dept. of Oncology, RF & UCMS, UCL, Royal Free Campus, Rowland Hill Street, London NW3 2PF

A biologically active recombinant fusion protein, comprising an anti-CEA single chain Fv (MFE-23) fused to the bacterial enzyme carboxypeptidase G2 (CPG2), has been constructed for use in ADEPT. This fusion protein (MFE-CP) has been expressed in *Pichia pastoris* to give high yields of a stable glycosylated product. Biodistribution studies of functional enzyme activity in nude mice bearing LS174T xenografts showed localisation of MFE-CP in tumours and rapid clearance from plasma at 6 hours (6.7% ID/g tumour and 0.0065% ID/ml plasma), resulting in tumour to plasma ratio of 1030:1. MFE-CP, in combination with prodrug gave reproducible growth delay of the xenografts, with no systemic toxicity.

MFE-CP is currently being manufactured for a clinical trial of ADEPT and it is desirable to develop a radiolabelling method to provide estimates of enzyme activity in tumour and normal tissues prior to prodrug administration. $^{123}$Iodine was selected for radiolabelling because of the favourable energy of its gamma emission for imaging patients and its comparable half-life (13.27 h) with the fusion protein clearance. MFE-CP was radiolabelled with $^{123}$iodine by either the chloramine T or iodogen method. Free and protein-bound $^{123}$iodine were separated using a PD10 column. Results showed that both iodination methods gave >90% incorporation of $^{123}$Iodine and retained CEA binding and functional enzyme activity of MFE-CP. $^{123}$I-MFE-CP was injected into nude mice bearing LS174T xenografts and biodistribution was investigated by gamma counting and by phosphor imaging. Results demonstrate the potential of $^{123}$I labelling of MFE-CP for assessing fusion protein distribution in future clinical trials of ADEPT.

Supported by the Cancer Research Campaign and the Association For International Cancer Research.

### P258 RELATIONSHIP BETWEEN MLH1 EXPRESSION AND CISPLATIN SENSITIVITY IN SMALL CELL LUNG CANCER

S Davidson[1], J Plumb[1], G Strathdee[1], R Milroy[2], R Brown[1], [1]CRC Department of Medical Oncology, University of Glasgow, Glasgow G61 1BD, [2]Department of Respiratory Medicine, Stobhill Hospital, Glasgow, G21 3UW

Deletion of chromosome 3p is common in both Small Cell (SCLC) and Non-Small Cell (NSCLC) lung cancer. The mismatch repair (MMR) protein MLH1 maps within the chromosomal region commonly deleted (3p21). Loss of MLH1 expression, and thus MMR activity, is associated with drug resistance in various tumour cell lines in vitro. We therefore wish to ask the question; does loss of MMR activity play a role in the drug resistance seen in lung cancer?

MLH1 expression has been measured by Western analysis and correlates with cisplatin sensitivity ($IC_{50}$) in a panel of seven SCLC call lines ($r^2 = 0.83$). Two related lines LS274 & LS310 have been derived from the same patient pre- and post-chemotherapy. The post treatment LS310 line is more resistant to cisplatin ($IC_{50}$: 10.6 ± 0.3 μM) than the LS274 line ($IC_{50}$: 4.5 ± 0.6 μM) and has reduced level of MLH1 protein. LS310 has the lowest MLH1 expression in the panel of SCLC lines and is the only cell line shown to have a methylated MLH1 promoter, as measured by methylation-specific PCR (MSP). However, MSP evaluates only the presence of methylation within one primer region. In order to determine whether the level of expression of MLH1 in the cell lines relates to methylation at specific sites within the gene promoter, we are sequencing the MSP amplified region to determine the precise methylation pattern.

Treatment of LS310 with the DNA demethylating agent Decitabine (2′-deoxy-5-azacytidine, 0.2 μM, 48 h) increased the cisplatin sensitivity ($IC_{50}$: 7.8 ± 1.0 μM) of this cell line. Treatment with Decitabine and a histone deacetylase inhibitor (Trichostatin A: TSA) together further increased the cisplatin sensitivity ($IC_{50}$: 5.6 ± 0.6 μM). Increased sensitivities correlate with an increase in MLH1 protein expression induced by these treatments. Decitabine and TSA appear to act synergistically since treatment of LS310 with TSA alone did not affect either cisplatin sensitivity or MLH1 expression. Treatment of LS274 with Decitabine, TSA or Decitabine + TSA had no effect on the cisplatin sensitivity or MLH1 expression level.

Our results demonstrate a spectrum of MLH1 expression within the SCLC cell line panel and that MLH1 expression correlates with cisplatin sensitivity. This mechanism of drug resistance is now being investigated in a clinical study of lung cancer patients receiving cisplatin-based chemotherapy. Furthermore our results suggest that there may be a role for the use of both a demethylating and histone deacetylase inhibitor in the reversal of acquired drug resistance of SCLC.

### P259 TAMOXIFEN-RESISTANT GROWTH IS ASSOCIATED WITH INCREASED EGFR/MAP KINASE SIGNALLING ACTIVITY IN MCF-7 BREAST CANCER CELLS

IR Hutcheson, JM Knowlden, D Barrow, CM Dutkowski, and RI Nicholson, Tenovus Centre for Cancer Research, Welsh School of Pharmacy, Cardiff University, Cardiff CF10 3XF, UK

The development of “acquired resistance” to anti-hormonal agents in breast cancer is a major therapeutic problem and major efforts are now being made to understand the mechanisms responsible for this condition. A significant inverse relationship between epidermal growth factor receptor (EGFR)/mitogen-activated protein kinase (MAPK) signalling activity and endocrine sensitivity has been reported in breast cancer. We have attempted to mimic the clinical situation by developing an antihormone-resistant cell line through long term exposure of endocrine-sensitive MCF-7 breast cancer cells to tamoxifen. In the present study we have characterised the expression and activation levels of EGFR/MAPK signalling components in both tamoxifen-sensitive (wild-type) and -resistant (TAM-R) cell lines. The role played by the EGFR/MAPK signalling pathway in the growth of these cell lines was assessed using an EGFR-selective tyrosine kinase inhibitor, ZD1839 ‘Iressa’ 1 μM), and a selective MEK inhibitor, PD098059 (50 μM). Under basal conditions there was a 2–3 fold increase in both EGFR and c-erbB2 mRNA and protein expression in TAM-R compared to wild-type MCF-7 cells, whereas, comparable levels of c-erbB3 and c-erbB4 mRNA and protein were expressed in both cell lines. Immunoprecipitation/WB studies demonstrated that, under basal conditions, all four c-erbB receptor types were found to exist as pre-formed heterodimers in both cell lines. However, only TAM-R cells demonstrated measurable levels of basal activation of EGFR/c-erbB2 and EGFR/c-erbB3 receptor dimers. There was no expression of phosphorylated c-erbB2/c-erbB3 dimers in either cell line suggesting that an EGFR-selective ligand was responsible for the activation of TAM-R cell c-erbB receptors. Activation levels of MAPK/extracellular-regulated kinase1/2 (ERK1/2) were also found to be higher in TAM-R compared to wild-type MCF-7 cells with immunocytochemical staining showing localisation within the nucleii. Treatment of TAM-R cells with ZD1839 prevented both receptor heterodimer and ERK1/2 activation and strongly inhibited cell growth. PD098059 had no effect on levels of phosphorylated EGFR and c-erbB2 but greatly attenuated activation of ERK1/2 and inhibited TAM-R growth to a similar degree to ZD1839. Although both agents abolished ERK1/2 activity in wild-type MCF-7 cells they had little effect on cell proliferation. These results demonstrate that our tamoxifen-resistant MCF-7 breast cancer cell line basally expresses increased total and activated levels of EGFR, c-erbB2 and ERK1/2 and that signalling via this EGFR/MAPK pathway is responsible for the growth of TAM-R but not wild-type MCF-7 cells.

‘Iressa’ is a trademark of the AstraZeneca Group of companies

### P260 CAVEOLIN EXPRESSION IN TAMOXIFEN-SENSITIVE MCF-7 BREAST CANCER CELL LINES

NPB Thomas[1,2], IR Hutcheson[1], D Barrow[1], CM Dutkowski[1], L Campbell[2], M Gumbleton[2] and RI Nicholson[1], [1]Tenovus Centre for Cancer Research and [2]Pharmaceutical Cell Biology Group, Welsh School of Pharmacy, Cardiff University, Cardiff CF10 3XF, UK

Use of the antihormone tamoxifen has proved to be an effective treatment for breast cancer, however, initially-responsive tumours eventually relapse due to the development of antihormone resistance. Tamoxifen-resistant tumour growth has been shown to be associated with elevated levels of epidermal growth factor receptor (EGFR) and activation of the associated mitogen activated protein kinase (MAPK) signalling pathway. The components of this signalling cascade are believed to be localised within caveolae and may be negatively regulated by the principal component of caveolae membranes, caveolin-1. It has recently been proposed that hyper-activation of the MAPK cascade, as a consequence of caveolin-1 down-regulation, may be a critical event in the oncogenic transformation of some cell lines. To investigate the potential role of caveolin-1 in the development of tamoxifen resistance we have studied the relationship between caveolin-1 and the EGFR/MAPK signalling pathway in tamoxifen-sensitive (wild-type) and -resistant (TAM-R) MCF-7 breast cancer cell lines. Caveolin-1 and EGFR mRNA and protein expression was measured at four time points: day 1, 4, 7 and 10 in the absence or presence of either epidermal growth factor (EGF, 10 ng/ml) and/or the specific EGFR tyrosine kinase inhibitor, ZD1839 (‘Iressa’ 1 μM). EGFR/MAPK signalling activity was assessed by measurement of phosphorylated MAPK/extracellular-regulated kinase 1/2 (ERK1/2). An inverse relationship between the basal expression of caveolin-1 and EGFR was observed in the two cell lines. Wild-type MCF-7 cells exhibited high caveolin-1 and low EGFR mRNA and protein levels, whereas, in TAM-R cells the reverse was evident. A direct correlation between EGFR expression and ERK1/2 activity was also apparent in both cell lines. Stimulation of wild-type MCF-7 cells with EGF had no effect on caveolin-1 mRNA expression but caused a small reduction in protein levels. There was no observable effect of EGF stimulation in TAM-R cells due to the low expression of caveolin-1 in this cell line. Treatment with ZD1839 significantly increased expression of caveolin-1 mRNA and protein levels in both cell lines, with the effect being far greater in TAM-R cells. These results indicate that the elevation of EGFR and activation of the MAPK pathway in TAM-R cells is associated with a decrease in basal caveolin-1 expression. Our data also suggests a negative regulatory role for the EGFR/MARK signalling pathway on caveolin-1 expression. It remains to be determined whether the increased EGFR/MAPK activity observed in TAM-R cells is the cause or the consequence of caveolin-1 down-regulation and whether the potentially beneficial effect of ZD1839 on caveolin-1 expression in these cells has a possible clinical correlate. ‘Iressa’ is a trademark of the AstraZeneca Group of companies.

### P261 ANDROGEN RECEPTOR TRANSCRIPTIONAL ACTIVITY IS DOWN-REGULATED BY HISTONE DEACETYLASE 1 AND 2

L Gaughan, DE Neal and CN Robson, Prostate Research Group, School of Surgical Sciences, University of Newcastle upon Tyne, The Medical School, Framlington Place, Newcastle upon Tyne, NE2 4HH

The mechanisms involved in the transition from androgen-dependent to androgen-independent prostate cancer remains largely undefined. The androgen receptor, a member of the steroid hormone receptor family, is thought to play an important role in the development of both androgen-dependent and -independent prostatic malignancy. The androgen receptor is an androgen-dependent transcription factor which activates expression of numerous androgen-responsive genes. Histone acetyltransferase (HAT)-containing proteins have been shown to increase activity of nuclear hormone receptors by eliciting histone acetylation, which facilitates promoter access to the transcriptional machinery. Conversely, histone deacetylases (HDACs) have been identified which reduce levels of histone acetylation, thereby returning active genes to a quiescent state.

It has recently been demonstrated that the AR is directly acetylated resulting in increased transcriptional activity. We have recently shown that Tip60 (Tat-interactive protein-60 kDa), a MYST protein-family member, is a *bona fide* co-activator protein for the AR. Here we show that Tip60 mediates direct acetylation of the AR. Using an AR mutant lacking this acetylation site, we demonstrate, in transient transfection studies, that Tip60 is incapable of enhancing AR-mediated transcriptional activity suggesting that direct Tip60-mediated acetylation of the AR is a requisite for AR-associated gene expression.

Both HDAC1 and HDAC2 specifically reduced transcriptional activity of wild-type AR, and in immunoprecipitation experiments, the AR was shown to interact with both HDAC1 and HDAC2. We also show in transfection experiments that Tip60 mediates reversal of HDAC-mediated effects, facilitating AR activity. These results suggest that the cellular status of HDAC and HAT proteins may influence physiological activity of the AR; an imbalance of which may lead to dysregulated AR function and cellular transformation.

### P262 ENHANCEMENT OF DOXORUBICIN INDUCED G2 ARREST AND APOPTOSIS BY AN INHIBITOR OF THE PI3K SURVIVAL PATHWAY

LSM McCarragher[1], BL Brown[1] & PRM Dobson[2], Institute of Endocrinology, [2]Institute for Cancer Studies, Division of Genomic Medicine, Sheffield University Medical School, Sheffield, UK

Doxorubicin (DOX) is an effective anticancer agent, used in the treatment of many cancers including those of the breast. However, current use of this drug manifests a wide range of serious side effects. The PI3 kinase-signalling pathway is a major survival pathway having a role in cellular survival and proliferation. Our aim was to investigate whether the inhibition of the PI3 kinase pathway could sensitise the human breast cancer cell line, MDA-MB-231, to the effects of DOX. Cells were treated with the specific PI3 kinase inhibitor, LY294002 at a dose (1 μg/ml) which had minimal effects on cellular growth (5–20% inhibition at 48 hours). After 45 minutes DOX (6–1000 ng/ml) was added for a further 24–96 hours. *In Situ* Nick Translation assay and Annexin-V-FITC binding were used as measures of apoptosis. We have previously shown that synergistic growth inhibition was observed at 48 hours, with lower dose of DOX (10 ng/ml) in the presence of LY294002. The present study was undertaken to analyse effects on the cell cycle and the expression of Akt, and cyclin B1 was investigated using western blotting. Cell cycle analysis showed G2 arrest from 24 hours in cells treated with doxorubicin alone and in combination with LY294002, but a synergistic enhancement of this G2 population was observed from 48 hours with the combination. *In situ* Nick Translation assay at 48 hours showed G2 cell cycle arrest, but no apoptosis. Cell cycle studies at 96 hours revealed a high sub-G0 population in cells treated with the combination and this was supported by the Annexin-V-FITC binding assay, which revealed a synergistic induction of apoptosis (>50%). Cyclin B1 was observed to be upregulated from 72 hours with the combined treatments only, with a more pronounced increase at 96 hours. There appeared to be no change in the PI3 kinase downstream effector, Akt, at 48 hours suggesting that PI3 kinase may not be fully inhibited with this low dose of LY294002. In conclusion, LY294002 appears to sensitise the breast cancer cell line MDA-MB-231 to the effects of DOX through an initial synergistic induction of a G2 cell cycle arrest at 48 hours, followed by a synergistic induction of apoptosis seen at 96 hours. These results support the use of survival signalling pathways as targets for anti-cancer treatment.

We are grateful to Yorkshire Cancer Research for funding.

### P263 THE EFFECT OF OVEREXPRESSION OF C-ERBB2 ON THE CHEMOSENSITIVITY OF A HUMAN OVARIAN CARCINOMA CELL LINE

V Smith, S Hobbs, P Workman and LR Kelland, CRC Centre for Cancer Therapeutics, Institute of Cancer Research, Sutton Surrey SM2 5NG

c-erbB2 (also known as HER2) is a 185 kDa transmembrane receptor of the EGF receptor family. It is overexpressed in about 30% of breast and ovarian cancer. Furthermore, overexpression of the protein is associated with poor prognosis and some preclinical studies have shown a correlation between high levels and resistance to chemotherapeutic drugs such as cisplatin and taxol.

The aim of this study was to investigate the possible role of c-erbB2 in mediating drug resistance in ovarian cancer. An isogenic human ovarian carcinoma cell line model (CH1) differing only in c-erbB2 status has been established by stable transfection of the gene encoding c-erbB2 status has been established by stable transfection of the gene encoding c-erbB2 and selection using puromycin. Immunoblotting studies revealed that the transfected line over-expressed c-erbB2 in comparison to empty vector controls. The sensitivity of the transfected line CH1/F405 compared its vector control (CH1/F373) – which does not express any detectable levels of c-erbB2 – was then determined to a variety of cytotoxic drugs and signal transduction inhibitors using 96 hour drug exposure and the sulforhodamine B (SRB) growth inhibition assay.

Results showed that overexpression of c-erbB2 caused an increase in sensitivity (5-fold; $IC_{50}$ in μM of 0.24 in CH1/F405 versus 1.36 in CH1/F373) to the heat shock protein (Hsp) 90 inhibitor, geldanamycin. Similarly, there was a trend towards increased sensitivity to another structurally unrelated Hsp 90 inhibitor, radicicol ($IC_{50}$ in μM of 0.23 in CH1/F405 versus 0.57 in CH1/F373). However, both lines showed similar sensitivity to the analogue of geldanamycin, 17-allyl-aminogeldanamyicin (17AAG) which is currently undergoing clinical trials ($IC_{50}$ in μM of 0.58 for CH1/F405 and 0.65 for CH1/373). Western blot analysis after exposure to 5 μM 17AAG for 24 hrs showed a decrease in c-erbB2 protein levels in the transfected line.

In contrast, the overexpression of c-erbB2 conferred resistance to some agents. 2.5-fold resistance was observed to emodin (a tyrosine kinase inhibitor which blocks c-erbB2 tyrosine kinase activity), 4-fold resistance to a PI3 kinase inhibitor, LY294002 and 2-fold resistance to cisplatin. No difference in $IC_{50}$ was observed after exposure to taxol, the cyclin dependent kinase inhibitor flavopiridol, a tyrosine kinase inhibitor of the epidermal growth factor receptor, PD153035, a MEK inhibitor, UO126 and a farnesyl transferase inhibitor, R115777.

These results show that high levels of c-erbB2 induced resistance to the PI3 kinase inhibitor LY294002 and cisplatin. In contrast, overexpression led to increased sensitivity to the Hsp90 inhibitor geldanamycin.

### P264 THE GROWTH INHIBITORY EFFECT OF ZD0473, A NEW GENERATION PLATINUM DRUG, IS NOT INFLUENCED BY MISMATCH REPAIR PROFICIENCY

RJ Sun[1], M Edelman[1], TC Stephens[2], PF Hausner[1], [1]University of Maryland Greenbaum Cancer Center, Baltimore, MD, USA, [2]AstraZeneca, Alderley House, Cheshire, UK

A significant fraction of primary cancers are functionally mismatch repair deficient. Additional cancers acquire mismatch repair deficiency in the course of chemotherapy and/or radiation usually due to selection of cancer cell clones with a methylated promoter of hMLH1. Mismatch repair deficiency provides cancers not only with a low grade resistance against cisplatin, methylating agents, and some other chemotherapeutic agents and radiation, but with an increased genomic instability as well. Thus, cytotoxic agents, which do not select for mismatch repair deficiency are preferable. We tested a new generation platinum derivative, ZD0473 developed to overcome platinum resistance, for differential growth inhibitory activity against mismatch repair deficient and proficient cells using the comparative growth assay. This assay is based on co-culture of two HCT116 colon cancer cell line derivatives, one mismatch repair deficient and permanently labelled with the green fluorescent protein and the other mismatch repair proficient and labelled with the yellow fluorescent protein. Co-cultures were exposed to the studied drugs for 72 hours and the relative growth suppression was evaluated. Similarly to oxaliplatin, and contrary to cisplatin, ZD0473 showed no significant preferential toxicity against mismatch repair proficient cells. ZD0473 inhibits *in vitro* the growth of mismatch proficient and deficient cells similarly.

### P265 ROLE OF MULTI-DRUG RESISTANCE (MDR) PHENOTYPE AS A PREDICTIVE MARKER OF RESPONSE TO CHEMOTHERAPY AND AS A PROGNOSTIC MARKER IN CARCINOMA BREAST

A Srivastava[1], SR Chaudhary[1], MC Mishra[1], O Coshic[1], SN Das[2] and A Goyal[1], [1]Dept. of Surgery, [2]Dept of Biotechnology, All India Institute of Medical Sciences, New Delhi-110029, India

Development of tests to predict response to chemotherapy will help to accurately select patients who may or may not benefit from such therapy. P-glycoprotein(Pgp) expression has been strongly associated with aggressive biologic behaviour, poor treatment response and poor outcome in many tumors.

**Objectives** a) To carry out sequential assessment of MDR gene expression before and after first cycle of neoadjuvant chemotherapy and to evaluate its role as a predictive marker for breast cancer response. b) To evaluate the role of MDR gene expression as a prognostic marker for local and systemic recurrences in patients undergoing post-operative adjuvant chemotherapy for breast cancer.

**Methodology** Group A included 26 patients of biopsy/FNAC proven breast cancer with locally advanced stage(IIIA, IIIB). They were studied for the multidrug resistant gene/Pgp expression before neoadjuvant chemotherapy (day 0) and after one cycle of chemotherapy (day 21). Chemotherapy responses were clinically assessed after three cycles of chemotherapy using the UICC criteria. Group B included 21 patients of early breast cancer(stage I,II) undergoing modified radical mastectomy/conservative surgery followed by adjuvant chemotherapy with CAF/CMF. Tumor tissues taken from the specimen after surgery was analysed for Pgp expression. Follow up information on local and systemic recurrence was recorded. We tried to evaluate the role of Pgp expression as a prognostic marker for local and systemic recurrence in these patients. Flowcytometric analysis of Pgp was done by fluoroscence activated cell staining, (FAC-SCAN) from the tissues taken by FNAC/operative specimen. Relation of MDR protein to known prognostic factors e.g lymph node status, menopausal status was assessed in both groups.

**Results** Group A:-Clinically after three cycles of chemotherapy 10 patients (38.5%) had complete response (CR), 9(34.6%) had partial response (PR) and 7 patients (26.2%) had no response (NR). The mean Pgp expression on day 0 (before chemotherapy) and day 21 (after one cycle of chemotherapy) in patients with CR were 18.73%(SD-19.9) and 10.97%(SD-18.52) respectively (difference not statistically significant on Kruskal-Walls H test). The mean expression on day 0 and day 21 in patients with PR were 34.38%(SD-30.5) and 27.2%(SD-38.4) respectively (difference not statistically significant). The mean expression on day 0 and day 21 in patients with NR were 45.82%(SD-19.8) and 45.94%(SD-35.94) respectively (difference not statistically significant).

Group B:-Mean Pgp expression in T1, T2, and T3 were 6.2%, 33.8% and 19.2% respectively. In 1 year follow up, only 1 patient developed systemic recurrence. There was no significant correlation between Pgp expression on both the days with systemic metastasis, lymphnode status and menopausal status in patients of both groups.

**Conclusions** Pgp may be a useful marker in predicting the response to neoadjuvant chemotherapy for breast cancer. No comment is possible on the association of Pgp expression and local and systemic recurrence due to the short followup.

### P266 RM175, A NOVEL RUTHENIUM (RU$^{II}$) ORGANO-METALLIC COMPLEX: PATTERNS OF RESISTANCE IN VITRO AND IN VIVO

RE Aird[1], J Cummings[1], R Morris[2], AA Ritchie[1], PJ Sadler[2], DI Jodrell[1], [1]ICRF Medical Oncology Unit, Western General Hospital, Edinburgh, EH4 2XU, UK, [2]Department of Chemistry, University of Edinburgh, Edinburgh, EH9 3JJ, UK

RM175, $[(\eta^6C_6H_5C_6H_5)RuCl(H_2NCH_2CH_2NH_2\text{-}N,N)]^+PF_6$, is a lead compound in a series of novel ruthenium (Ru$^{II}$) organo-metallic complexes which show activity in the human ovarian cancer cell line A2780. RM175 has an $IC_{50}$ value of 6μM compared to 0.5μM for cisplatin and 6μM for carboplatin. (Cummings *et al*, 2000, Clin Cancer Res, 6, 4494s). In the present study the activity of RM175 was evaluated in drug-resistant sub-clones of A2780: cisplatin resistant A2780cis cells (obtained from ECACC) and multidrug resistant $2780^{AD}$ cells (gifted by Prof. Ozols, USA). $2780^{AD}$ cells exhibit a classic multidrug resistant phenotype due to over expression of *mdrl* and to a lesser extent *mrp*. Cross-resistance was observed in $2780^{AD}$ (38-fold) but co-incubation in these cells with verapamil (50μM) reduced the degree of resistance to only 3-fold, strongly suggesting active efflux of the compound by P170. A2780cis cells are 10-fold resistant to cisplatin but no cross resistance was observed in A2780cis when treated with RM175. A2780cis cells do not over express *mdrl* or *mrp*. In the absence of increased expression of drug transporters, we looked at the level of expression of the mismatch repair protein, hMLH1 as a possible mechanism of resistance to cisplatin in these cells. Western blotting showed that hMLH1 is expressed in the parental line, but not in the A2780cis cells suggesting that damage caused by RM175 is not recognised by mismatch repair. When tested against A2780 xenograft RM175 (25 mg/kg i.p., days 1 & 5) showed statistically significant antitumour activity vs. control tumour on days 4, 7 and 9 ($P$ = 0.003). The compound was also evaluated against the cisplatin-resistant A2780cis xenograft, in parallel with cisplatin. RM175 showed significant activity in this xenograft (days 6, 8, 11 & 13) compared to both the control ($P$ = 0.02) and the cisplatin treated group ($P$ = 0.03). Cisplatin showed no significant activity against the A2780cis xenograft.

Therefore RM175 has demonstrated activity in a platinum resistant human ovarian cancer model both in vitro and in vivo.

### P267 IDENTIFICATION OF GENES ASSOCIATED WITH INTERFERON RESISTANCE IN MALIGNANT MELANOMA

DP Jackson[1], R Drake[1], TJ Hay[1], JM Smith[1], PJ Selby[1], PM Patel[1], [1]ICRF Clinical Cancer Centre, St James's University Hospital, Leeds, LS9 7TF

Interferon α is used in the treatment of malignant melanoma, but is of benefit in only a limited proportion of patients. In addition to the clinical variation in response, there is heterogeneity in the response of melanoma cell lines to the anti-proliferative effects of interferon α *in vitro*. The mechanism of interferon resistance in melanoma is unknown, and currently there is no reliable method of predicting response either clinically or *in vitro*.

The aim of this work is to identify genes associated with interferon resistance that may provide information as to the mechanism of resistance, and act as markers of potential utility in predicting interferon response and directing therapy.

We have shown that two melanoma cell lines, characterised as sensitive and resistant to the anti-proliferative effects of IFN-α have intact and functional interferon signalling via the JAK/STAT pathway, suggesting that resistance must be mediated through components either downstream or additional to this pathway.

We have used a method of cDNA subtractive hybridisation to identify differences in interferon regulated gene expression between these two cell lines.

A number of genes were identified that showed differential regulation by interferon. These included a protein tyrosine phosphatase, previously associated with cellular transformation, that was expressed constitutively at a higher level in the resistant cell line relative to the sensitive cell line, and which was down regulated by interferon α in both cell lines. This pattern of expression is consistent with a role for this gene in negative regulation of the interferon anti-proliferative response. The regulation of this phosphatase by interferon α is a novel observation.

Further analysis of differential gene expression between these cell lines has been performed using cDNA microarray technology. This has yielded a wealth of information, and has identified a number of genes that are up or down regulated by interferon α specifically in either the sensitive or resistant cell line.

Genes identified by these techniques will be put forward as candidate markers of interferon response and screened in a panel of established and primary melanoma cell lines.

### P268 BUTYRATE INDUCES TERMINAL DIFFERENTIATION AND APOPTOSIS IN CELL LINES DERIVED FROM ORAL SQUAMOUS CELL CARCINOMAS

A Hague, KE Shefford, DM Marland and SJ Thavaraj, Dept of Oral & Dental Science, University of Bristol, Lower Maudlin Street, Bristol, BS1 2LY, UK

Since malignant keratinocytes can be defective in terminal differentiation, agents that can reactivate terminal differentiation pathways and/or induce apoptosis would be valuable therapeutic tools for oral cancer. Sodium butyrate is a potent differentiation agent that induces terminal differentiation in normal skin keratinocytes. In this study, we questioned whether malignant oral keratinocytes would be induced to undergo differentiation or apoptosis in response to butyrate. In keratinocytes, terminal differentiation is thought to be distinct from apoptosis, and defects in terminal differentiation pathways could result in the induction of apoptosis by butyrate as a result of conflicting signals. In two cell lines derived from oral squamous cell carcinomas, butyrate inhibited cellular proliferation and induced both apoptosis and terminal differentiation. Butyrate treated cells had increased numbers of cells with G2 DNA content. Consistent with cell cycle arrest, the mitotic index decreased and p21 Waf-1 was induced (0.5 mM to 6 mM). By contrast, levels of p53 were reduced. Concentrations of 2 mM and above induced cellular flattening and increased cell size and expression of the cornified envelope protein, involucrin. This was accompanied by a reduction in colony forming efficiency after 72 hours of treatment, even if the cells were subsequently allowed to recover for 24 hours in butyrate-free medium. These results suggest that at least some of the tumour cells retain the ability to undergo terminal differentiation in response to butyrate. However, lower concentrations of sodium butyrate (0.5 and 1 mM) induced primarily growth inhibition and apoptosis. Furthermore, using concentrations that induced involucrin, apoptosis was also induced, particularly during the first 24 hours of treatment. Cells shed into the medium had apoptotic morphology and alterations in the balance of pro-apoptotic to anti-apoptotic members of the Bcl-2 family were detected, including a marked decrease in Bcl-$x_L$. The potent growth inhibitory effects of butyrate and loss of cell viability subsequent to treatment highlight the potential of butyrate analogues as therapeutic tools for oral cancer.

### P269 A PHASE II STUDY USING RETINOIDS AS REDIFFERENTIATION AGENTS TO INCREASE IODINE UPTAKE IN METASTATIC THYROID CANCERS

SC Short[1], G Cook[2], G Flux[2] L Vini[1] and CL Harmer[1], [1]Dept. Clinical Oncology, The Royal Marsden Hospital, Sutton, Surrey, [2]Dept. Nuclear Medicine, The Royal Marsden Hospital, Sutton, Surrey SM25PT

Radio-iodine is the most effective treatment for metastatic differentiated thyroid cancers. In some cases however, these tumours fail to take up radio-iodine and for these patients treatment options are very limited. Failure of iodine uptake is thought to occur because of de-differentiation of tumour cells, which might be reversible using re-differentiating agents. Retinoids demonstrate re-differentiating effects in a variety of cell types and increase iodine uptake in thyroid tumour cells *in vitro*. The aim of this study is to assess whether oral iso-tretinoin can increase radio-iodine uptake in patients with iodine uptake negative metastatic follicular and papillary thyroid cancers.

Patients who had iodine negative metastatic papillary or follicular thyroid cancers were selected from the thyroid database at The Royal Marsden Hospital and enrolled to an open, non-randomised phase II trial. Sites of metastatic disease were assessed using CT or MRI and lack of iodine uptake was confirmed using a diagnostic radio-iodine scan prior to study entry. In eligible patients iso-tretinoin was prescribed at 1.5 mg/kg/day orally for 6 weeks. Response was assessed within 2 weeks of completing treatment with repeat radio-iodine scan. In patients who showed iodine uptake after treatment dosimetric data were collected using SPECT scanning. All patients were reviewed 2-weekly during treatment for assessment of toxicity and side effects.

The results of the first cohort of patients entered to this study between December 2000 and June 2001 will be presented. These will represent the first data showing the effect of retinoids in this patient population in the UK.

### P270 CHARACTERISATION OF THE DNA DAMAGE RECOGNISED BY A MONOCLONAL ANTIBODY RAISED AGAINST CISPLATIN-MODIFIED DNA

EL Meczes[1], ADJ Pearson[2] and MJ Tilby[1], [1]Cancer Research Unit and [2]Dept. Child Health, University of Newcastle Upon Tyne, Newcastle Upon Tyne, NE2 4HH

**Background** The widely used cancer chemotherapy drug cisplatin, is thought to exert its cytotoxic action as a result of the formation of cisplatin-DNA adducts. Cross-links between two adjacent guanines and between adjacent guanine and adenine, amount to approximately 60% and 20% respectively of all the adducts formed by cisplatin on DNA. Other types of adducts involve reaction with a single guanine or cross-linkage between two non-adjacent purines, either in the same or opposite DNA strands. To quantify the these adducts we have developed effective immunological techniques based on a monoclonal antibody (Tilby *et al*. 1991 Cancer Research **51**, 123). This antibody is being used in a widening range of clinical and experimental studies here and in other centres.

**Aims** The present study aims to confirm the hypothesis that antibody, CP9/19/1i, recognises the major cisplatin-DNA adducts.

**Methods** A series of appropriately designed oligodeoxynucleotides (oligos) and polydeoxynucleotides (polymers) were first reacted with cisplatin. The amount of bound platinum, following dialysis to remove unreacted drug, was determined by atomic absorption spectrophotometry. The ability of platinum-DNA adducts to be recognised by the antibody was determined by competitive ELISA. The concentration of competing adducts required to cause 50% inhibition (the K value), is inversely proportional to the immunoreactivity of the particular adduct(s) formed.

**Results and Conclusions** Adducts on oligos lacking guanines were, at most, very weakly immunoreactive (e.g. mean K values on oligo dT = 2.7 pmol/well, oligo dA > 200 pmol/well). Adducts on oligos containing single guanine residues were also very weakly immunoreactive (median K value = 3.3 pmol/well). Highest levels of immunoreactivity, comparable to that observed with high molecular weight DNA (K = 0.002–0.006 pmol/well), required at least two adjacent guanines interspersed by pyrimidines. However, adducts on oligo dG or poly dG were recognised less well (median K = 0.13 pmol/well). Levels of immunoreactivity were detected on platinated oligos upon which only cross-links between a guanine residue and an adenine residue could form (median K = 0.47 pmol/well), i.e. up to five hundred fold less immunoreactive than on equivalent oligos containing adjacent guanines. These results show that antibody CP9/19/1i preferentially recognises platinum-DNA cross-links formed between adjacent guanines. The data also suggest that antibody recognition is dependent not only on the platinum adduct itself, but also on the nature of the surrounding sequence. This information will be important for the interpretation of clinical and experimental data from several studies both in Newcastle and other research centres.

### P271 MECHANISMS UNDERLYING DIFFERENCES IN IMMUNOREACTIVITY OF CISPLATIN DNA ADDUCTS FORMED IN CERTAIN CANCER CELLS

A Azim-Araghi*[1], CJ Ottley[2], DG Pearson[2], and MJ Tilby[1], [1]Cancer Research Unit, University of Newcastle, NE2 4HH, [2]Department of Geological Sciences, Durham University

**Aims** To investigate the basis of differences in immunoreactivity of cisplatin-DNA adducts, previously seen in two cell lines.

**Methods** The two cell lines used were the drug sensitive small cell lung cancer line, H69, and the inherently drug resistant adenocarcinoma lung cancer line, Mor. The two lines were exposed to a range of concentrations of cisplatin (2 h), DNA extracted and adduct levels determined using a competitive ELISA technique based on a monoclonal antibody (1). The *total* amount of cisplatin bound to DNA was quantified by inductively coupled plasma mass spectrometry (ICP-MS). In contrast to the total Pt measured by ICP-MS, the ELISA method determined only immunoreactive adducts.

**Results** Platinum associated with protein contaminating the purified DNA could have caused a trivial explanation of previously seen differences in apparent adduct immunoreactivity (Table). This hypothesis was excluded by analyses in which ICP-MS, ELISA and protein assay were carried out on the same DNA samples. The amount of adducts needed to cause 50% reduction in immunoassay signal (K value) was independent of protein level in the DNA samples. Protein levels were mostly in the range of 1–5% of the total DNA. Also the level of protein contamination was not significantly different between the DNA from the two cell lines. The difference in immunoreactivity was also independent of the adduct level.

| Cisplatin Dose (μM) | K Value (fmole per well) ±SD | |
|---|---|---|
| | H69 | Mor |
| 25 | 2.0 ± 0.5 | 3.5 ± 0.4 |
| 50 | 1.8 ± 0.4 | 3.5 ± 0.8 |
| 100 | 1.1 ± 0.2 | 3.4 ± 1.1 |

Each value represents the mean of 2–4 replicates of 8 different experiments.

**Conclusion** A 2-fold change in the overall immunoreactivity indicates a considerable difference in some aspect of platinum DNA adduct structure in the Mor cells. This was due to differences in the nature of the DNA adducts, and not the different

**P271 Cont'd**

protein levels in the DNA of the two cell lines. To establish the exact nature of this difference, ion exchange chromatography (FPLC) is currently being carried out to further analyse the DNA of each line, and to determine if there are differences in the nature or relative frequencies of individual adduct structures (e.g. GG and AG intra-strand cross-links).

1. Tilby et al 1991. *Cancer Res*. **51**: 123.

This work is supported by the Cancer Research Campaign.

### P272 MECHANISTIC STUDIES AND EVALUATION OF CLINICAL MARKERS FOR PHORTRESS, A NEW AGENT FOR CLINICAL TRIALS

TD Bradshaw, HL Browne, I Hutchinson, CA Laughton, CO Leong, S O'Brien, MFG Stevens, V Trapani, AD Westwell*, Cancer Research Laboratories, School of Pharmaceutical Sciences, University of Nottingham, NG7 2RD

The lysyl amide prodrug of 2-(4-amino-3-methylphenyl)-5-fluoro-benzothiazole, Phortress, has been selected for Phase I clinical evaluation (Cancer Research Campaign). The 2-(4-aminophenyl)benzothiazole series is characterised by a number of unique antitumour properties: rapid conversion of the active species 2-(4-amino-3-methylphenyl)-5-fluoro-benzothiazole (5F 203) from the parent prodrug *in vivo* (18 mg/Kg iv, efficacious plasma levels of active drug >6 h in mice); extremely potent *in vitro* and *in vivo* activity in certain human cancer cell lines only (e.g. breast, ovarian and colon xenografts); a unique (NCI COMPARE -ve) mechanism of action involving selective uptake into sensitive cells only, binding to the Aryl hydrocarbon Receptor (AhR) followed by nuclear translocation, induction of the P450 isoform CYP1A1 leading to generation of an (as yet unidentified) reactive intermediate and formation of DNA adducts leading to cell death.

The aforementioned mechanistic scenario provides several opportunities for the development of novel biological markers for clinical trials due to begin in early 2002. In order to probe drug distribution in vivo (PET scanning), the synthesis of the $^{18}F$ radiolabelled version of 5F 203 has been accomplished. Key features of this route include the regiospecific synthesis of 2-(4-amino-3-methylphenyl)-5-bromo-benzothiazole, its conversion to a 5-trimethylstannyl analogue *via* palladium-catalysed coupling and reaction with [$^{18}F$]fluorine (P.M. Price, F. Brady and colleagues, Hammersmith Hospital, London). Detection of drug-DNA adducts through analysis of DNA extracted from treated cells by the $^{32}P$ postlabelling assay (P.B. Farmer, E.A. Martin, MRC Toxicology Unit, University of Leicester) has led to the hypothesis that the number of DNA adducts formed on dose escalation may correlate with tumour response in the clinic. In addition, AhR nuclear translocation, evident in sensitive cells only, may provide a means of measuring tumour response.

Recent efforts to understand the nature of the critical drug interaction with CYP1A1 and the identity of the resulting reactive intermediate have been undertaken since the original lead compound 2-(4-amino-3-methylphenyl)benzothiazole (DF 203) was found to undergo deactivating hydroxylative metabolism in addition to its cytotoxic effect. In the case of the fluorinated substrates, only the 5-fluoro-and 7-fluoro-DF 203 analogues appeared to thwart this deactivating hydroxylation. Frontier molecular orbital calculations on this series have been used to predict potential sites of metabolic hydroxylation and also to predict the likely nature of the electrophilic reactive intermediate.

### P273 BIOCHEMOTHERAPY FOR METASTATIC KIDNEY CANCER: THE NEED FOR RANDOMISED TRIALS

JPC Steele and RTD Oliver, Department of Medical Oncology, St Bartholomew's Hospital/The Royal London Hospital, London

**Introduction** The combination of interleukin-2 (IL-2), interferon-alpha (IFN-α), and 5-fluorouracil (5-FU) for metastatic renal cancer as originally described by Atzpodien in Hannover, Germany (*Eur J Cancer* 1993; 29 suppl 5:S6–8) has proved controversial. Other centres have had difficulty in reproducing results equivalent to Atzpodien's – though the combination appears to have clinical activity. Since 1993, 46 patients have been treated with biochemotherapy at St Bartholomew's Hospital and the London Hospital. We present the results from these patients.

**Patients and Methods** From 1993, 46 patients (pts) with advanced metastatic kidney cancer have been treated with subcutaneous IL-2 and IFN-α, and intravenous 5-FU. Eleven pts had lung metastases only; 34 pts had had a nephrectomy. Histological subtypes were available for 32 pts: 22 clear-cell adenocarcinoma, 7 papillary carcinoma, two clear-cell/sarcomatoid, and one had papillary/sarcomatoid.

**Results** There was one (2%) complete response; six (13%) partial responses; eight (17%) minor responses; seven (15%) pts had stable disease on therapy. Twenty-one (46%) pts progressed on therapy. Three patients were inevaluable for response but were included in survival analyses. Two patients with papillary carcinoma had PRs. All three pts with sarcomatoid histology progressed on therapy. All but one pt responding had previously had a nephrectomy. Intention-to-treat analysis showed a median progression-free survival of 4.7 months and an overall survival of 11.4 months. The median overall survival of pts with PR or CR was 22.8 months.

**Summary** These data confirm results from other phase II trials of IL-2/IFN-α/5FU: the overall response rate is low but a small number of pts experience prolonged progression-free survival. We still do not know whether the effect of biochemotherapy is real or reflects patient selection bias. Randomised phase III trials comparing biochemotherapy with single-agent interferon, chemotherapy or novel agents are required.

### P274 IPM CHEMOTHERAPY IN CYTOKINE-REFRACTORY RENAL CELL CARCINOMA (RCC)

J Shamash, JPC Steele, P Wilson, M Nystrom, W Ansell, RTD Oliver, Department of Medical Oncology, St Bartholomew's Hospital, London

**Introduction** Irinotecan has documented activity in renal cell lines in vivo. In several malignancies synergy has been demonstrated with either cisplatin or mitomycin. The regimen IPM (irinotecan 100 mg/m$^2$ d1 + d15, cisplatin 40 mg/m$^2$ d1 + d15 and mitomycin 6 mg/m$^2$ d1 repeated every 28 days) was given to 27 patients with RCC who had progressive disease despite interferon based cytokine therapy.

**Results** Patient characteristics were: performance status 1 (range 0–3), metastatic sites 2 (range 1–3). Patients received a median of 3.5 courses of IPM. Twenty-four patients were evaluable for response:-PR 4%, MR 29%, SD 21%, PD 46%. Thirteen out of 24 (58%) who were symptomatic improved. Median progression free interval (PFI) was 4.8 months (0.4–15.5); the median PFI for the same patients on initial cytokine therapy had been 3.8 months (0.4–21). Median overall survival following IPM was 8.0 months (0.5–19). Grade 3/4 toxicities:-neutropenia 14% thrombocytopenia 0%, diarrhoea 2%, vomiting 1%, alopecia 31% and renal dysfunction 0%. Five out of 14 patients (35%) who had progressed during cytokine therapy had measurable responses to IPM.

**Conclusions** In this cohort the response rate and PFI was comparable to that of cytokine therapy despite being used following its failure.

### P275 RADICAL CHEMORADIATION FOR SQUAMOUS CELL CARCINOMA OF THE ANUS USING A TWO PHASE TECHNIQUE WITH NO BOOST

A Melcher, C Brammer, D Sebag-Montefiore, Cookridge Hospital, Leeds, UK

**Purpose** To assess the toxicity and outcome of radical chemoradiation (CRT) for squamous cell carcinoma of the anus using a simplified radiation technique with no boost.

**Methods** between 1996 and 1999 50 patients with histologically proven squamous cell carinoma of the anus were treated. Median age was 62 (range 35–85). Staging was T1 in 2 patients, T2 in 13, T3 in 31, and T4 in 4. 38 patients were N0/1, and 12 N2/3. Radiotherapy was delivered by a 2 phase technique. Phase 1 was 30 Gy in 15 fractions to wide fields, to include primary and nodal disease and both inguinofemoral regions. Phase 2 was 20 Gy in 10 fractions, treating presenting macroscopic disease with a 2–3 cm margin, by i) parallel opposed fields if nodes involved, ii) a planned volume if anal canal involved, or iii) a direct field for disease restricted to the anal margin only. Anal bolus was used throughout, and no boost radiotherapy was given. Chemotherapy was Mitomycin C 8–12 mg/m2 day 1, and 5FU 750–1000 mg/m2 days 1–4 and 29–32.

**Results** Other than the anticipated severe skin reaction, grade 3/4 toxicity was diarrhoea in 14% and myelosuppression in 10%. 47 patients received 50 Gy as planned, whilst 18 required modification of the planned chemotherapy regime. Overall treatment time was extended to 40 or more days in only 6 patients, and 38 had no treatment interruptions for toxicity. After a median follow-up of 24 months, local control has been achieved in 80% of patients overall (93% of T1/2 tumours, and 74% of T3/4 tumours). 9 patients have died from anal cancer, 1 remains alive with disease, and 38 are currently disease free. 8 have required salvage surgery following CRT.

**Conclusion** This CRT protocol has acceptable local control and acute toxicity. It has the advantage of a continuous schedule and the use of a two phase technique reduces skin morbidity. The lower total dose of irradiation used may reduce late morbidity. This schedule appears suitable for testing within the ACT2 trial, comparing cisplatin against mitomycin C during concurrent CRT, and adjuvant cisplatin/5FU versus no further treatment following CRT.

### P276 CHEMORADIOTHERAPY FOR CARCINOMA OF THE OESOPHAGUS: EXPERIENCE OF A SINGLE INSTITUTION

E Toy, J Abraham, T Crosby, A Brewster, D Mort, T Maughan, Velindre Hospital, Whitchurch, Cardiff, CF14 2TL, UK

**Background** Over the last ten years there has been interest in the use of combined modality therapy for locally advanced oesophageal cancer.

**Methods** Since 1995, 86 non-randomised patients at Velindre Hospital have received Chemoradiotherapy (CRT) using a variety of Cisplatin based regimens: [2 cycles of Cisplatin d1 5-FU d1-4 × 2 (32 patients), 4 cycles of Cisplatin d1 + PVI 5-FU q 21 (38 patients), other Platinum regimens (16 patients) ] and radiation schedules (90% received 40–50 Gy in 15–25F) as either neo-adjuvant (CRT+S) or bi-modality (CRT) treatment outside a clinical trial. Patients selected for CRT alone included those unfit for or refusing surgery, T4 N1 or heavy nodal positivity on EUS criteria.

**Results** 86 patients, mean age 61, 48% squamous, 2% anaplastic, 50% adenocarcinomas were treated.

| | Number | Median Survival | 2 year Survival | 3 year Survival |
|---|---|---|---|---|
| CRT+S | 37 | 765 days | 54% | 43% |
| CRT | 49 | 363 days | 42% | 30% |
| Overall | 86 | 593 days | 48% | 36% |

60% of patients experienced at least one Grade 3 or 4 toxicity, including thromboembolism, myelosuppression, vomiting and dysphagia however only 30% required chemotherapy dose reduction. Initial experience was that the regimen described by Walsh et al. (1996) gave unacceptable morbidity and mortality (30% peri-operative mortality) and led to a change in clinical practice. Subsequent peri-operative mortality has been 6%. Of those in whom resection was performed pathological CR rate was 39%.

**Conclusions** Chemoradiation has considerable activity in oesophageal cancer, is well tolerated, and produces long term control comparable with surgical series. Further research is needed to determine the optimal regimen and the additional value of surgery.

Walsh T, Noonan N, Hollywood D, et al (1996) A comparison of multimodal therapy and surgery for oesophageal adenocarcinoma. *NEJM*: **335**: p. 462

### P277 A PILOT STUDY OF PREOPERATIVE COMBINED MODALITY THERAPY USING PACLITAXEL, CISPLATIN,5FU AND RADIOTHERAPY IN LOCALLY ADVANCED OPERABLE OESOPHAGEAL CARCINOMA

J Abraham, T Crosby, E Toy, A Brewster, C Askill, J Manson, G Williams, R Hardwick, T Havard, W Lewis, South Wales Upper GI Group

**Background** In patients with resectable oesophageal cancer, there is some evidence that combined modality therapy is superior to surgery alone. However the toxicity and duration of therapy are increased and the optimum regimen is yet to be established. Most previous studies have combined 5FU, cisplatin (C) and radiotherapy (RT). Paclitaxel (T), a radiation sensitiser, when used as a single agent and in combination has significant activity in the treatment of oesophageal cancer.

**Aim** To test the feasibility, efficacy and tolerability of this regimen.

**Method** From Aug. 1999-Oct. 2000, 16 patients, WHO PS 0–1, median age 49 (31–73) with T3 and/or N1 carcinoma of the middle/lower oesophagus were recruited. Patients were staged by EUS, CT and laparoscopy as 13(81%) lower oesophageal tumours, 14(88%) Adenocarcinomas and 11(69%) T3N1M0.

**Regimen** *Weeks 0 – 6*; 2 cycles induction chemotherapy (Cx) at 3 weekly intervals, T 125 mg/m2, C 60 mg/m2 and continuous infusional(CI)5FU 200 mg/m2. *Weeks 7–11*; Concurrent chemoradiation (Cx/RT) with daily RT at 45Gy in 25#, weekly T 40 mg/m2, C 30 mg/m2 and CI5FU 200 mg/m2. *Weeks 17–21*; Planned radical 2–3 stage oesophagectomy.

**Results** 16 patients have completed Cx/RT and to date 9 have had surgery. (3 awaited, 1 progressive disease, 1 cardiac deterioration, 1 declined, 1 pre-operative death). Specific pathological criteria were defined and applied to assess response. There have been 3(33%) complete pathological responses, 2(22%) minimal residual disease, 2(22%) partial responses and 2(22%) with no definite response (CR/MRD = 55%). The median survival is not yet reached.

A dose reduction of T from 175 mg/m2 to 125 mg/m2, in the induction phase was made after the first 4 patients were recruited, due to unacceptable Grade 3/4 diarrhoea and stomatitis. Since this dose modification, the incidence of Grade 3/4 toxicities are; diarrhoea 12%, stomatitis 0%, asthenia 37%, oesophagitis 37%, neutropenia 12%. 4(25%) thrombotic events have occurred, 1 resulting in a preoperative death. There have been 2(22%) post-operative deaths. 1 patient bled from the gastric conduit outside the radiation field on day 21.1 developed ARDS and subsequent multi-organ failure on day 11.

**Conclusion** This regimen is highly active in the treatment of locally advanced oesophageal carcinoma. The toxicity and duration of therapy are a concern and analysis of the quality of life data will be instructive. Comparative trials are needed to ascertain the role of combined modality therapy and define the optimum regimen. Treatment costs were supported by Bristol Myers Squibb.

### P278 A RANDOMISED PLACEBO-CONTROLLED TRIAL OF DEXAMETHASONE IN THE TREATMENT OF THE IMMEDIATE SIDE EFFECTS FROM CHEST RADIOTHERAPY FOR LUNG CARCINOMA

DLS Furniss[1], RD Jones[2], MQ Hatton[1], [1]Weston Park Hospital, Whittam Road, Sheffield. [2]Beatson Oncology Centre, Dumbouton Road, Glasgow

**Introduction** Palliative radiotherapy using hypofractionation is commonly used by clinical oncologists in the United Kingdom. Large doses per fraction however are associated with immediate side effects and have also been shown to reduce peak expiratory flow rate (PEFR) 6 hours following treatment. This study looks to see if a single dose of dexamethasone can reduce these immediate side effects and prevent the reduction in PEFR.

**Methods** Patients treated with palliative radiotherapy 17 Gy in 2 weekly fractions were randomised to receive dexamethasone, single dose 4 mg 1 hour before treatment, with their first or second radiotherapy fraction and placebo with the remaining fraction. Patients were asked to complete a questionnaire 24 hours post treatment and perform home PEFR monitoring for 72 hours post treatment. Baseline PEFR's for the patients were obtained in the 2–3 weeks prior to radiotherapy. The frequency of symptoms reported with the 2 fractions were compared using McNemars test. The PEFR's were calculated as percentages of their baseline and compared using a paired t-test.

**Results** 54 patients were recruited. 76% of patients reported one or more symptoms with their treatment. There was a statistically significant difference between the incidence of chest pain reported between the 2 fractions, with less chest pain reported in the dexamethasone fraction alone 22% vs 2% $P = < 0.01$. There was also a statistically significant difference between the incidence of rigors between the 2 fractions, 10% in the placebo fraction only vs 0% in the dexamethasone fraction only $P = <0.05$. No difference was found between the incidence of flu-like symptoms or sweating. A fall in PEFR at 4 – 6 hours post treatment was demonstrated in the placebo arm only, but the difference between the 2 groups did not reach statistical significance.

**Conclusion** Dexamethasone can reduce some of the immediate side effects of hypofractionated palliative radiotherapy for lung cancer. However we did not measure any significant difference in the fall of PEFR following treatment, leaving some concern about large fraction radiotherapy in patients whose airway patency is significantly compromised.

### P279 THE ROLE OF INTENSITY-MODULATED RADIOTHERAPY IN THE TREATMENT OF PAROTID TUMOURS

CM Bragg[1], J Conway[2] and MH Robinson[1], [1]YCR Department of Clinical Oncology and [2]Department of Radiotherapy Physics, Weston Park Hospital, Whitham Road, Sheffield, S10 2SJ

The introduction of multi-leaf collimators as a means of shaping radiotherapy treatment beams enabled a reduction in the volumes of normal tissue receiving high doses. Intensity-modulated radiotherapy (IMRT) has shown potential as a means of improving the quality of treatments by shaping the dose distribution more closely to the tumour, further sparing normal tissue compared to conventional radiotherapy treatments. In this study we investigate the ability of IMRT to improve treatment of cancer of the parotid gland.

Nine patients previously treated at Weston Park Hospital were considered in this planning study. Treatment plans were produced for each patient using 3D conformal radiotherapy (3D-CRT) and ten IMRT beam arrangements. Between 3 and 9 fields were used in the IMRT plans, which were produced using the Helios inverse planning system (Varian Oncology Systems). The IMRT plans were compared to the 3D-CRT plans in terms of dose statistics, regret scores and uncomplicated tumour control probability (UTCP).

3D-CRT and the three-four-and five-field IMRT arrangements produced similar dose distributions to the target in terms of underdosing, overdosing, dose homogeneity and conformation of the 95% isodose to the planning target volume (PTV). The seven- and nine-field IMRT plans produced significantly ($P < 0.05$) better target dose homogeneity and conformation, as well as reducing the underdosing of the target. All IMRT arrangements significantly reduced the maximum dose to critical structures, reductions of 50% (approximately 20 Gy) being achieved in the spinal cord and smaller reductions in both the contralateral parotid gland (up to 16.9 Gy) and the brain (up to 7.9 Gy). The doses to the contralateral lens were equivalent from the different treatment techniques, although small increases in the maximum doses to the ipsilateral lens (up to 3.1 Gy) were seen in six IMRT beam arrangements. The IMRT arrangements with 4,5,7, and 9 beams produced higher UTCP values than did the 3D-CRT plans, the largest absolute improvement being 9.6%.

IMRT produced better dose distributions than the 3D-CRT plans, sparing greater amounts of normal tissue while treating the tumour to the same or a better extent and therefore is a suitable technique for the treatment of parotid tumours. The best distributions were obtained from a 7-field IMRT plan, although clinically acceptable results were obtained with a 5-field plan, which is proposed as a class solution for the treatment of parotid tumours.

### P280 MINIMAL DISEASE STUDY IN RHABDOMYOSARCOMA USING REVERSE TRANSCRIPTASE-POLYMERASE CHAIN REACTION FOR THREE DIFFERENT TARGET GENES

F Sartori, M Carli, A Rosolen, Clinica di Oncoematologia Pediatrica, azienda Ospedaliera di Padova, Via Giustiniani 3, 35128 Padova, Italy

Rhabdomyosarcoma (RMS) is a soft tissue tumour of childhood that arises from cells of skeletal muscle lineage. It is a tumour whose differential diagnosis might be difficult within the group of small round blue cell tumours.

Some immunohistochemical and molecular features, including MyoD1 and myogenin are useful markers in the diagnosis of RMS. Furthermore, the alveolar subtype is often associated to the reciprocal chromosomal translocations t(2;13)(q35;q14) or t(1;13)(p36;q14), involving PAX3 and FKHR or PAX7 and FKHR genes, respectively.

We established RT-PCR protocols to specifically detect MyoD1, myogenin and PAX/FKHR transcripts in tumour tissue and bone marrow aspirates in order to define the feasibility of this approach to study minimal disseminated disease in children with RMS.

We first determined the sensitivity of the method by limiting dilutions of alveolar RMS tumour cells RH30, which tested positive for MyoD1, myogenin and PAX3-FKHR transcripts in haematopoietic cells, in vitro. Then we studied 30 tumour biopsies and bone marrow aspirates obtained at diagnosis from patients affected by RMS, mostly with advanced-stage disease, in whom at least one of the markers was expressed.

Depending on the molecular target, we found that RT-PCR analysis can detect $10^{-4}$ to $10^{-6}$ RMS tumour cells in vitro. MyoD1 RT-PCR was more sensitive than myogenin and PAX3-FKHR assays, although sensitivity varies depending on reaction conditions. Preliminary data from 20 of the 30 patients indicate a low prevalence of bone marrow involvement at diagnosis: 2 positive cases out of 20, 1 of whom was detected by microscopic analysis, as well.

In these 2 patients we determined the response kinetics of bone marrow to first line chemotherapy, with subsequent RT-PCR analyses performed at different time points. Relative sensitivity of the molecular markers studied and prevalence of minimal disseminated disease in relationship with histologic and clinical characteristics will be discussed.

This study suggests that RT-PCR assays can be useful in the staging work-up of children with RMS, for monitoring response to treatment and to detect minimal tumour contamination of bone marrow or PBSC harvests in patients undergoing intensification treatment.

### P281 DOES LONGER BREAST-FEEDING PROTECT AGAINST CHILDHOOD LEUKAEMIA AND LYMPHOMAS

Abdulbari Bener[1], Srdjan Denic[2], Sehamuddin Galadari[3], Depts. of Community Medicine[1], Internal Medicine[2] and Biochemistry[3], Faculty of Medicine, UAE University, PO Box 17666, Al Ain, United Arab Emirates.
e-mail: abener@uaeu.ac.ae

**Background and aim** The protective role of breastfeeding against childhood acute leukemia and lymphomas in uncertain. We investigated this issue in a case-controlled study.

**Methods** 117 patients with acute lymphocytic leukemia (ALL), Hodgkin's (HL) and non-Hodgkin's lymphoma (NHL), age 2–14, were retrospectively collected (15 year-period) in the United Arab Emirates. 117 controls were matched to patients for age, sex and ethnicity. The primary variable was duration of breastfeeding, and the secondary variables were environmental proxies for increased infection transmission or suspected confounders. Information was collected by telephone interview of mothers.

**Findings** Median duration of breastfeeding in patients was significantly shorter than in controls, 7 and 10 months respectively ($P < 0.0001$). Breastfeeding of 0 to 6 months duration, when compared with feeding longer than 6 months, was associated with increased odds of ALL (OR = 2.5, CI 1.2–5.3), HL (OR = 3.8, CI 0.80–18.7), NHL (OR = 4.18, CI 0.8–22.6) and overall (OR = 2.8, CI 1.5–5.1). In the patient group, there were significantly higher number of children and people per family, and patients were of a higher birth order than controls. In multivariate analysis, breastfeeding duration continues to be an independent predictor of lymphoid malignancies.

**Conclusion** Breastfeeding longer than 6 months may protect against childhood acute leukemia and lymphomas.

### P282 CONSANGUINITY AND FAMILY HISTORY OF CANCER IN CHILDREN WITH LEUKEMIA AND LYMPHOMAS

Abdulbari Bener[1], Srdjan Denic[2], Mariam Al-Mazrouei[1], Depart. of Community Medicine[1], Dept. of Internal Medicine[2], Faculty of Medicine & Health Sciences, UAE University, PO Box 17666, Al Ain, United Arab Emirates.
e-mail: abener@uaeu.ac.ae

**Background** The consanguinity rate among the nationals of the United Arab Emirates (UAE) is 50.5%. This study was designed to determine whether the rates of consanguinity and family history of cancer are higher among the families of children with lymphoid malignancy than the rates in the general population of UAE nationals.

**Methods** This case-control study comprised 117 patients with acute lymphocytic leukemia (ALL) and Hodgkin's (HL) and non-Hodgkin's lymphoma (NHL), ranging in age from 1 to 15 years, and 117 matched controls. In a telephone interview, each mother was asked to provide data regarding the biological relationship between her and her husband as well as that between both sets of grandparents; each was also asked whether any family relative had cancer and if so, of what type.

**Results** Among the 69 ALL cases, 80% of families were consanguineous and 20% were nonconsanguineous. Among the 26 NHL and 22 HL cases, each group included 3 consanguineous families, 12% and 14%, respectively. The consanguinity rates for ALL, NHL, and HL were all significantly different from the 50.5% consanguinity rate in the UAE population (all three $P$ values <0.0001). The family history of cancer was more often positive in ALL patients than in controls (odds ratio 2.30; confidence interval 1.12–4.80). Overall and for each malignancy, there was no difference in family history of cancer between consanguineous and nonconsanguineous groups of cases.

**Conclusion** The consanguinity rate in the families of patients with ALL is significantly higher and in those with NHL and HL significantly lower than that in the UAE population. The family history of cancer is more often positive among ALL cases than controls – consanguinity having no effect.

### P283 EXPRESSION OF TRUNCATED ENDOTHELIN-A RECEPTOR IS ASSOCIATED WITH MALIGNANCY

PA Berry & SA Burchill, Candlelighter's Children's Cancer Research Laboratory, ICRF Cancer Medicine Research Unit, SJUH, Leeds LS9 7TF

Endothelins (ET-1, ET-2 and ET-3) are small 21 residue peptides that activate one or both of the G-protein coupled endothelin receptors, ET-A and ET-B. ET-A preferentially binds ET-1, and ET-B binds each ligand with similar affinity. These proteins have been implicated in the regulation of proliferation, migration, differentiation and angiogenesis in a number of different cell types. We have previously reported expression of a truncated ET-A receptor missing exons three and four, the binding region for ET ligands. This receptor does not bind ET-1.

The aim of this study was to examine the expression and potential role of this truncated ET-A receptor in malignancy.

A panel of 41 tumours, 25 tumour derived cell lines, and 12 normal tissues were examined by RT-PCR for ET-A and truncated ET-A receptor expression. The tumours included 14 neuroblastomas (NBL), 9 Ewing's sarcomas (ES), and 12 additional paediatric tumours.

In 35/41 (85%) tumours full length wild-type (FLWT) ET-A receptors were expressed and 17/41 (41%) tumours expressed the truncated ET-A receptor. In 12/14 (86%) NBL FLWT ET-A was present, but only 3/12 (21%) expressed truncated ET-A receptors. All 9 ES expressed FLWT ET-A and 6/9 (67%) expressed truncated ET-A receptors. Of the other paediatric tumours examined, 9/12 (75%) expressed FLWT ET-A and 5/12 (42%) also expressed truncated ET-A receptors.

Six ES and 4 NBL cell lines expressed both FLWT ET-A and truncated ET-A receptors, and 10/15 (67%) of the other cell lines expressed FLWT ET-A and 9/15 (60%) expressed truncated ET-A receptors. All 12 normal tissues expressed FLWT ET-A, where as the truncated ET-A receptor was restricted to only 2/12 (17%) tissues (bladder and gut).

Expression of the truncated ET-A receptor appears to be up regulated in established tumour cell lines, compared to primary tumour.

In conclusion these results show the truncated ET-A receptor is more frequently expressed in tumours than in normal tissues. This suggests the ET-A receptor splice variant may play a role in the development or progression of malignancy. This may be particularly important in ES as 67% expressed the truncated ET-A receptor.

### P284 THE RELATIONSHIP BETWEEN EWS-ETS GENE REARRANGEMENTS, PROLIFERATION INDEX AND SURVIVAL IN THE EWING'S SARCOMA FAMILY OF TUMOURS

S Brownhill*[1], C Mangham[2], R Grimer[3], IJ Lewis[4] and SA Burchill[1], [1]Candlelighter's Children's Cancer Research Laboratory, and [4]Paediatric Oncology, St. James's University Hospital, Leeds, [2]Department of musculoskeletal Pathology and [3]Royal Orthopaedic Hospital, Birmingham

EWS gene rearrangements, involving the EWS gene on chromosome 22q and one of several ETS transcription factor genes have been described in over 95% of Ewing's sarcoma family of tumours (ESFT). Recent studies have suggested the fusion transcript type may be related to proliferation index and be of prognostic significance. The aims of this study were to examine ESFT for EWS-ETS gene rearrangements by RT-PCR and *in situ* RT-PCR, and to correlate these findings with proliferation index and clinical outcome. RNA from primary tumours of the Ewing's sarcoma family was amplified by RT-PCR for EWS-FLII and EWS-ERG fusion transcripts. *In situ* RT-PCR was performed on 7μm tumour sections. Proliferation was measured by immunohistochemistry for Ki67; cells with nuclear staining for Ki67 were scored as proliferating and proliferation index scored. Proliferation index (PI) = [number of Ki67 positive nuclei ÷ number of nuclei scored] × 100. EWS-FLII type 1 fusion types were detected in 21/27 (78%) of ESFT analysed. The presence of EWS-FLI1 type 1 fusion transcripts did not predict survival (Log Rank test $P$ = 0.87) or low PI (Fisher's exact test $P$ = 0.385). However, there was a significant correlation between PI and survival (Log Rank test $P$ = 0.03), tumours with a high PI (>10% cells proliferating) correlating with a poor outcome. In one ESFT both EWS-FLI1 and EWS-ERG fusion types were detected by RT-PCR, the expression pattern of the fusion types within this tumour was investigated by *in situ* RT-PCR. In conclusion, tumours with a proliferation index greater than 10 predict poor survival for children and young adults with ESFT. In this study no correlation between EWS-ETS fusion type and survival was found, furthermore there was no association between fusion transcript type and proliferation.

### P285 BASIC FIBROBLAST GROWTH FACTOR (bFGF) IS PRODUCED AND SECRETED BY TUMOURS OF THE EWING'S SARCOMA FAMILY

F Diaz-Mendez and SA Burchill, Candlelighter's Children's Cancer Research Laboratory, ICRF Cancer Medicine Research Unit, St James's University Hospital, Leeds bFGF and its receptors regulate cell growth. Their aberrant expression has been associated with tumourigenesis, increasing tumour growth and angiogenesis. The aim of this study is to examine the expression and potential role of bFGF and its receptors in ESFT.

The expression and localisation of bFGF and its receptors was examined by immunohistochemistry and Western blot. RT-PCR was used to identify specific FGF receptor subtypes. The levels of bFGF and FGF receptors in ESFT have been quantified by real-time RT-PCR.

All ESFT examined expressed bFGF, as demonstrated by immunohistochemistry and Western blotting. By immunohistochemistry, bFGF was localised in vesicles within the ESFT cell. These vesicles were particularly abundant at the cell surface. Furthermore, bFGF was detected in vesicles outside the cell body, attached to the extracellular matrix.

Expression of FGF receptors was also evident in all the Ewing's sarcoma cell lines. Immunofluorescence using an FGF receptor-1 antibody showed diffuse staining throughout the cell. The same antibody used for Western blotting confirmed the presence of FGF receptor-1. Using RT-PCR it was possible to demonstrate the presence of all four high affinity receptors in the Ewing's sarcoma cell lines studied. Two variant forms of FGF receptor-3 were detected; sequence analysis showed these variant forms to be lacking the second half of the third immunoglobulin-like loop of the extracellular domain and the transmembrane domain. In addition, the Ewing's sarcoma cell lines SK-N-MC and A673 expressed a truncated form of FGF receptor-2, missing the first immunoglobulin-like loop of the extracellular domain.

In summary, ESFT produce and secrete bFGF. bFGF associated with the extracellular matrix may play a critical role regulating the growth and metastasis of ESFT. This may in part be mediated by down-regulation of FGF receptor expression.

### P286 REAL TIME IMAGING OF APOPTOSIS IN NEUROBLASTOMA CELL LINES

M Elliott[1], B Nelson[1], MRH White[1] & HP McDowell[2]. [1]School of Biological Sciences, University of Liverpool, Life Sciences Building, Crown Street, Liverpool, L69 7ZB. [2]Royal Liverpool Children's NHS Trust, Eaton Road, Liverpool, L12 2AP

**Background** Neuroblastoma often has a poor long-term response to cytotoxic chemotherapy agents. The apoptotic response of cells to these agents is important in their success as a treatment modality. p53 has long been recognised as being important in the initiation of apoptosis in response to DNA damage. Apoptotic cells can be identified by changes in the cell membrane, mitochondria or nucleus. We have attempted to determine apoptotic rates in neuroblastoma cells in real time rather than performing assays at isolated time points.

**Methods** Using Western Blotting technique, we characterised the p53 response of neuroblastoma cell lines (SK-N-SH, SK-N-MC, IMR-32, SH-SY-5Y, SHEP and SK-N-AS) to DNA damage by etoposide and ultra-violet (UV) light. The cell lines were cultured on "Mattek" dishes and then treated with etoposide or uv light. After adding fluorescently labelled annexin V and propidium iodide (PI) to the culture media, a field of cells was then imaged for 48–72 h using a confocal laser-scanning microscope, taking images at 6 min time intervals. The equipment allowed temperature, humidity and carbon dioxide concentration to be kept constant over a long time period, maintaining the cells in a physiological environment. The timing of annexin V and PI binding was determined for each cell in the field (20–40). Apoptotic cells bind annexin V prior to binding PI and this timing permitted us to clearly differentiate apoptosis from necrosis. Using flow cytometry, large populations of cells were analysed at given time points, to confirm the microscopy findings.

**Results** A p53 response to both etoposide and UV light occurred in all cell lines apart from SK-N-AS. The degree of p53 response was maximal by 4–6 h and the maximum response was dose dependent in all cell lines. The SK-N-SH and SH-SY-5Y cell lines became apoptotic within 4 h of exposure to a DNA damaging agent. However, all of the other cell lines did not show evidence of apoptosis, but did demonstrate necrosis if a sufficient level of DNA damaging agent was used. These results were confirmed by flow cytometry analysis of the cell lines.

**Conclusions** The cell lines are known to express wild type p53 apart from SK-N-MC, which expresses mutant p53. Despite a p53 response to DNA damage, four of the cell lines were unable to become apoptotic suggesting a defect in the p53 dependent apoptotic pathway. This poor apoptotic response in cell lines may be relevant to the often poor response of neuroblastoma to cytotoxic agents used ckinically. Using real time imaging of living cells, it is possible to analyse more subtly the mechanism of cell death and makes the differentiation of apoptosis and necrosis possible.

### P287 THERAPEUTIC RESPONSES IN NEUROBLASTOMA ARE CORRELATED TO PHENOTYPE: SK.N.SH IN VITRO STUDIES

S Hankin*, and J Lawry, Institute for Cancer Studies, University of Sheffield Medical School, Sheffield, UK

Human neuroblastoma is a clinically and biologically heterogeneous childhood tumour. The selection of resistant sub-clones during chemotherapeutic treatment is the central cause of failure in disease management. Two human neuroblastoma cell lines, Kelly and SK.N.SH, have been used to monitor drug-induced cell cycle arrest and apoptosis with cisplatin, etoposide and cyclophosphamide using flow cytometry. SK.N.SH shows the coexistence of morphologically and biochemically distinctive cell types; neuronal cells (N-cells) and substrate-adherent cells (S-cells). Kelly is composed only of N-type cells. Cell cycle effects were examined by staining with the DNA intercalating agent propidium iodide (PI) and kinetic data was obtained by incorporating the thymidine analogue 5-Bromo-2-deoxyuridine (BrdU).

Both dose and time dependent increases in the number of apoptotic cells were observed with the Annexin-V assay and the terminal deoxynucleotide transferase dUTP nick end labeling (TUNEL) technique. Each morphologically distinctive cell phenotype of SK.N.SH had a specific response to the chemotherapeutic drugs. The N-phenotype cells showed classic apoptotic morphology and dose dependent cell death. The S-phenotype appeared more refractory to cytotoxic insults, and displayed morphological signs of differentiation.

**The Percentage of Apoptotic Cells at 72 Hours of Drug Incubation (TUNEL Assay)**

| Cell Line | Untreated Control | 1 μg/ml Cisplatin | 2 μg/ml Etoposide | 200 μg/ml Cyclophosphamide |
|---|---|---|---|---|
| Kelly (N-cells) | 0.60 | 10.70 | 65.17 | 29.85 |
| SK.N.SH (S-cells) | 0.10 | 0.40 | 0.14 | 0.21 |
| SK.N.SH (N-cells) | 0.14 | 45.32 | 28.97 | 38.11 |

Current work includes evaluating drug-associated changes in both cell cycle and apoptosis-related protein expression in these human neuroblastoma cell lines. The coexistence of diverse cell phenotypes provides a unique model for preclinical studies that may interpret the mixed clinical response associated with neuroblastoma.

This work is funded by Yorkshire Cancer Research.

### P288 IDENTIFICATION AND MOLECULAR CHARACTERISATION OF A *CALM:AF10* FUSION IN ACUTE MEGAKARYOBLASTIC LEUKAEMIA IDENTIFIES A NOVEL *CALM* VARIANT

LK Jones[1], B Linder[1], PJ Wu[1], M Neat[2], BD Young[2] and V Saha[1], [1]ICRF Children's Cancer Group and [2]Medical Oncology Unit, Charterhouse Square, London EC1M 6BQ

The t(10;11)(p13;q14) is a non-random translocation described in both adult and paediatric acute lymphoblastic and myeloid leukaemias. It results in the fusion of the gene *CALM*, which encodes a clathrin assembly protein, on 11q14 to the *AF10* gene, a putative transcription factor on 10p13. Here we describe for the first time, the occurrence of a *CALM-AF10* fusion in a case of acute megakaryoblastic leukaemia (FAB M7). Fluorescence *in situ* hybridisation and reverse transcriptase polymerase chain reaction were used to confirm the presence of a *CALM-AF10* fusion, together with the reciprocal *AF10:CALM* fusion. Cloning and sequencing of the amplified PCR products revealed that fusion of the *CALM:AF10* occurred at nucleotide position 1926 of the *CALM* cDNA and position 424 of the *AF10* sequence. The reciprocal transcript fused *AF10* 423 to *CALM* 1987. All samples sequenced were found to contain a novel splice variant of *CALM*, missing nucleotides 1927–2091. This new variant WT *CALM* cDNA encodes a protein lacking amino acids 594–648.

The breakpoints in *CALM* are located towards the carboxy terminal, resulting in the majority of *CALM* fused to various lengths of *AF10* in the *CALM-AF10* fusion products. CALM is a cytoplasmic protein, while AF10 can be detected in both the cytoplasm and the nucleus. Sequence analysis of AF10 has identified three putative bipartite Nuclear Localisation Signals (NLS). Using GFP-AF10 fusion constructs we have shown that the most N-terminal NLS (aa232-247) alone mediates the nuclear transport of AF10. These results were confirmed by site directed mutagenesis of the NLS consensus sequence. The described breakpoints in *AF10* in the *CALM:AF10* fusion include nt 423/424, 589/560, 883/884 and 979/980. Depending on where the breakpoints are in *AF10*, the fusion product will retain or loose some of the interactive domains of AF10. The *CALM:AF10* 424 and 590, will retain the bipartite NLS, and so localise to the nucleus. To support this data, we have confirmed by immunohistochemistry that the CALM:AF10 product localises to both the nucleus and cytoplasm in the U937 cell line. The *CALM:AF10* 883 and 979 will lack the NLS, and we therefore predict that these fusions will localise to the cytoplasm. However, as with all the *CALM:AF10* fusions, the AT-hook, Q-rich region and leucine zipper are retained. The relatively even distribution of patients over the four described *AF10* breakpoints suggest that, irrespective of the positioning on the NLS the retention of the interactive leucine zipper is critical for leukaemogenesis.

### P289 EXPRESSION OF PAX-3 ALTERNATIVELY SPLICED TRANSCRIPTS IN HUMAN TUMOURS OF NEURAL CREST ORIGIN

C Parker, SG Shawcross[1], I Hart[1], S Kumar[2], R MacKie[3], K Sisley[4,5], and P Kumar[1], [1]Manchester Metropolitan University M1 5GD, [2]St Thomas' Hospital, London SE1 7EH, [3]Manchester University M13 9PT, [4]Royal Infirmary Glasgow G4 0SF, [5]Royal Hallamshire Hospital, Sheffield S10 2JF

The developmental gene, PAX-3, is expressed in the early embryo in developing muscle and elements of the nervous system, including the brain. Recent studies have demonstrated PAX-3 over-expression in both embryonal and alveolar rhabdomyosarcomas (ERMS and ARMS), while we have demonstrated its expression in melanomas and small cell lung carcinomas (SCLCs). Alternative transcripts encoding two additional exons downstream of exon 8 have been identified. The original isoform has been designated PAX-3c, the isoform which includes one additional exon, PAX-3d, and that encoding two extra exons, PAX-3e. In the study here, RT-PCR was used to screen for the alternative transcripts, PAX-3c, PAX-3d and PAX-3e, in human and murine melanoma cell lines, primary human ocular melanomas, secondary human cutaneous melanomas and human SCLC cell lines. Sets of primers for each of the isoforms were designed and their specificity confirmed by sequence analysis of the products.

All 8 primary human ocular melanomas expressed PAX-3c and 3d, and 7/8, PAX-3e. Two human ocular melanoma cell lines expressed PAX-3d, 1/2 cell lines, PAX-3c, (in both cases expression was barely detectable), but neither, PAX-3e.

In the secondary human cutaneous melanomas, 14/17 showed PAX-3c expression, 16/17, PAX-3d and 15/17, PAX-3e. The 8 human cutaneous melanoma cell lines all expressed PAX-3c, PAX-3d and PAX-3e.

In the case of SCLC, 4/5 cell lines expressed PAX-3d, but none PAX-3c or PAX-3e.

In summary, we found all three transcripts expressed in every cutaneous melanoma cell line and the majority (>80%) of cutaneous melanoma tumour tissues. All of the ocular melanomas expressed PAX-3c and PAX-3d, with over 80% expressing PAX-3e. There was less predictable expression of the PAX isoforms in ocular melanoma cell lines. SCLC cell lines appear to express only PAX-3d. In addition, a comparison of the different amplicon staining intensities for any one sample, indicated that PAX-3d was the predominant transcript, followed by PAX-3c. PAX-3e was often barely detectable and may be expressed at very low levels. The role of the three isoforms in tumourigenesis and cancer progression is currently under investigation.

### P290 THE GEOGRAPHICAL DISTRIBUTION OF CHILDHOOD LEUKAEMIA AND LYMPHOMA IN NORTH WEST ENGLAND

RJQ McNally[1], DP Cairns[1], OB Eden[1], AM Kelsey[1] & JM Birch[1], [1]Royal Manchester Children's Hospital, Manchester M27 4HA

The objective was to explore the geographical distribution of leukaemia and lymphoma in cases included in the Manchester Children's Tumour Registry, 1977 to 1996. Cases of acute lymphoblastic leukaemia (ALL), acute myeloid leukaemia (AML), Hodgkin's disease (HD) and non-Hodgkin's lymphoma (NHL) were allocated to the electoral ward in which they were resident at the time of diagnosis. Observed (O) and expected (E) numbers of cases and standardised morbidity ratios were obtained by electoral ward. Poisson regression was used to examine the relationship between incidence rates and (i) population density; (ii) ethnic composition of the ward; and (iii) deprivation index for the ward (Carstairs and Townsend), based on decennial census data. ALL had a particularly heterogeneous distribution; higher rates were associated with: (i) wards with high population density (O/E = 1.13 for highest quintile; O/E = 0.87 for lowest quintile; $P = 0.06$); (ii) wards with a greater proportion of Pakistanis (O/E = 1.12 for highest quintile; O/E = 0.87 for lowest quintile; $P = 0.04$); wards with a greater proportion of Indians (O/E = 1.09 for highest quintile; O/E = 0.71 for lowest quintile; $P = 0.07$); and wards with a greater proportion of Afro-Caribbeans (O/E = 1.09 for highest quintile; O/E = 0.81 for lowest quintile; $P = 0.08$). Conversely, lower rates for ALL were associated with wards with a greater proportion of white Europeans (O/E = 0.71 for highest quintile; O/E = 1.11 for lowest quintile; $P = 0.04$). (iii) There was no association between incidence of ALL and deprivation, as measured by the Townsend and Carstairs indices. For AML, HD and NHL, no associations were found between incidence and any of these factors. This may be due, at least in part, to the small numbers of cases. Results differ from other recent findings, linking higher incidence of ALL with increased affluence, but are consistent with a role for infection in the aetiology of ALL. Increased opportunity for exposure is associated with higher population density, which may also explain the differences in incidence found by ethnic group stratification.

### P291 CLUSTERING IN NON-CENTRAL NERVOUS SYSTEM CHILDHOOD SOLID TUMOURS

RJQ McNally[1], AM Kelsey[1], FE Alexander[2], OB Eden[1], & JM Birch[1], [1]Royal Manchester Children's Hospital, Manchester M27 4HA, [2]Dept of Public Health Sciences, University of Edinburgh, Edinburgh EH8 9AG

The objective was to analyse space-time clustering of non-central nervous system solid tumours in children included in the Manchester Children's Tumour Registry, 1954 to 1998. Knox tests for space-time interactions between cases were applied with fixed thresholds of close in space, <5 km and close in time, <1 year apart, to determine whether there are more pairs of cases occurring in close proximity than expected by chance. Tests were repeated replacing geographical distance with distance to the Nth nearest neighbour to adjust for population density. N was chosen such that the mean distance was 5 km. Data were also examined by a second order procedure based on K-functions to allow for multiple testing and boundary effects. Reference points in time and space were dates and addresses at birth and diagnosis respectively. The methods showed statistically significant evidence of space-time clustering for sarcomas and Wilms' tumour, based on time and location at birth, but not time and location at diagnosis. For sarcomas, $P = 0.02$ using the geographical distance version of the Knox test and $P = 0.004$ using the nearest neighbour version. Using the geographical distance version of the K-function method $P = 0.006$ and $P < 0.001$ using the nearest neighbour version. For Wilms' tumour the clustering was particularly apparent amongst children aged 0–4 years: $P = 0.007$ using the geographical distance version of the Knox test and $P = 0.006$ using the nearest neighbour version. Using the geographical distance version of the K-function method $P = 0.004$ and $P = 0.01$ using the nearest neighbour version. There was no evidence of space-time clustering amongst cases of neuroblastoma, peripheral neuroectodermal tumours, Hodgkin's disease and non-Hodgkin's lymphoma. These are the first results to demonstrate the presence of space-time clustering for childhood sarcomas and Wilms' tumours. Results are consistent with environmental exposure hypotheses relating to locations pre-natally or peri-natally.

### P292 *MLL* CLEAVAGE AND REARRANGEMENT FOLLOWING TOPOISOMERASE 2 INHIBITOR THERAPY IN PAEDIATRIC t-AML

A Ng[1,2], GM Taylor[1] & OB Eden[2], Immunogenetics Laboratory, St. Mary's Hospital[1], Manchester M13 0JH, Academic Unit of Paediatric Oncology, Royal Manchester Children's Hospital[2], Manchester M27 4HA, UK

Treatment related acute myeloid leukaemia (t-AML) is the most serious complication of effective DNA topoisomerase 2 (topo 2) inhibitor therapy used for childhood cancers and rarely, for non-malignant conditions. Most cases are characterised by abnormalities involving the Mixed Lineage Leukaemia (*MLL*) gene on chromosome 11q23, and a poor outcome. Although frequent scheduling and high cumulative dosage of topo 2 inhibitors have been shown to associate with the development of t-AML, individual risk prediction remains ill defined. Molecular analyses of *MLL* abnormalities during topo 2 inhibitor treatment may improve our understanding on the process of secondary leukaemogenesis and contribute to identifying patients at risk of t-AML.

Genomic DNA from serial blood and bone marrow samples were obtained from a boy who received etoposide-based therapy for primary, non-familial haemophagocytic lymphohistiocytosis (HLH) but subsequently developed a t-AML 6 months post treatment. Cleavage and rearrangement of the *MLL* gene were studied using Southern blotting and hybridisation with a *MLL* cDNA probe and real-time autoradiography. Panhandle PCR was also used to detect *MLL* rearrangement in bone marrow specimens obtained during his primary therapy and at the onset of t-AML. *MLL* cleavage was identified in the bone marrow 3 months after the start of etoposide treatment and rearrangement of the gene was found at the time of t-AML diagnosis. The abnormalities were not detected in his marrow at the presentation of HLH or in any of his serial blood samples.

These findings provide further support that the *MLL* gene is implicated in the molecular pathogenesis of paediatric t-AML associated with epipodophyllotoxin therapy. They also suggest the potential of *MLL* cleavage and rearrangement as biomarkers for treatment-related leukaemogenesis, and that bone marrow is the most informative material for such detection. The relationship between *MLL* cleavage and rearrangement, their actual roles in leukaemogenesis and risk prediction for t-AML warrant further investigations.

### P293 ANALYSIS OF EPENDYMOMAS USING COMPARATIVE GENOMIC HYBRIDISATION

M Carter[1], FM Ross[1], JC Nicholson[1,5], R Allibone[4], V Balaji[2], JA Crolla[1], RJ Gilbertson[3], RH Perry[3], DA Walker[4], DW Ellison[2,3], [1]Wessex Regional Genetics Lab., Salisbury; Universities of [2]Southampton, [3]Newcastle and [4]Nottingham; [5]Addenbrookes Hospital, Cambridge

**Background and objectives** Ependymomas are the third most common CNS tumours in childhood, and account for 9–12% of CNS neoplasms in all age groups. However, the prognosis for cases not completely excised is poor and the underlying biology of their development and progression is poorly understood. The few studies published to date have suggested that specific chromosomal abnormalities may be associated with the development of a significant proportion of these tumours. We set out to screen a large series of intracranial and spinal ependymomas for genetic imbalances, and to correlate these with histology and clinical behaviour.

**Methods** Comparative genomic hybridisation (CGH) was used to detect chromosome imbalances on 86 ependymomas from 77 patients, of which 22 were children under 16, treated at one of three UK centres (Newcastle, Nottingham, Southampton). Cases were first analysed without reference to histology or clinical features, and were then divided up according to anatomical location, histology and age at presentation.

**Results** Chromosomal imbalances were detected in tumours from 63/77 patients (82%). The majority involved entire chromosomes or chromosome arms, many showing patterns of gains suggestive of intermediate ploidy. Of previously reported abnormalities in ependymoma, the most frequent findings were gain of 1 q, seen in 13 cases (17%), and loss of 22 in 20 (26%). Whereas 1 q gain was seen mainly in posterior fossa tumours and was restricted to those with classic and anaplastic histology, loss of 22 was rarely observed in tumours from this location and their histology was predominantly classic or myxopapillary. In contrast to previous studies, loss of 6q was found in only 6 cases (8%) and in only one child. Out of 7 tumours biopsied at presentation and relapse, 4 revealed imbalances and 3 of these demonstrated progression of abnormalities at relapse.

**Conclusions** Chromosomal imbalance is common in ependymoma and patterns of abnormality are emerging that are associated with histology or location. Further studies are needed to establish the prognostic significance of these abnormalities.

### P294 EWING'S TUMOUR: NOVEL RECURRENT CHROMOSOMAL ABNORMALITIES DEMONSTRATED BY MOLECULAR CYTOGENETIC ANALYSIS OF SEVEN CELL LINES AND ONE PRIMARY CULTURE

Danielle C Shing[1,2], Catherine A Morley-Jacob[1,3], Ian Roberts[2], Elisabeth Nacheva[4], and Nicholas Coleman[2,5], [1]Joint first authors, [2]Department of Pathology, University of Cambridge, Tennis Court Road, Cambridge, United Kingdom, [3]Department of Paediatrics, Addenbrooke's Hospital, Hills Road, Cambridge, United Kingdom, [4]Department of Haematology, Cambridge Institute for Medical Research, Hills Road, Cambridge, United Kingdom, [5]Department of Histopathology, Addenbrooke's Hospital, Hills Road, Cambridge, UK

Conventional cytogenetics has led to the identification of the primary t(11;22) (q24; q12) translocation in the Ewing's family of tumours, and to the demonstration of certain recurring secondary aberrations that may contribute to neoplastic progression. Other important cytogenetic abnormalities may previously have been overlooked due to the limited resolution of chromosome banding. We have applied the molecular cytogenetic techniques of SKY, M-FISH and CGH to the characterisation of seven Ewing's tumour cell lines and one primary culture. These complementary techniques have enabled us to produce a detailed description of the karyotypes of the cell lines and to demonstrate recurring numerical and structural abnormalities. In particular, we have identified a novel, unbalanced translocation involving chromosomes 16 and 17 in three of eight samples, including the primary culture. The unbalanced translocation was associated with CGH evidence of loss of 16 q and 17p, copy number imbalances that were seen in five and four of the eight samples respectively. Recurrent breakpoints at 16p11.2, 16q11.1, 17p11.2 and 17q11.2 were identified. Our findings indicate that chromosomes 16 and 17 should be investigated further in the search for genes involved in the development of Ewing's family tumours.

### P295 THE EWINGS SARCOMA EWS/FLI-1 PROTEIN UP-REGULATES THE STROMELYSIN AND PARP PROMOTERS IN NIH-3T3 CELLS.

BTS Ubhi, DR Rainey, AR Craig & DM Meredith. Molecular Medicine Unit, University of Leeds, Beckett St, Leeds, LS9 7TF

**Introduction** The EWS/Fli-1 protein results from the translocation of the EWS gene on chromosome 22 with the Fli1 gene on chromosome 11. This translocation is present in 95% of cases of Ewings sarcoma and results in the production of a chimeric transcription factor. Fli-1 is a member of the ETS family of transcription factors with a DNA binding domain. The role of EWS/Fli-1 in the pathogenesis of Ewings sarcoma is thought to involve the up regulation of specific target genes. These genes are as yet unknown although several workers have proposed genes such as PARP, WAF-1, PIM-1 and stromelysin. Since stromelysin (one of the matrix metalloproteinases) has a putative role in tissue invasion and metastases and since PARP (poly ADP ribose polymerase) is expressed at very high levels in Ewings sarcoma cells we sought to characterise these genes in terms of the responsiveness of their promoter regions to both EWS/Fli-1 and Fli-1. In addition we investigated the effect of TEL, a putative tumour suppressor gene on trans-activation of the Stromelysin promoter by EWS/Fli-1 and Fli-1.

**Method** The EWS/Fli-1 type 1, Fli1 and TEL genes were cloned into the pcDNA3.1 mammalian expression vector. Similarly, two reporter plasmids were constructed based on the pG1-3 firefly luciferase reporter system with the Stromelysin promoter region and the PARP promoter region cloned in. The EWS/Fli1 and Fli-1 vectors were co-transfected with the reporter vector into NIH-3T3 cells using Superfect, a liposomal transfection system. Cells were harvested at 24 hours and assayed for luciferase activity. The study was repeated with the addition of the TEL expression vector. All experiments were done in triplicate and repeated with similar results.

**Results** EWS/Fli-1 demonstrated a 2–3 fold increase in activity at the PARP promoter in contrast to a 3–4 fold increase with Fli-1. EWS/Fli1 also showed up to a 10-fold increase in activity at the Stromelysin promoter in contrast to a 20-fold increase in activity seen with Fli1 alone. TEL caused a 5% decrease in EWS/Fli-1 trans-activation and a 17% decrease in Fli-1 trans-activation.

**Conclusion** The results suggest that both Stromelysin and PARP promoters are direct targets of both EWS/Fli-1 and Fli1 with early up regulation of both promoters after co-transfection. This supports previous data seen using RDA although a greater response was seen with Fli-1 than in previous studies. TEL resulted in a decrease in trans-activation particularly with Fli-1.

### P296 EXPRESSION AND ASSOCIATION OF THE HUMAN TRITHORAX AND POLYCOMB GROUP OF PROTEINS

Pei Jun Wu[1], Julia I Bardos[2], Denise Sheer[3] Paul Freemont[2], Vaskar Saha[1], [1]ICRF Children's Cancer Group, St. Bartholomew's and The Royal London School of Medicine and Dentistry, London EC1M 6BQ, [2]ICRF, Molecular Structure and Function Laboratory, [3]ICRF, Human Cytogenetics Laboratory, PO Box 123, London WC2 3PX, UK

We recently proposed a hypothesis that genes disrupted by chromosomal translocations in acute leukaemia, may have a common mechanism for leukaemogenesis, by acting on chromatin pathways regulating the *HOX* gene cascade via interactions of the trithorax (TrG) and polycomb (PcG) proteins.

The nuclear distribution of TrG (MLL) and PcG (RING1, BMI1 and HPC3) and their co-localisations in different cell lines (HeLa, U937, HT1080 and U2OS) were examined by immunofluorescent labelling with antibodies against MLL and RING1, or BMI1, or HPC. Cells were fixed and permeabilized with formaldehyde and Triton X-100, incubated with the antibody, mounted in Citifluor containing DAPI then visualised and recorded on a Zeiss fluorescent microscope. Images were analysed and 3-D reconstruction was carried out with the Zeiss LSM program. MLL displays a punctate nuclear distribution, while polycomb proteins form discrete foci in a cell line specific manner. Partial colocalisation of MLL and polycomb was evident in a small but consistent number of foci in all cell lines tested and appeared to be peri-nucleolar or at the rim of the nucleus.

To test for physical interactions between the TrG and PcG proteins, immunoprecipitations of MLL-HPC3/RING1 were carried out in U2OS cells, which were transiently transfected with GFP-tagged HPC3 or RING1, using the SuperFect transfection reagent (Qiagen). The cells were harvested after transfection and a cell extract was pre-cleared with protein G Sepharose. anti-MLL antibody was added to the extracts, and incubated with continuous mixing. Protein G Sepharose was added and the immunoprecipitate washed. After heating and removal of the protein G beads, the proteins were separated by SDS-PAGE and blotted onto nitrocellulose. The blots were probed with a monoclonal anti-GFP antibody, and the ECL chemiluminescence system (Amersham) was used for detection. However, to date we have not been able to detect any bands.

Our preliminary studies suggest that there are nuclear regions where TrG and PcG complexes aggregate together. There appears to be no physical interaction between the complexes, and the co-localisation may reflect the state of chromatin activation in these regions.

### P297 RQ-PCR STRATEGIES TO DETECT MINIMAL RESIDUAL DISEASE IN CHILDHOOD ALL

Maria Pitsiouni[1], Naina Patel[1], Lindsey K Goff[2], David Samuel[1], Susanna J Wilkes[3], Vaskar Saha[1], [1]Imperial Cancer Research Fund Children's Cancer Group, [2]Medical Oncology Unit, St. Bartholomew's and The Royal London School of Medicine and Dentistry, London EC1M 6BQ and [3]Department of Haematology, Royal London Hospital, London E1 1BB

We present our experience in the use of real-time PCR to quantify genomic and RNA targets for the detection of minimal residual disease in childhood acute lymphoblastic leukaemia (ALL). The genomic strategy involves the detection of clone specific IgH gene rearrangements and the RNA approach the detection of *ETV6-CBFA2* transcript detectable in approximately 25% of children with ALL.

**Methods** Bone marrow samples from 23 ALL patients were analysed by end point PCR using consensus primers to the FR1 region and a JH consensus primer, allowing the design of allele specific oligonucleotides. This strategy was used to underpin the design of a Real-Time PCR (RQ-PCR) using consensus probes to the FRIII and JH regions. We have also extensively evaluated the methodology for reverse-transcriptase RQ-PCR to detect *ETV6-CBFA2* fusion in 6 patients. RQ-PCR strategy employed a forward primer and probe on exon 5 of *ETV6* and a reverse primer on exon 2,3 or 4 of *CBFA2*.

**Results** Using consensus probes to the FRIII region (as opposed to the JH region) for the RQ-PCR quantification of the IgH region appears to be reliable and as sensitive ($10^{-4}$) as the end point PCR technique. With both techniques, it is possible to track disease and the majority of standard risk patients do not have detectable disease at day 15. For the RNA based analyses, we found cDNA synthesis to be most reliable using random hexamers and MMLV-RT, rTth giving occasionally skewed results and B2microglobulin as the best internal control. In comparison to genomic detection, transcripts are detectable in most patients at d30 and often upto 4 months.

**Conclusions** RQ-PCR can be used reliably to monitor disease levels in childhood ALL. It is fast, less prone to contamination and will provide a sensitivity of at least $10^{-4}$. Further analysis is required to determine whether this will be a useful tool to risk stratify patients.

